# A Short-Term High-Fat Diet Improved the Survival of Fat Grafts in Mice by Promoting Macrophage Infiltration and Angiogenesis

**DOI:** 10.3389/fcell.2022.856839

**Published:** 2022-03-17

**Authors:** Xingtang Niu, Zhuhao Lai, Xihang Chen, Feng Lu, Jianhua Gao, Yi Yuan

**Affiliations:** Department of Plastic and Cosmetic Surgery, Nanfang Hospital, Southern Medical University, Guangzhou, China

**Keywords:** fat grafting, high fat diet, macrophage, lipolysis, angiogenesis

## Abstract

**Background:** Adipose tissue is an ideal filler material that is widely used for soft tissue defects. But the low survival rate and complications associated with such grafts pose a serious challenge, which limits their clinical application. Adipose tissue is a metabolic diet-responsive tissue; however, the influence of diets on fat grafting remains ambiguous.

**Methods:** We extracted inguinal fat pads from C57/BL6 male mice, and transplanted them into the dorsal region of recipient mice (0.3 ml). Post-fat-grafting, mice (*n* = 54) were randomized into three groups, namely normal diet (ND), high carbohydrate diet (HC), and high-fat diet (HF). Structural changes were assessed by histological staining. Lipolysis activity and vascular regeneration of grafts on day 30 were analyzed using real-time polymerase chain reaction, immunofluorescence, and western blotting.

**Results:** The grafts of mice on HC and HF diets exhibited significantly fewer oil cysts and larger volume retention (0.18 ± 0.01, 0.21 ± 0.01, and 0.25 ± 0.01 ml, for ND, HC, and HF group, respectively, *p* < 0.05) on day 90. In comparison, grafts for the mice belonging to the HF groups exhibited higher expression of lipolysis-related genes, including adipose triglyceride lipase (ATGL), hormone-sensitive lipase (HSL), and carnitine palmitoyltransferase 1 (CPT1), on day 30. Furthermore, increased infiltration of macrophages (F4/80+) and the higher expression of angiogenesis genes were reported in the HF groups.

**Conclusion:** Altogether, the administration of short-term HF diet remarkably enhanced angiogenesis and improved the quality of fat grafts, which was characterized by fewer oil cysts and higher long-term volume retention. The possible mechanisms may be due to the increased macrophage infiltration, and the promoted angiogenesis in HF grafts.

## Introduction

In the past few years, fat grafting has gained immense attention worldwide. Particularly, it is routinely applied in diverse reconstruction surgery, including breast reconstruction after breast cancer mastectomy (Pearl et al., 2012), and contour deformities caused by facial muscle atrophy (Simonacci et al., 2017). The wide applicability of fat grafting is primarily attributed to the accessibility, biocompatibility, and versatility of autologous fat. However, the clinical application of fat grafting is somewhat compromised owing to the unpredictability of volume retention following transplantation. Although an array of surgical techniques, including cell-assisted lipotransfer (CAL) and brava-assisted fat grafting, have been explored to improve the retention of fat grafts (Yoshimura et al., 2008; Khouri et al., 2015), the challenges of long-term volume retention remain unaddressed so far. In addition, unavoidable oil cyst formation and fibrosis hinders the application of fat grafting (Dixit and Wagh, 2013; Abdallah et al., 2020). Therefore, there is an urgent need to devise new strategies for advancing the practical application of fat grafting.

Oil droplets have been previously shown to be closely associated with the occurrence of complications following fat grafting (Chen et al., 2021). Particularly, two main sources of oil droplets have been identified in fat grafts. The mechanical shear force utilized during liposuction induced fragmentation of adipocytes, which inevitably results in redundant oil droplets in the aspirated fat. In addition, the necrotic adipocytes in the hypoxic-ischemic center zone of grafts released oil droplets and initiated lipotoxicity related inflammation (Eto et al., 2012). After fat grafting, bursting of oil droplets in a short time induced the formation of lipid-laden macrophages (Webb and Moore, 2007), and the persistent activation of lipotoxicity associated inflammation, followed by fibrosis and cyst formation (Chen et al., 2021). Therefore, harnessing oil-scavenging capacity is pivotal for the high-quality retention of grafts. Physicians have so far focused on devising different harvesting and processing techniques to remove oil before grafting (Allen et al., 2013). Past studies have primarily focused on adipocyte viability and the number of stem cells (Smith et al., 2006; Kurita et al., 2008; Ferraro et al., 2011). Although the effects of these techniques appeared to be positive, the oil droplets from necrotic adipocytes following fat grafting remained a major challenge, which in turn hindered the clinical application of fat grafting.

Macrophages play an important role in the lipid metabolism (Kim et al., 2018). It has been reported that a HF diet promoted macrophage infiltration in adipose tissue of mice (Tang et al., 2014; Liu et al., 2020). Promoting early macrophage infiltration using used macrophage colony stimulating factor (M-CSF) improved fat graft survival by inducing angiogenesis (Cai et al., 2018). And the administration of HF diet for 8 weeks upregulated the expression of adipose triglyceride lipase (ATGL) and promoted triglyceride hydrolysis in the subcutaneous adipose tissues of mice (Gaidhu et al., 2010). Furthermore, the observations made in clinical practice revealed that the increasing consumption of food leads to the improved volume retention of fat grafts. Hence, it was hypothesized that, in the case of fat grafting, manipulation of macrophage infiltration by a short-term HF diet might reinforce the lipolysis and angiogenesis in grafts, and can assist in achieving fat grafts with decreased fibrosis and oil cyst formation. To prove this hypothesis, the present study established fat grafting models in mice. After fat grafting, the mice were respectively provided a normal diet, a high carbohydrate diet (HC, 80% carbohydrate), or a high-fat diet (HF, 60% fat) for 30 days. The long-term volume retention and quality of the grafts were assessed. In addition, the changes in lipolysis and angiogenesis in fat grafts were evaluated in detail.

## Materials and Methods

### Animals

All experiments were approved by the Nanfang Hospital Animal Ethics Committee Laboratory and conducted according to the guidelines of the National Health and Medical Research Council of China. All male C57/BL6 mice obtained from Southern Medical University, were housed in individual cages with a 12-h light/dark cycle and an available *ad libitum* diet and distilled water.

### Fat Grafting Model and Treatments

The mice of age 10–12 weeks were used in this study. The mice were first anesthetized with 5% isoflurane and maintained using a 1.5% isoflurane/medical air mixture. Inguinal fat pads from the sacrificed donor mice were harvested and soaked in sterile PBS for 2 times, subsequently put on sterile gauze to remove adherent fluid, and finally placed into a sterile container. The harvested inguinal fat pads were repeatedly minced into tiny pieces with scissors to simulate autologous fat transplantation in humans. These fat particles were then individually grafted into the dorsal region of recipient mice (0.3 ml fat/per mouse) using 1 ml syringes with 21G needles. The fat-grafted mice (*n* = 54) were separately raised on a normal diet (ND, 12% Kcal from fat, 67% Kcal from carbohydrate; LabDiet Cat no. SFS9112, Xietong, China), a HC diet (9.3% Kcal from fat, 80.1% Kcal from carbohydrate; LabDiet Cat no. D191122, Dyets Inc., Bethlehem, PA), and a HF diet (HF, 60% Kcal from fat, 20% Kcal from carbohydrate; LabDiet Cat no. D112252, Dyets Inc., Bethlehem, PA) for 1 month, and then all the fat-grafted mice were fed with a normal diet until execution. The mice were sacrificed after 1, 2, and 3 months (*n* = 6 per time point per group), and the volumes of the harvested grafts were measured by using the Archimedes water-drainage method.

### Histological Analyses

The fat tissue sections were sectioned at 4 μm and stained with hematoxylin and eosin (HE). Immunofluorescence assay was performed with the following primary antibodies: rat anti-mouse perilipin (1:100; Abcam) and rabbit anti-mouse CD31 (1:100; Abcam). The sections images were captured using a light microscope (BX51; Olympus, Tokyo, Japan).

### Real-Time Quantitative Polymerase Chain Reaction Analysis

Fat tissue samples from the grafts were homogenized with a tissue grinder in Trizol (Invitrogen, United States). After centrifugation at 12,000 rpm for 5 min, the upper oil layer was removed, and total RNA was isolated with chloroform, isopropanol, and ethanol according to the manufacturer’s protocol. Total RNA was then used to synthesize cDNA with the RevertAid First Strand cDNA Synthesis Kit (Thermo Scientific, Waltham, United States). qPCR was conducted using the LightCycler 480 Fluorescence Quantitative PCR instrument (Roche, Indianapolis, IN, United States) and the Hieff qPCR SYBR Green Master Mix (Yesen, Shanghai, China). The gene expression was calculated as Ct, while the relative expression levels were calculated with the 2^−ΔΔCt^ method. The primers used in the study are as follows: (Table 1)

**TABLE 1:** The primers of genes.

Primers	Primer sequence (5′-3′)
*CD36*	Forward: AAC​TGG​TGG​ATG​GTT​TCC​T
	Reverse: GTG​GCC​CGG​TTC​TAC​TAA​T
*FABP4*	Forward: AATCACCGCAGACGACA
	Reverse: ACATTCCACCACCAGCTT
*ATGL*	Forward: GTCCTTCACCATCCGCTT
	Reverse: CTCTTGGCCCTCATCACC
*HSL*	Forward: GCATGGATTTACGCACGA
	Reverse: CCCGAACACCTGCAAAG
*CPT1*	Forward: CCCAGTCAGATTCCAACC
	Reverse: TCA​CCA​AAA​TGA​CCT​AGC​C
*VEGFα*	Forward: GTG​GAA​ATC​AGC​AGA​CGA​A
	Reverse: CCCCAAAAGCAGGTCAG
*PDGFα*	Forward: CCA​TTC​GCA​GGA​AGA​GAA​G
	Reverse: CAGGAAGTTGGCCGATG

### RNA-Seq Analysis of Fat Tissue Before and After Grafting

To investigate the influence of fat grafting on lipolysis, RNA was extracted from the fat tissues before grafting and 1 week after grafting for RNA-seq analysis (3 biological replicates per group). RNA-seq experiments was performed by Novogene (Beijing, China). Total RNA was drawn from the tissues with TRIzol reagent (Absin, Shanghai, China). Transcriptome sequencing were performed by using the Illumina HiSeq X Ten (Novogene Bioinformatics Technology Co., Ltd., Beijing, China). Mapping of 150-bp paired-end reads to the genes was undertaken, and the fragments per kilobase of transcript per million fragments mapped (FPKM) were analyzed. The significance *p* values (padj) were adjusted by the Benjamini and Hochberg method (https://www.ncbi.nlm.nih.gov/geo/info/linking.html). The heat map was prepared with gplots and heatmap.2.

### Western Blotting

Total protein was extracted by lysis with the RIPA buffer (RIPA, Beyotime, China) containing phenylmethanesulfonyl fluoride (PMSF, Beyotime). Primary rabbit antibodies against ATGL, HSL, and p-HSL were sourced from CST Company (Boston, United States). After incubation with goat anti-rabbit IgG (H + L)-HRP secondary antibodies, each protein band were detected using an enhanced chemiluminescence solution. GAPDH was used as an internal control.

### Statistical Analyses

Quantitative data were analyzed by *t*-test or a one-way or two-way analysis of variance using the GraphPad Prism software (version 8.0) (GraphPad Software, Inc., La Jolla, CA, United States). Data are presented as mean ± standard deviation. *p* < 0.05 was considered to indicate statistical significance, while *p* < 0.01 indicated highly statistical significance.

## Results

### Lipid Metabolism-Related Genes in Fat Grafts Were Down-Regulated One-Week Post-grafting

**FIGURE 1:**
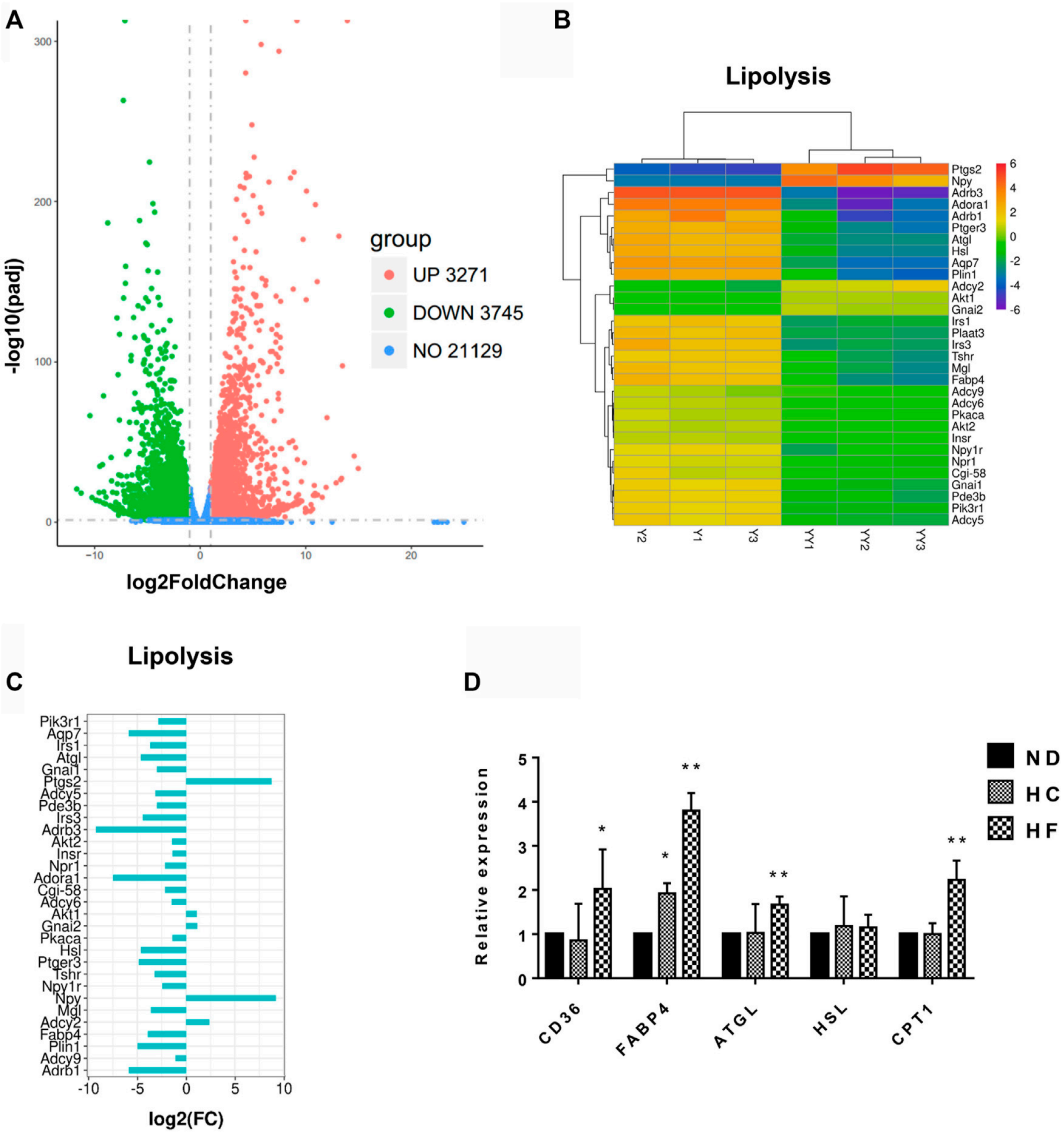
Transcriptome data analysis of fat tissues before and 1 week after grafting, and the genes changes in the inguinal fat of mice fed with different diets. **(A)** Volcano plots of all expressed genes in samples before (Y1, Y2, Y3) and 1 week after fat grafting (YY1, YY2, YY3). **(B)** Heatmap of differentially expressed lipolytic genes. **(C)** Histogram of Log2 Fold change (FC) of the down-regulated lipolytic genes in tissues before and 1 week after grafting. **(D)** Lipolysis gene expression was examined by qPCR. **p < 0.01 and *p < 0.05. *n* = 6 mice.

To analyze the changes in gene expression after fat grafting, adipose tissue before and after 1 week of grafting were subjected to whole transcriptome sequencing, wherein 28,145 genes were detected. Among these, 3,271 genes were found to be upregulated, while 3,745 genes were downregulated (*p*_*ad*j_ < 0.05 and log2(FC) > 1) in grafts after 1 week of grafting, when compared with that in the natural fat tissues (Figures 1A–C), the genes related to lipid metabolism in adipose tissue, such as ATGL, hormone-sensitive lipase (HSL), monoacylglycerol lipase (MGL), comparative gene identification**-**58 (CGI58), and fatty acid-binding protein 4 (FABP4), were among the 3,745 genes that were significantly downregulated. At the same time, expression levels of genes related to lipogenesis and lipolysis were determined by qPCR. When compared with the control group, inguinal fat from HF diet mice showed an elevated expression of lipolysis genes, including ATGL (1.6 ± 0.2 folds), HSL (1.4 ± 0.1 folds), CD36 (2.7 ± 0.5 folds), FABP4 (3.8 ± 0.4 folds), and carnitine palmitoyltransferase 1 (CPT1, 2.2 ± 0.4 folds) (Figure 1D).

### A Short-Term HF Diet After Fat Grafting Improved Volume Retention of Fat Grafts

**FIGURE 2:**
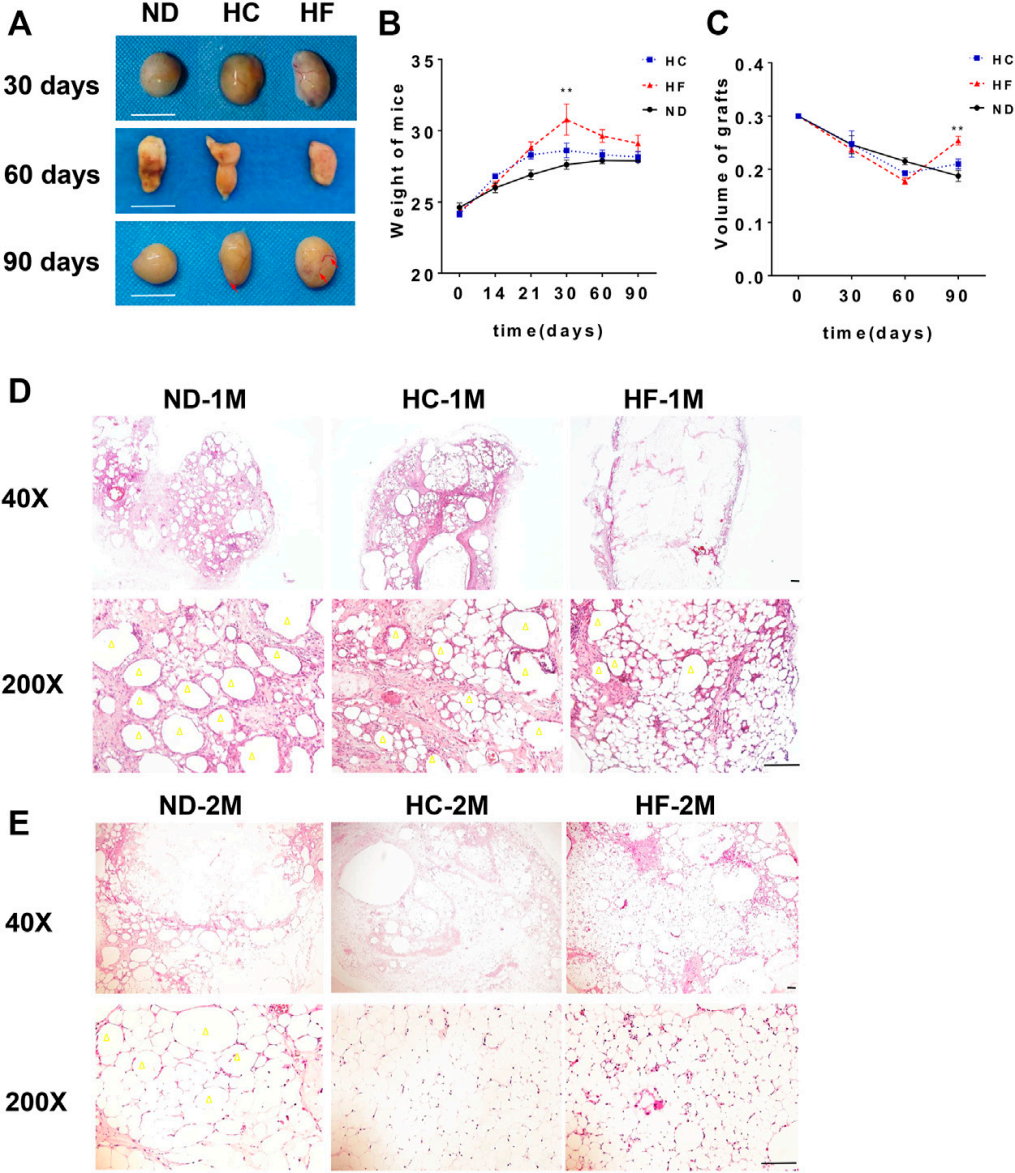
The changes of fat grafts in the 3 study groups **(A)** Representative gross appearance of the harvested fat grafts on days 30, 60, and 90. **(B)** The weight of mice on different diets and **(C)** the quantification of the graft volume. Scale bars = 10 mm Representative HE staining of the fat grafts obtained on day 30 **(D)**, and 60 **(E)**. Scale bars = 100 μm. Grafts on day 30 are marked as ND-1M, HC-1M, and HF-1M. Grafts on day 60 are marked as ND-2M, HC-2M, and HF-2M. Yellow arrow: oil cyst. **p < 0.01 and *p < 0.05. *n* = 6 mice.

To evaluate the affect of sequential diet administration on lipid metabolism in fat grafts, After fat grafting, the mice were divided into three groups and fed with ND, HC, or HF diets for 1 month, followed by ND for the remaining time. The grafts samples were collected on days 30, 60, and 90. when compared with the ND group, intuitively, a better vascularized texture (red arrow) were observed in the fat grafts of HC and HF groups (Figure 2A). The weight of C57BL/6 mice reached the peak when administered with HC diet or HF diet for 1 month. After ND diet feeding for the next 2 months, a similar decline was observed in mice of three groups (Figure 2B). The volume retention of fat grafts in the HC (0.21 ± 0.01 ml, *p* < 0.05) and HF (0.25 ± 0.01 ml, *p* < 0.01) group was higher than that of the ND group (0.18 ± 0.01 ml) in 90 days (Figure 2C).

### A Short-Term High Fat Diet After Fat Grafting Reduced Oil Cyst Formation and Fibrosis in Fat Grafts

**FIGURE 3:**
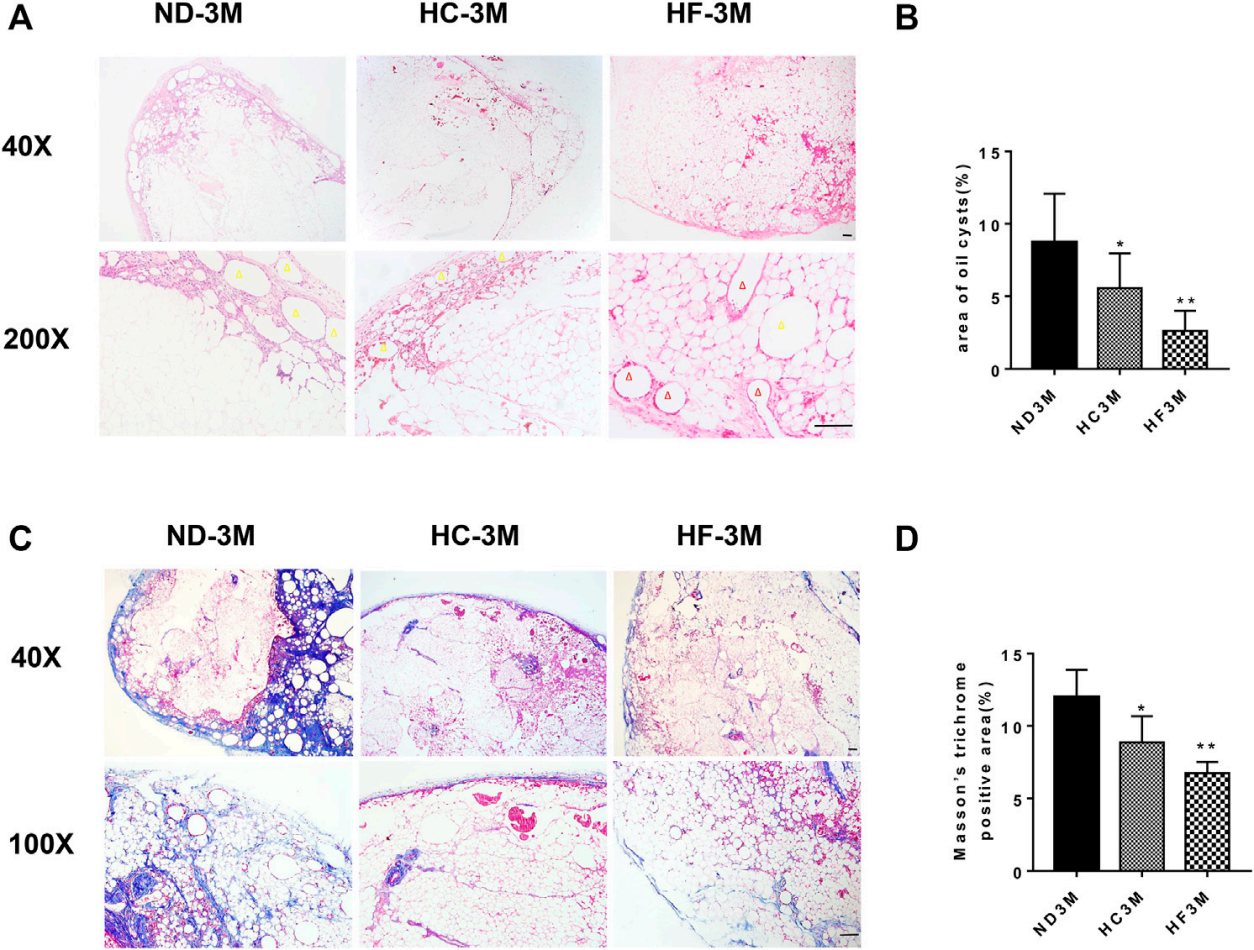
Histological changes in fat grafts. Representative HE staining of grafts obtained on day 90 **(A)**, and the quantification of the area of oil cysts **(B)**. Representative Masson trichrome staining **(C)** and the quantification of Masson trichrome staining **(D)** of 90-days fat grafts from mice on different diets. Grafts on day 90 are marked as ND-3M, HC-3M, and HF-3M. Yellow arrow: oil cyst. Red arrow: microvessel. Scale bars = 100 μm **p < 0.01 and *p < 0.05.

H&E staining was performed to analyze the effect of diets on the structure change in fat grafts. The number of oil cysts (yellow arrow) observed in the grafts of mice belonging to HC and HF groups were less than that in the ND group at day 30. The grafts of HF group exhibited the least number of oil cysts (Figure 2D), which were hardly visible at days 60 (Figure 2E). Furthermore, a larger number of small adipocytes were visualized in grafts of the HF group at days 30 and 60. On day 90, adipocytes in grafts of these three groups were similar in size (Figure 3A). Importantly, oil cysts were still visible in the 90-days ND grafts. However, oil cysts were nearly invisible in HF grafts (Figure 3B). Instead, a distinct vascular structure (red arrow) was observed. Moreover, the results for Masson staining indicated that the fibrosis was less in grafts of the HF (*p* < 0.01) and HC (*p* < 0.01) group grafts (Figures 3C,D).

### A Short-Term High-Fat Diet After Grafting Promoted the Lipolytic Gene Expression and Increased the Infiltration of Macrophages in Fat Grafts

**FIGURE 4:**
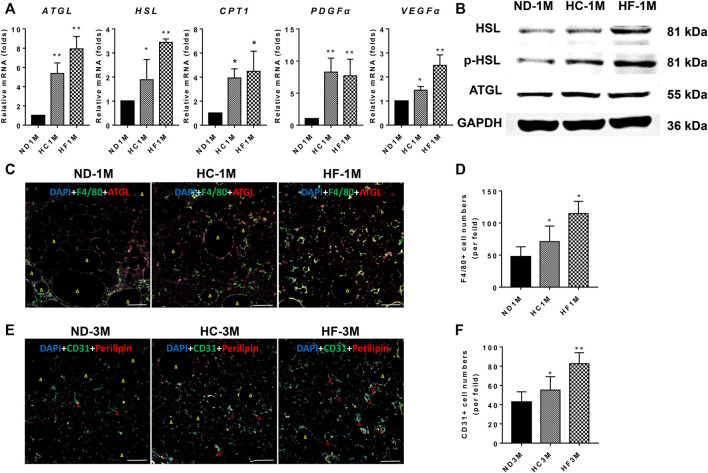
Expression changes of genes related to lipolysis and angiogenesis. **(A)** The mRNA expression levels of ATGL, HSL, CPT1, PDGFα, and VEGFα. **(B)** Western blotting analysis of ATGL, HSL, and p-HSL. **(C,D)** Representative immunofluorescence staining of ATGL (red) and F4/80 (green) in 30-days grafts, and the number of macrophages (F4/80 + cells) quantified. **(E,F)** Representative immunofluorescence staining of Perilipin (red) and CD31 (green) in 90-days grafts, and the number of CD31^+^ cells quantified. Grafts on day 30 are marked as ND-1M, HC-1M, and HF-1M. Yellow arrow: oil cyst. Red arrow: microvessel. Scale bars = 100 μm **p < 0.01 and *p < 0.05.

The relative expression of ATGL and HSL were determined by qPCR. As shown in Figure 4A, a 1**-**month HF diet significantly (*p* < 0.01) increased the mRNA expression of ATGL (7.0 fold), HSL (3.4 fold), and CPT1 (4.5 fold). The expression of ATGL (5.4 fold), HSL (2.2 fold) and CPT1 (3.9 fold) was also enhanced in 1-month HC grafts as compared with those in ND grafts. Meanwhile, grafts obtained from HC and HF group on day 30 displayed increased levels of lipolytic proteins, including ATGL, HSL, and p**-**HSL (Figure 4B). Increased infiltration of macrophages (F4/80+) was also observed in HF grafts (*p* < 0.05), which was accompanied by enhanced ATGL expression (Figures 4C,D).

### A Short-Term HF Diet After Fat Grafting Promoted Angiogenesis in Fat Grafts

The results for qPCR analysis demonstrated that angiogenesis-related genes, including VEGFα and PDGFα, were upregulated in grafts of HC (*p* < 0.05) and HF groups (*p* < 0.05) on day 30 (Figure 4A). Further, the study assessed the angiogenesis marker (CD31, green) and lipid droplet marker (perilipin, red) in day-90 grafts (Xu et al., 2009). Immunostaining of perilipin demonstrated that grafts in the HF group exhibited fewer oil cysts (yellow arrow) than that of the ND group, while HC grafts exhibited more oil cysts than the HF grafts on day 90 (Figures 4E,F). Moreover, HC (*p* < 0.05) and HF grafts (*p* < 0.01) showed higher CD31 positive cells as compared with the ND grafts. HF grafts exhibited the highest microvessel density (red arrow).

## Discussion

The recent decades, accumulated evidence indicated that the adipose tissues act as both an energy reservoir and an endocrine mediation organ (Luo and Liu, 2016). Following sprouting from mesenchymal stem cells, adipocytes deposit lipid droplets under the influence of nutrition and hormones, accompanied by the lipogenic and lipolytic loops. Large lipid droplets formed in mature adipocytes. However, mechanical damage to brittle adipocytes during fat transplantation and the lack of adequate blood and oxygen supply to the grafts during the early stage after fat grafting induced adipocyte necrosis and another wave of oil release (Eto et al., 2012; Sunaga et al., 2013). Next, massive oil droplets get instantaneously accumulated in the fat grafts, causing oil aggregation beyond the scrapable capacity and the formation of oil cysts. The effect incurred by rapidly increasing oil droplets on fat grafts remain unclear. Thus, focus are gained onto lipid metabolism in fat grafting in this study.

The lipolysis procedure involves successive cascades. Particularly, ATGL and HSL act as rate**-**limiting enzymes for lipolysis and regulate the catabolism of triglycerides in oil droplets (Haemmerle et al., 2006; Haemmerle et al., 2002). These enzymes are responsible for the hydrolysis of about 95% triglycerides (Schweiger et al., 2006). Meanwhile, CGI**-**58 along with ATGL has been shown to modestly enhance the lipolysis activity (Lass et al., 2006). Each hydrolysis step yielded one fatty acid. Thus, the attenuated expression of these lipolysis genes would induce the accumulation of triglycerides (Radner et al., 2010; Liu et al., 2017). The results for high**-**throughput sequencing in the present study demonstrated that lipolysis genes, including ATGL, CGI-58, HSL, and MGL, were remarkably downregulated after grafting. Therefore, the formation of oil cysts at a later stage may be directly related to the burst accumulation of oil droplets during the early stage and the attenuated lipolytic capacity. Meanwhile, the oleaginous swamp might evoke enduring inflammation and fibrosis, which drag down the long-term outcomes of fat grafts (Chen et al., 2021). Thus, rejuvenation of the lipolytic activity appears to be a feasible avenue to improve fat grafting, particularly in terms of inhibiting vesicle formation. Interestingly, Gaidhu et al. reported that the expression of ATGL in adipose tissues can be unregulated by HF diet (Gaidhu et al., 2010). In the present study, HF diets could promote the expression of ATGL and HSL in the inguinal fat tissue. This was accompanied by the elevation expression of CD36, FABP4, and CPT1 (Figure 1D), which regulate the uptake of fatty acids (Wolf Greenstein et al., 2017) and the mitochondrial β-oxidation activity (Labrie et al., 2015).

Furthermore, we found that the grafts of the HC and HF groups exhibited lower volume as compared with those of the ND group on day 60. However, the volume of grafts in the HF group on day 90 was larger than that in the ND group. In parallel, HC and HF groups exhibited lesser oil cysts and more smaller preadipocytes on day 30 and 60. On day 90, the previously shrunken adipocytes swelled to a mature size. Weight gain was reported in mice belonging to HC and HF groups by day 30, but after being fed with an ND diet for the next 2 months, no significant differences were recorded in bodyweight among these three groups (Figure 2B). Cumulatively, we presumed that a HF diet increased lipid metabolism, promoted the absorption and scavenging of lipid droplets, and reduced the size of adipocytes. Increased expression of ATGL and HSL was observed in HC and HF grafts on day 30. The HC and HF diets also promoted the expression of *CPT1*, a key factor that regulats fatty acid metabolism (Chakravarthy et al., 2005). The higher expression of lipolysis genes indicated that the oil droplets in the grafts would be catabolized more quickly. This provided a possible explanation for the existence of less oil cysts and small adipocytes during the early stage of fat grafting in HF grafts.

Macrophages are actively involved in lipid engulfment and degradation (Flaherty et al., 2019). Free fatty acids and glycerol are the hydrolyzed products of triglycerides through lipolysis. In damaged fat tissues, the free fatty acids are shifted to mitochondria, and participate in fatty acid oxidation. Increased angiogenesis and adequate blood supply are critical to the β-oxidation process. With sufficient tissue blood supply and oxygenation, released fatty acids are effectively scavenged (Ahmadian et al., 2007; Onal et al., 2017). However, in fat grafts, early ischemia and hypoxia is inevitable (Kato et al., 2014). The slow vascular regeneration process suppressed the lipolysis and β-oxidation of fatty acids, and resulted in the formation of oil cysts. Conversely, decreased oil cysts were observed in fat grafts assisted by stromal vascular fraction cells or vascular endothelial growth factor (VEGF) (Lu et al., 2009; He et al., 2019). Simultaneously, macrophages act as major immune cells in promoting angiogenesis and vascular regeneration (Godwin et al., 2013; Godwin et al., 2017). As a repetitious propellant for blood supply, vascular regeneration in the early stage seems pivotal for the final volume retention of fat grafts (Mashiko and Yoshimura, 2015). Stimulating macrophage infiltration in fat grafts using macrophage colony stimulating factor was able to improve angiogenesis and fat graft survival (Cai et al., 2018). And increased VEGF expression promoted angiogenesis and decreased hypoxia in adipose tissue. In addition, platelet derived growth factor (PDGF) secreted by macrophages prevented apoptosis of preadipocytes by binding to the PDGF receptor on the surface of preadipocytes (Molgat et al., 2009). Those positive changes can offset the imbalance of lipid metabolism (Sung et al., 2013).

It was previously reported that HF diets promoted macrophage infiltration in adipose tissue (Tang et al., 2014; Liu et al., 2020). In the present study, a short-term HF diet promoted the infiltration of macrophages, and increased the expression of VEGFα and PDGFα in fat grafts. In addition, enhanced ATGL expression was observed around macrophages in Figure 4C. The increased average microvascular density in HF grafts was showed by CD31 staining. Therefore, we were prone to speculate that the macrophages induced by HF diets promoted angiogenesis and vascular regeneration by paracrine excretion of VEGFα and PDGFα. And the increased macrophages activated lipolysis process. The effective vascular regeneration and oxygen supply provided favorable conditions for the enhanced fatty acid metabolism of macrophages. Eventually, the timely clearance of oil droplets mitigated lipotoxicity and reduced fibrosis in grafts. This may provide a possible preliminary explanation for the improvement effects of short-term HF diets on fat grafts. More in-depth studies were needed to explore the specific mechanism between macrophages and lipolysis regulation in fat grafts.

## Conclusion

The present results demonstrated that a short-term HF diet stimulated macrophage infiltration, upregulated the lipolytic activity and promoted angiogenesis in the grafts. Moreover, the rapid clearance of oil droplets and timely revascularization further promoted each other, increased the survival rate of transplanted adipocytes, and improved the long-term volume retention of grafts. However, we acknowledge that this study has some limitations as is unavoidable in an animal study, and hence further clinical research is warranted to validate our results. Nonetheless, we demonstrated an intriguing nonsurgical treatment approach through lipolysis regulation that presents a novel direction for improving the outcomes of fat grafting.

## Data Availability

The datasets presented in this study can be found in online repositories. The names of the repository/repositories and accession number(s) can be found in the article/Supplementary Material.
