# Supplementary figures and images for: A Short-Term High-Fat Diet Improved the Survival of Fat Grafts in Mice by Promoting Macrophage Infiltration and Angiogenesis (part 1 of 2)
Source: Front Cell Dev Biol. 2022 Mar 17;10:856839. doi: 10.3389/fcell.2022.856839 (PMC8968084; doi:10.3389/fcell.2022.856839)

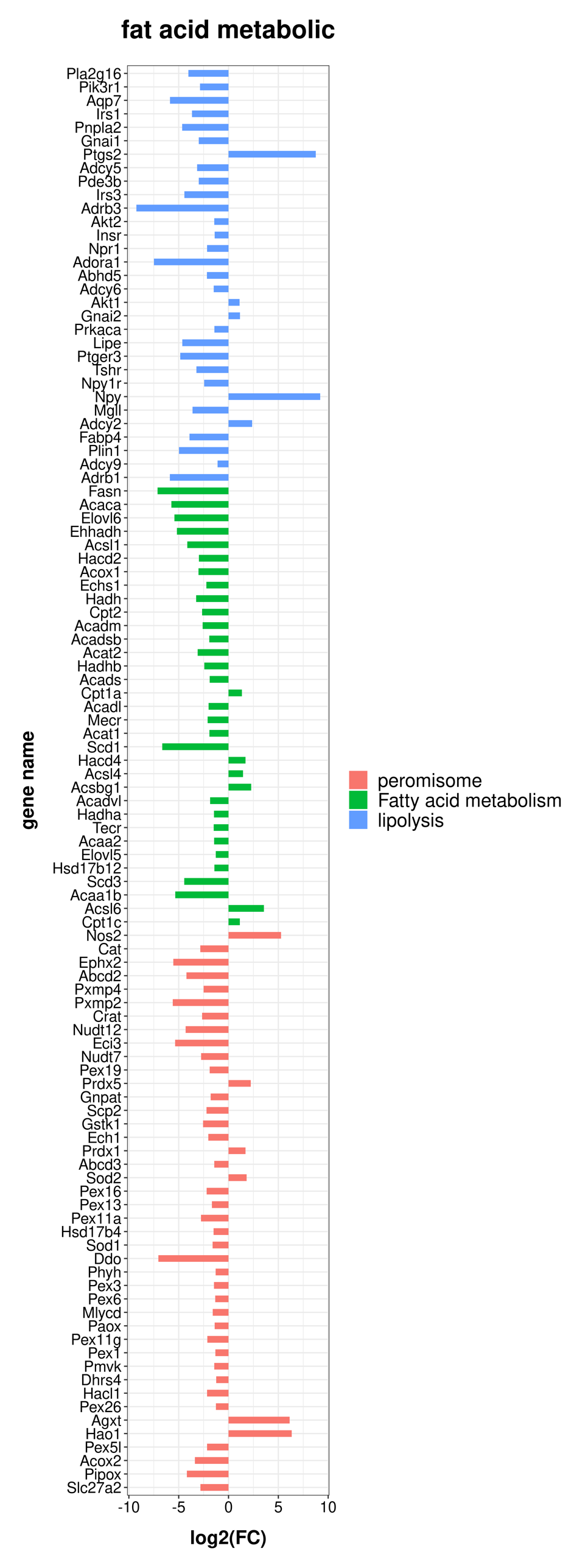

Supplement: Supplementary file 1 [file DataSheet4.ZIP › data for figure 1/fat acid metabolic FC .png]

# Lipolysis

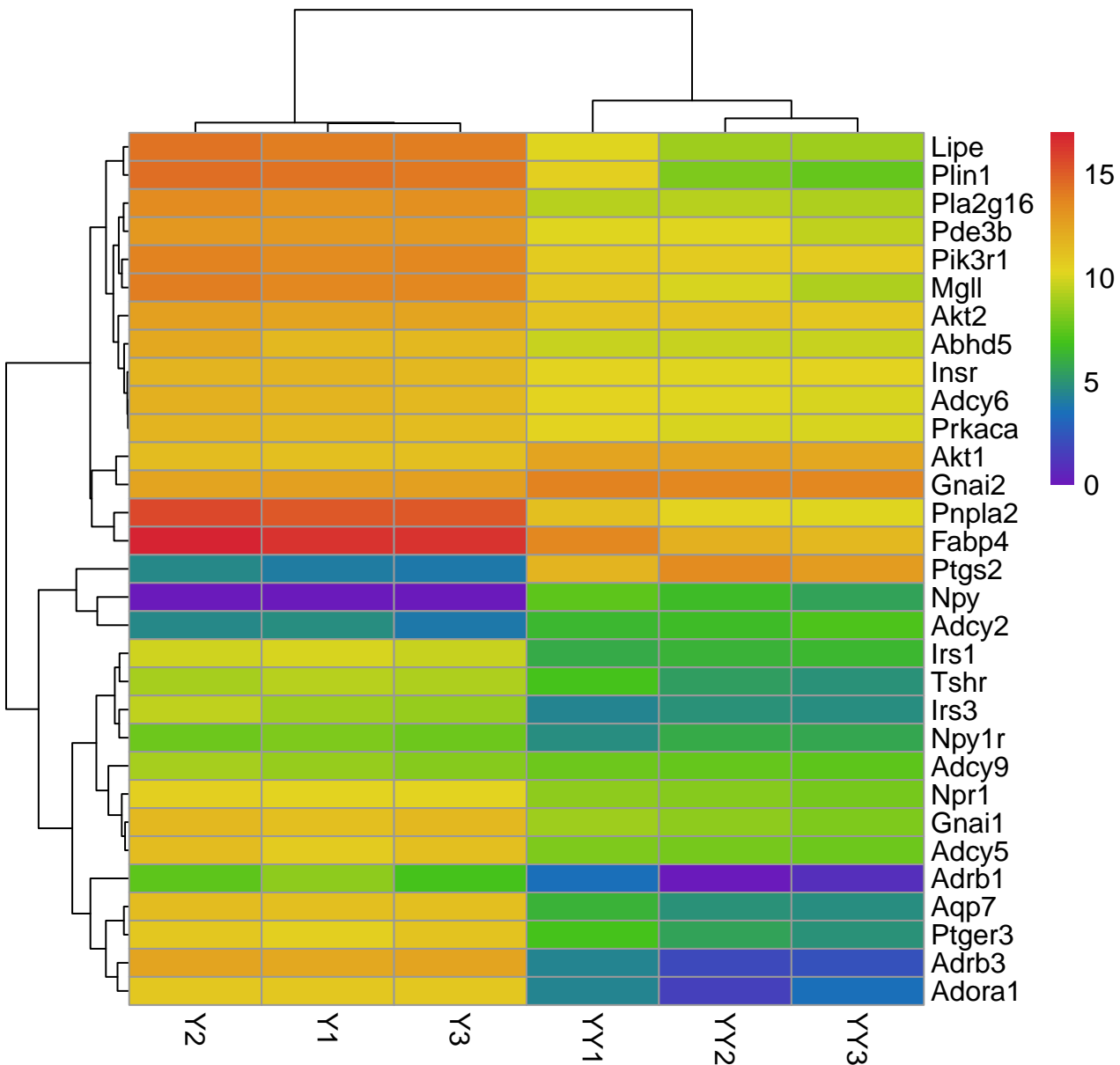

Supplement: Supplementary file 1 [file DataSheet4.ZIP › data for figure 1/Heatmap Lipolysis.pdf]

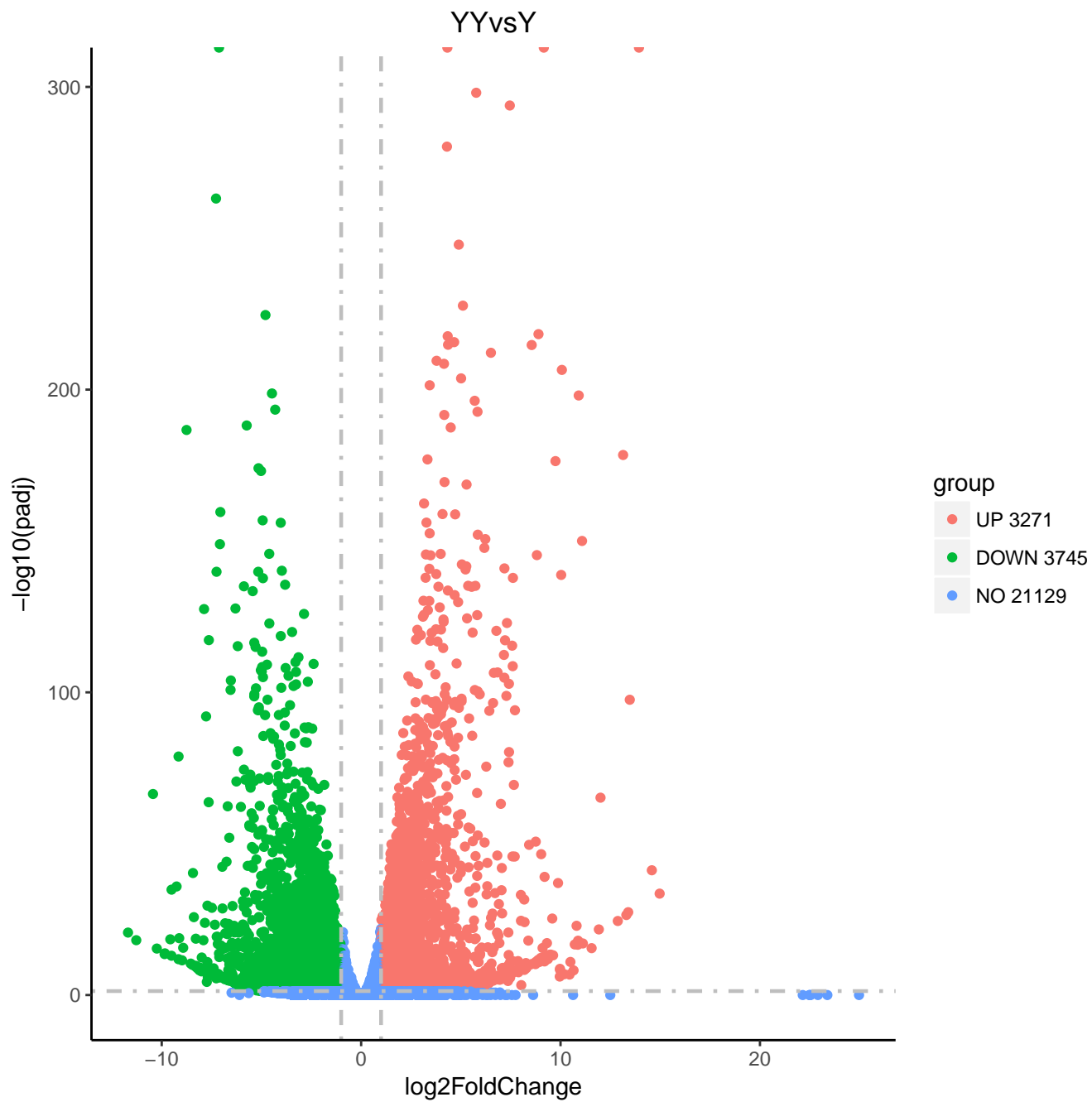

Supplement: Supplementary file 1 [file DataSheet4.ZIP › data for figure 1/YYvsY_Volcanoplot.pdf]

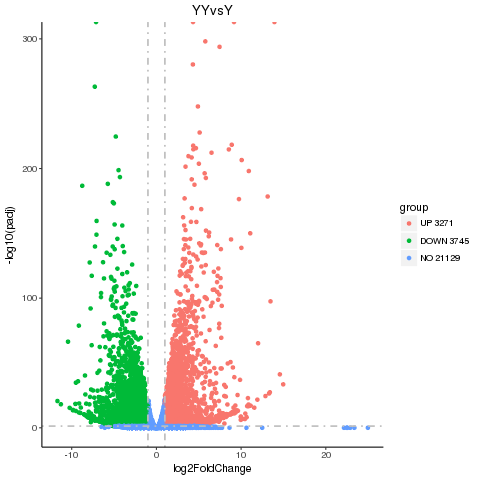

Supplement: Supplementary file 1 [file DataSheet4.ZIP › data for figure 1/YYvsY_Volcanoplot.png]

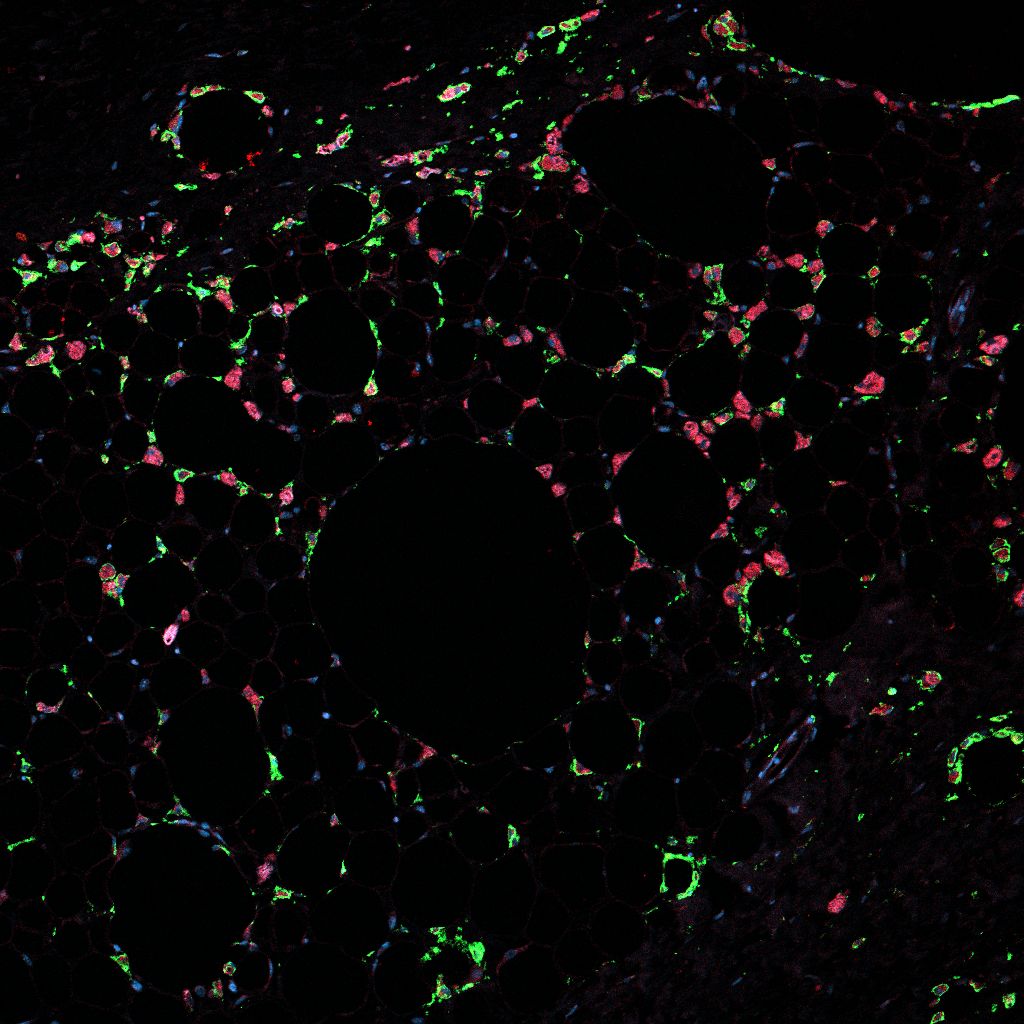

Supplement: Supplementary file 2 [file DataSheet1.ZIP › data for figure 4/IF ATGL F4-80 in ND-HC-HF 1M grafts/HC-1M-F480-ATGL-01.jpg]

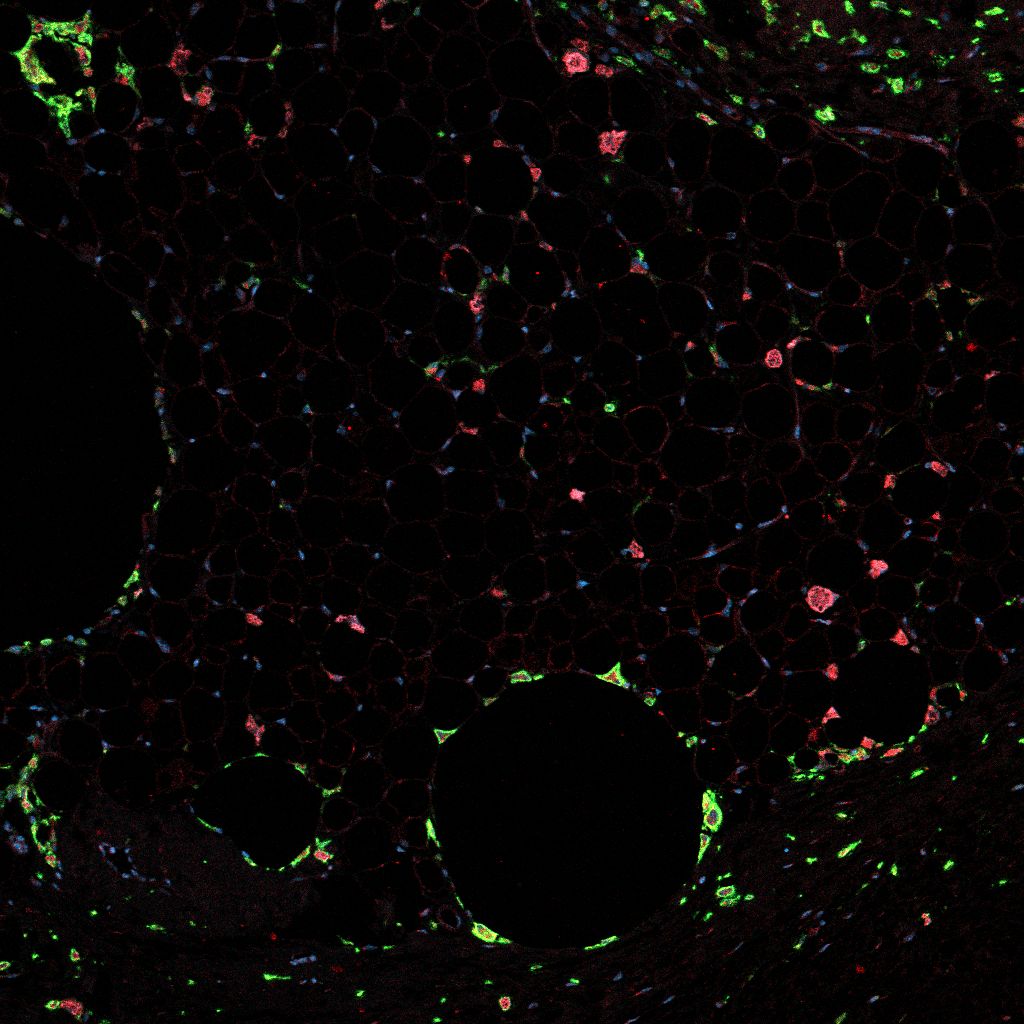

Supplement: Supplementary file 2 [file DataSheet1.ZIP › data for figure 4/IF ATGL F4-80 in ND-HC-HF 1M grafts/HC-1M-F480-ATGL-08.jpg]

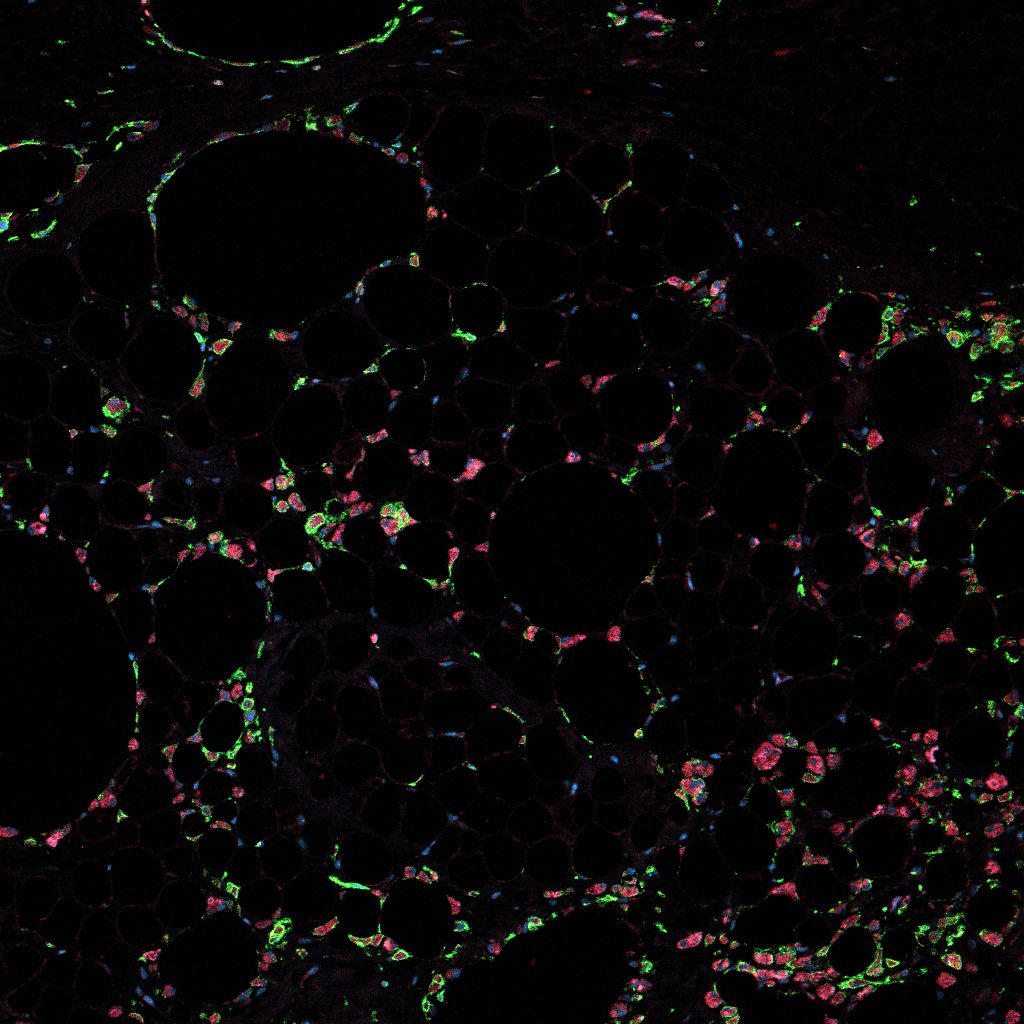

Supplement: Supplementary file 2 [file DataSheet1.ZIP › data for figure 4/IF ATGL F4-80 in ND-HC-HF 1M grafts/HC-1M-F480-ATGL-09.jpg]

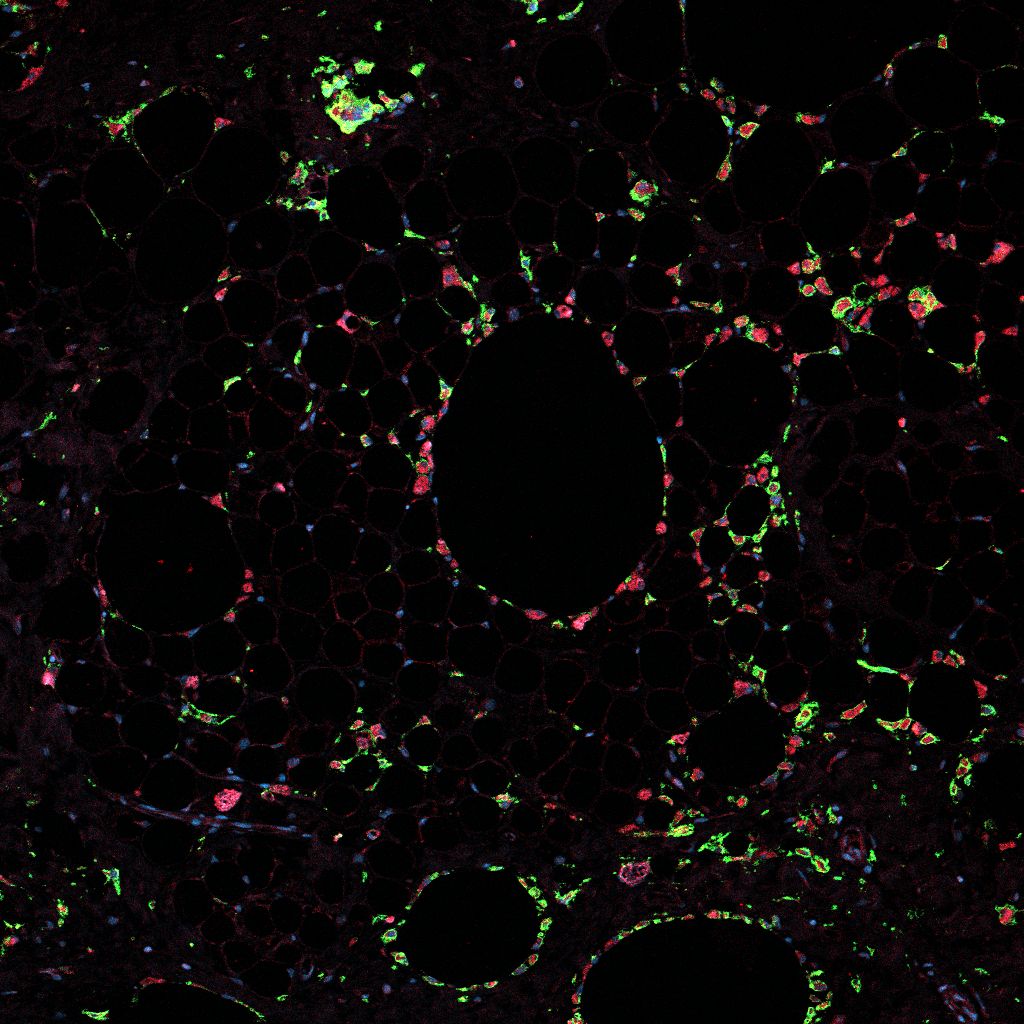

Supplement: Supplementary file 2 [file DataSheet1.ZIP › data for figure 4/IF ATGL F4-80 in ND-HC-HF 1M grafts/HC-1M-F480-ATGL-10 used in figure.jpg]

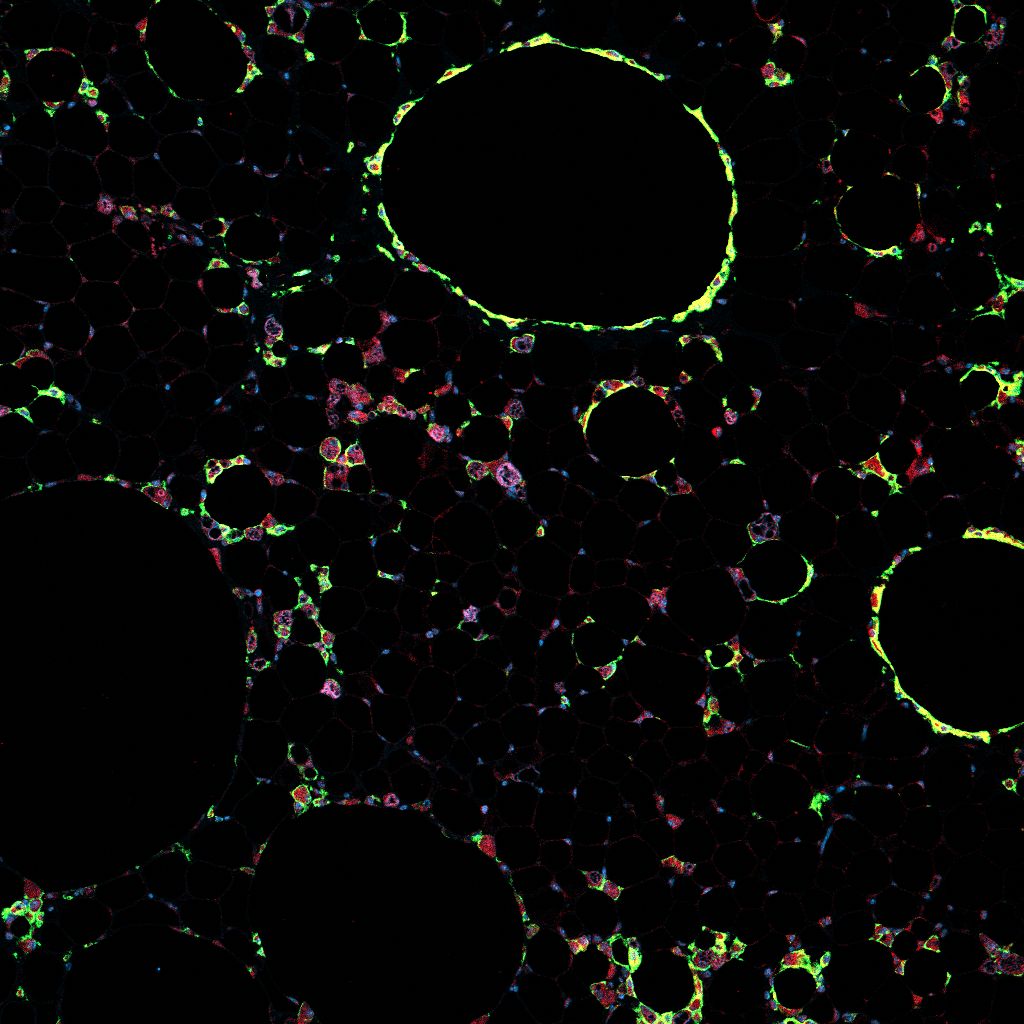

Supplement: Supplementary file 2 [file DataSheet1.ZIP › data for figure 4/IF ATGL F4-80 in ND-HC-HF 1M grafts/Hf-1M-F480-ATGL-02.jpg]

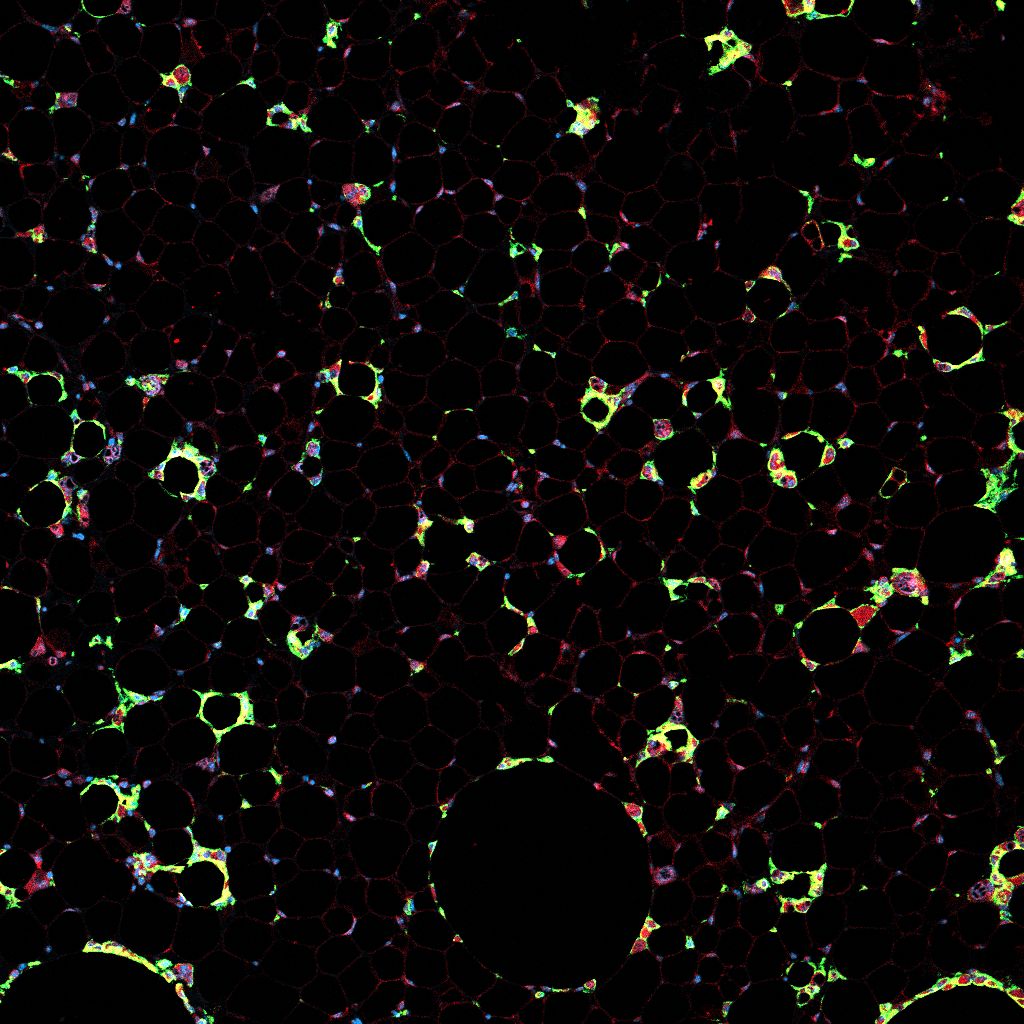

Supplement: Supplementary file 2 [file DataSheet1.ZIP › data for figure 4/IF ATGL F4-80 in ND-HC-HF 1M grafts/Hf-1M-F480-ATGL-03 used in figure.jpg]

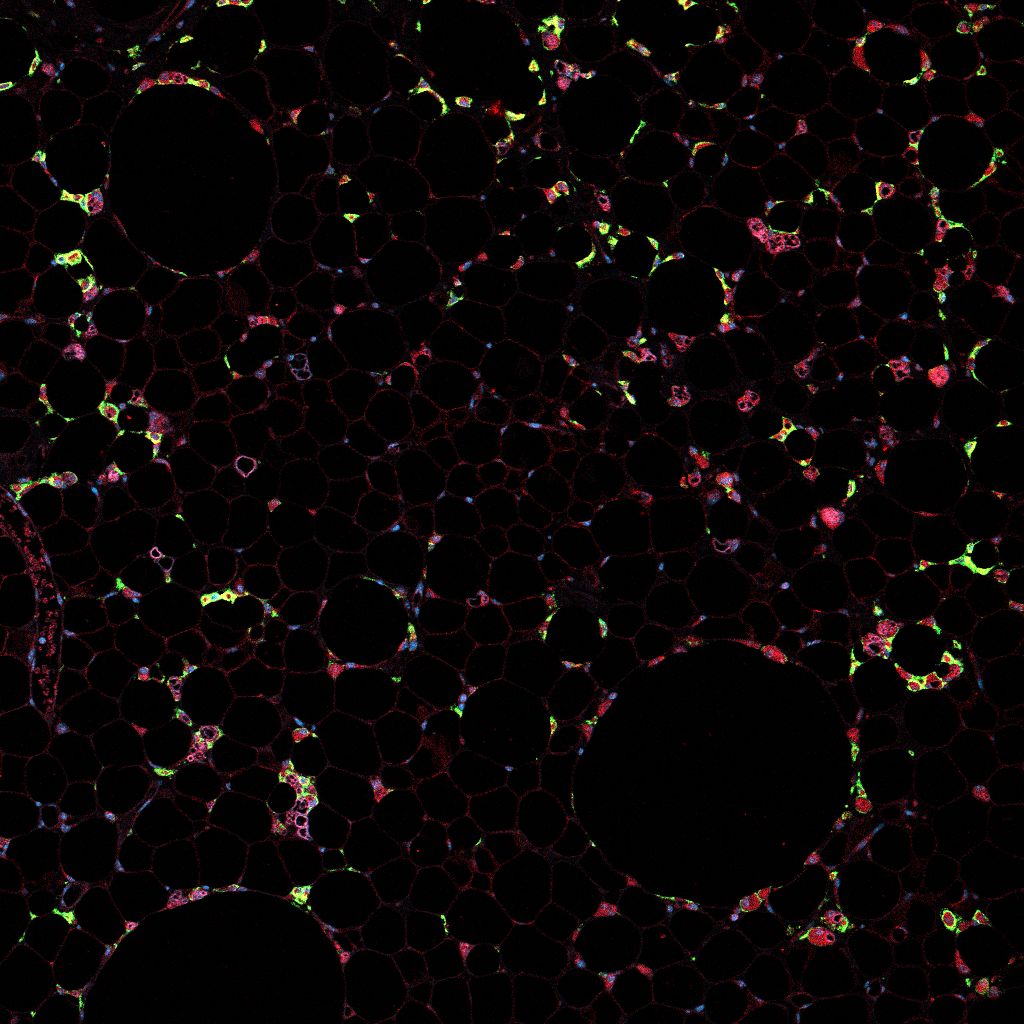

Supplement: Supplementary file 2 [file DataSheet1.ZIP › data for figure 4/IF ATGL F4-80 in ND-HC-HF 1M grafts/Hf-1M-F480-ATGL-05.jpg]

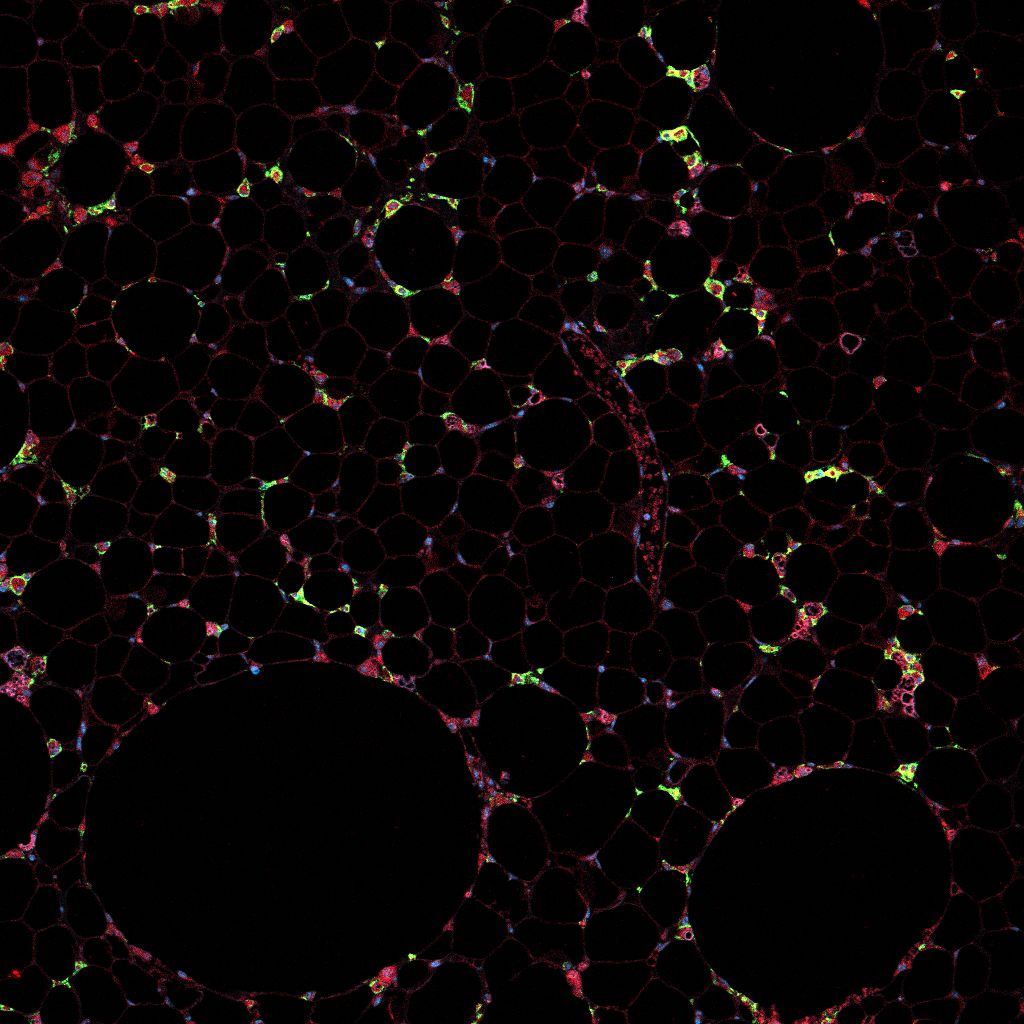

Supplement: Supplementary file 2 [file DataSheet1.ZIP › data for figure 4/IF ATGL F4-80 in ND-HC-HF 1M grafts/Hf-1M-F480-ATGL-06.jpg]

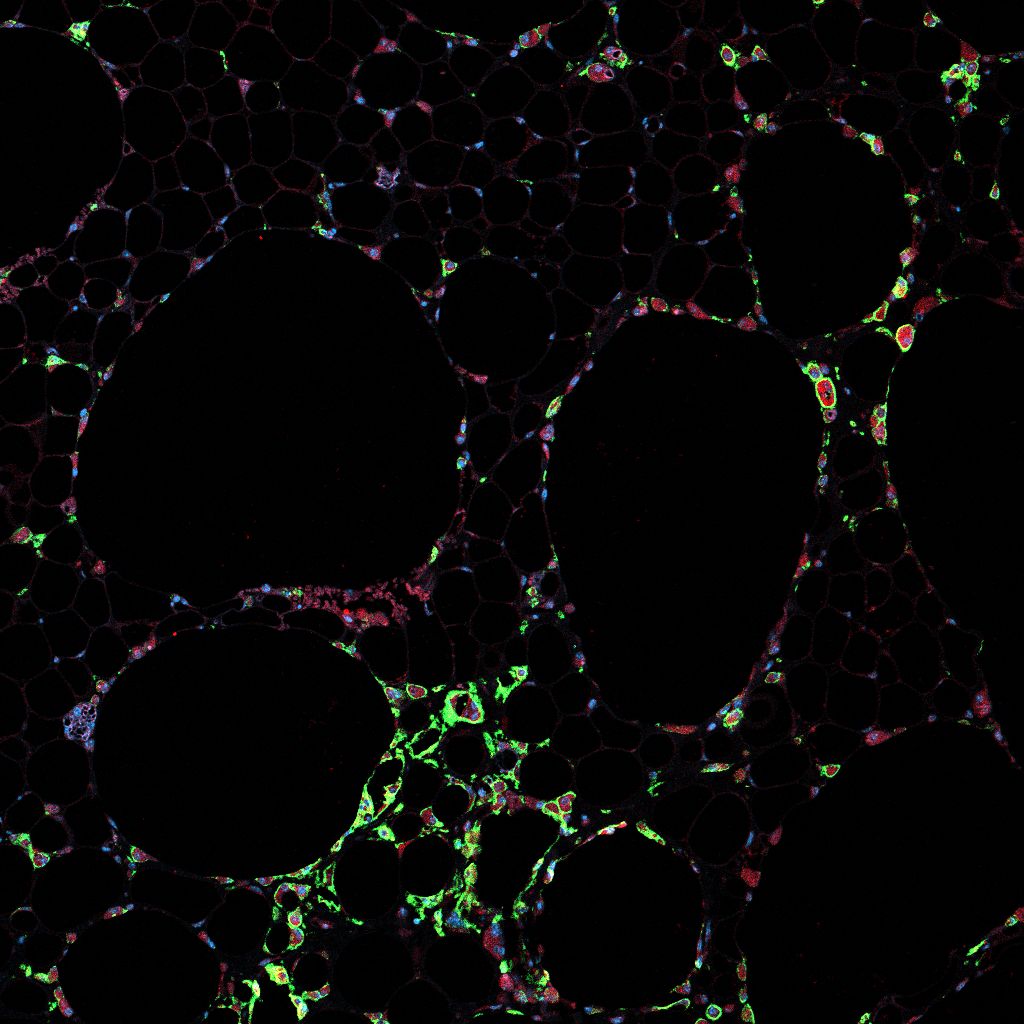

Supplement: Supplementary file 2 [file DataSheet1.ZIP › data for figure 4/IF ATGL F4-80 in ND-HC-HF 1M grafts/Hf-1M-F480-ATGL-08.jpg]

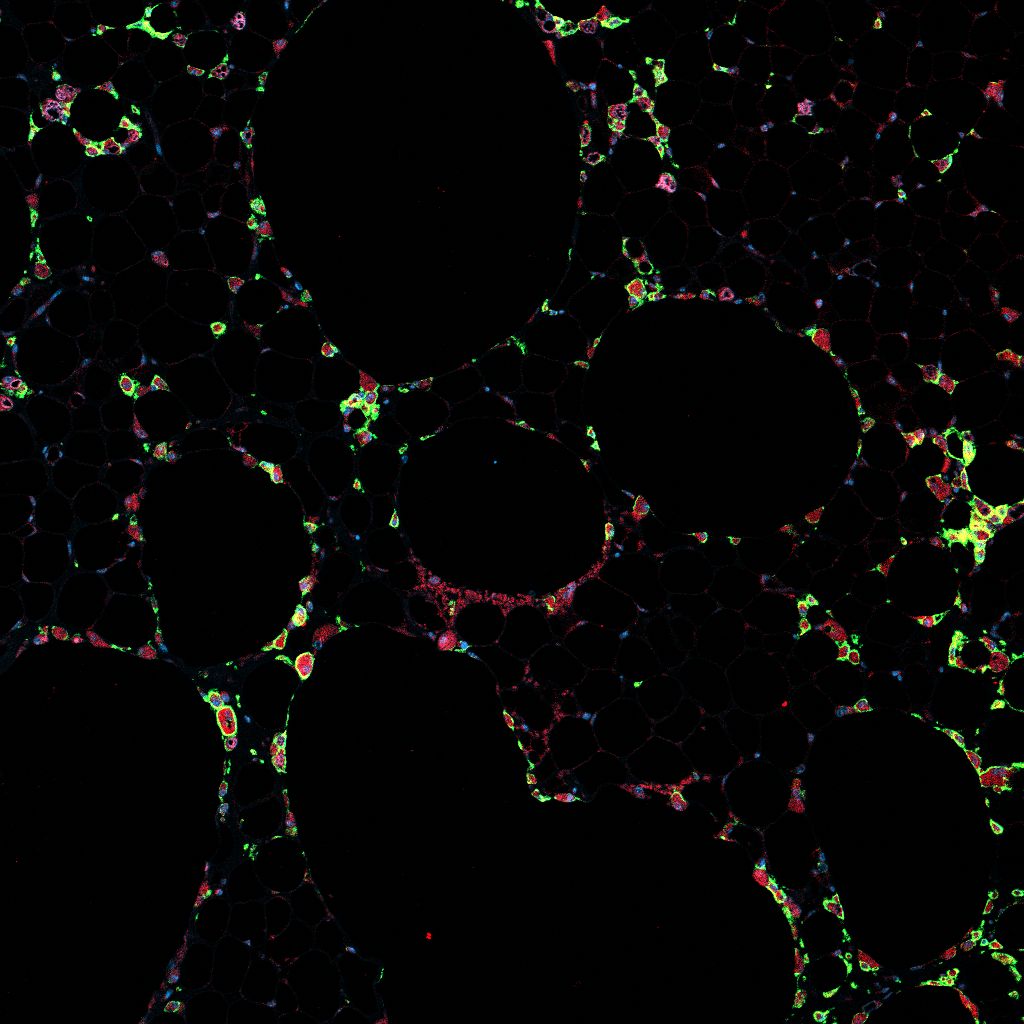

Supplement: Supplementary file 2 [file DataSheet1.ZIP › data for figure 4/IF ATGL F4-80 in ND-HC-HF 1M grafts/Hf-1M-F480-ATGL-09.jpg]

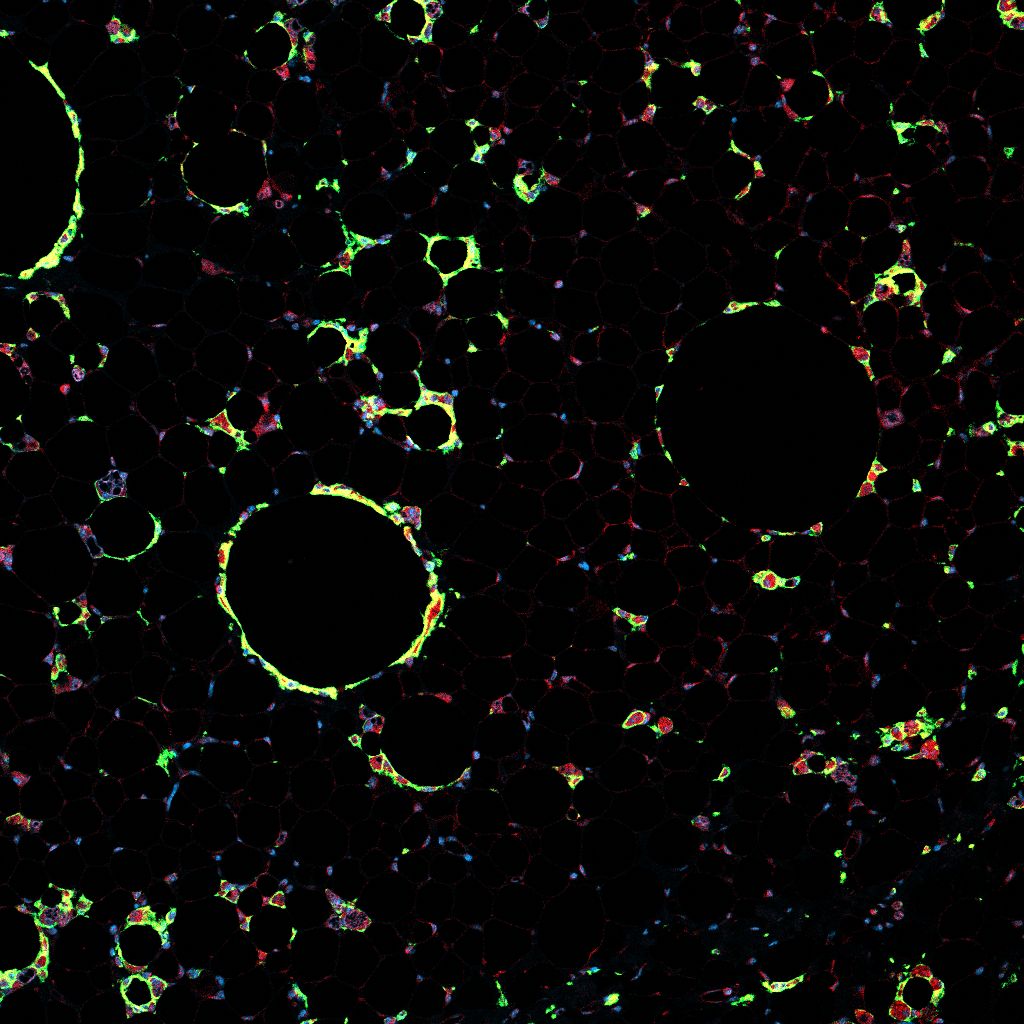

Supplement: Supplementary file 2 [file DataSheet1.ZIP › data for figure 4/IF ATGL F4-80 in ND-HC-HF 1M grafts/Hf-1M-F480-ATGL-10.jpg]

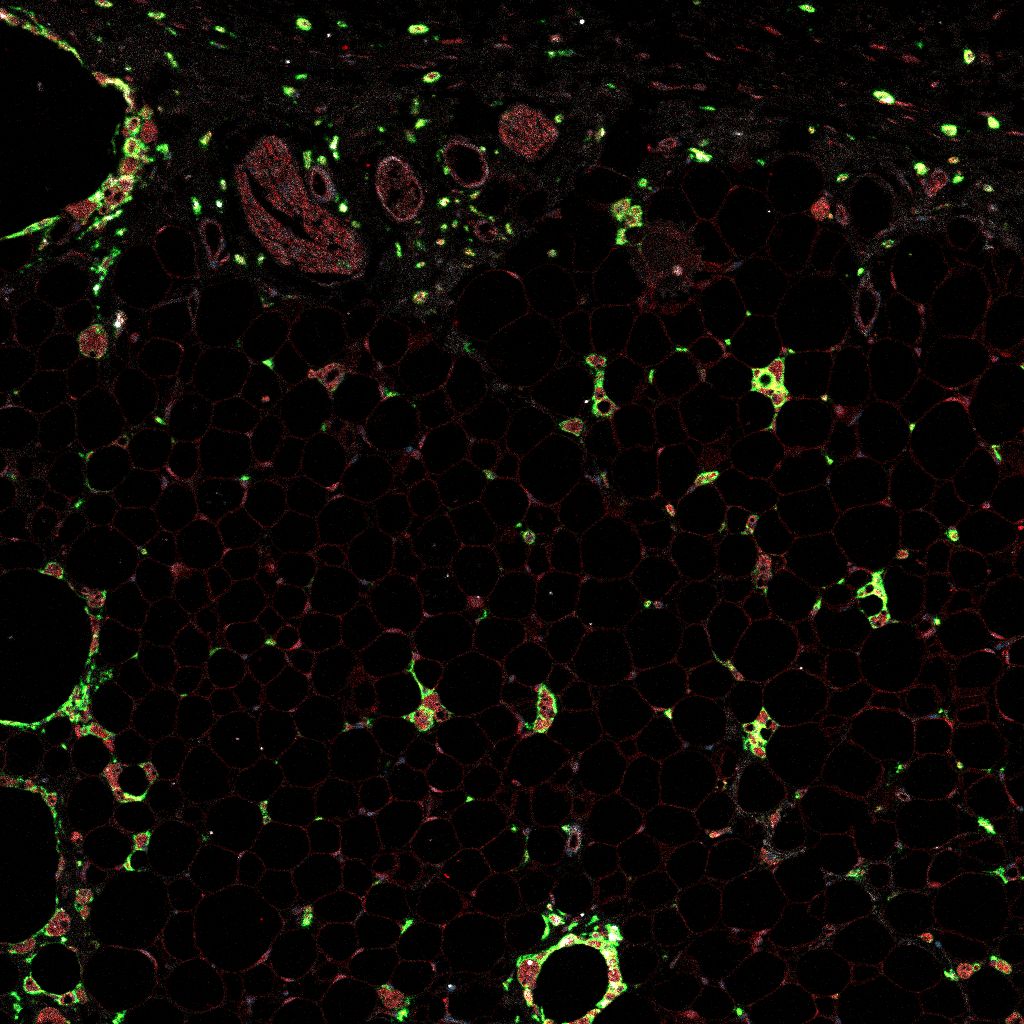

Supplement: Supplementary file 2 [file DataSheet1.ZIP › data for figure 4/IF ATGL F4-80 in ND-HC-HF 1M grafts/Hf-1M-F480-ATGL-12.jpg]

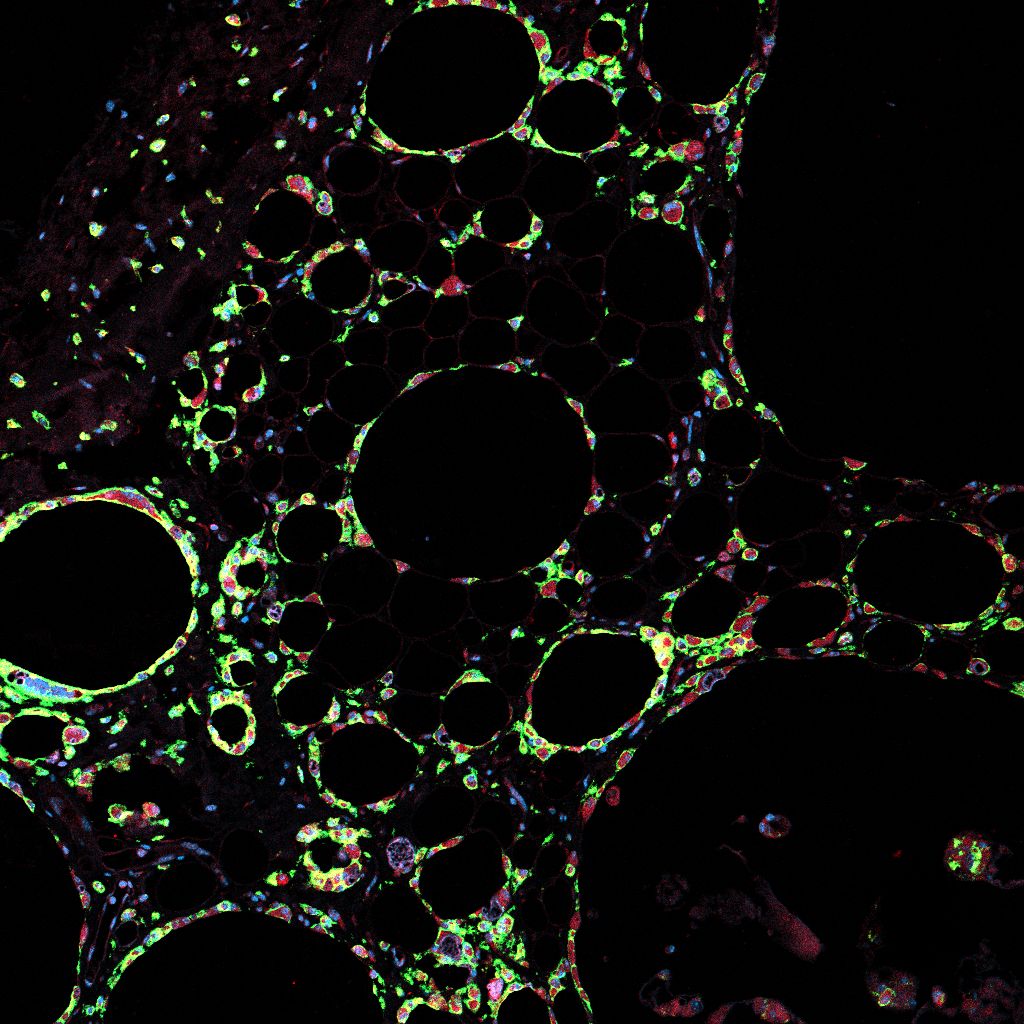

Supplement: Supplementary file 2 [file DataSheet1.ZIP › data for figure 4/IF ATGL F4-80 in ND-HC-HF 1M grafts/Hf-1M-F480-ATGL-13.jpg]

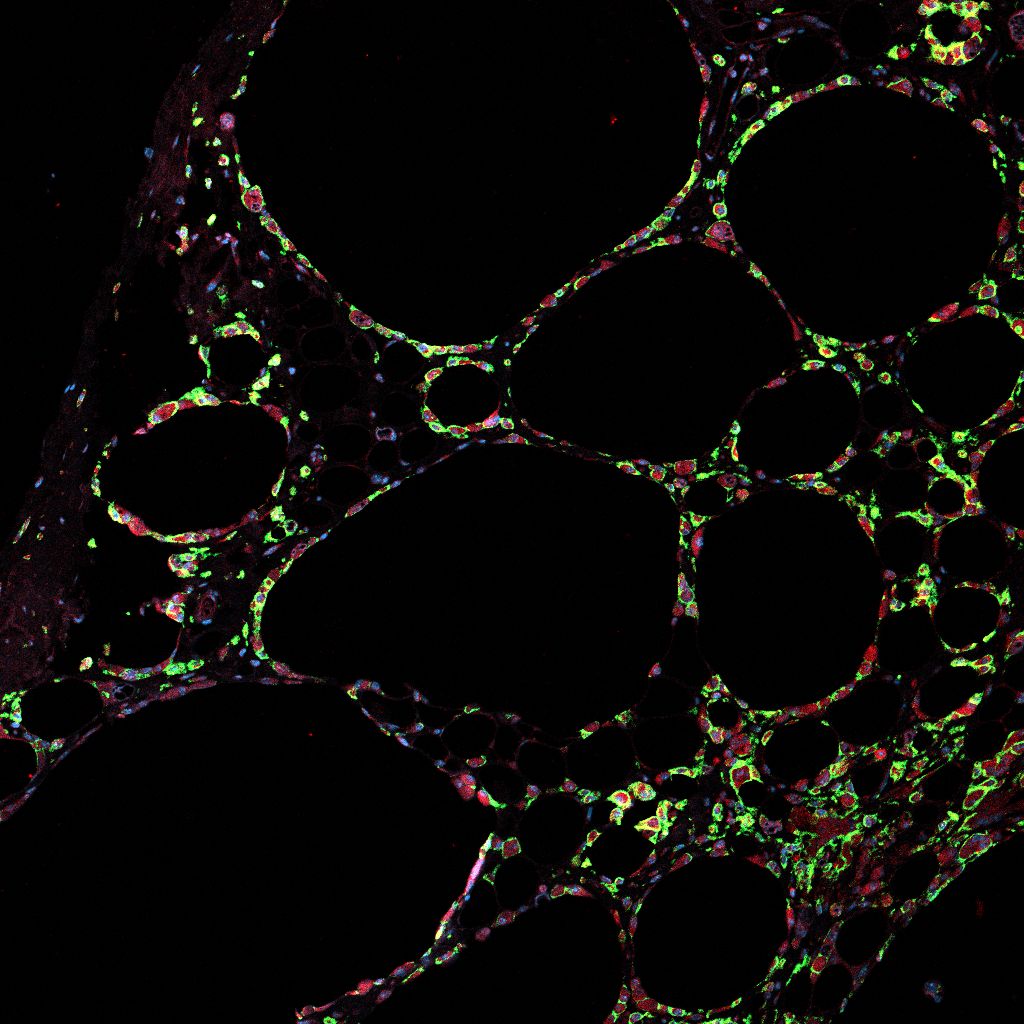

Supplement: Supplementary file 2 [file DataSheet1.ZIP › data for figure 4/IF ATGL F4-80 in ND-HC-HF 1M grafts/Hf-1M-F480-ATGL-14.jpg]

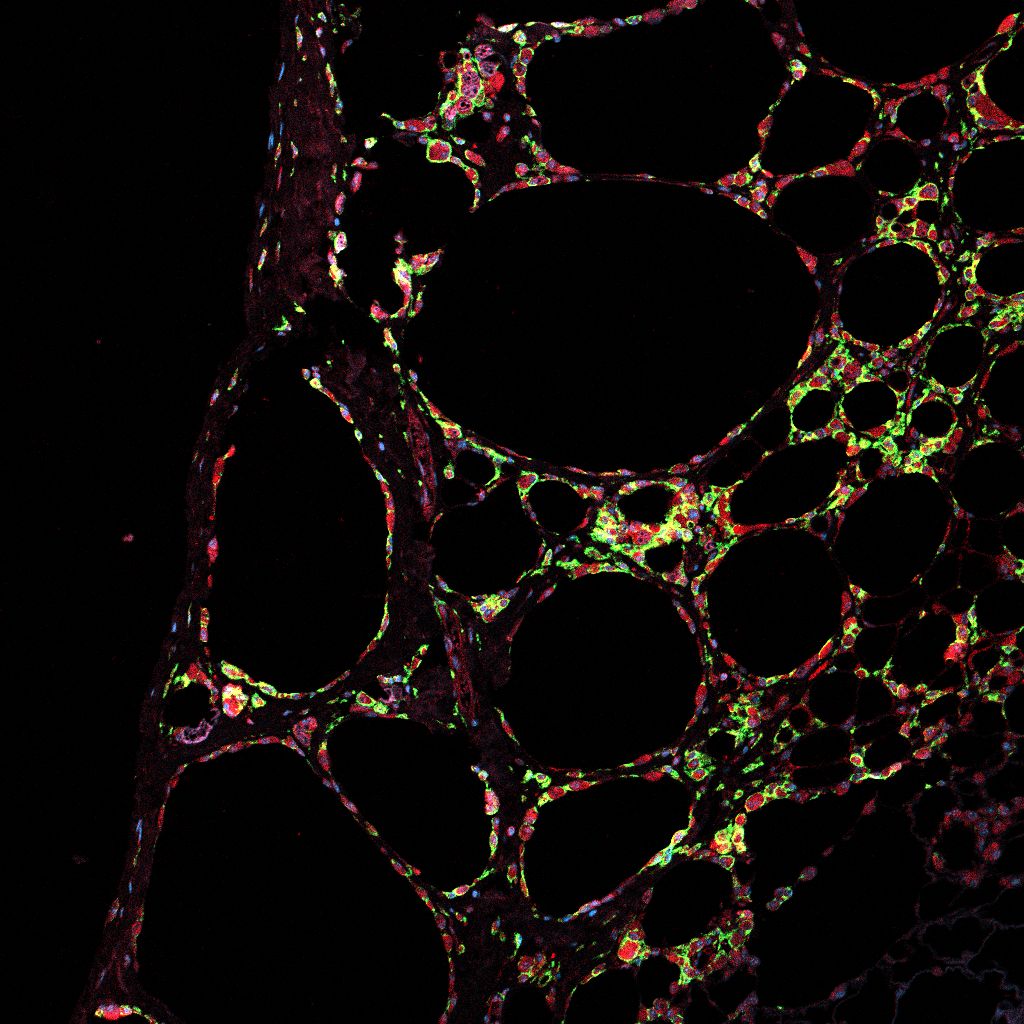

Supplement: Supplementary file 2 [file DataSheet1.ZIP › data for figure 4/IF ATGL F4-80 in ND-HC-HF 1M grafts/Hf-1M-F480-ATGL-15.jpg]

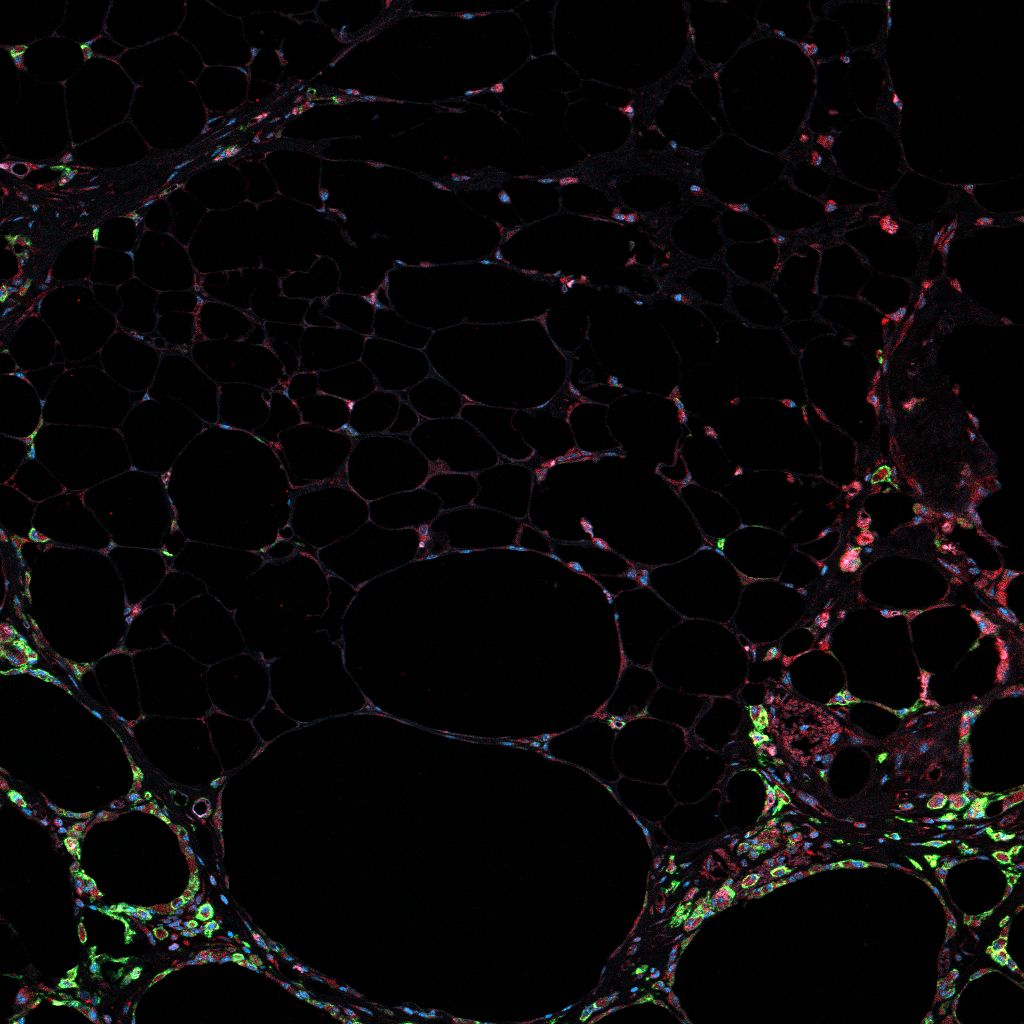

Supplement: Supplementary file 2 [file DataSheet1.ZIP › data for figure 4/IF ATGL F4-80 in ND-HC-HF 1M grafts/ND-1M-F480-ATGL-02 used in figure.jpg]

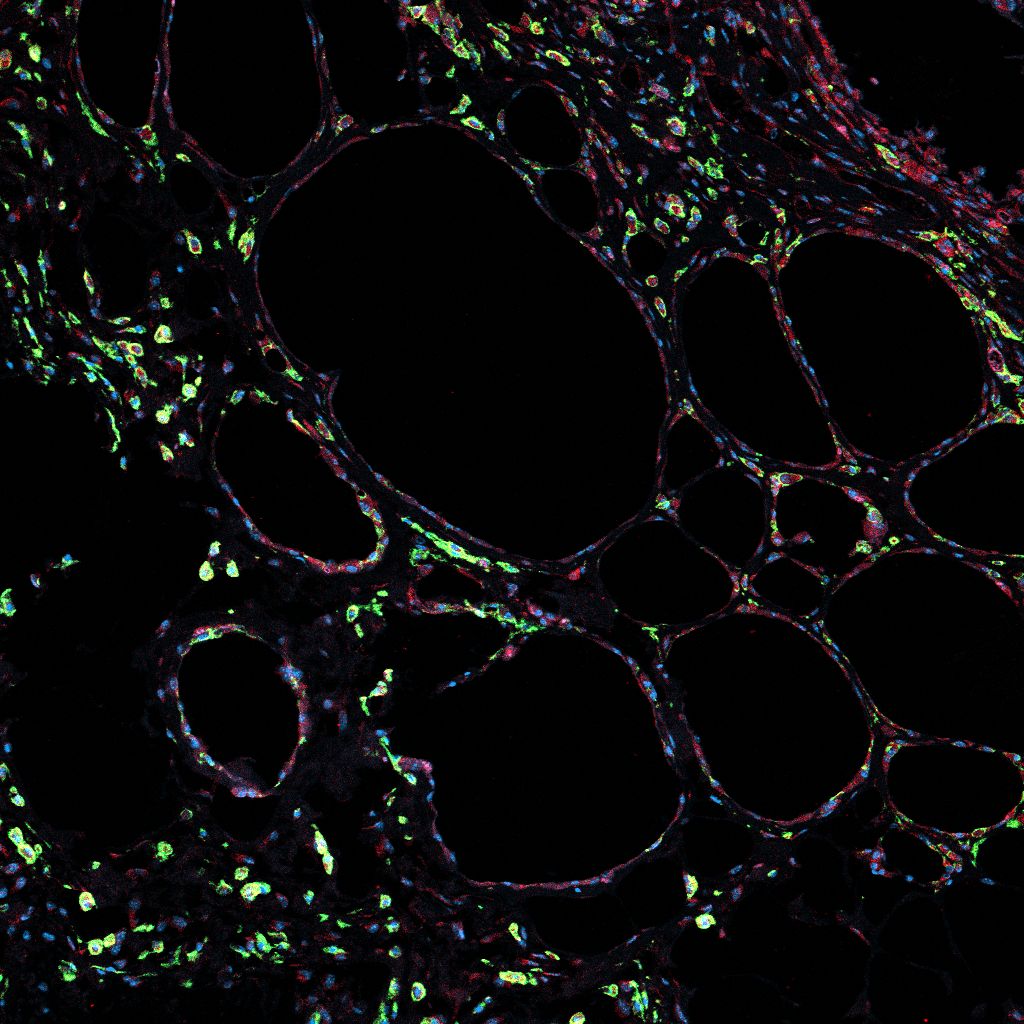

Supplement: Supplementary file 2 [file DataSheet1.ZIP › data for figure 4/IF ATGL F4-80 in ND-HC-HF 1M grafts/ND-1M-F480-ATGL-03.jpg]

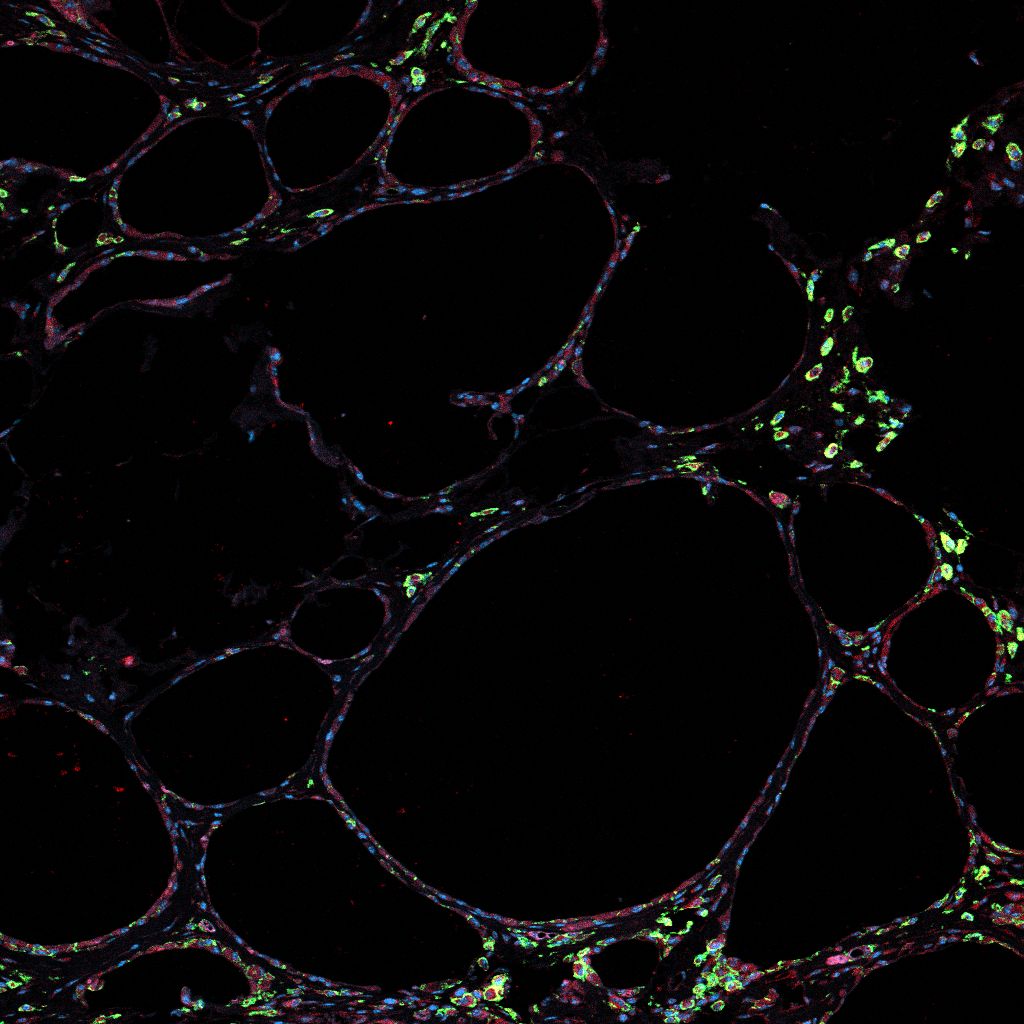

Supplement: Supplementary file 2 [file DataSheet1.ZIP › data for figure 4/IF ATGL F4-80 in ND-HC-HF 1M grafts/ND-1M-F480-ATGL-04.jpg]

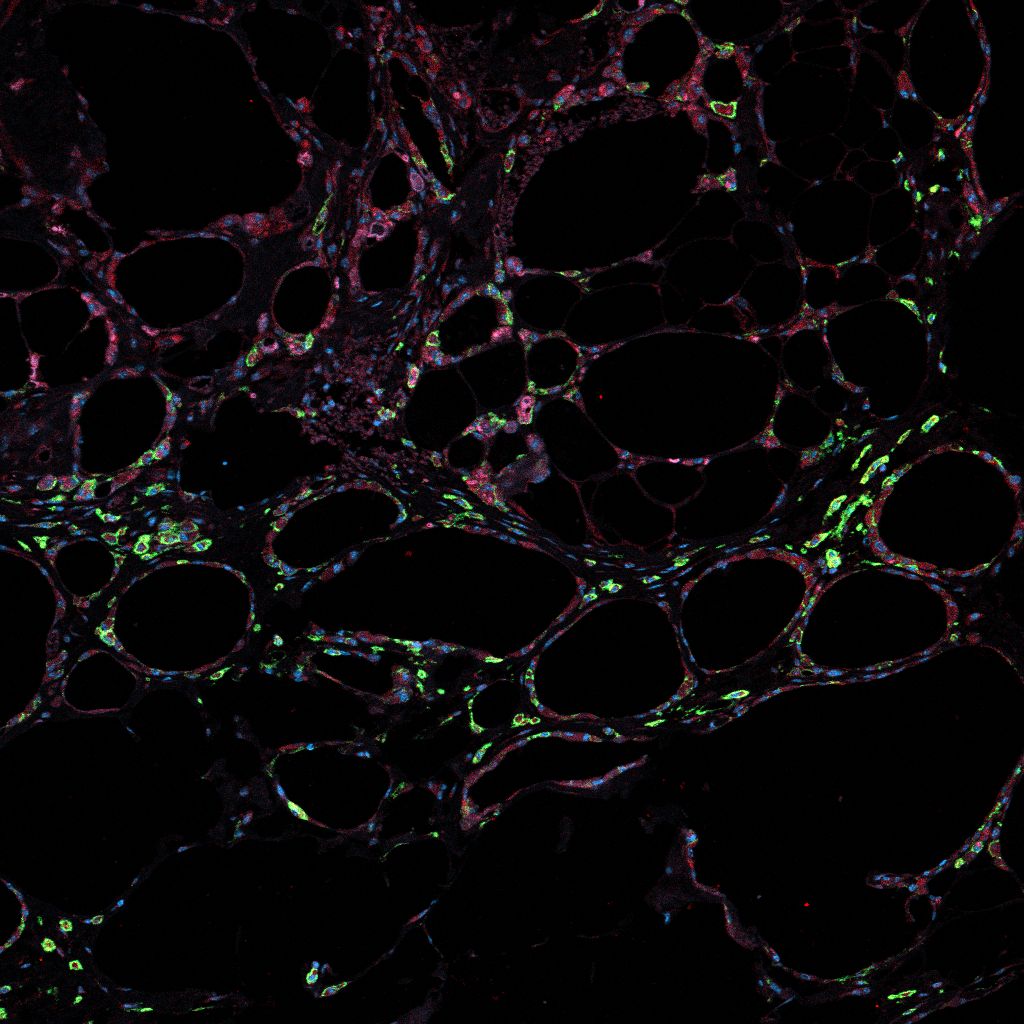

Supplement: Supplementary file 2 [file DataSheet1.ZIP › data for figure 4/IF ATGL F4-80 in ND-HC-HF 1M grafts/ND-1M-F480-ATGL-06.jpg]

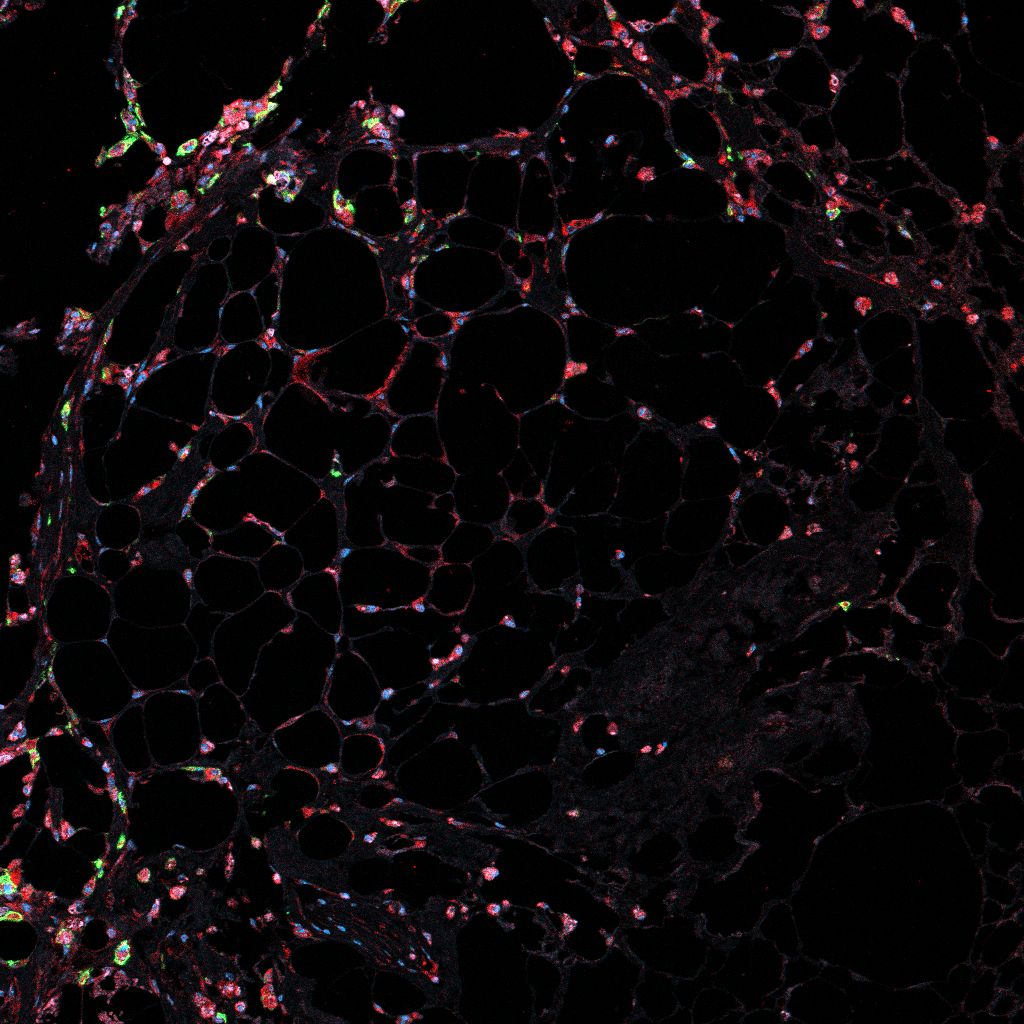

Supplement: Supplementary file 2 [file DataSheet1.ZIP › data for figure 4/IF ATGL F4-80 in ND-HC-HF 1M grafts/ND-1M-F480-ATGL08.jpg]

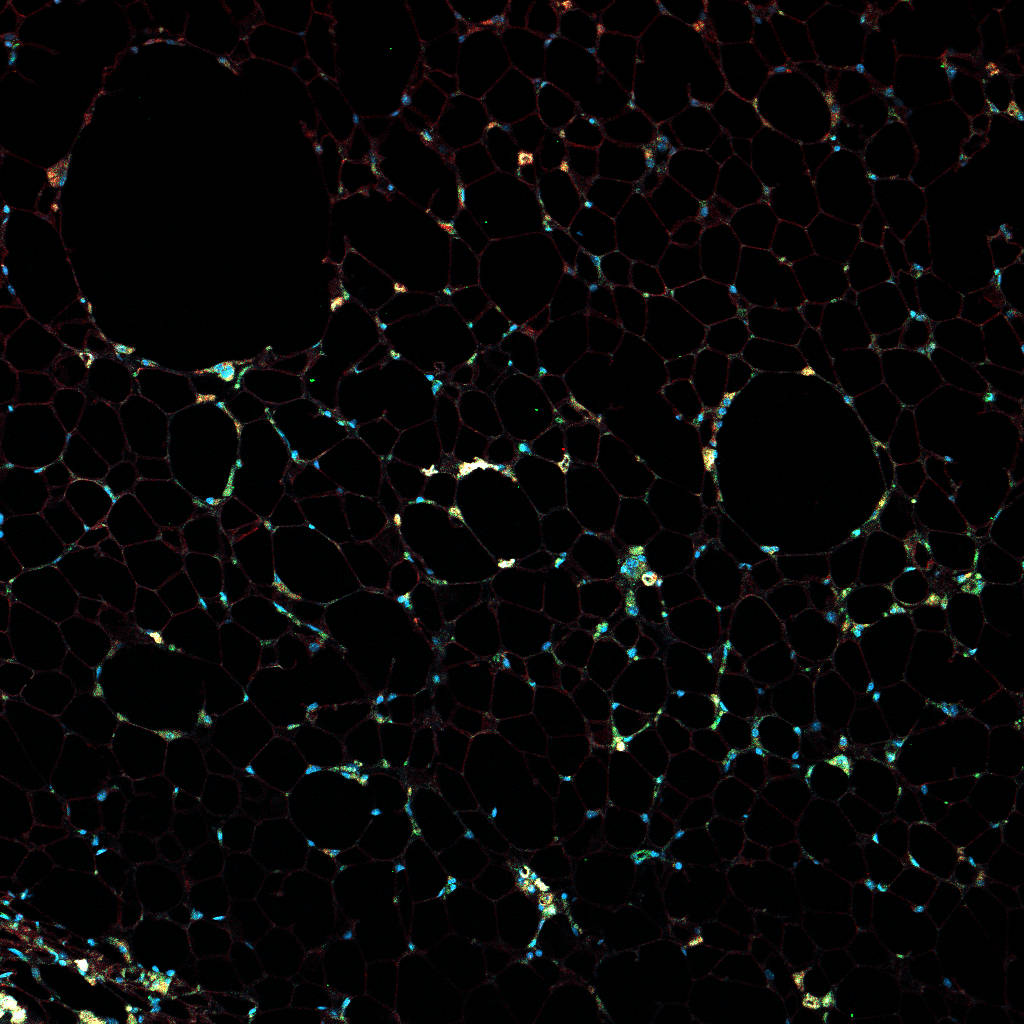

Supplement: Supplementary file 2 [file DataSheet1.ZIP › data for figure 4/IF CD31 Perilipin in ND-HC-HF 3M grafts/HC-3M CD31-Peri-10X-07 used in figure.tiff]

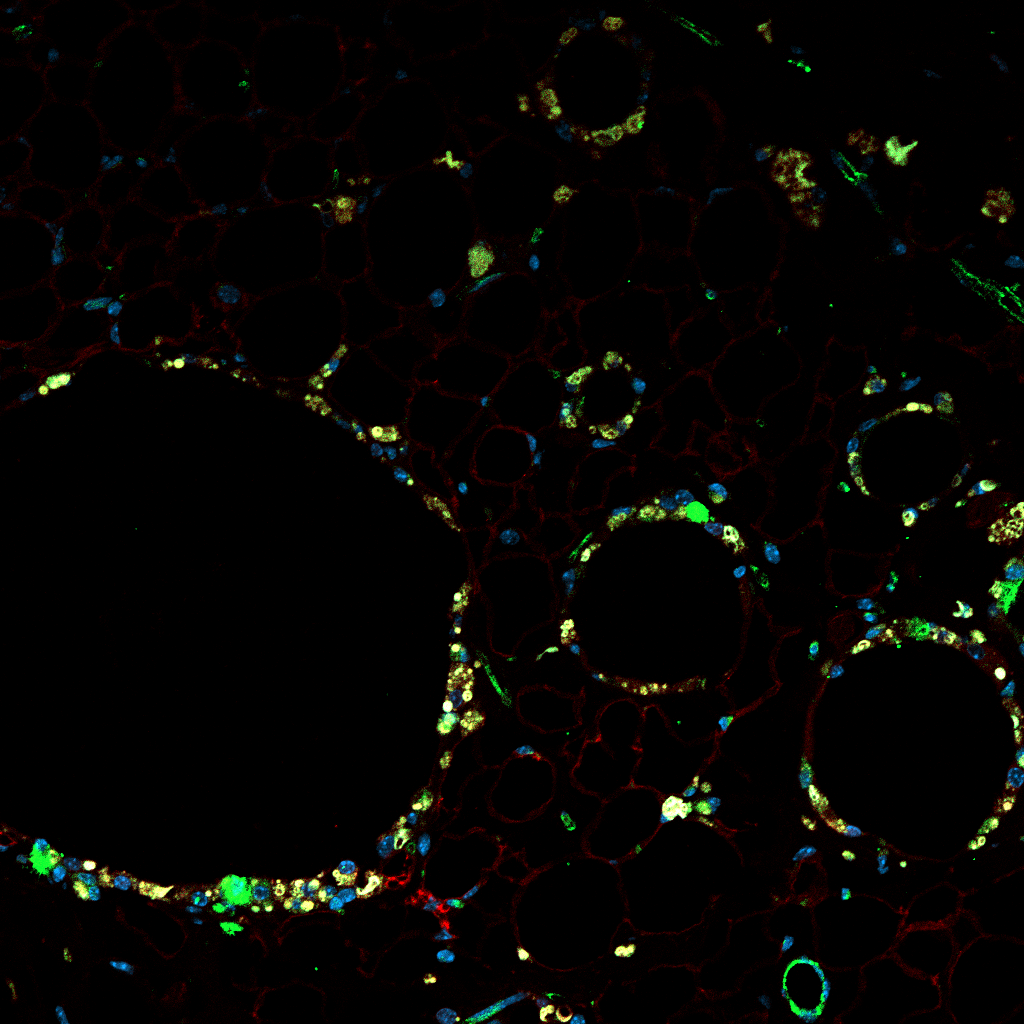

Supplement: Supplementary file 2 [file DataSheet1.ZIP › data for figure 4/IF CD31 Perilipin in ND-HC-HF 3M grafts/HC-3M1 CD31-Peri-20X-01.tiff]

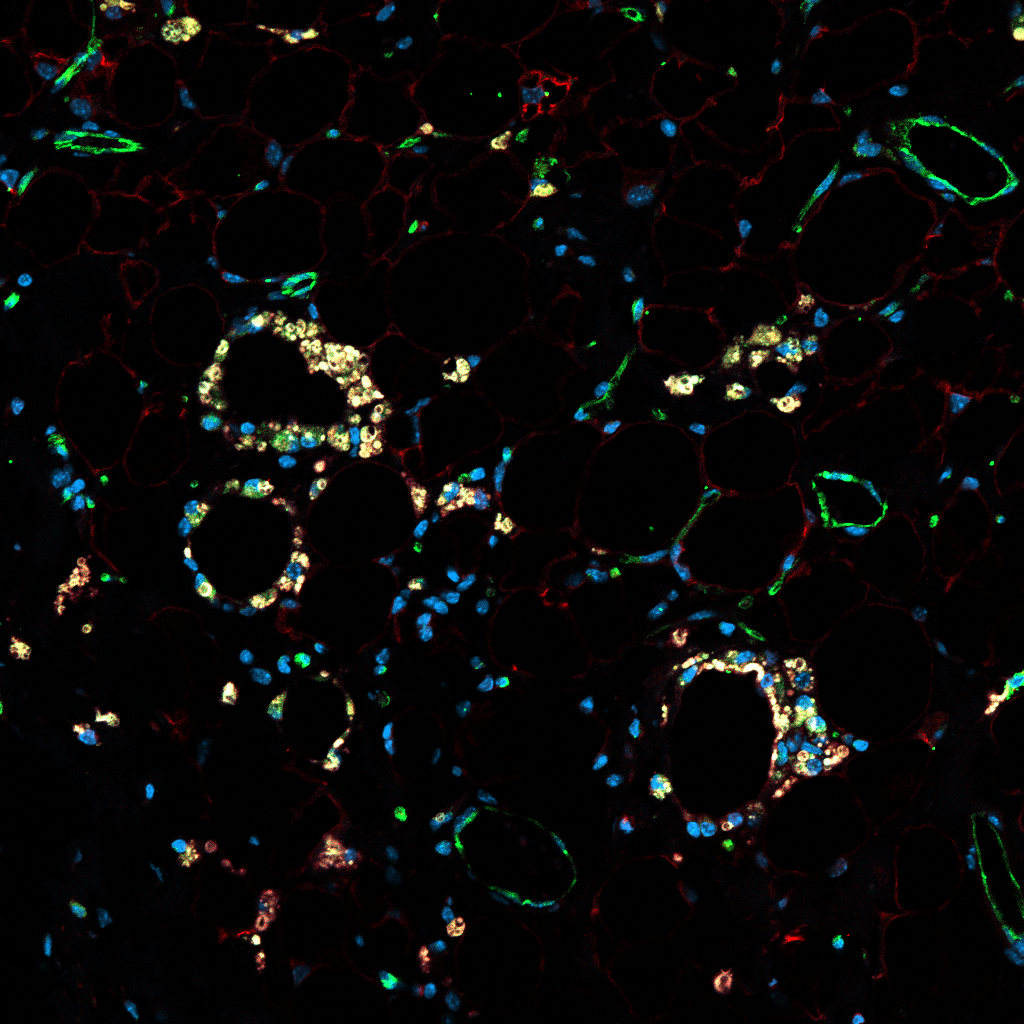

Supplement: Supplementary file 2 [file DataSheet1.ZIP › data for figure 4/IF CD31 Perilipin in ND-HC-HF 3M grafts/HC-3M1 CD31-Peri-20X-02.tiff]

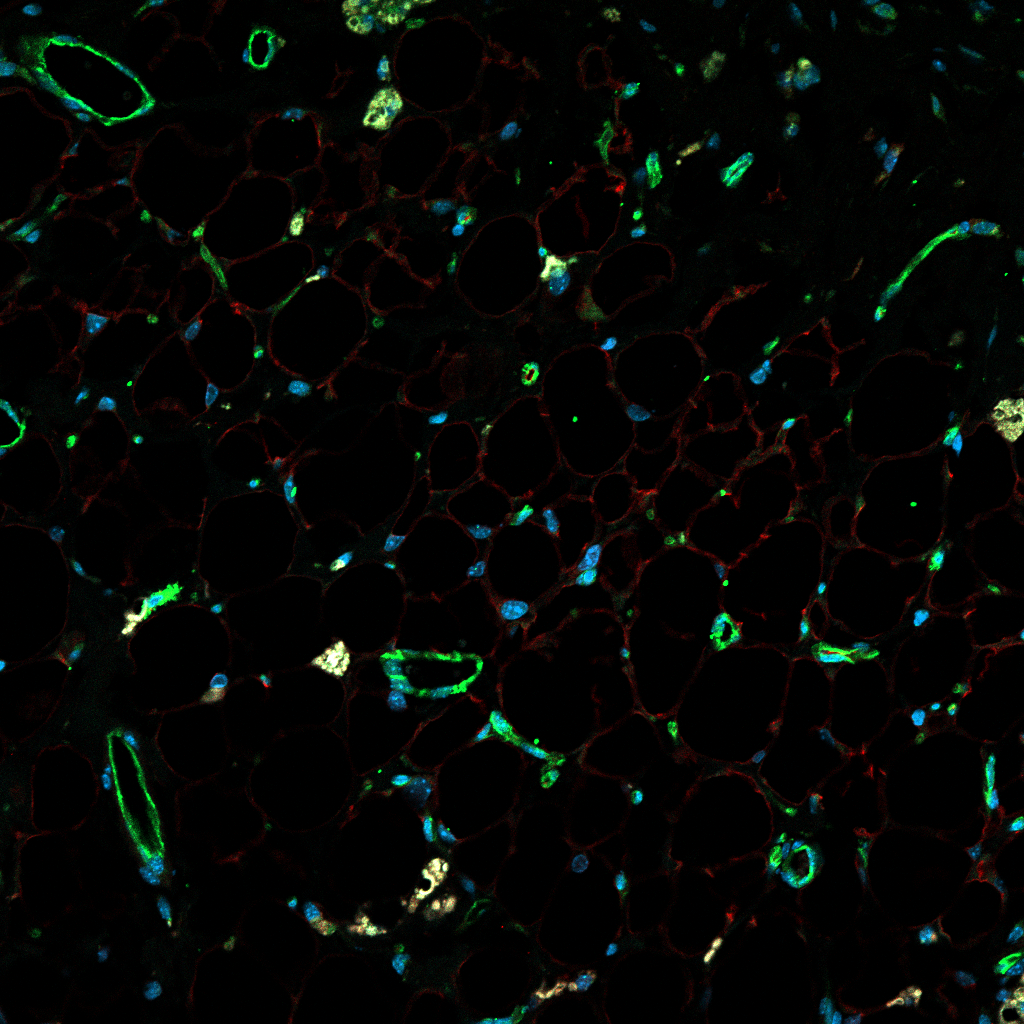

Supplement: Supplementary file 2 [file DataSheet1.ZIP › data for figure 4/IF CD31 Perilipin in ND-HC-HF 3M grafts/HC-3M1 CD31-Peri-20X-05.tiff]

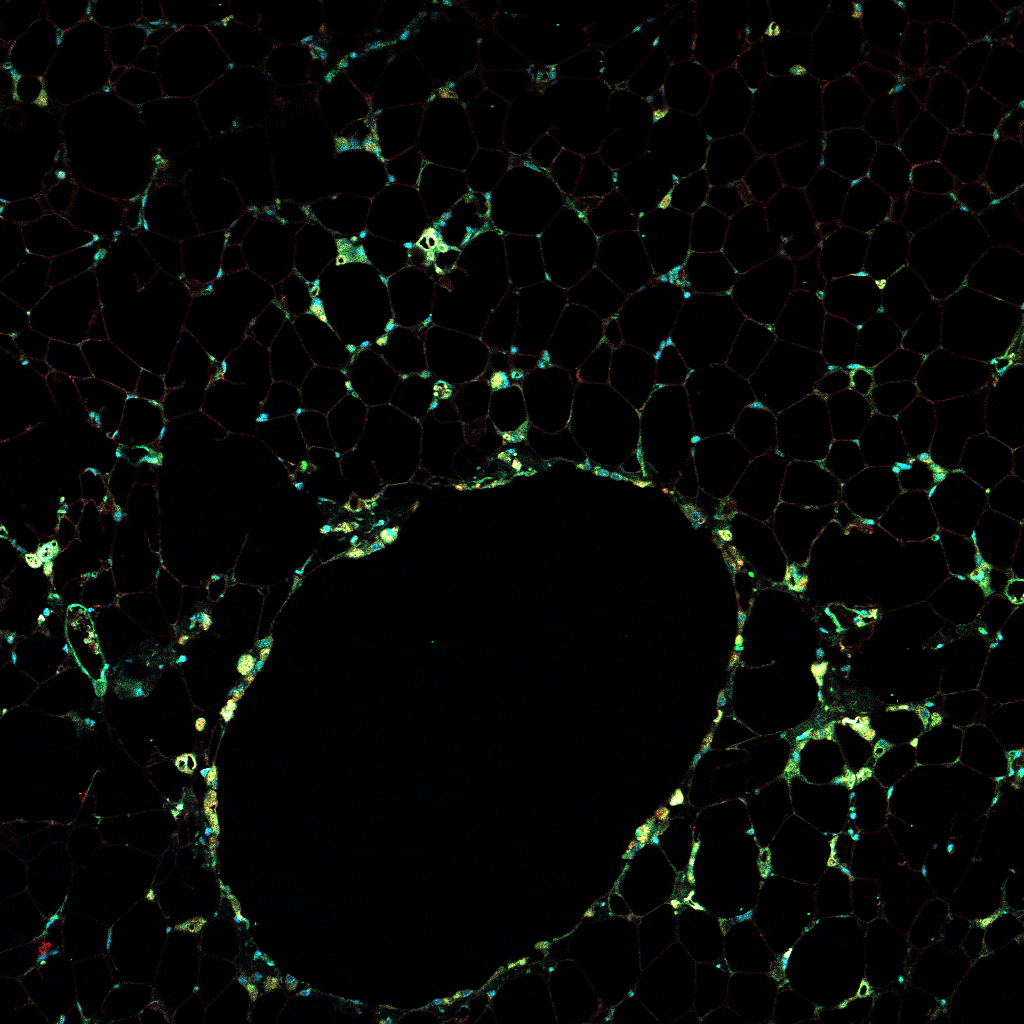

Supplement: Supplementary file 2 [file DataSheet1.ZIP › data for figure 4/IF CD31 Perilipin in ND-HC-HF 3M grafts/HF-3M CD31-Peri-10X-01.tiff]

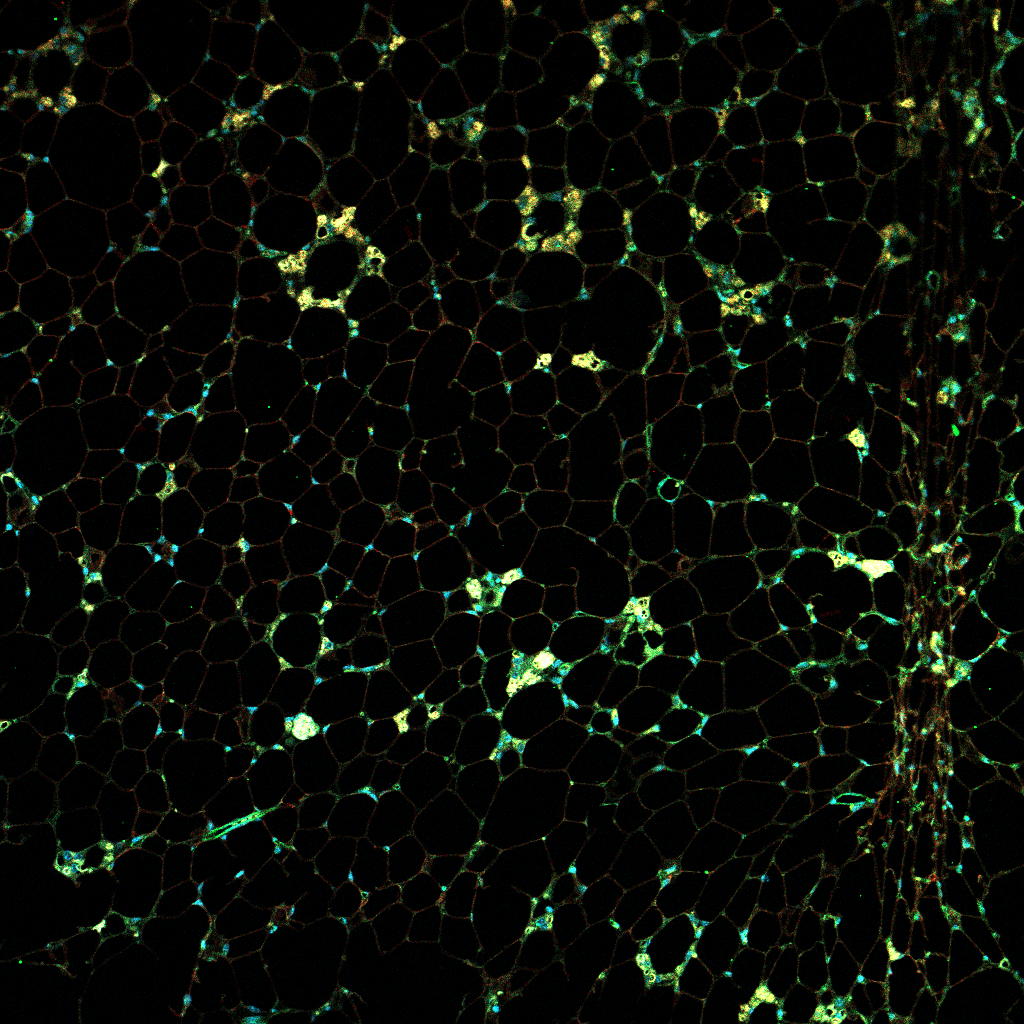

Supplement: Supplementary file 2 [file DataSheet1.ZIP › data for figure 4/IF CD31 Perilipin in ND-HC-HF 3M grafts/HF-3M CD31-Peri-10X-02.tif]

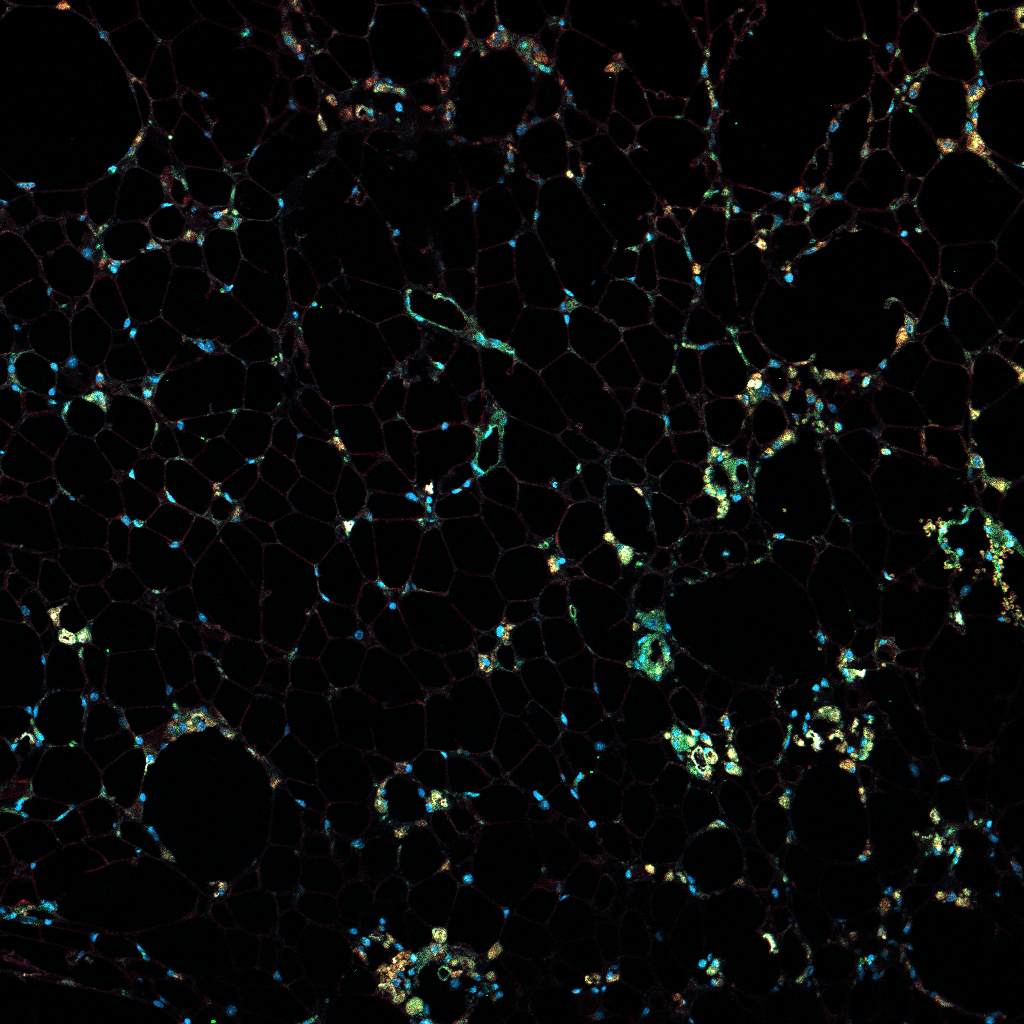

Supplement: Supplementary file 2 [file DataSheet1.ZIP › data for figure 4/IF CD31 Perilipin in ND-HC-HF 3M grafts/HF-3M CD31-Peri-10X-06 used in fgure.tiff]

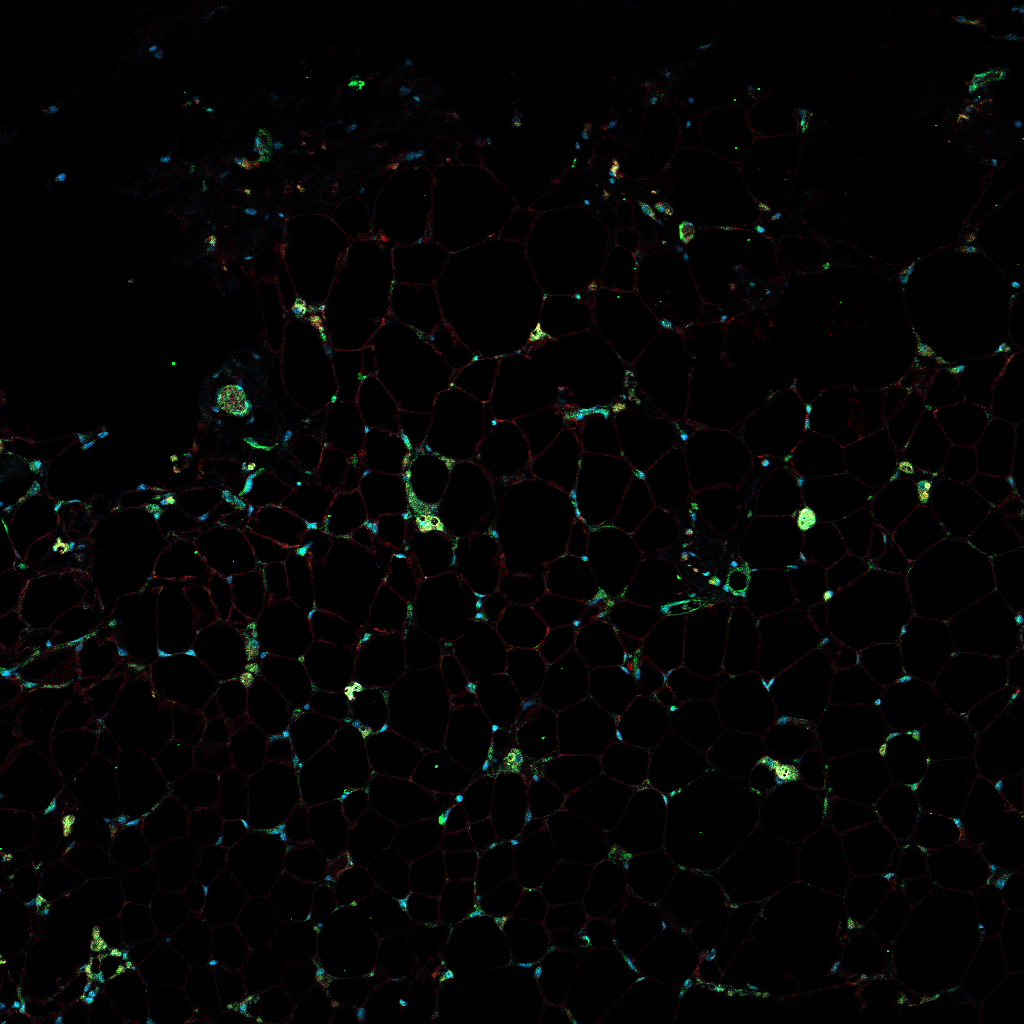

Supplement: Supplementary file 2 [file DataSheet1.ZIP › data for figure 4/IF CD31 Perilipin in ND-HC-HF 3M grafts/ND-3M CD31-Peri-10X-03 used in figure.tiff]

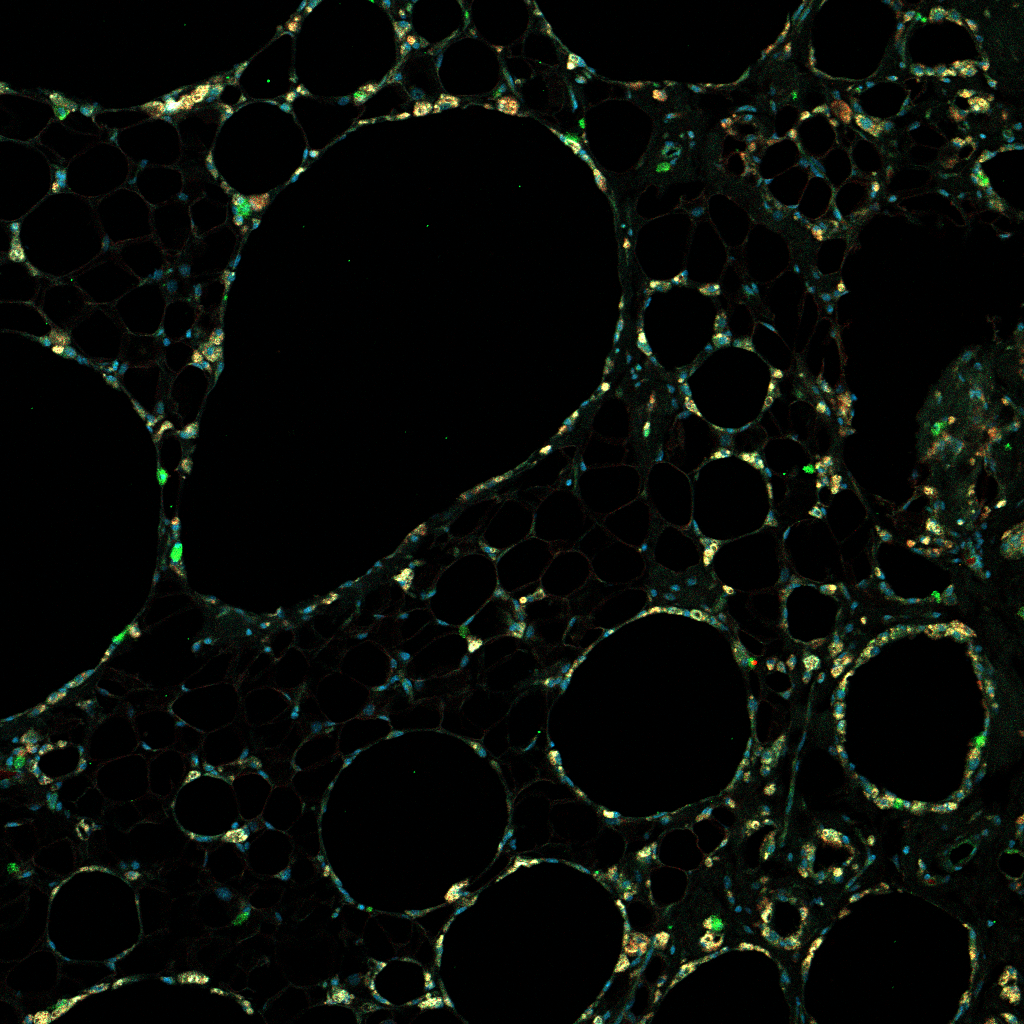

Supplement: Supplementary file 2 [file DataSheet1.ZIP › data for figure 4/IF CD31 Perilipin in ND-HC-HF 3M grafts/ND-3M CD31-Peri-10X-03.tiff]

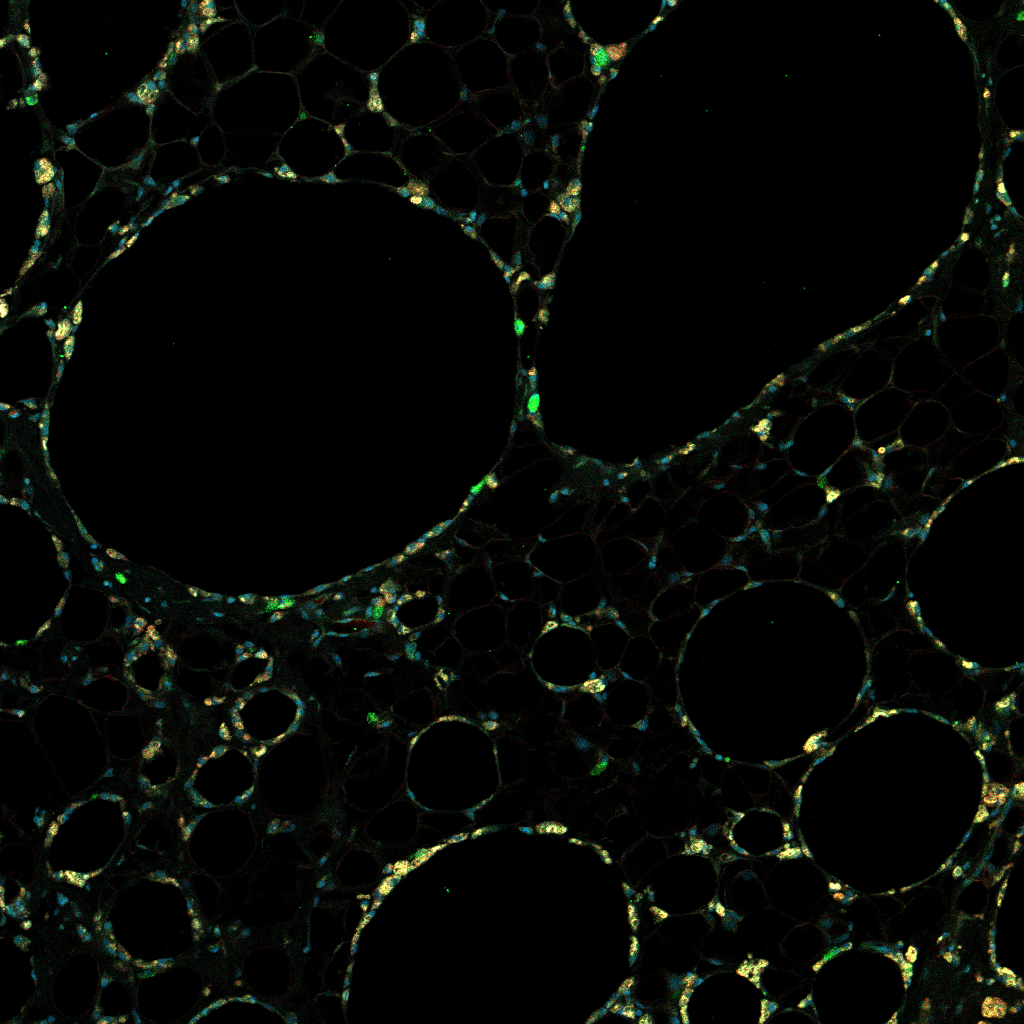

Supplement: Supplementary file 2 [file DataSheet1.ZIP › data for figure 4/IF CD31 Perilipin in ND-HC-HF 3M grafts/ND-3M CD31-Peri-10X-04.tiff]

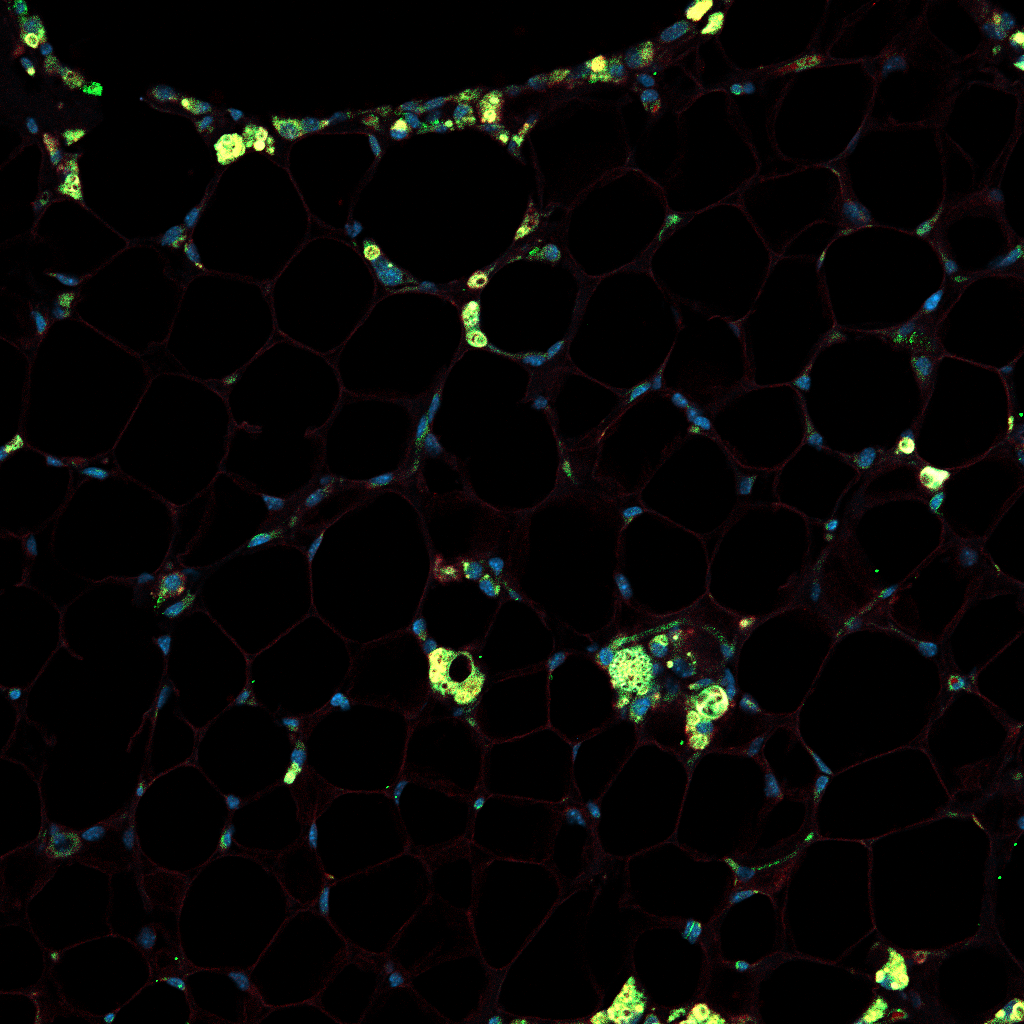

Supplement: Supplementary file 2 [file DataSheet1.ZIP › data for figure 4/IF CD31 Perilipin in ND-HC-HF 3M grafts/ND-3M CD31-Peri-20X-02.tiff]

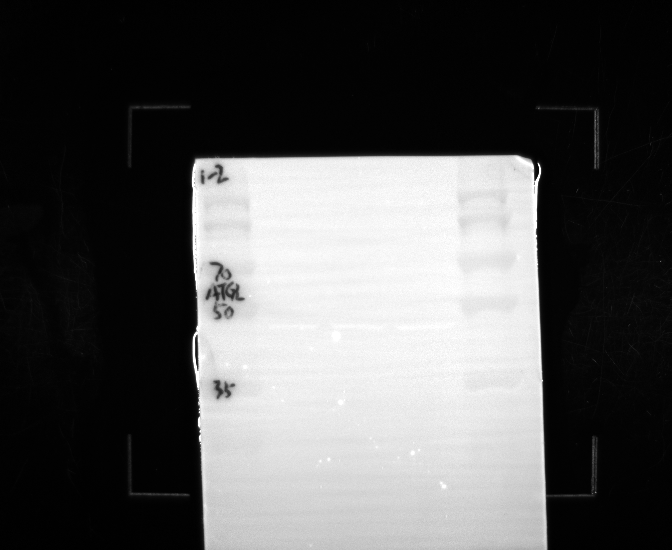

Supplement: Supplementary file 2 [file DataSheet1.ZIP › data for figure 4/Western blot of ATGL HSL p-HSL in ND-HC-HF 1M grafts/ATGL/004-bright[20220117-185026-ZDY].tif]

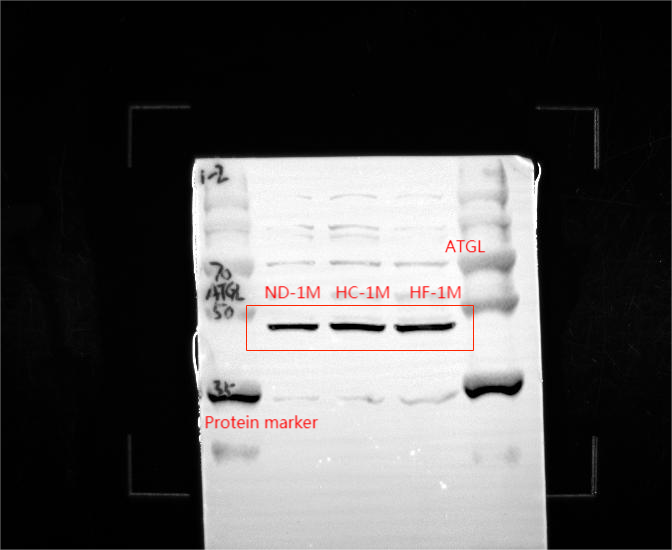

Supplement: Supplementary file 2 [file DataSheet1.ZIP › data for figure 4/Western blot of ATGL HSL p-HSL in ND-HC-HF 1M grafts/ATGL/004-merger[20220117-185026-ZDY].png]

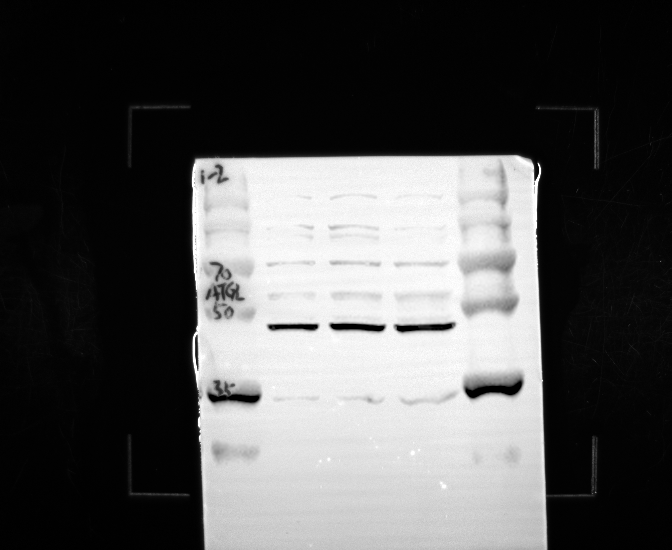

Supplement: Supplementary file 2 [file DataSheet1.ZIP › data for figure 4/Western blot of ATGL HSL p-HSL in ND-HC-HF 1M grafts/ATGL/004-merger[20220117-185026-ZDY].tif]

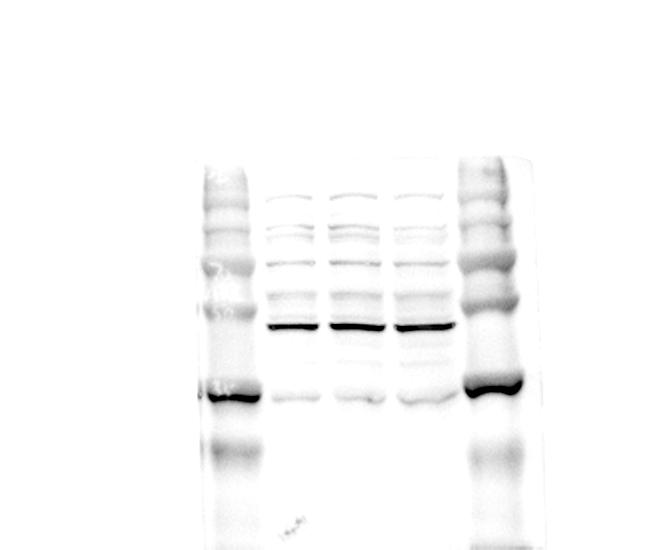

Supplement: Supplementary file 2 [file DataSheet1.ZIP › data for figure 4/Western blot of ATGL HSL p-HSL in ND-HC-HF 1M grafts/ATGL/004-shine[20220117-185026-ZDY].tif]

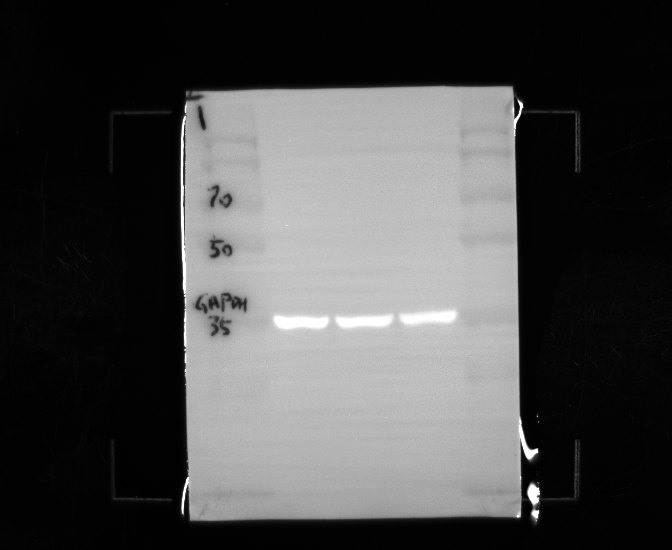

Supplement: Supplementary file 2 [file DataSheet1.ZIP › data for figure 4/Western blot of ATGL HSL p-HSL in ND-HC-HF 1M grafts/GAPDH/005-bright[20220117-183406-ZDY].tif]

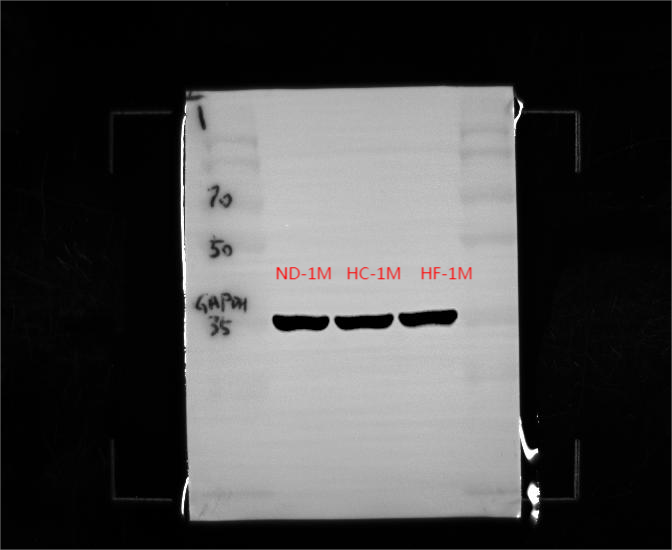

Supplement: Supplementary file 2 [file DataSheet1.ZIP › data for figure 4/Western blot of ATGL HSL p-HSL in ND-HC-HF 1M grafts/GAPDH/005-merger[20220117-183406-ZDY].png]

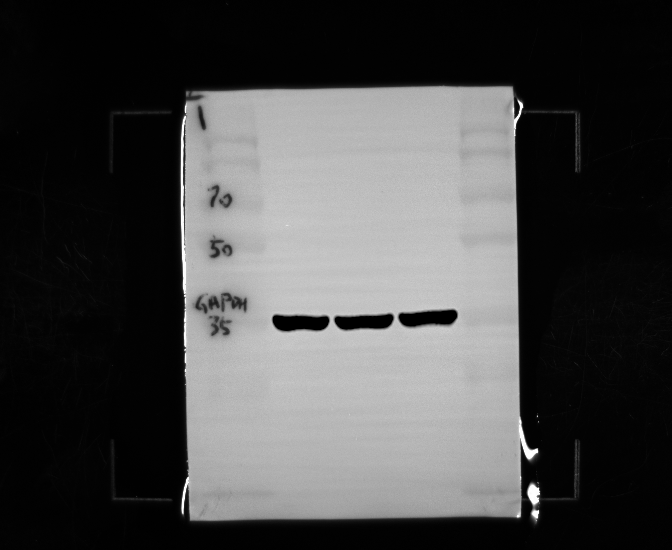

Supplement: Supplementary file 2 [file DataSheet1.ZIP › data for figure 4/Western blot of ATGL HSL p-HSL in ND-HC-HF 1M grafts/GAPDH/005-merger[20220117-183406-ZDY].tif]

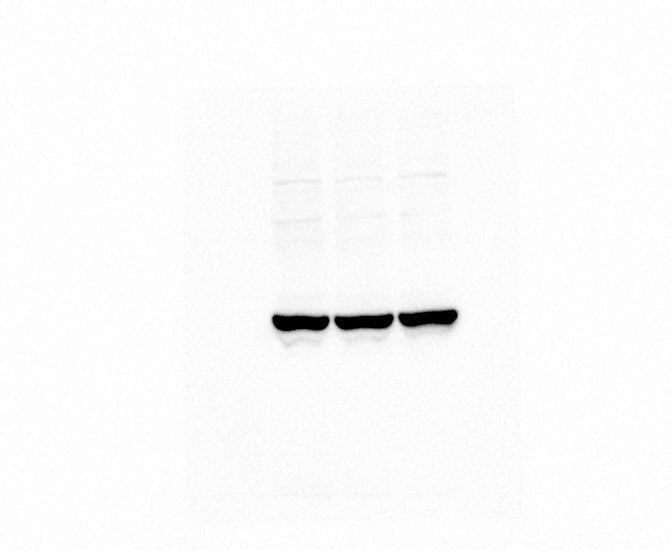

Supplement: Supplementary file 2 [file DataSheet1.ZIP › data for figure 4/Western blot of ATGL HSL p-HSL in ND-HC-HF 1M grafts/GAPDH/005-shine[20220117-183406-ZDY].tif]

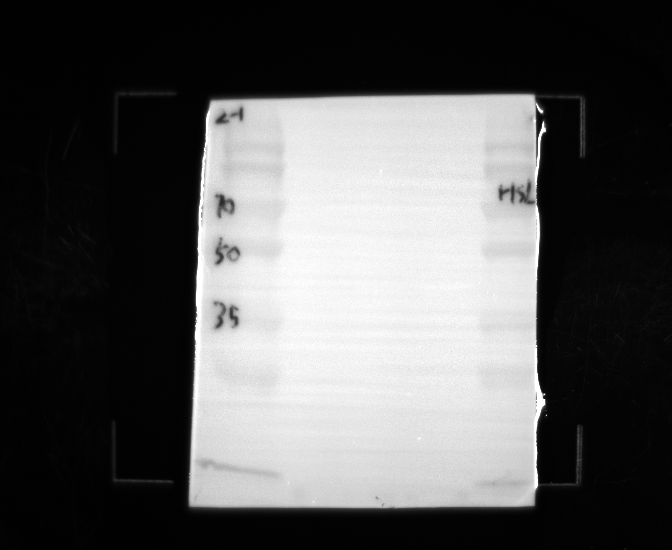

Supplement: Supplementary file 2 [file DataSheet1.ZIP › data for figure 4/Western blot of ATGL HSL p-HSL in ND-HC-HF 1M grafts/HSL/003-bright[20220117-183937-ZDY].tif]

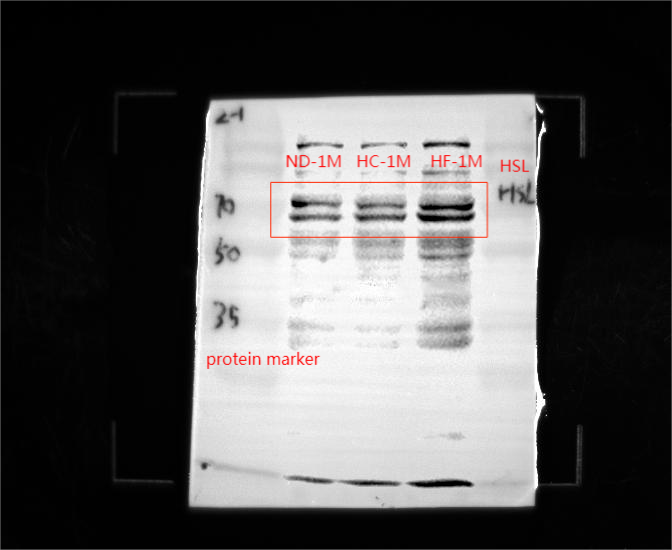

Supplement: Supplementary file 2 [file DataSheet1.ZIP › data for figure 4/Western blot of ATGL HSL p-HSL in ND-HC-HF 1M grafts/HSL/003-merger[20220117-183937-ZDY]-1.png]

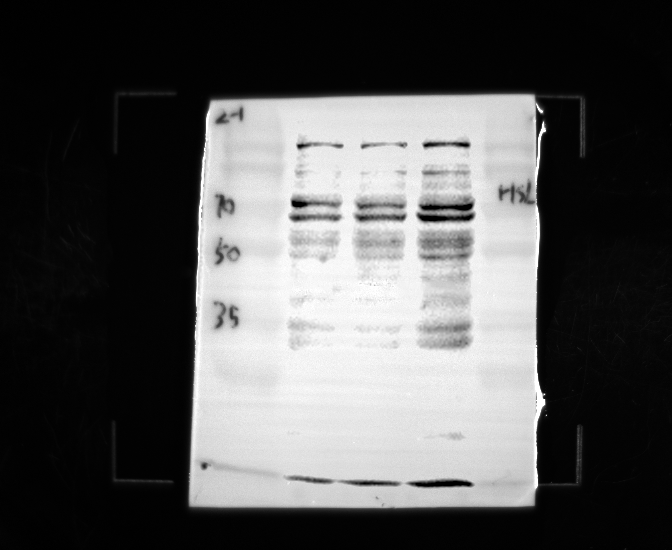

Supplement: Supplementary file 2 [file DataSheet1.ZIP › data for figure 4/Western blot of ATGL HSL p-HSL in ND-HC-HF 1M grafts/HSL/003-merger[20220117-183937-ZDY]-1.tif]

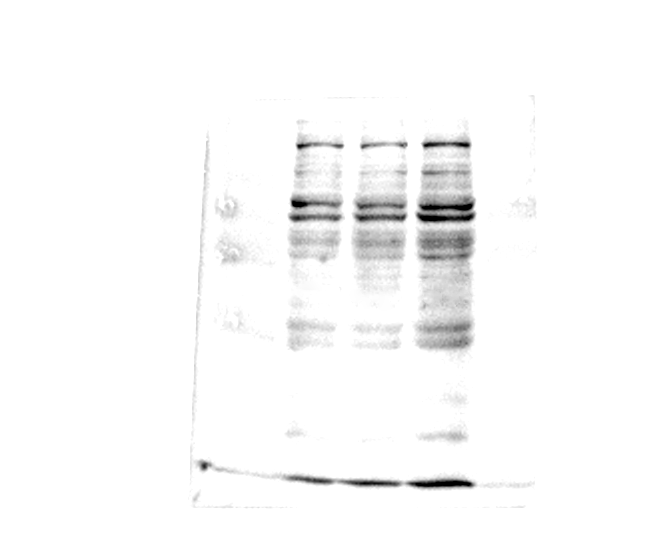

Supplement: Supplementary file 2 [file DataSheet1.ZIP › data for figure 4/Western blot of ATGL HSL p-HSL in ND-HC-HF 1M grafts/HSL/003-shine[20220117-183937-ZDY]-1.tif]

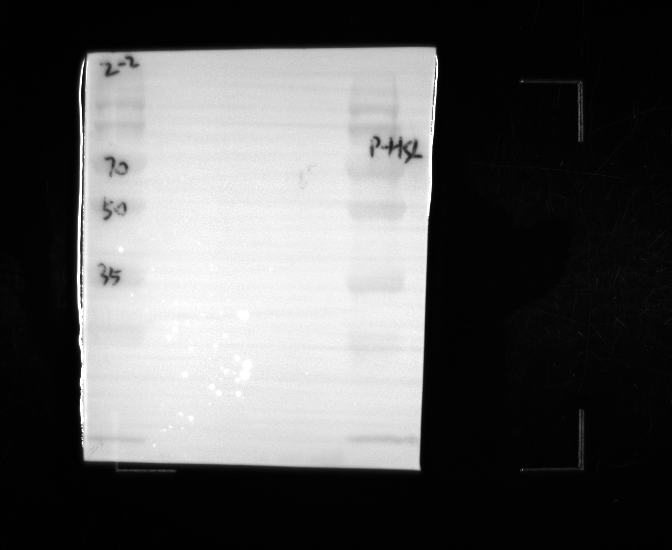

Supplement: Supplementary file 2 [file DataSheet1.ZIP › data for figure 4/Western blot of ATGL HSL p-HSL in ND-HC-HF 1M grafts/p-HSL/005-bright[20220117-184457-ZDY].tif]

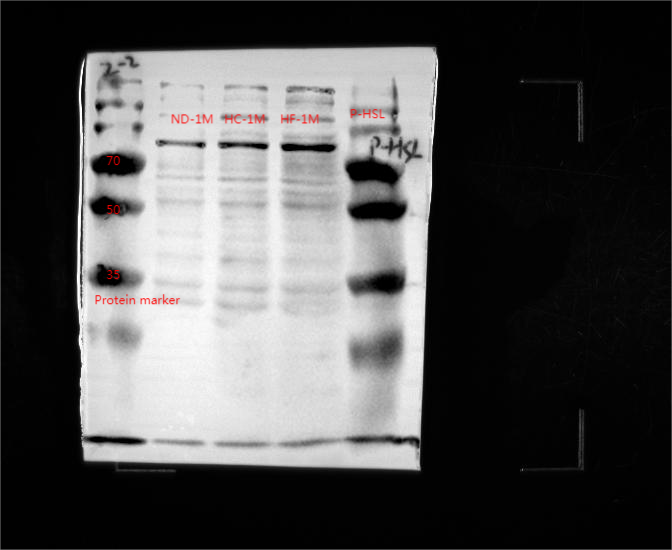

Supplement: Supplementary file 2 [file DataSheet1.ZIP › data for figure 4/Western blot of ATGL HSL p-HSL in ND-HC-HF 1M grafts/p-HSL/005-merger[20220117-184457-ZDY].png]

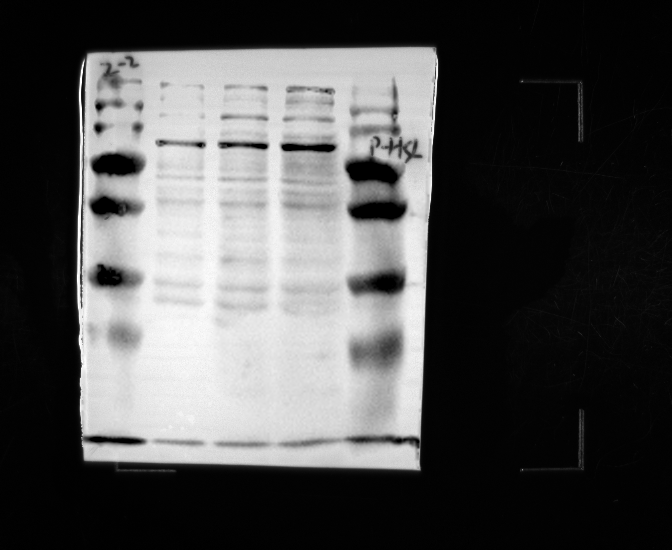

Supplement: Supplementary file 2 [file DataSheet1.ZIP › data for figure 4/Western blot of ATGL HSL p-HSL in ND-HC-HF 1M grafts/p-HSL/005-merger[20220117-184457-ZDY].tif]

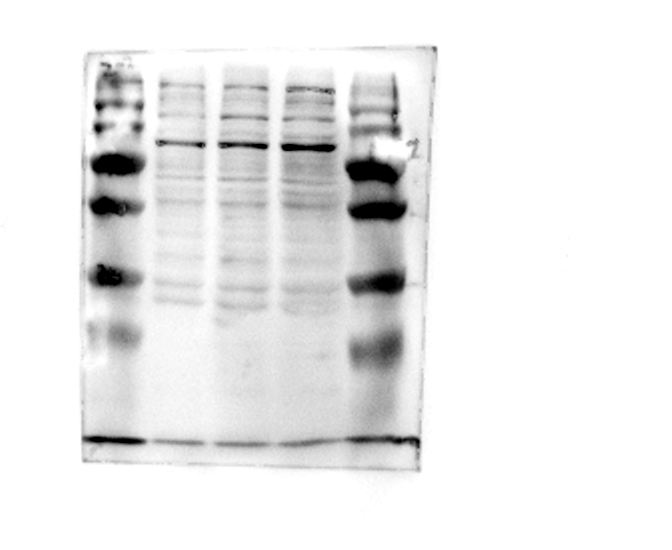

Supplement: Supplementary file 2 [file DataSheet1.ZIP › data for figure 4/Western blot of ATGL HSL p-HSL in ND-HC-HF 1M grafts/p-HSL/005-shine[20220117-184457-ZDY].tif]

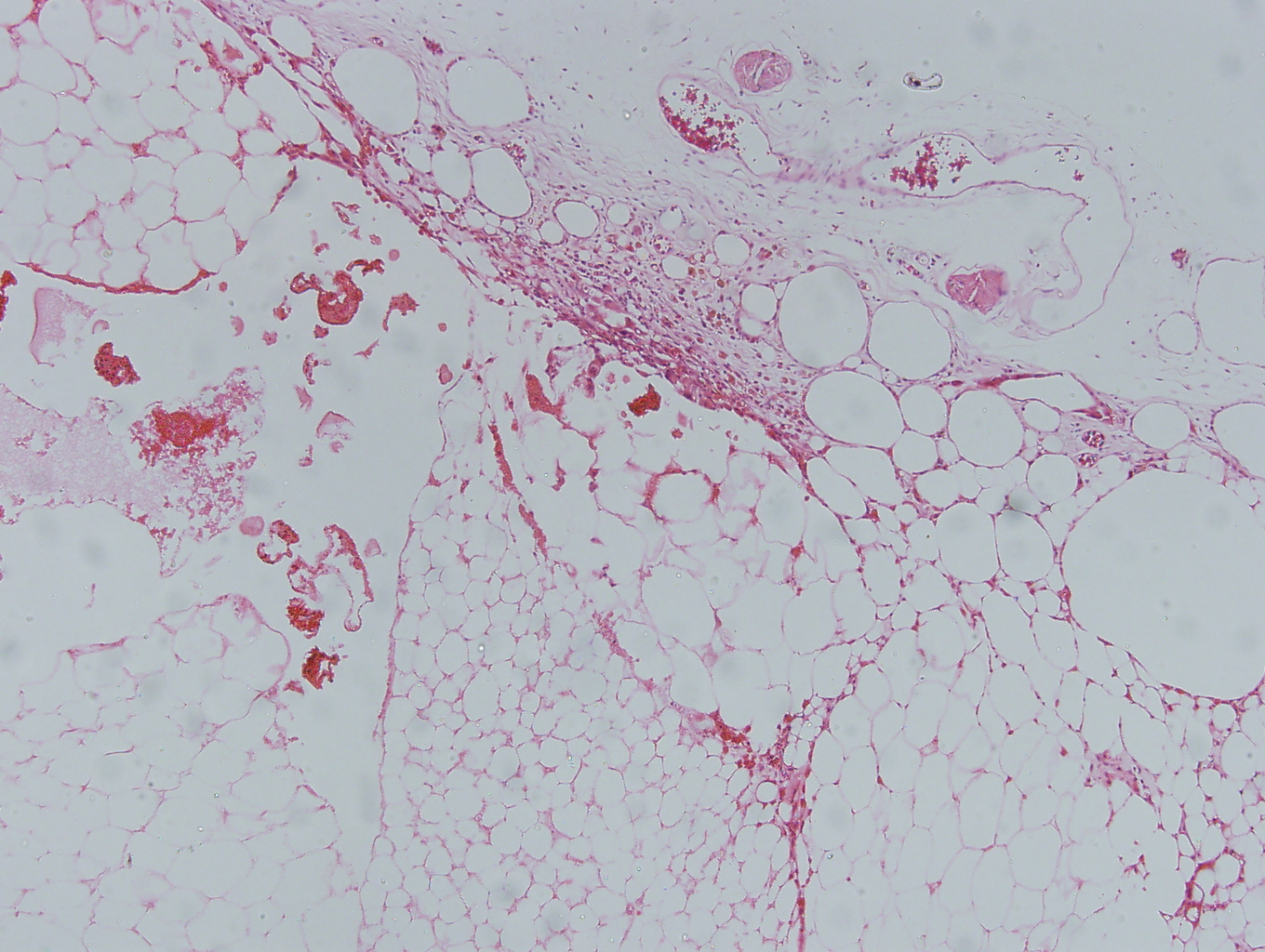

Supplement: Supplementary file 3 [file DataSheet6.ZIP › data for figure 3/HE in figure 3/ND-HC-HF 3M HE figure/HC-3M1-10X-7.jpg]

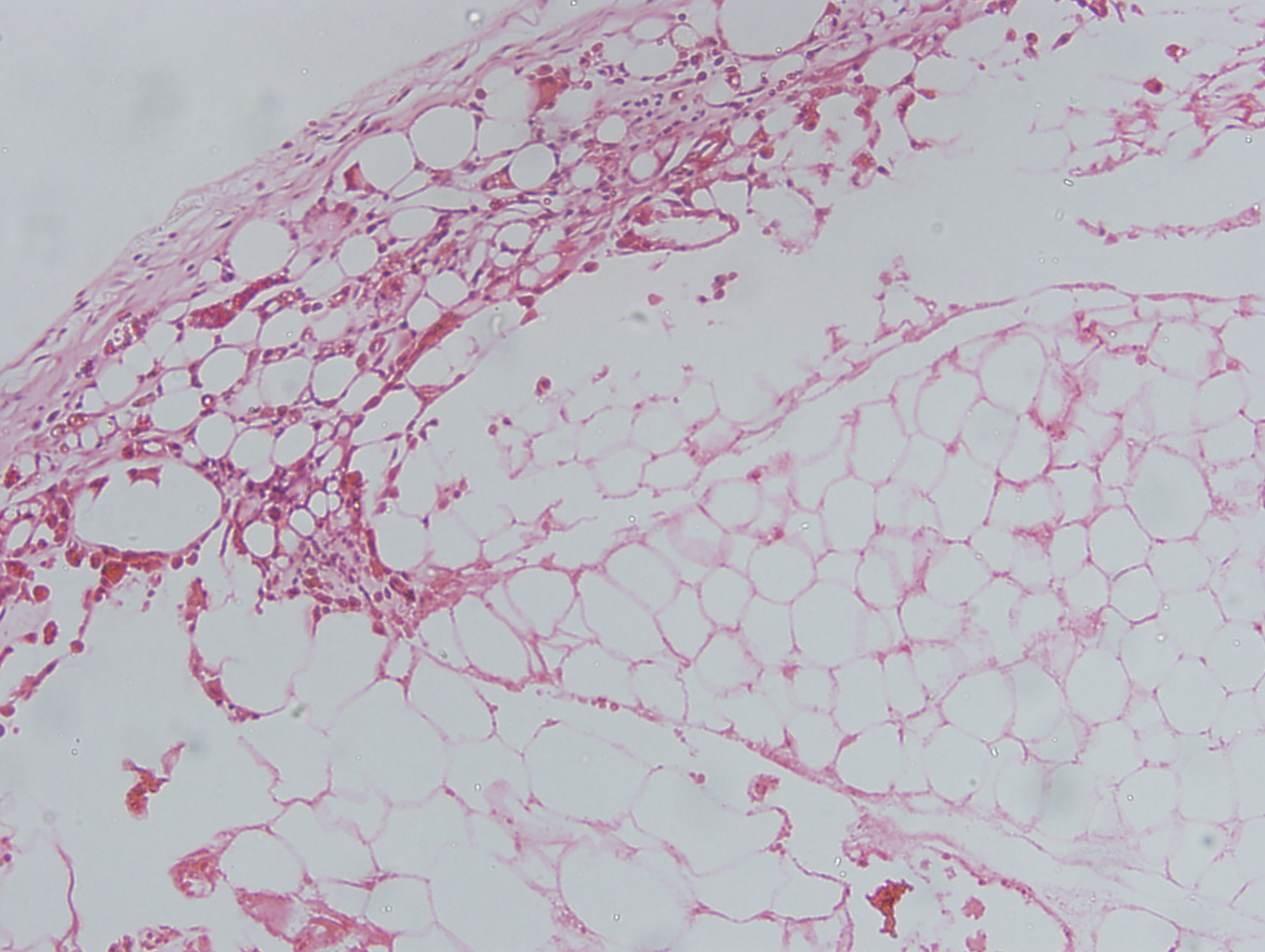

Supplement: Supplementary file 3 [file DataSheet6.ZIP › data for figure 3/HE in figure 3/ND-HC-HF 3M HE figure/HC-3M1-20X-12.jpg]

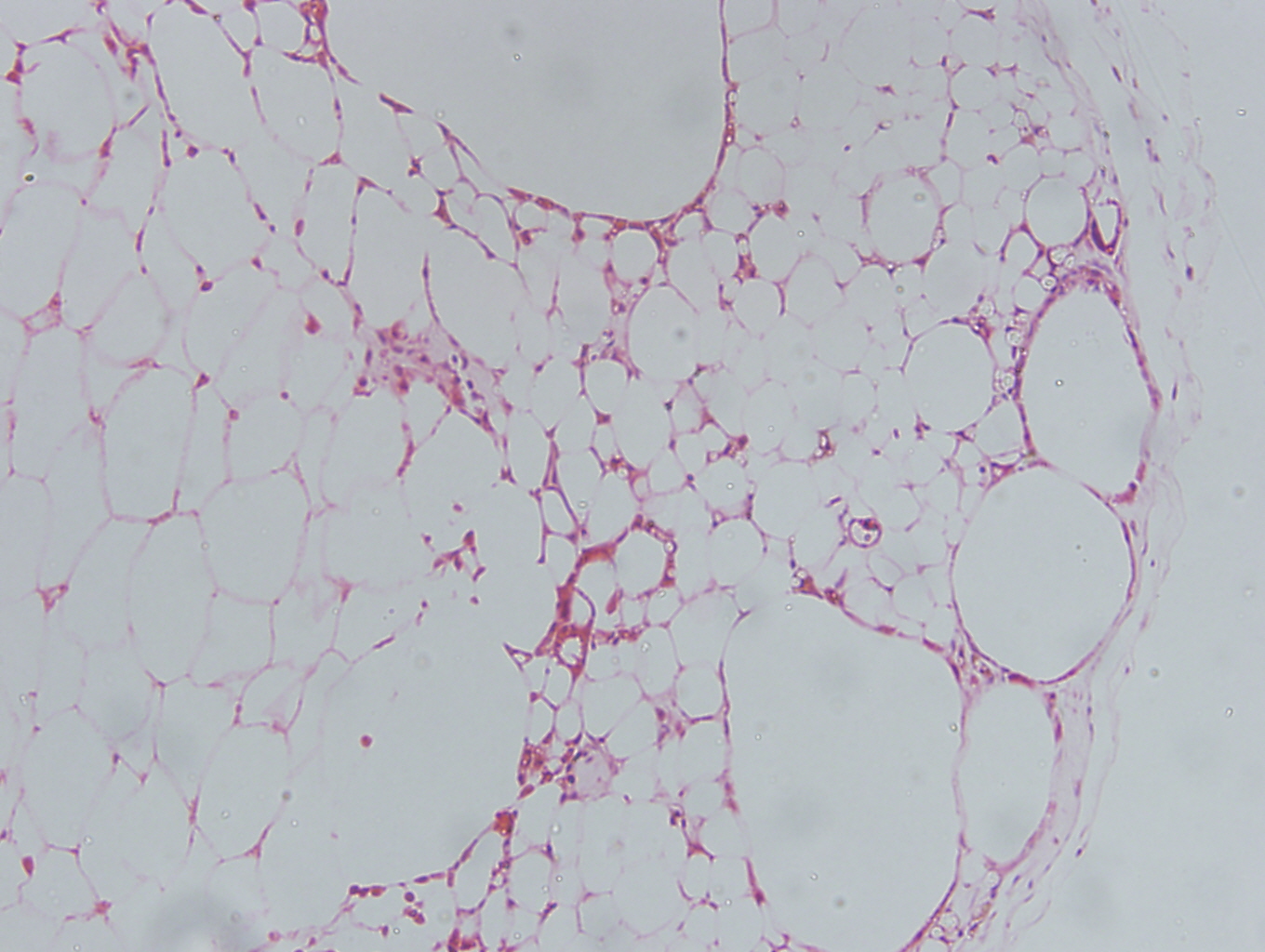

Supplement: Supplementary file 3 [file DataSheet6.ZIP › data for figure 3/HE in figure 3/ND-HC-HF 3M HE figure/HC-3M1-20X-8.jpg]

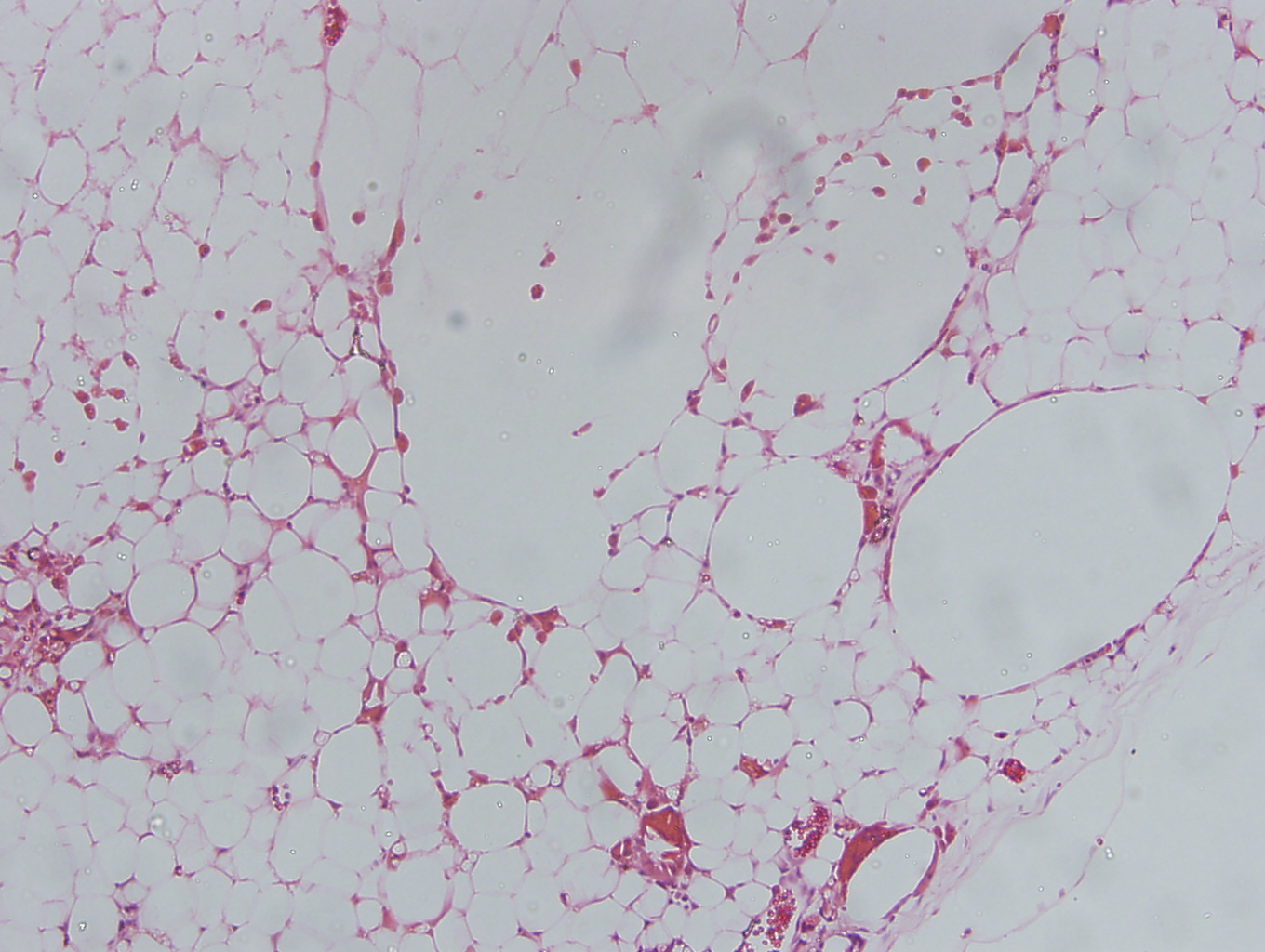

Supplement: Supplementary file 3 [file DataSheet6.ZIP › data for figure 3/HE in figure 3/ND-HC-HF 3M HE figure/HC-3M1-20X-9.jpg]

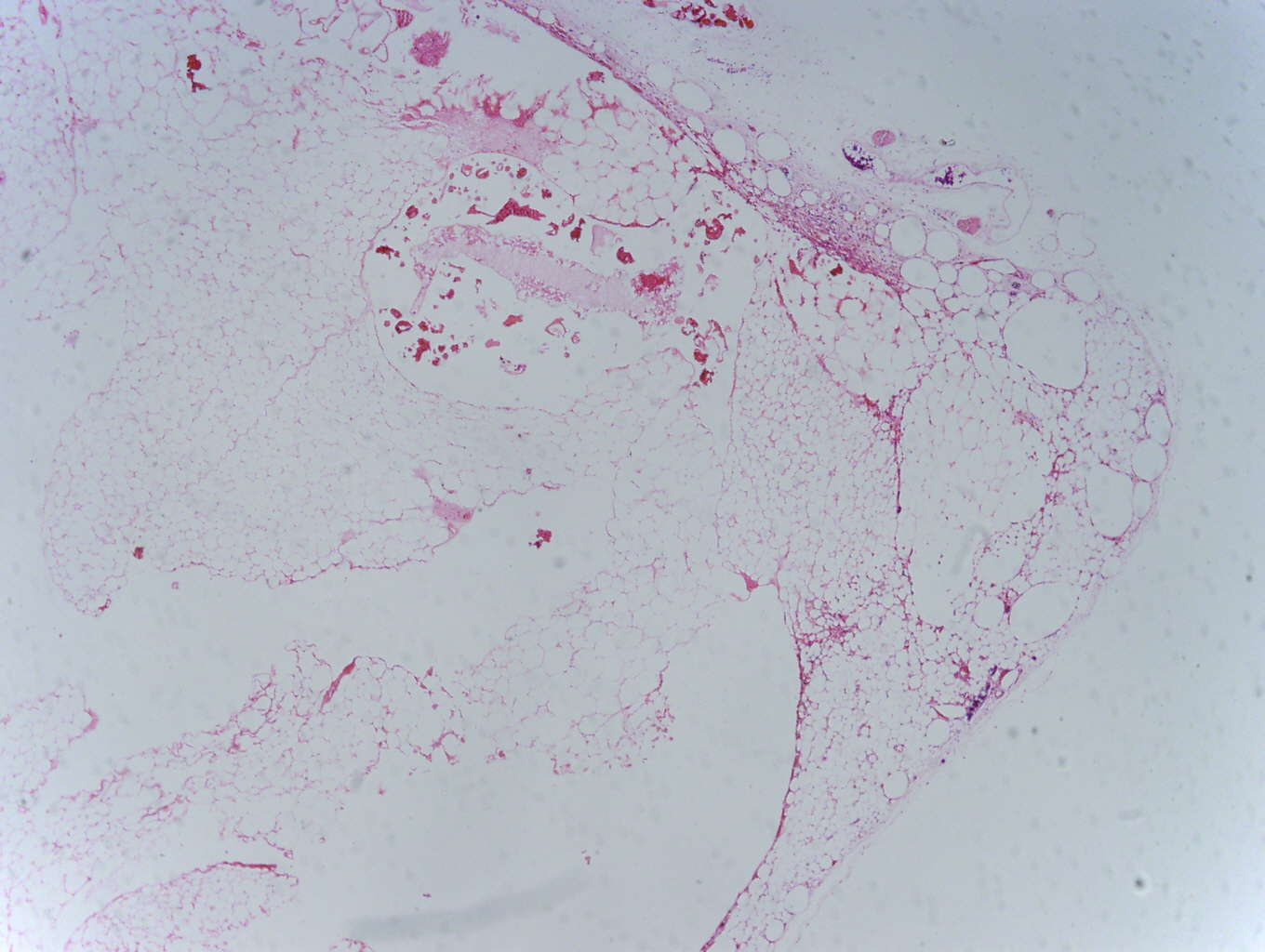

Supplement: Supplementary file 3 [file DataSheet6.ZIP › data for figure 3/HE in figure 3/ND-HC-HF 3M HE figure/HC-3M1-4X-2-2.jpg]

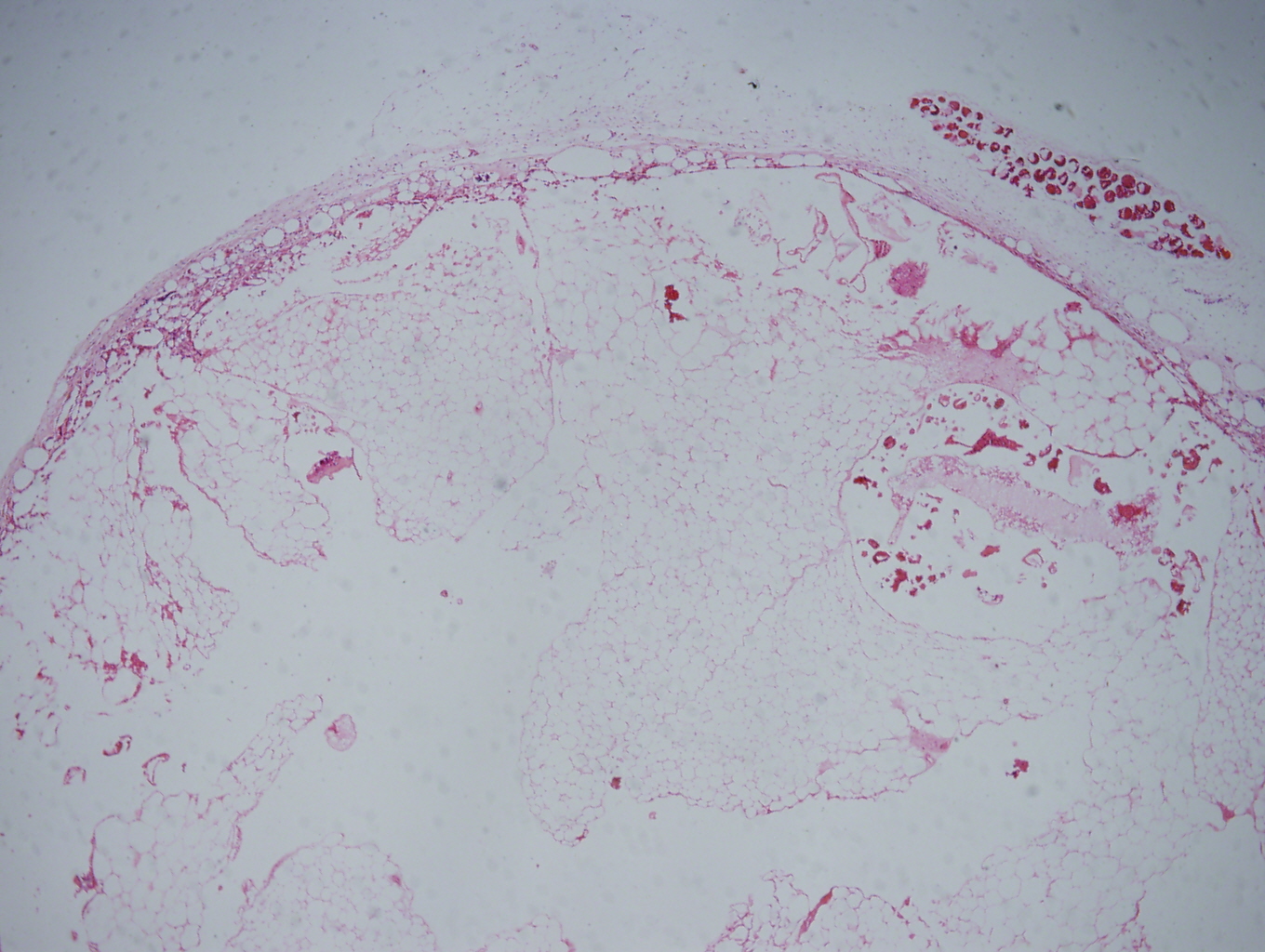

Supplement: Supplementary file 3 [file DataSheet6.ZIP › data for figure 3/HE in figure 3/ND-HC-HF 3M HE figure/HC-3M1-4X-3.jpg]

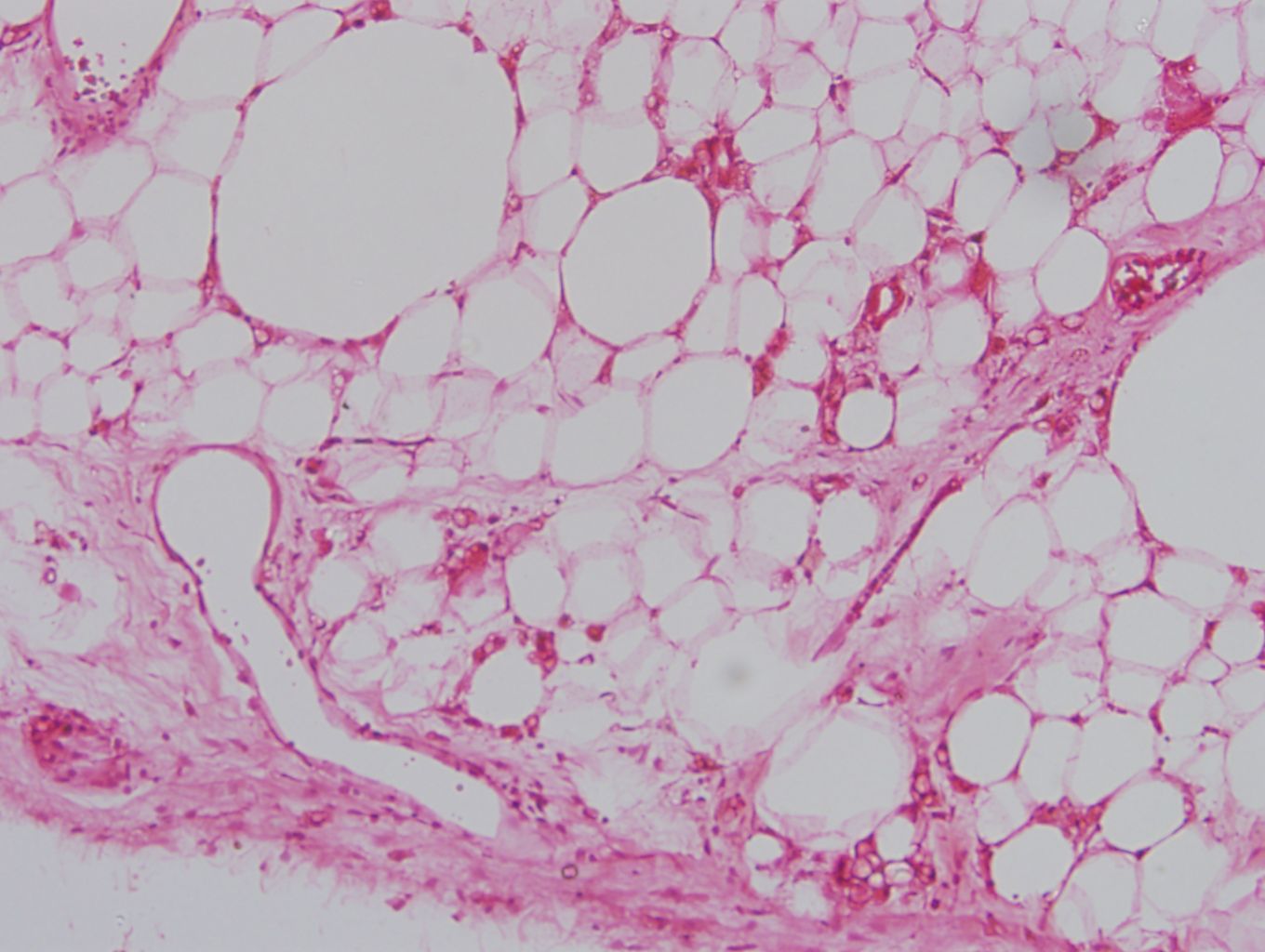

Supplement: Supplementary file 3 [file DataSheet6.ZIP › data for figure 3/HE in figure 3/ND-HC-HF 3M HE figure/HF-3M 20X-23.jpg]

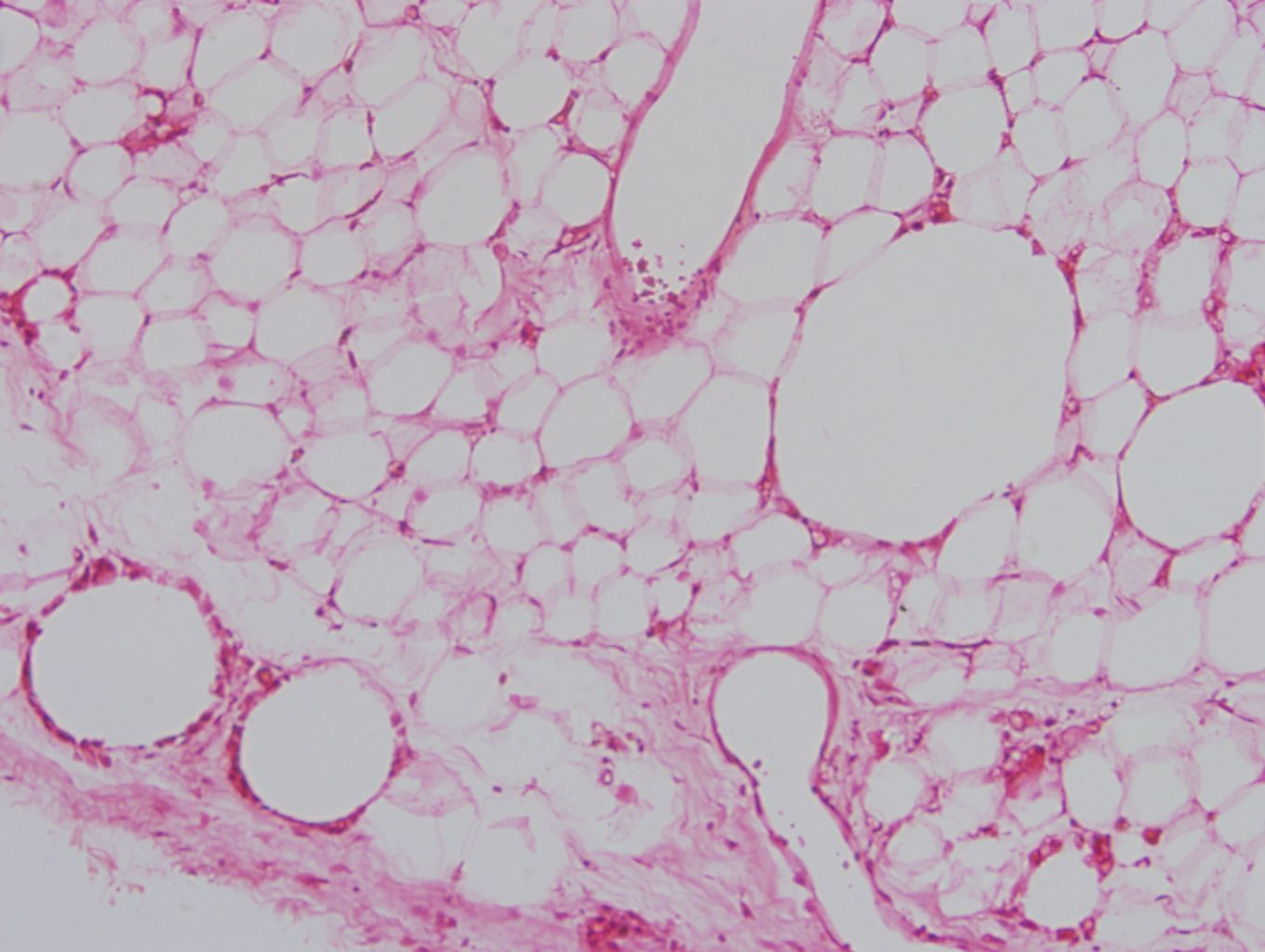

Supplement: Supplementary file 3 [file DataSheet6.ZIP › data for figure 3/HE in figure 3/ND-HC-HF 3M HE figure/HF-3M 20X-24.jpg]

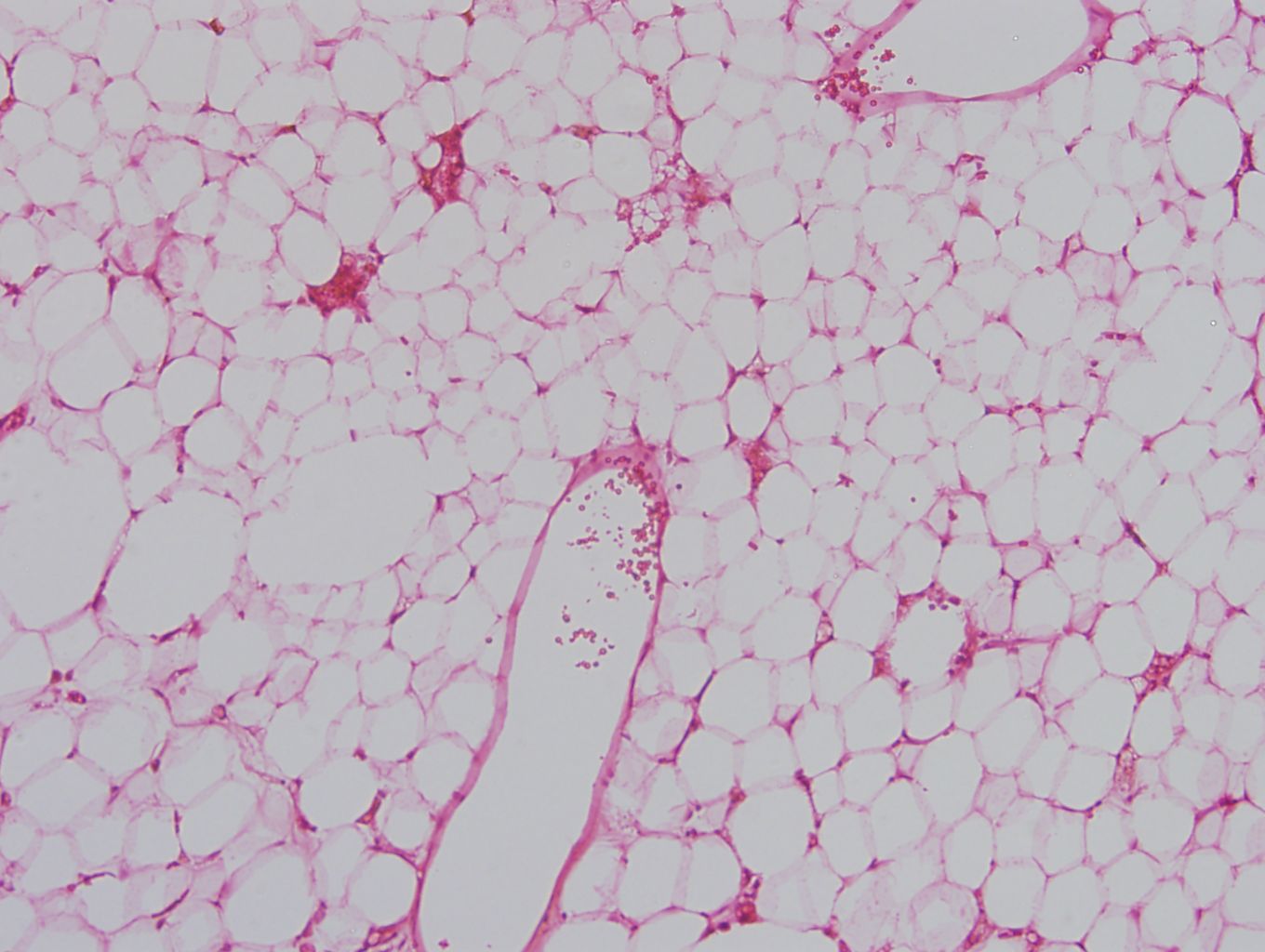

Supplement: Supplementary file 3 [file DataSheet6.ZIP › data for figure 3/HE in figure 3/ND-HC-HF 3M HE figure/HF-3M 20X-25.jpg]

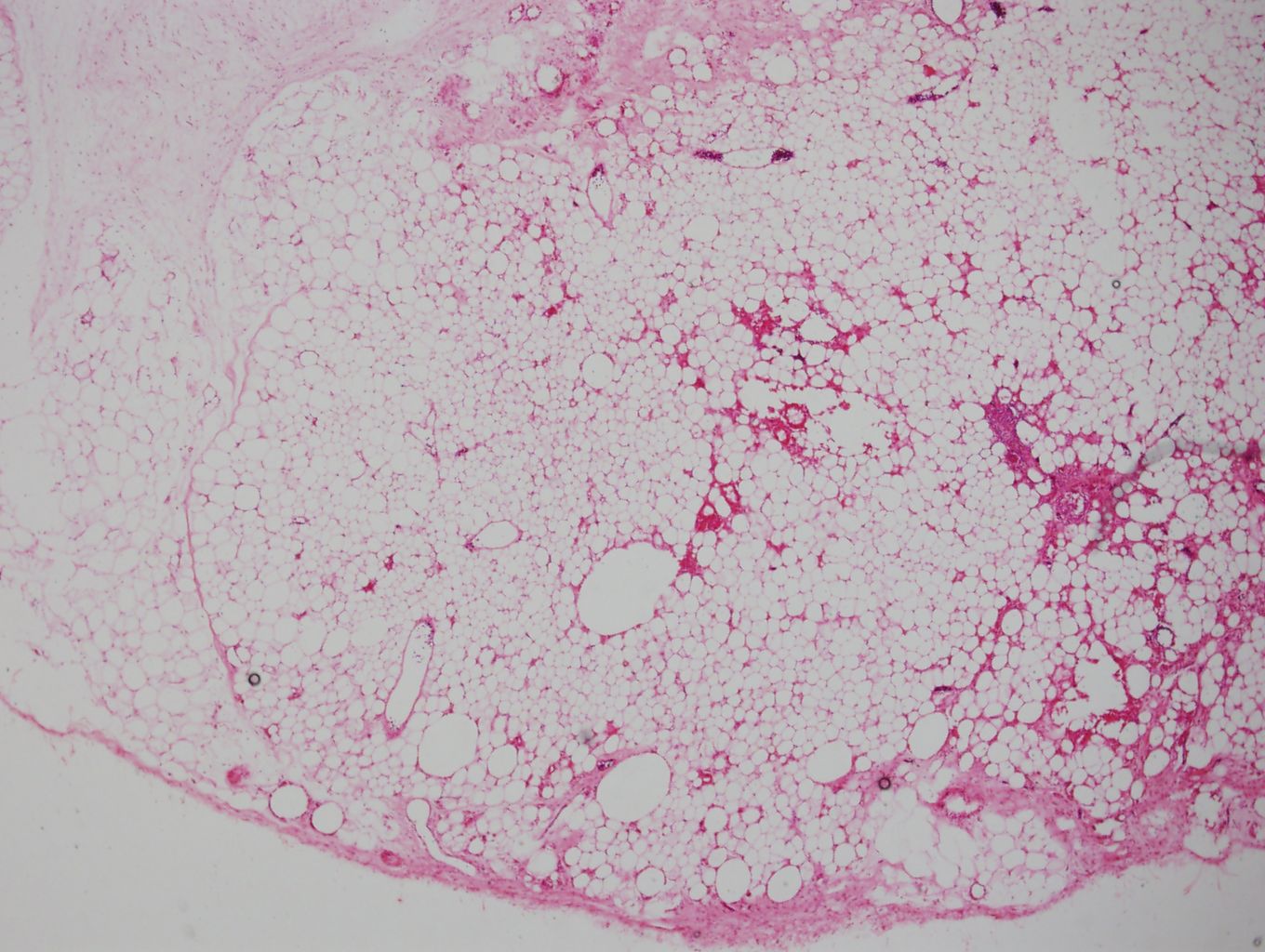

Supplement: Supplementary file 3 [file DataSheet6.ZIP › data for figure 3/HE in figure 3/ND-HC-HF 3M HE figure/HF-3M 4X-01.jpg]

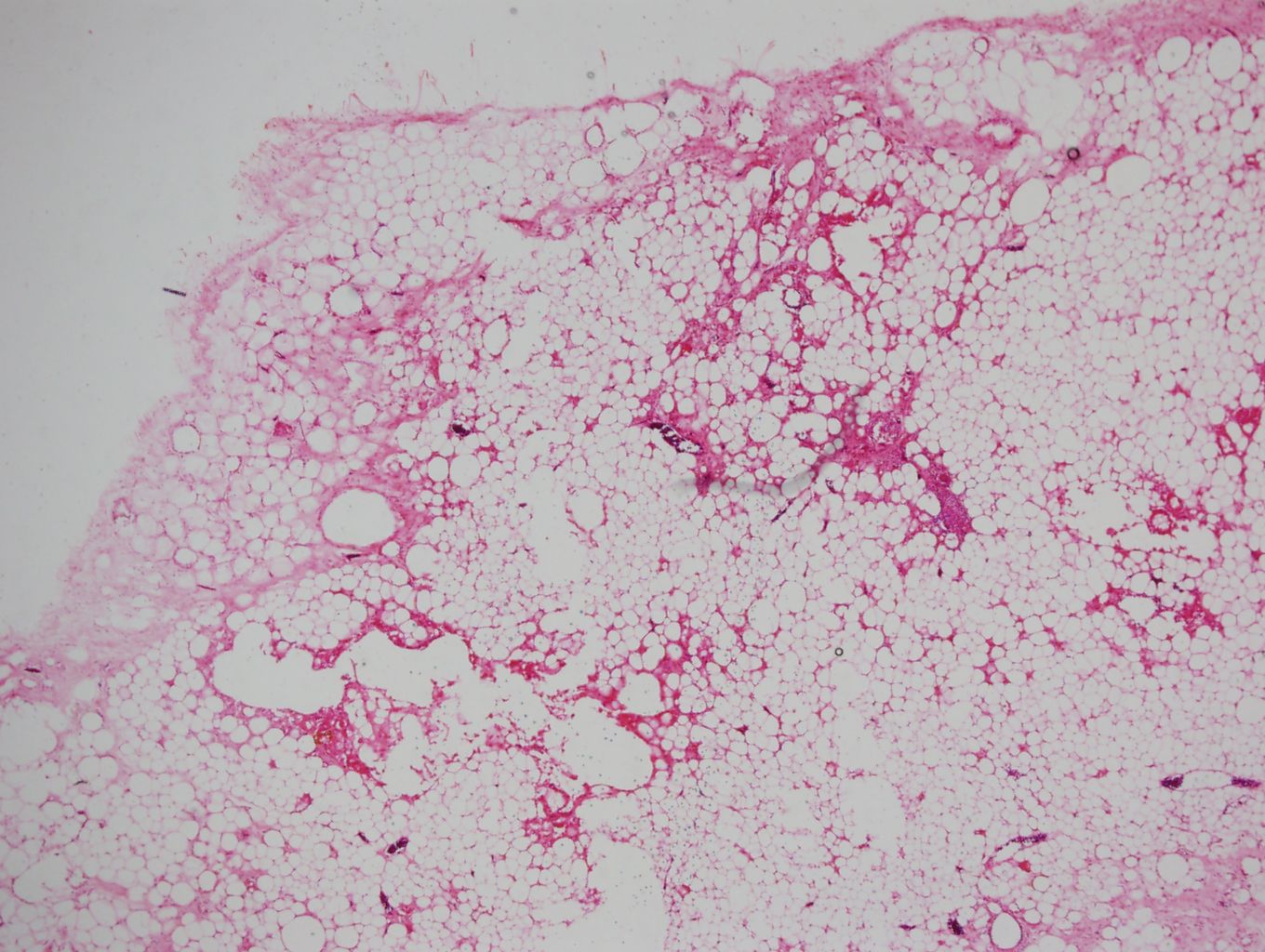

Supplement: Supplementary file 3 [file DataSheet6.ZIP › data for figure 3/HE in figure 3/ND-HC-HF 3M HE figure/HF-3M 4X-02.jpg]

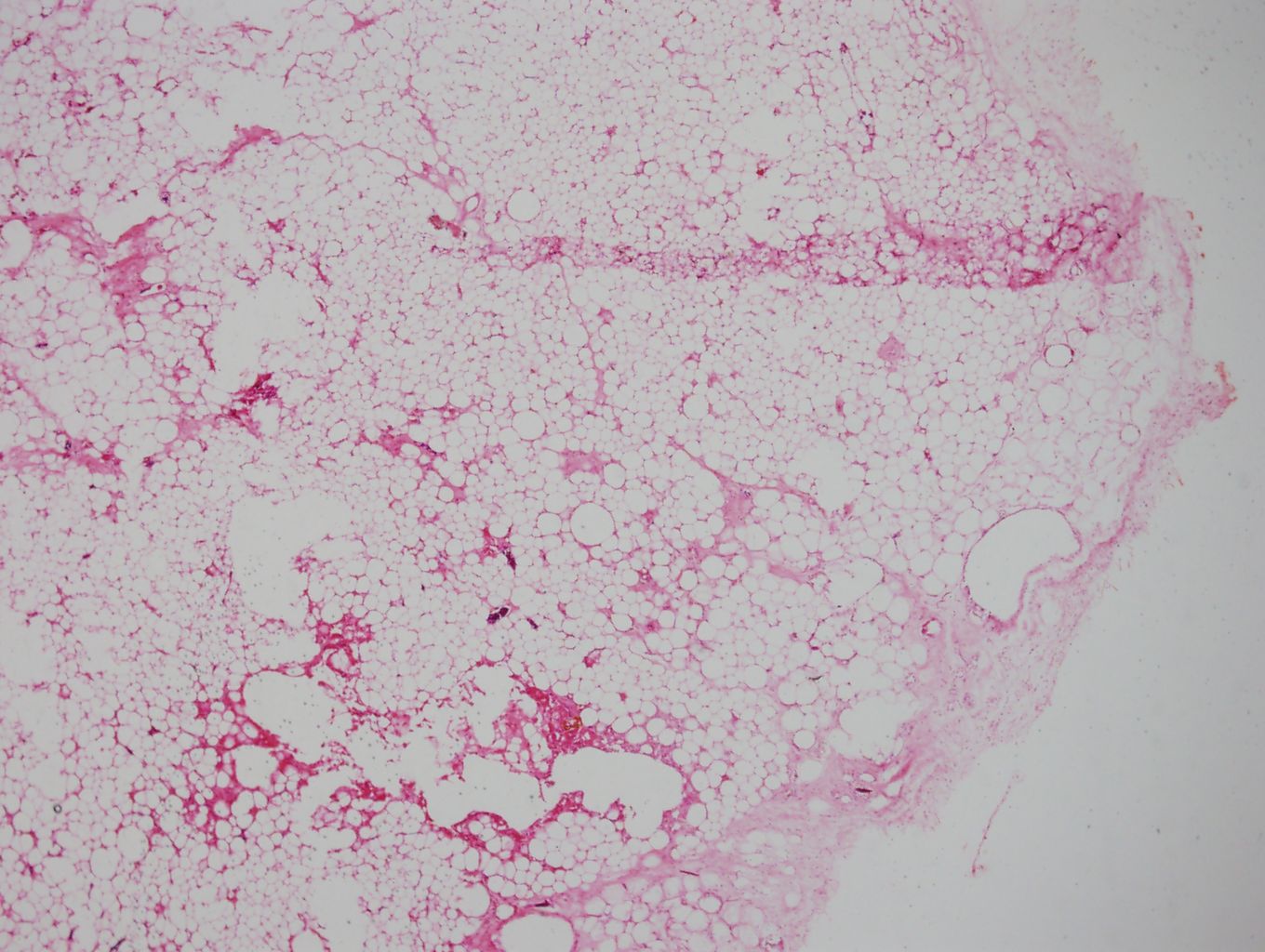

Supplement: Supplementary file 3 [file DataSheet6.ZIP › data for figure 3/HE in figure 3/ND-HC-HF 3M HE figure/HF-3M 4X-03.jpg]

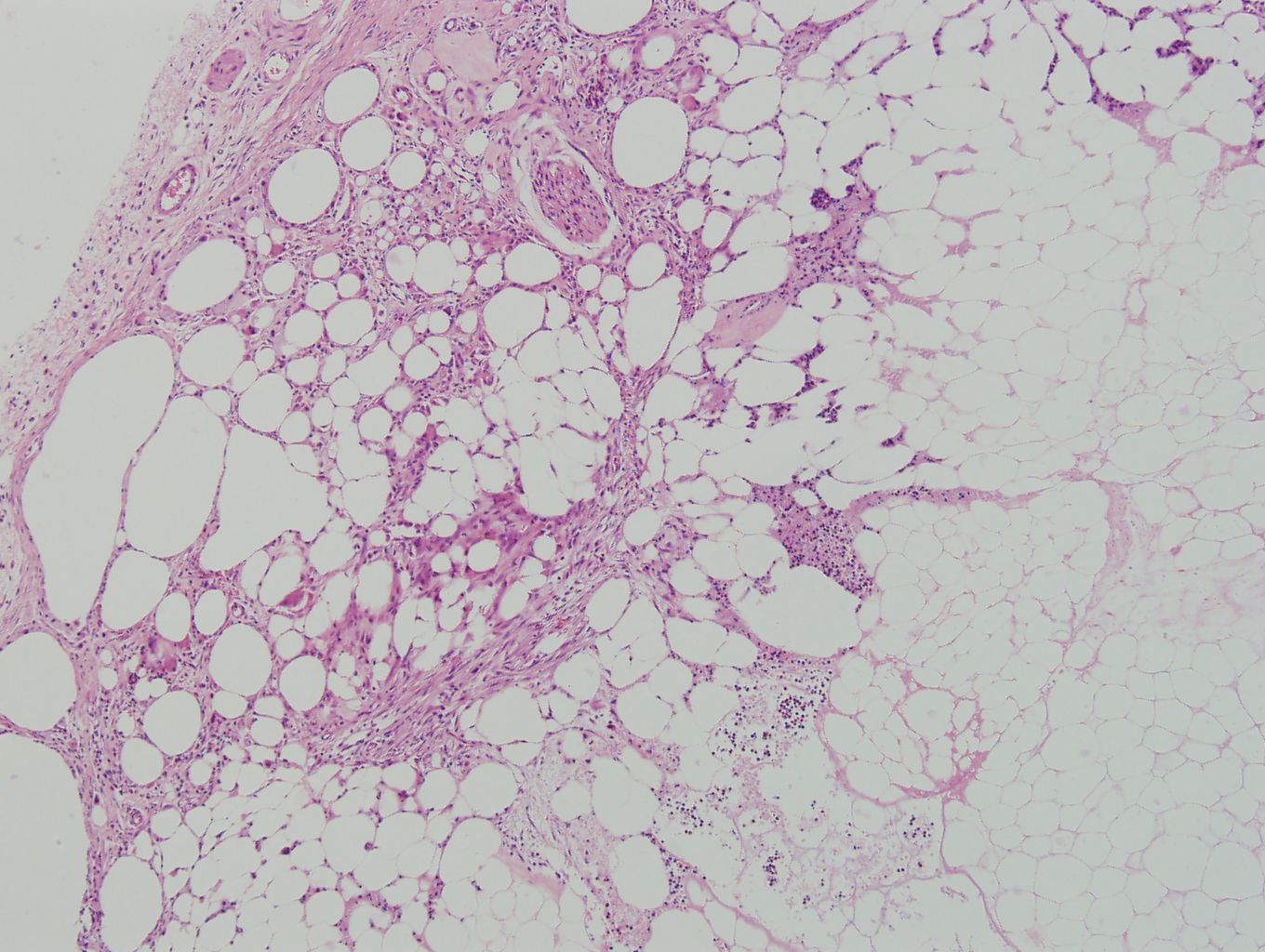

Supplement: Supplementary file 3 [file DataSheet6.ZIP › data for figure 3/HE in figure 3/ND-HC-HF 3M HE figure/ND-3M1-10X--04.jpg]

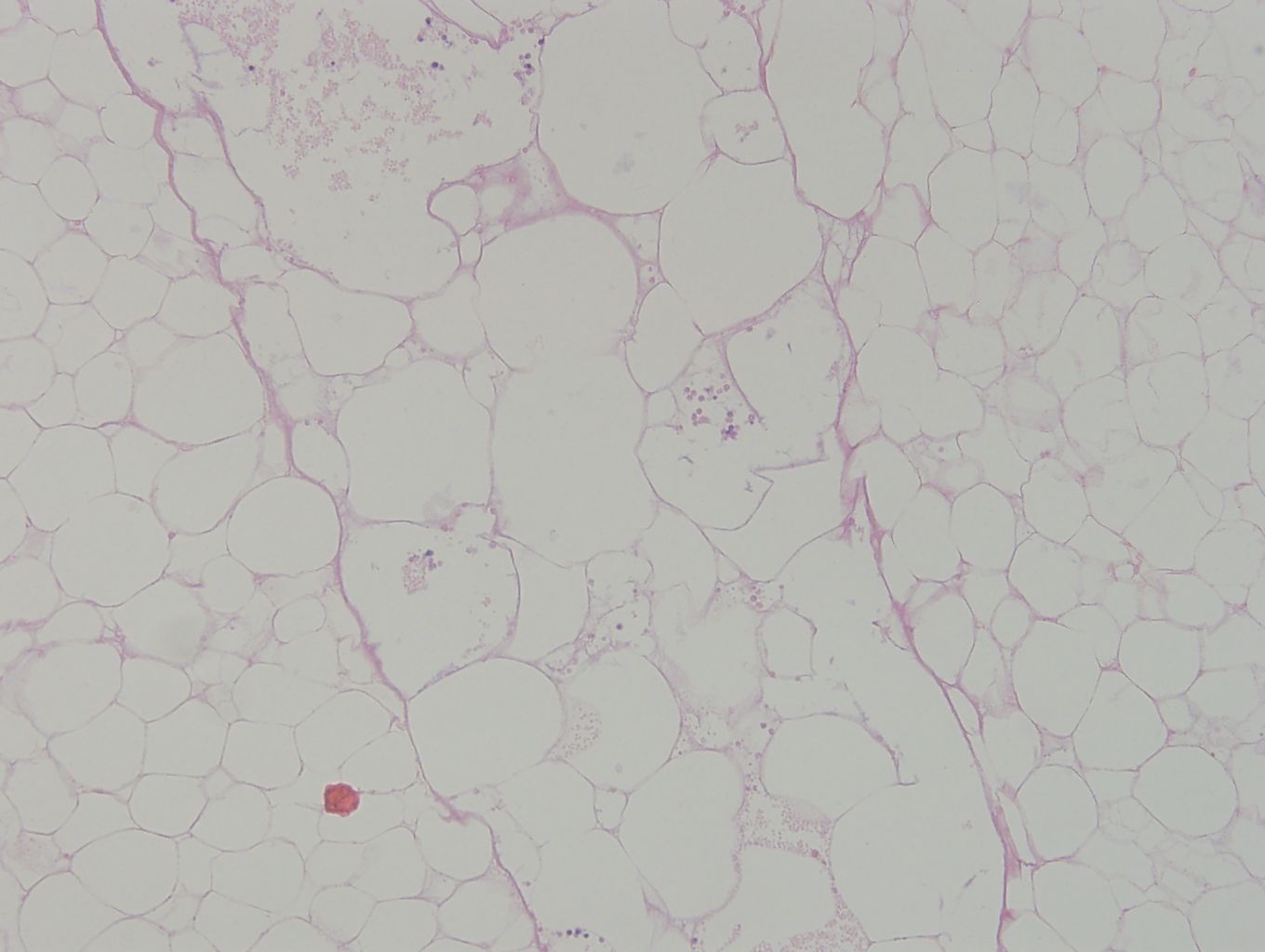

Supplement: Supplementary file 3 [file DataSheet6.ZIP › data for figure 3/HE in figure 3/ND-HC-HF 3M HE figure/ND-3M1-20X--15.jpg]

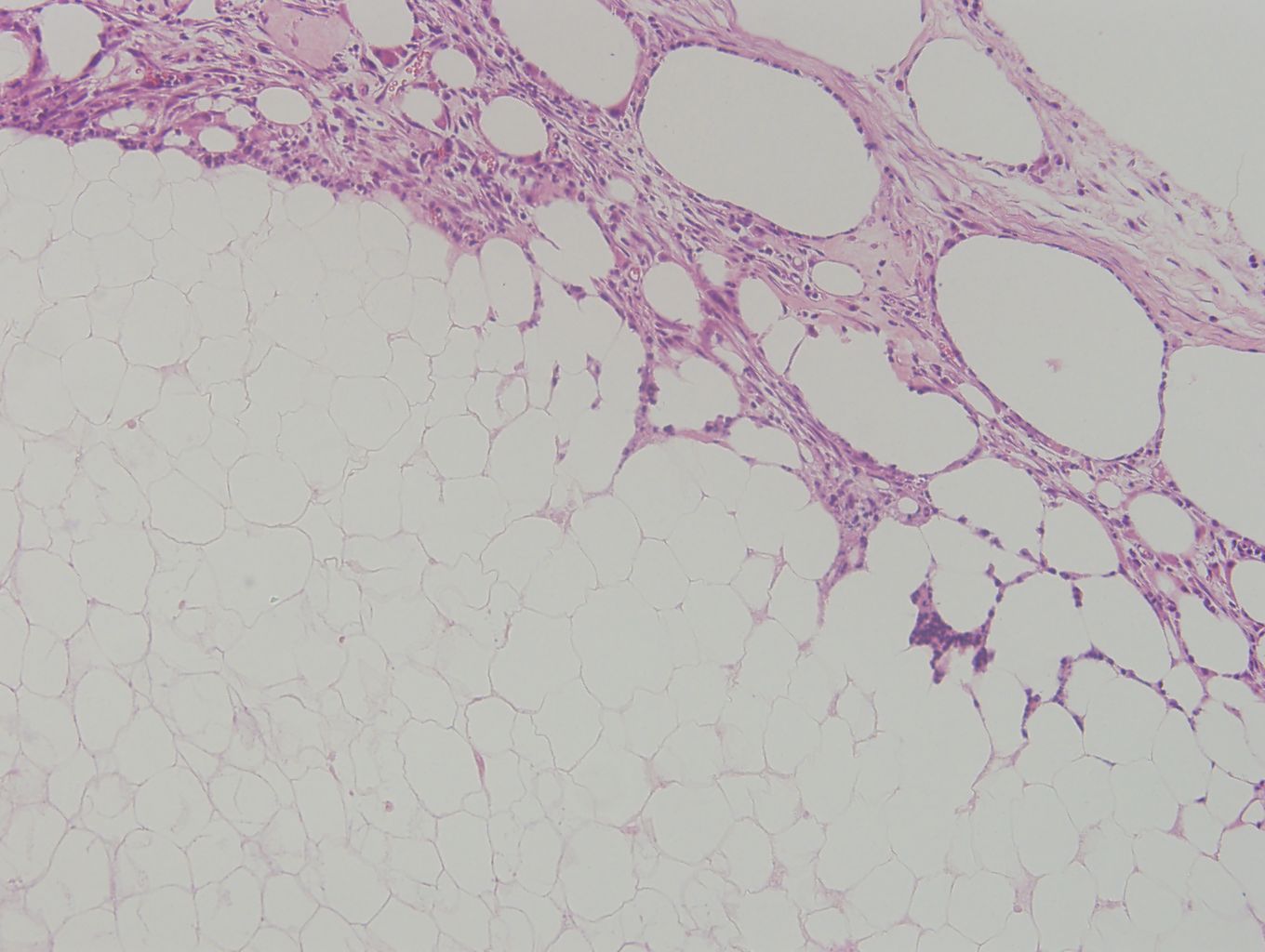

Supplement: Supplementary file 3 [file DataSheet6.ZIP › data for figure 3/HE in figure 3/ND-HC-HF 3M HE figure/ND-3M1-20X--16.jpg]

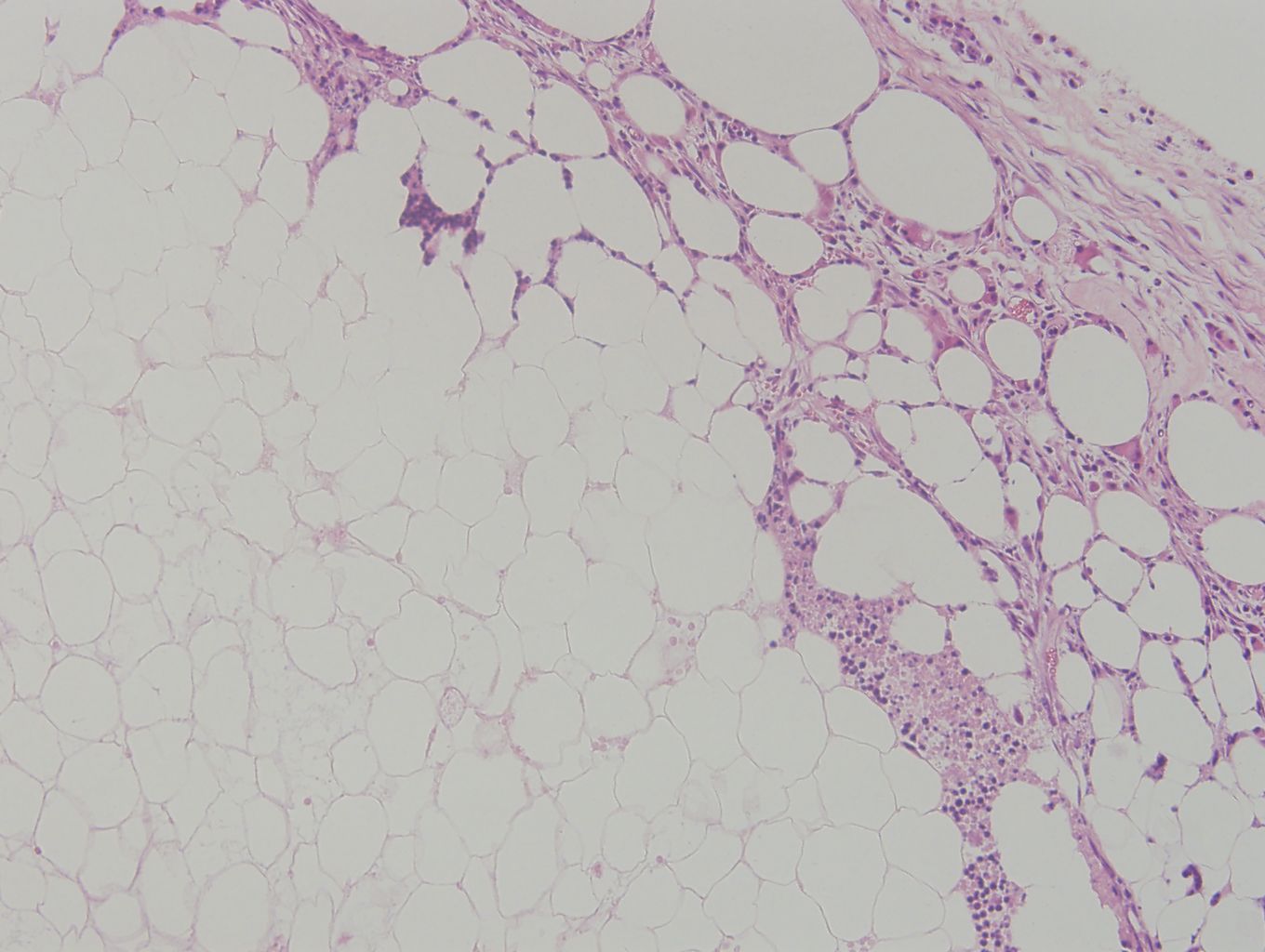

Supplement: Supplementary file 3 [file DataSheet6.ZIP › data for figure 3/HE in figure 3/ND-HC-HF 3M HE figure/ND-3M1-20X--17.jpg]

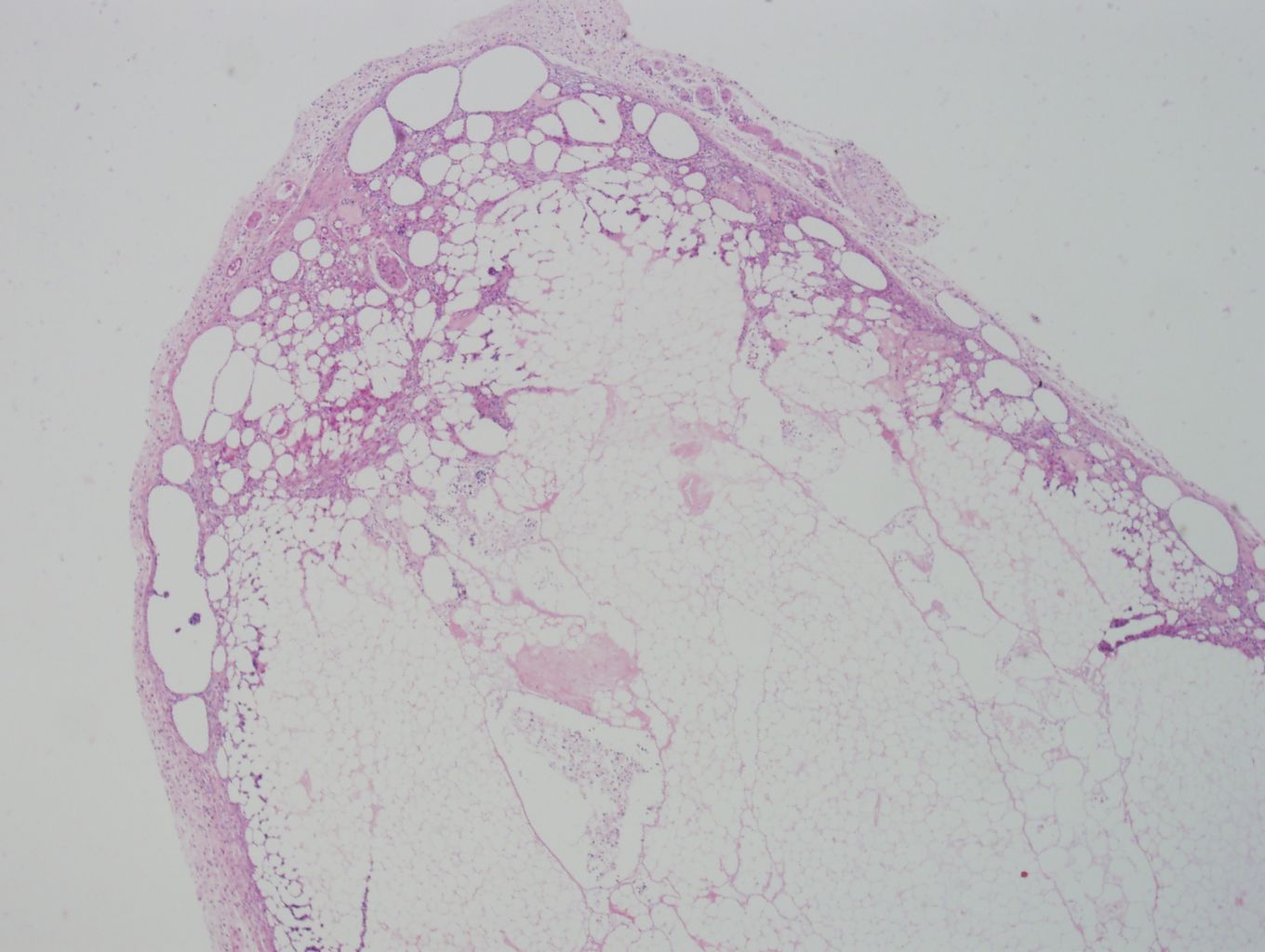

Supplement: Supplementary file 3 [file DataSheet6.ZIP › data for figure 3/HE in figure 3/ND-HC-HF 3M HE figure/ND-3M1-4X--01.jpg]

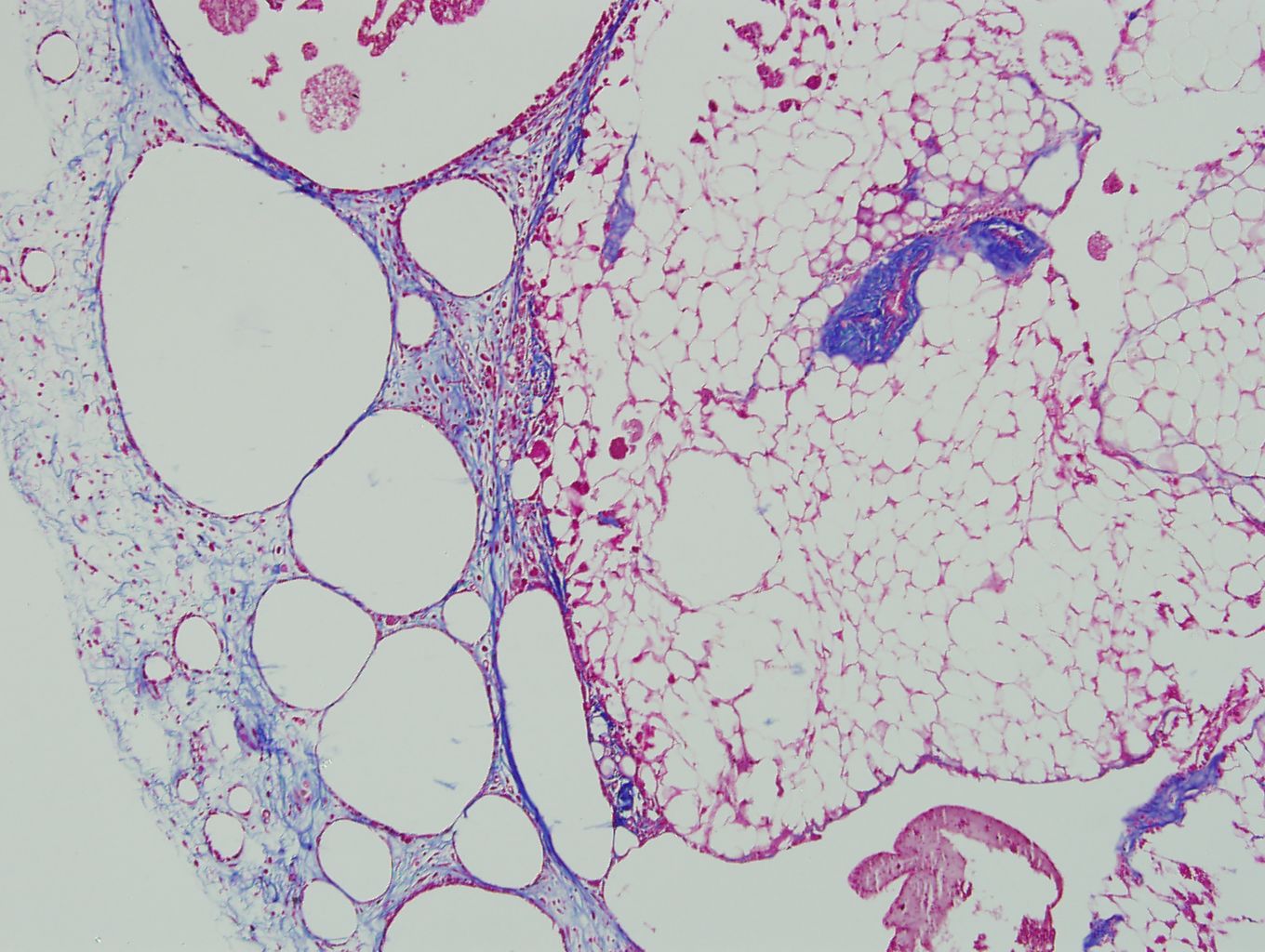

Supplement: Supplementary file 3 [file DataSheet6.ZIP › data for figure 3/masson staining of ND-HC-HF 3M/HC-3M MASSON 10X-04.jpg]

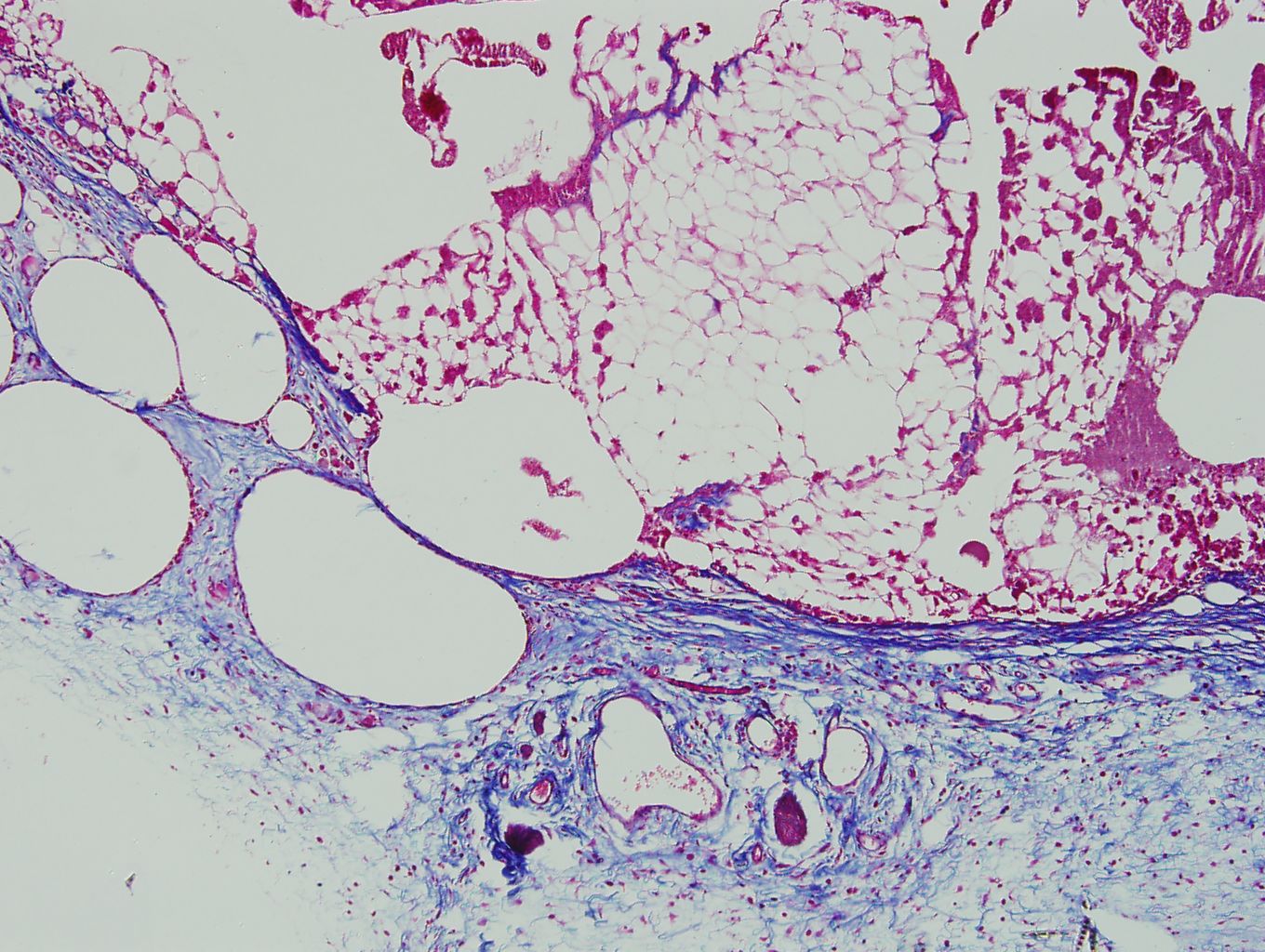

Supplement: Supplementary file 3 [file DataSheet6.ZIP › data for figure 3/masson staining of ND-HC-HF 3M/HC-3M MASSON 10X-05.jpg]

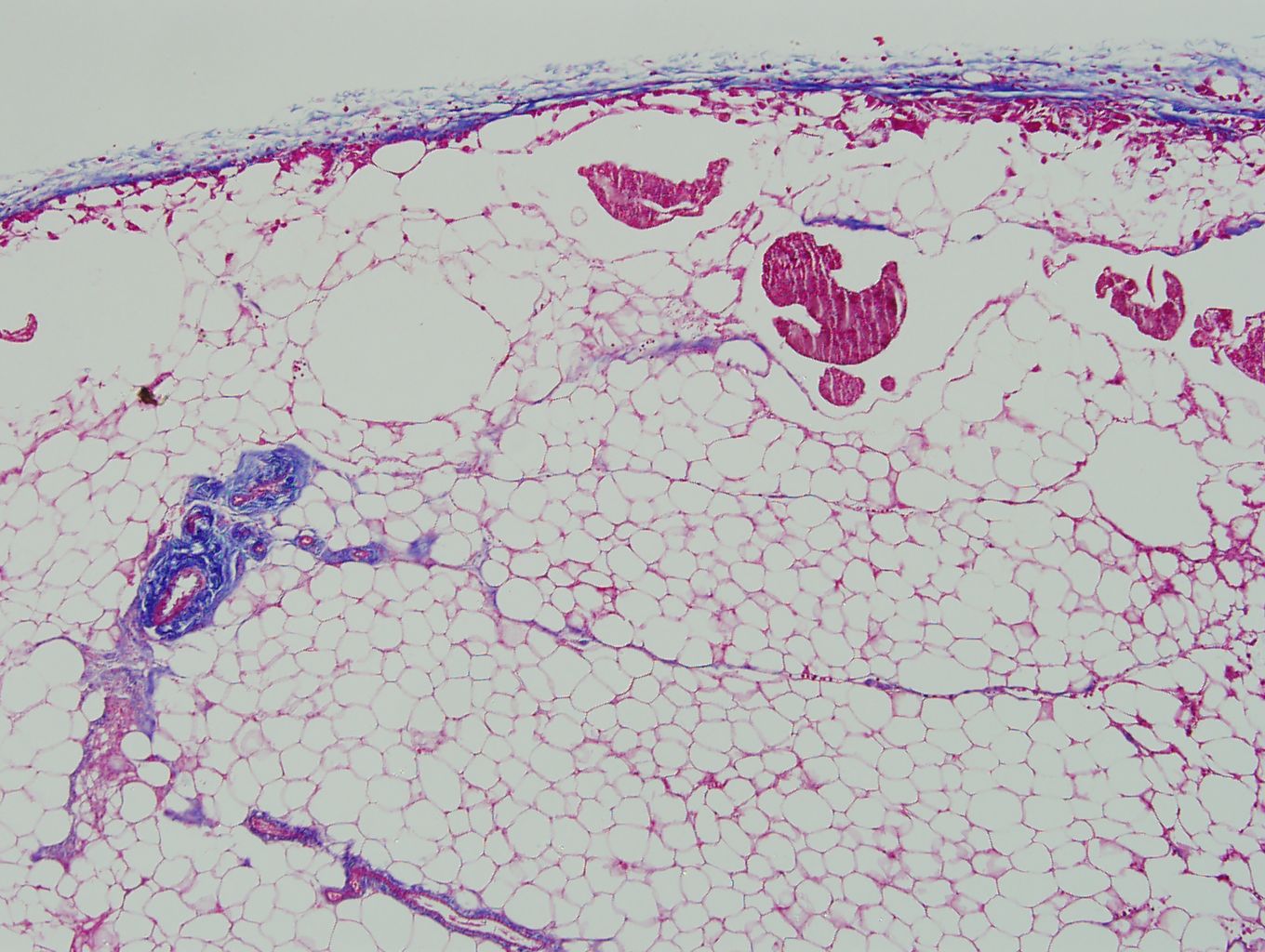

Supplement: Supplementary file 3 [file DataSheet6.ZIP › data for figure 3/masson staining of ND-HC-HF 3M/HC-3M MASSON 10X-08.jpg]

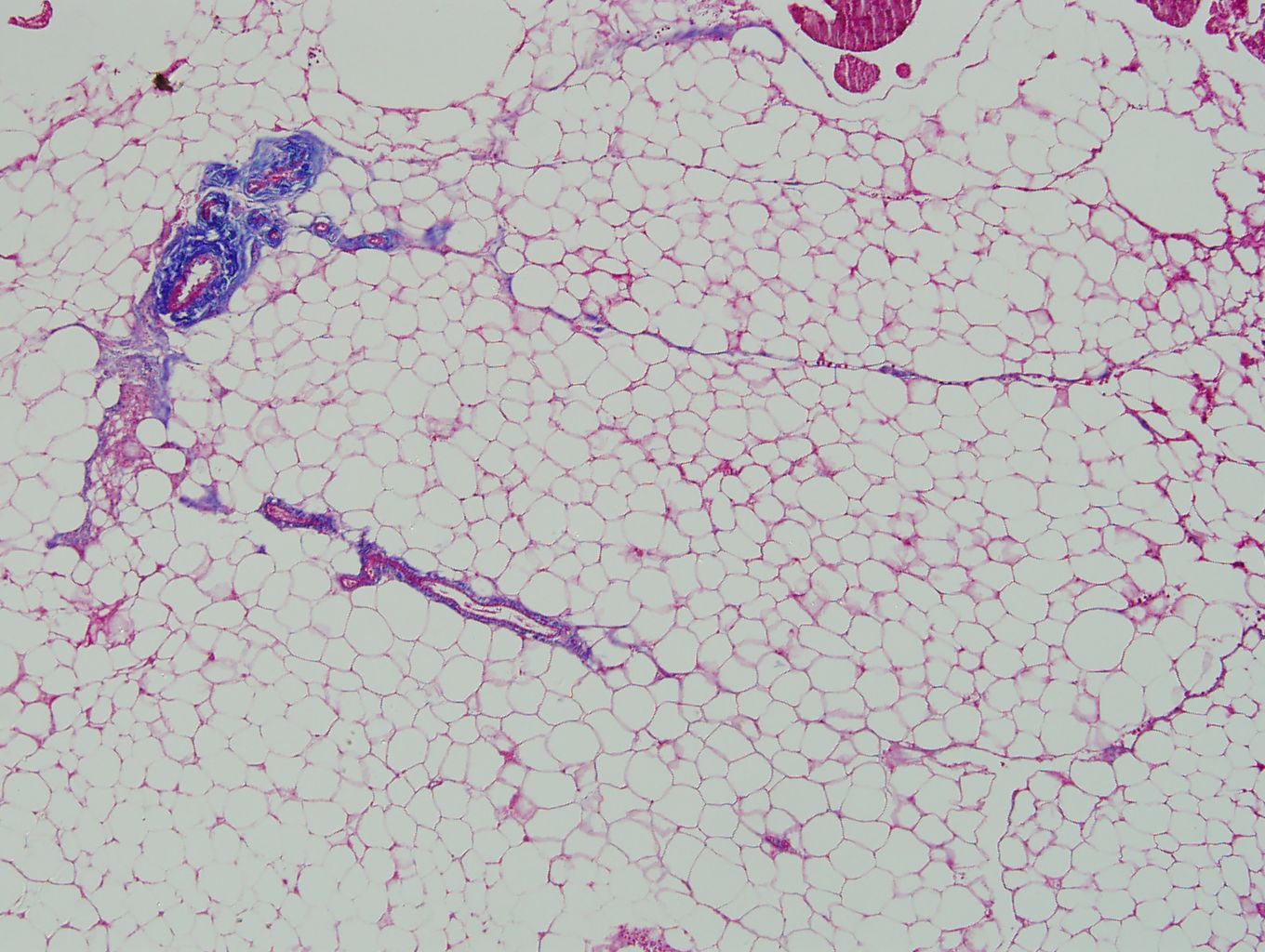

Supplement: Supplementary file 3 [file DataSheet6.ZIP › data for figure 3/masson staining of ND-HC-HF 3M/HC-3M MASSON 10X-09.jpg]

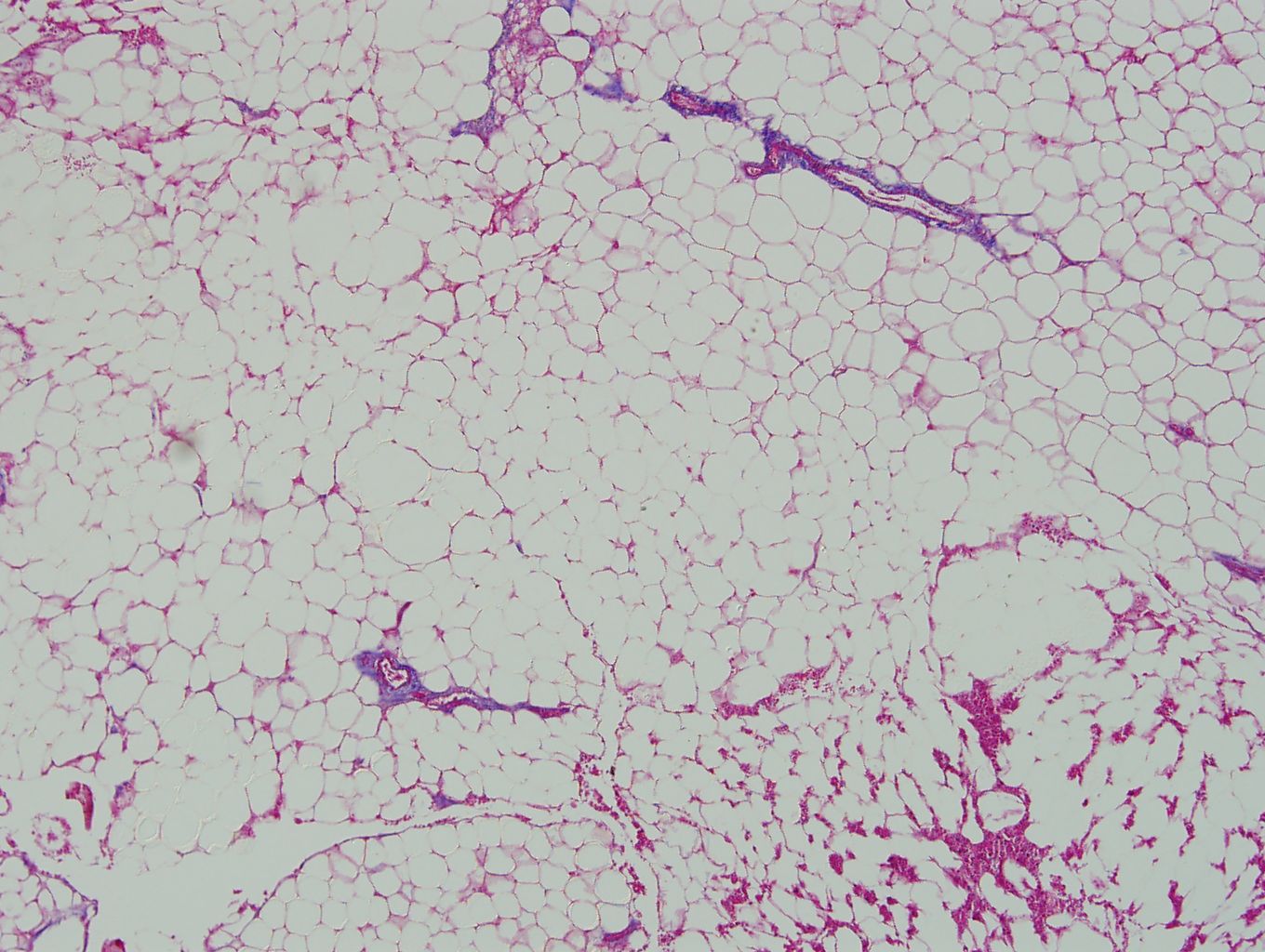

Supplement: Supplementary file 3 [file DataSheet6.ZIP › data for figure 3/masson staining of ND-HC-HF 3M/HC-3M MASSON 10X-10.jpg]

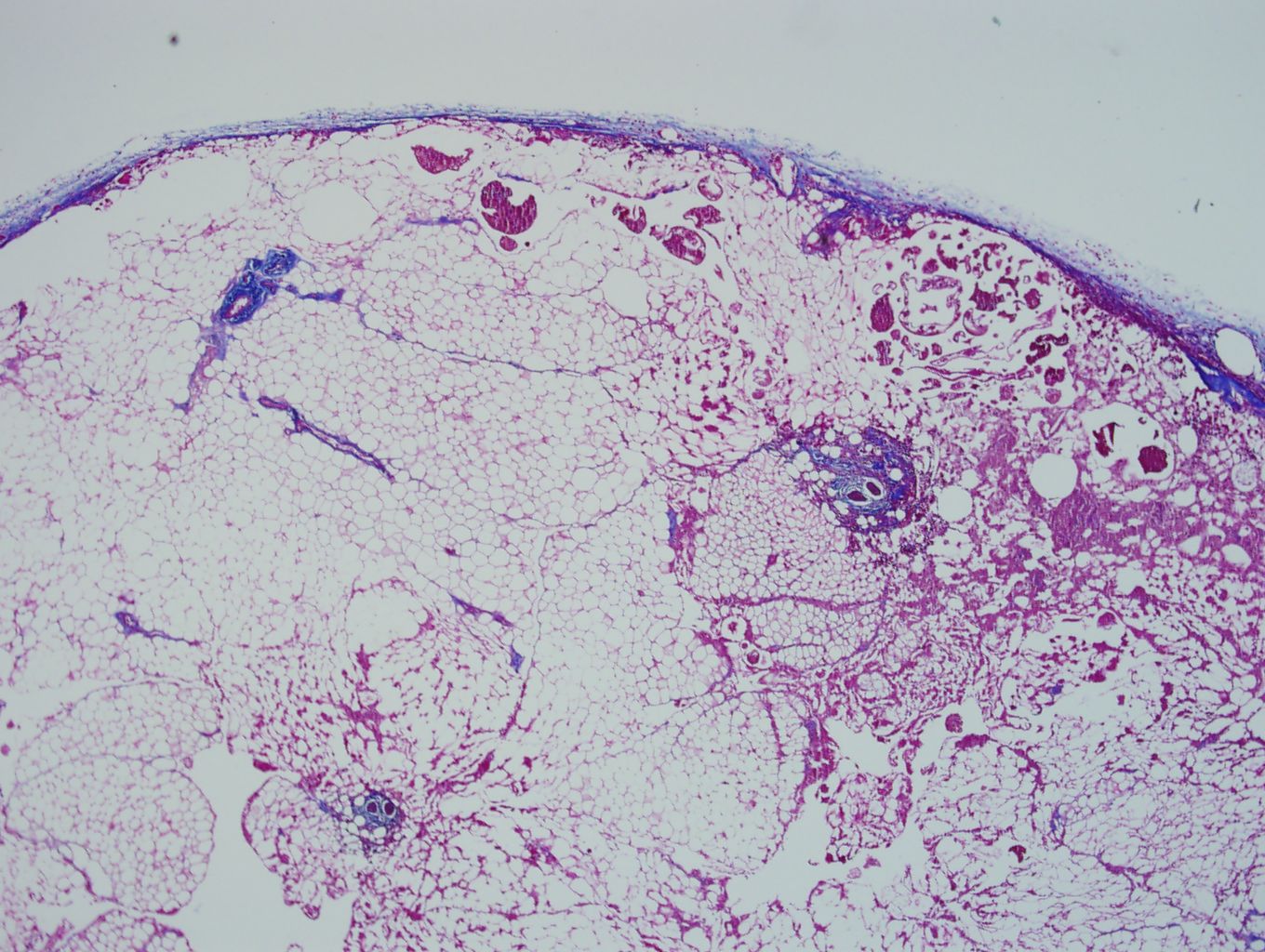

Supplement: Supplementary file 3 [file DataSheet6.ZIP › data for figure 3/masson staining of ND-HC-HF 3M/HC-3M MASSON 4X-03.jpg]

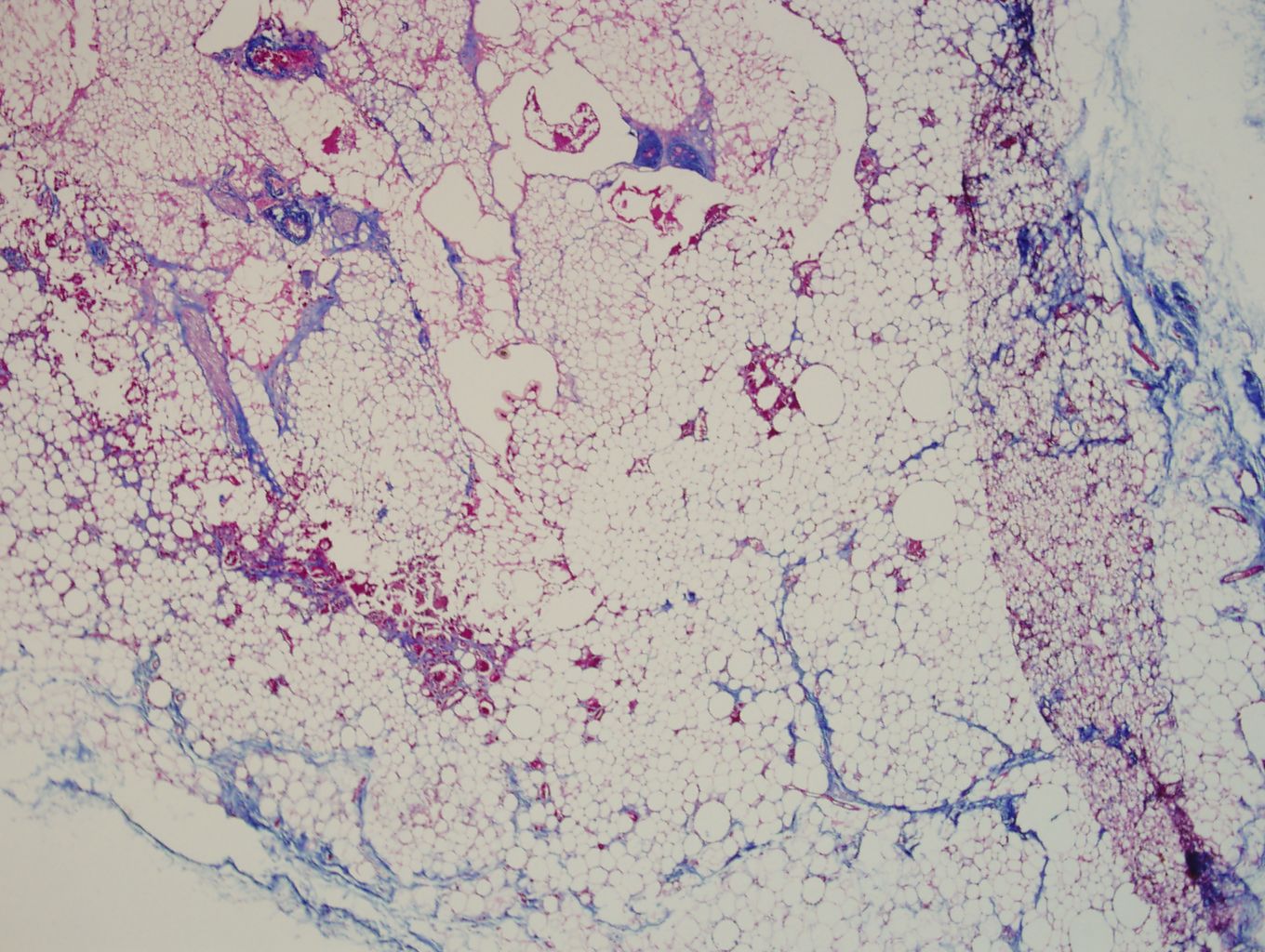

Supplement: Supplementary file 3 [file DataSheet6.ZIP › data for figure 3/masson staining of ND-HC-HF 3M/HF-3M MASSON-28.jpg]

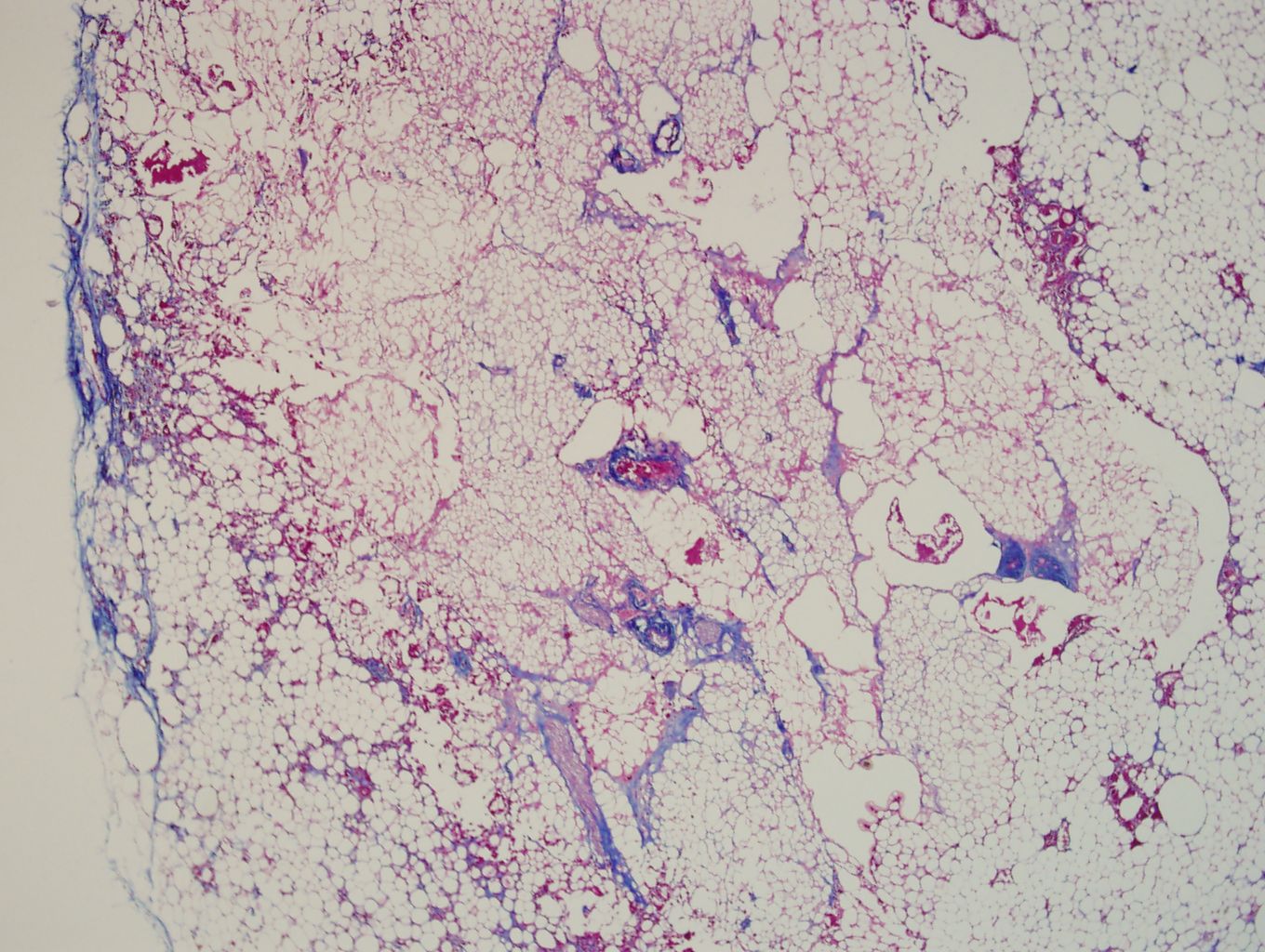

Supplement: Supplementary file 3 [file DataSheet6.ZIP › data for figure 3/masson staining of ND-HC-HF 3M/HF-3M MASSON-29.jpg]

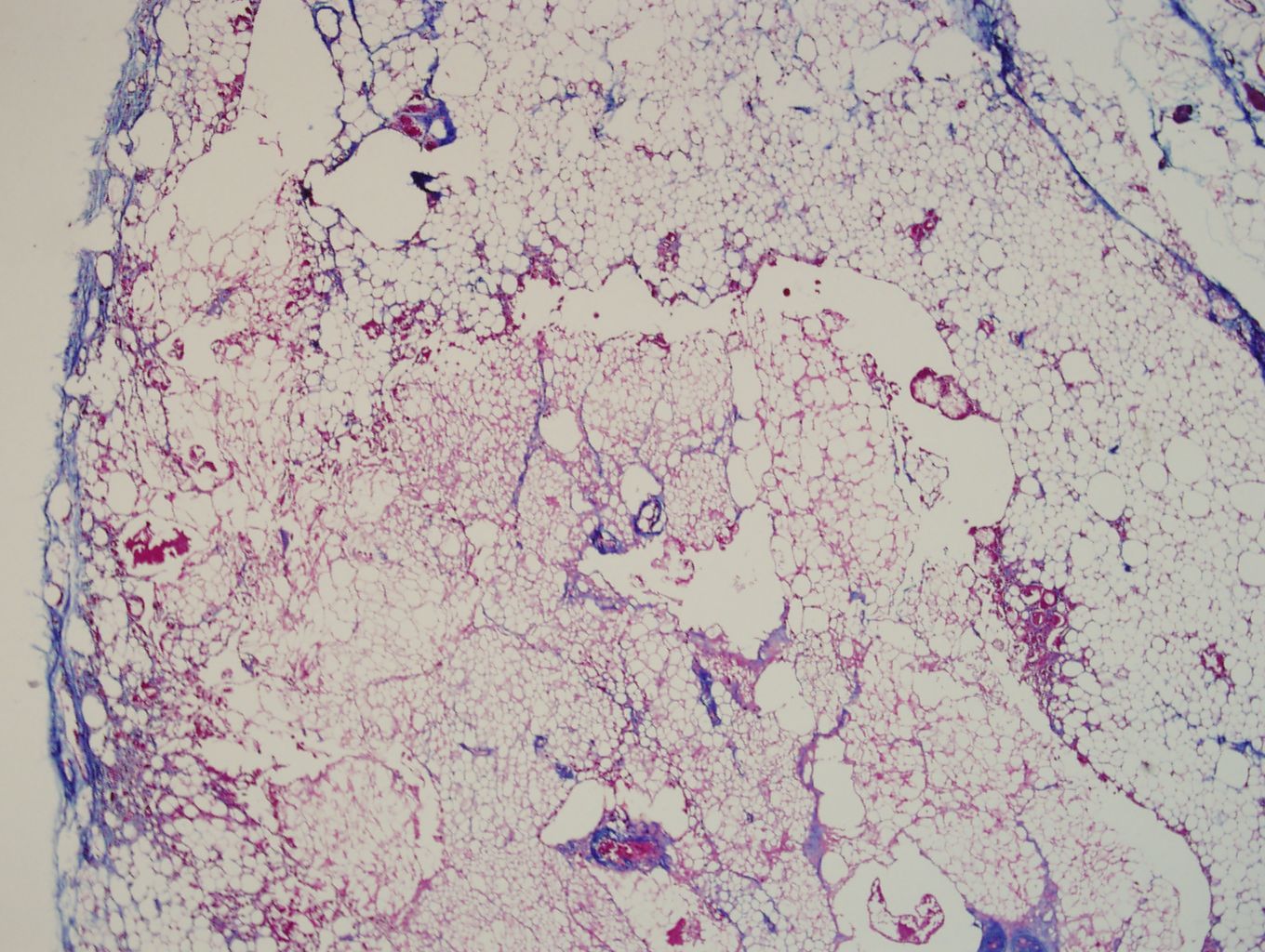

Supplement: Supplementary file 3 [file DataSheet6.ZIP › data for figure 3/masson staining of ND-HC-HF 3M/HF-3M MASSON-30.jpg]

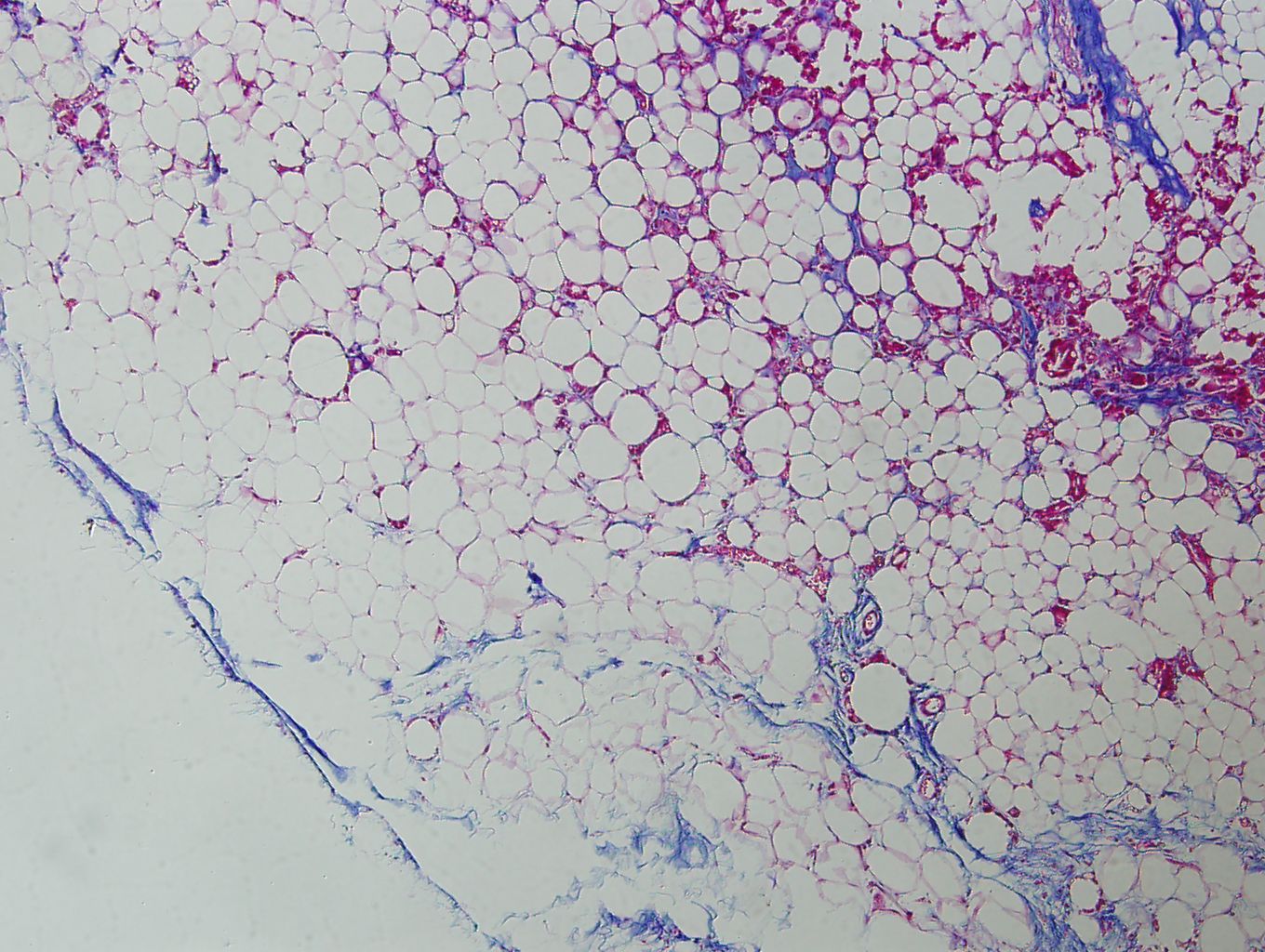

Supplement: Supplementary file 3 [file DataSheet6.ZIP › data for figure 3/masson staining of ND-HC-HF 3M/HF-3M MASSON-36.jpg]

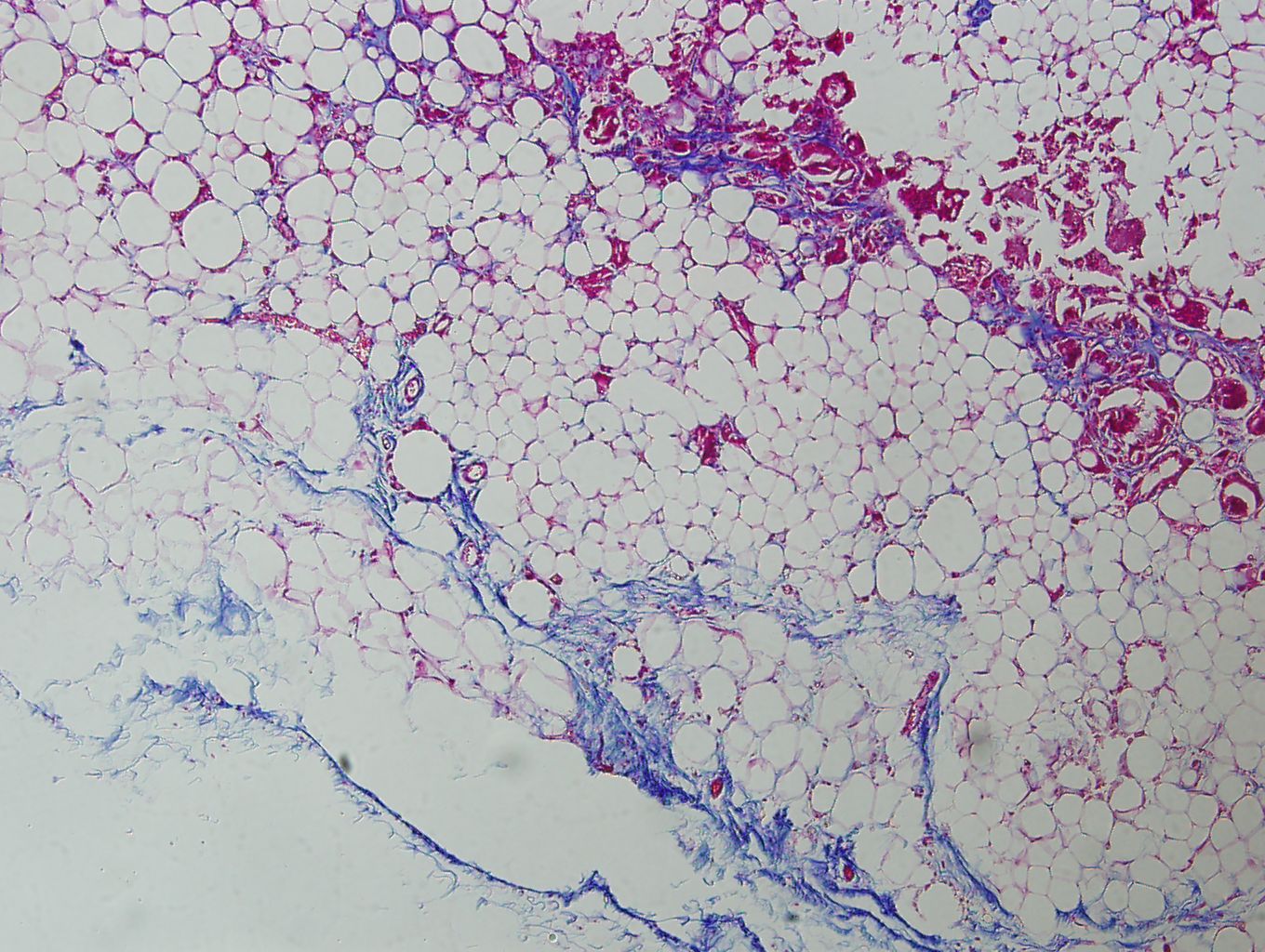

Supplement: Supplementary file 3 [file DataSheet6.ZIP › data for figure 3/masson staining of ND-HC-HF 3M/HF-3M MASSON-37.jpg]

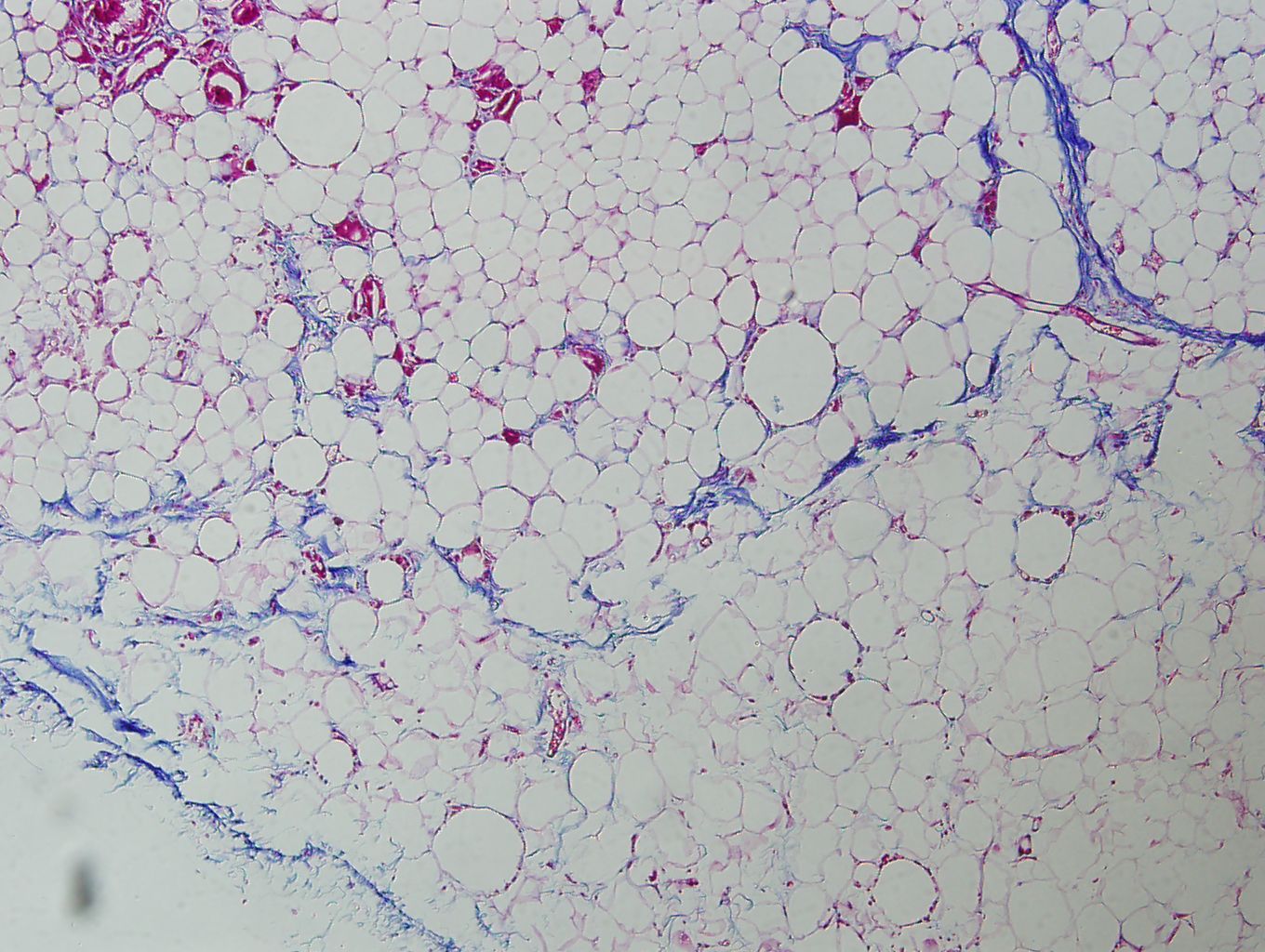

Supplement: Supplementary file 3 [file DataSheet6.ZIP › data for figure 3/masson staining of ND-HC-HF 3M/HF-3M MASSON-38.jpg]

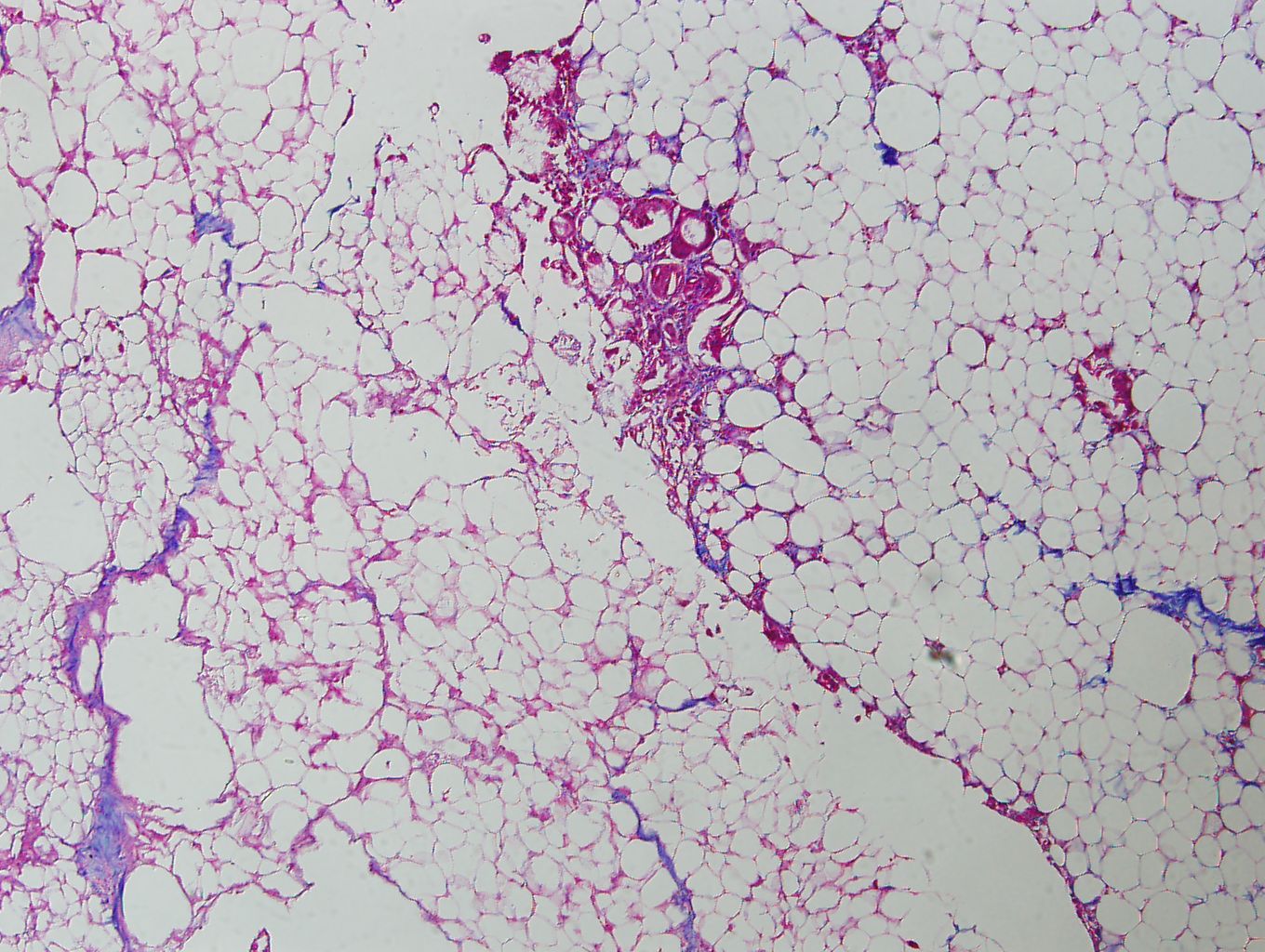

Supplement: Supplementary file 3 [file DataSheet6.ZIP › data for figure 3/masson staining of ND-HC-HF 3M/HF-3M MASSON-45.jpg]

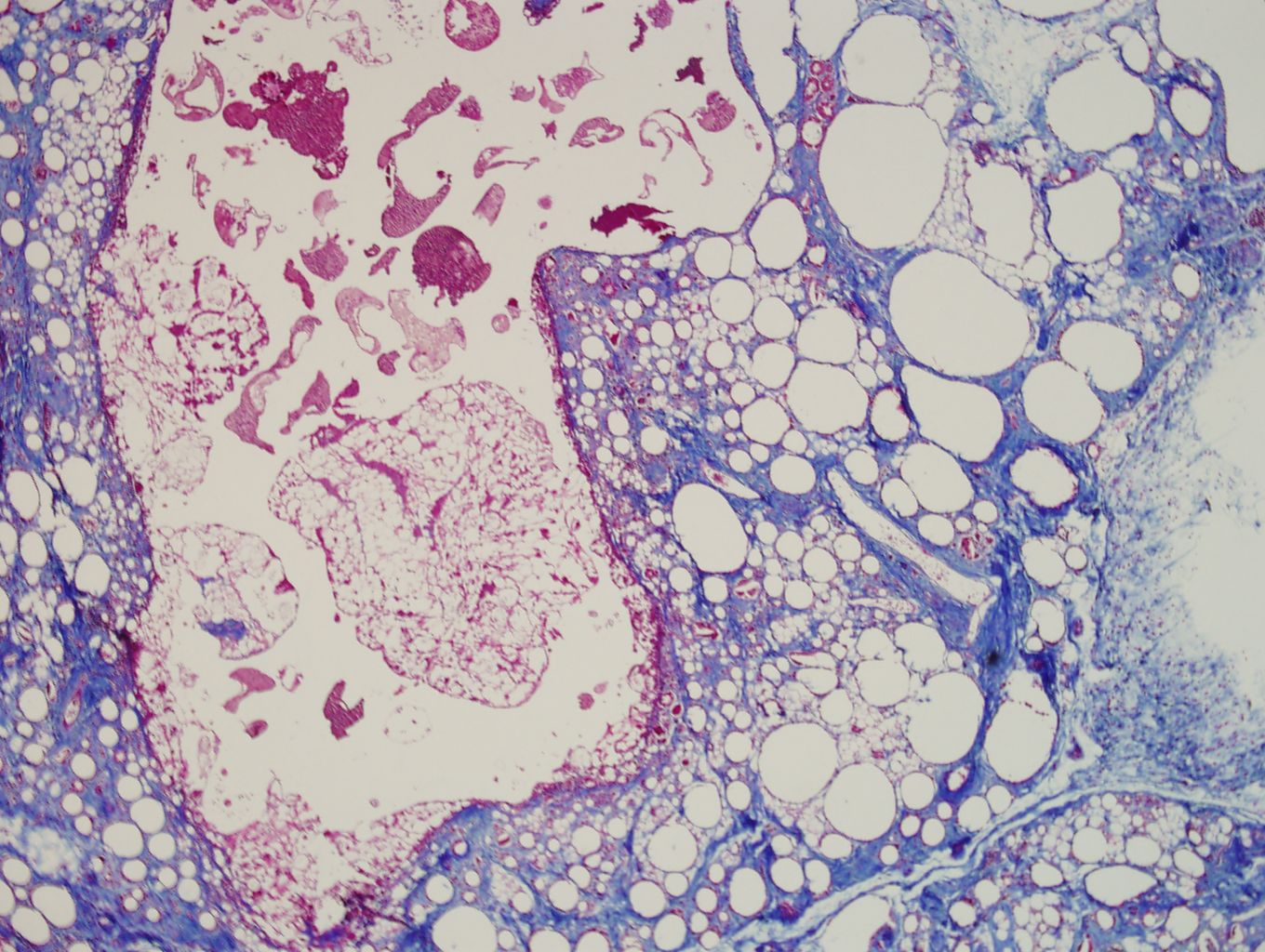

Supplement: Supplementary file 3 [file DataSheet6.ZIP › data for figure 3/masson staining of ND-HC-HF 3M/ND-3M MASSON-101.jpg]

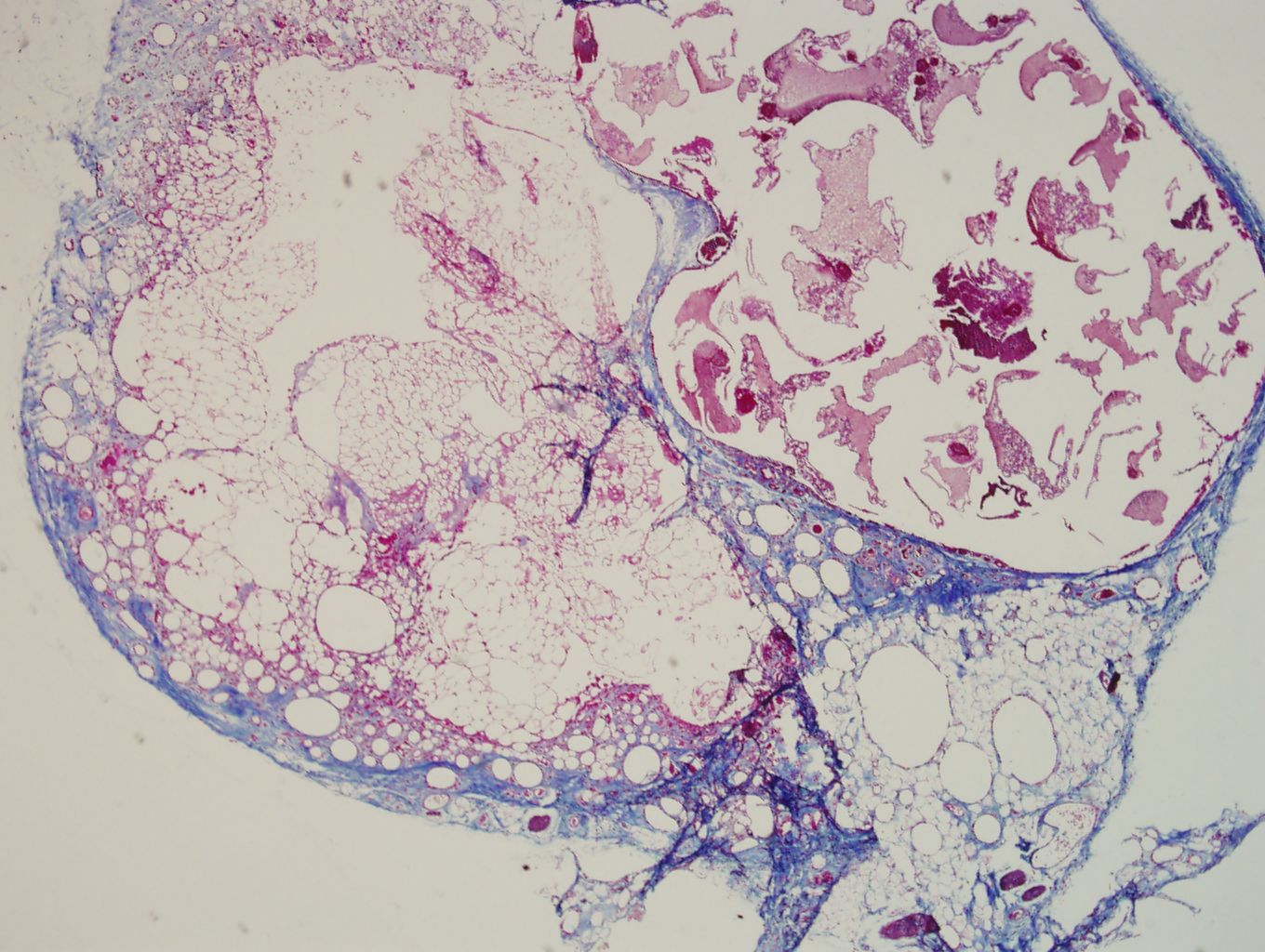

Supplement: Supplementary file 3 [file DataSheet6.ZIP › data for figure 3/masson staining of ND-HC-HF 3M/ND-3M MASSON-103.jpg]

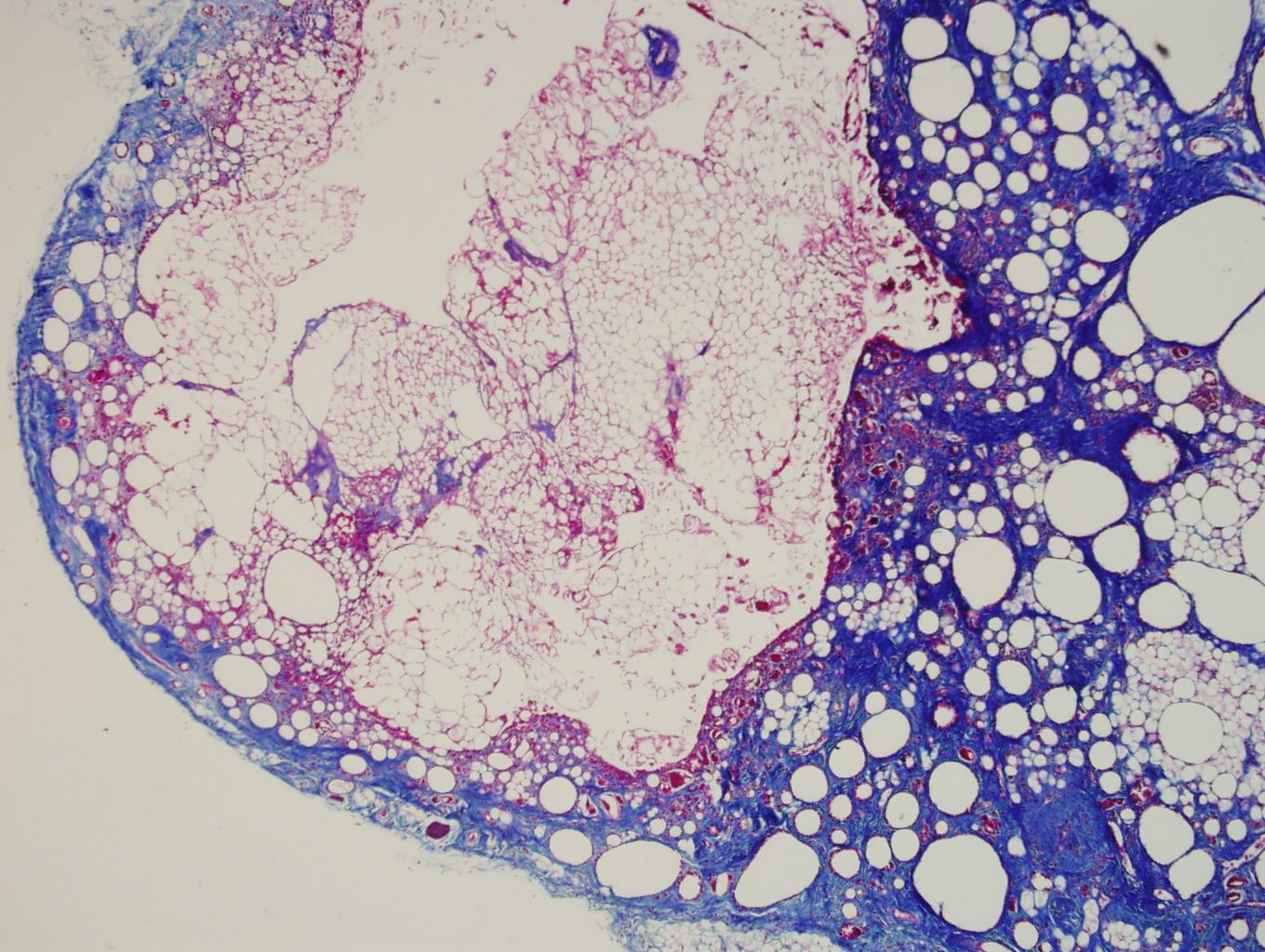

Supplement: Supplementary file 3 [file DataSheet6.ZIP › data for figure 3/masson staining of ND-HC-HF 3M/ND-3M MASSON-105.jpg]

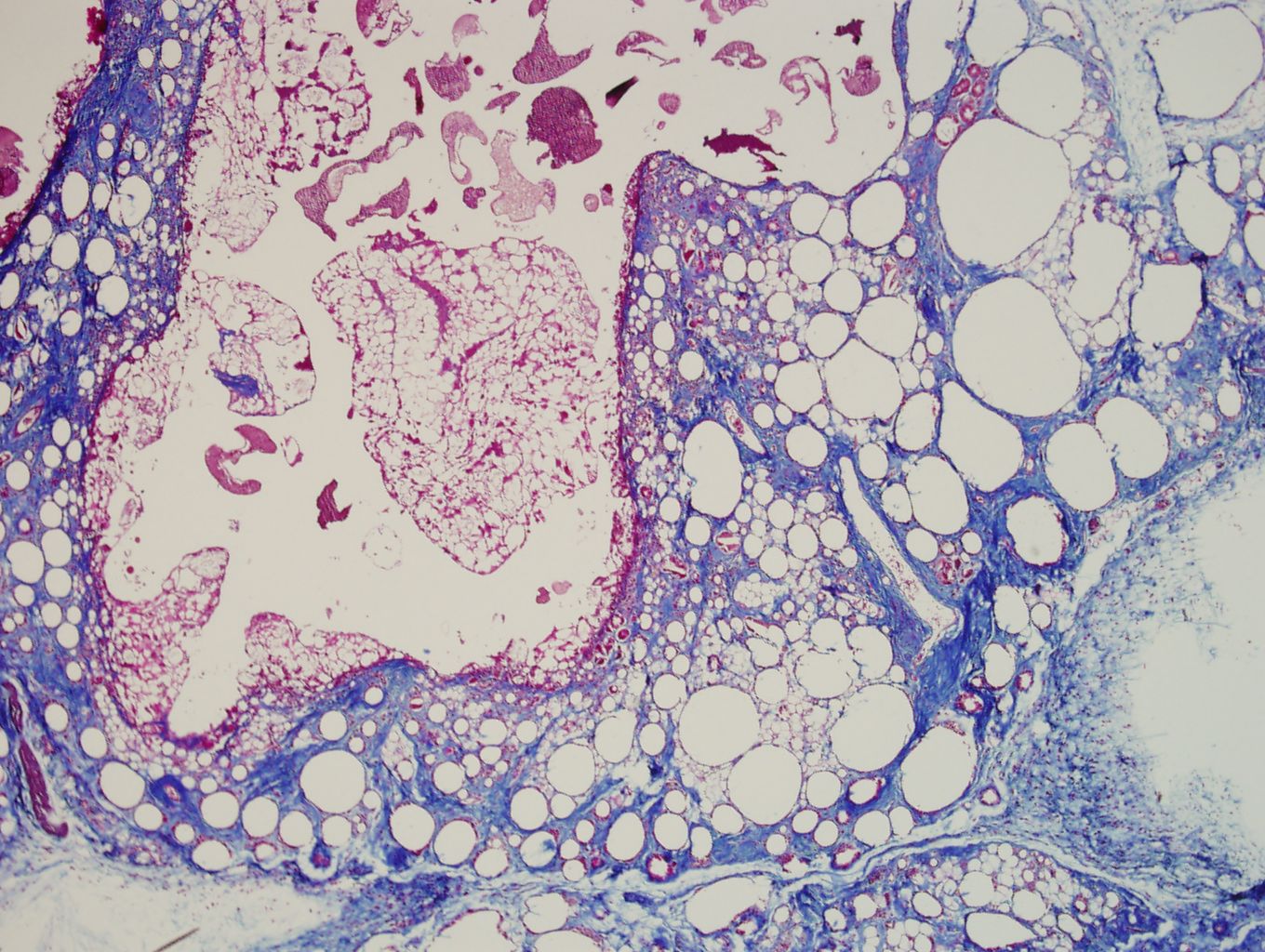

Supplement: Supplementary file 3 [file DataSheet6.ZIP › data for figure 3/masson staining of ND-HC-HF 3M/ND-3M MASSON-75.jpg]

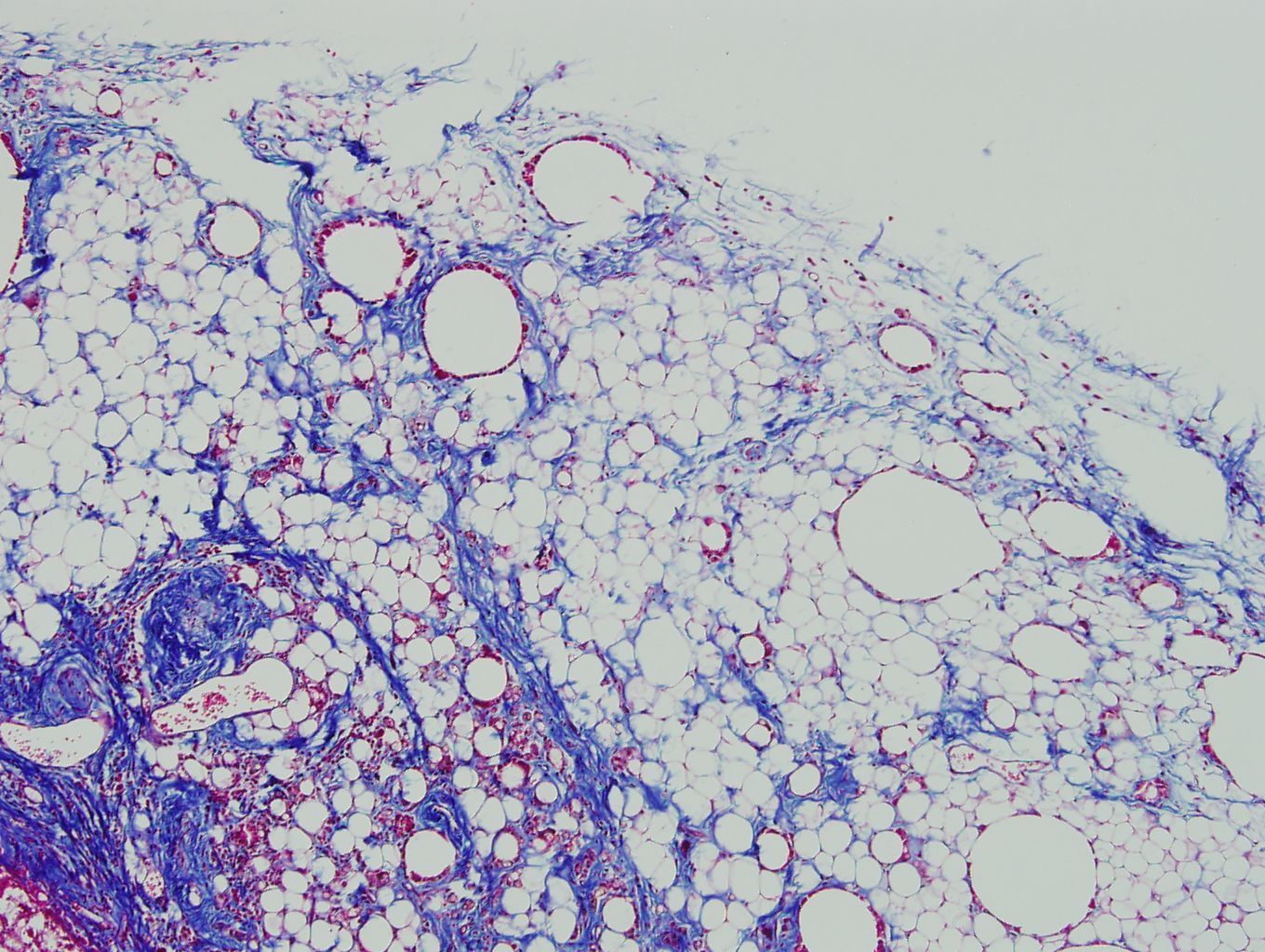

Supplement: Supplementary file 3 [file DataSheet6.ZIP › data for figure 3/masson staining of ND-HC-HF 3M/ND-3M MASSON-79.jpg]

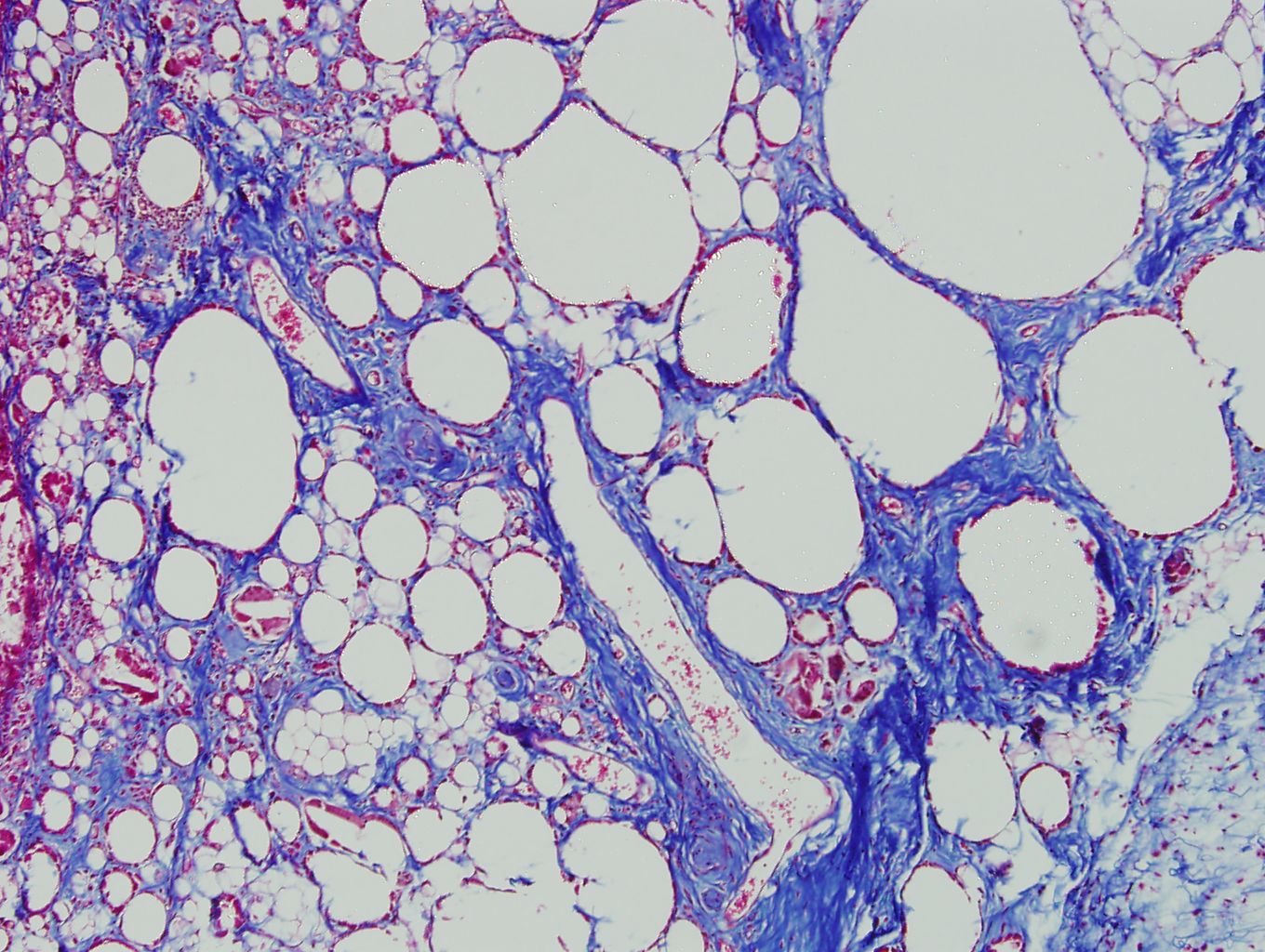

Supplement: Supplementary file 3 [file DataSheet6.ZIP › data for figure 3/masson staining of ND-HC-HF 3M/ND-3M MASSON-86.jpg]

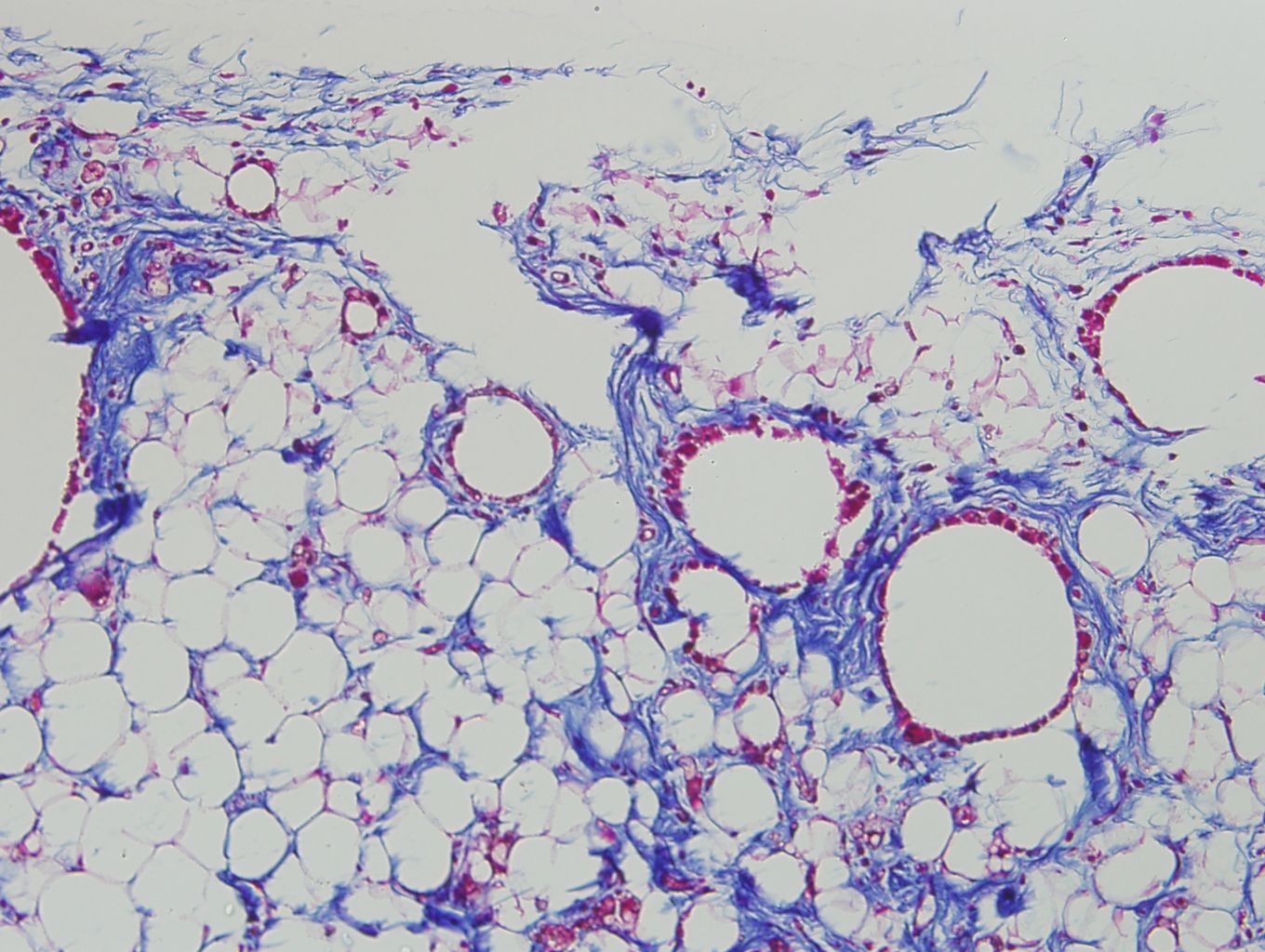

Supplement: Supplementary file 3 [file DataSheet6.ZIP › data for figure 3/masson staining of ND-HC-HF 3M/ND-3M MASSON-89.jpg]

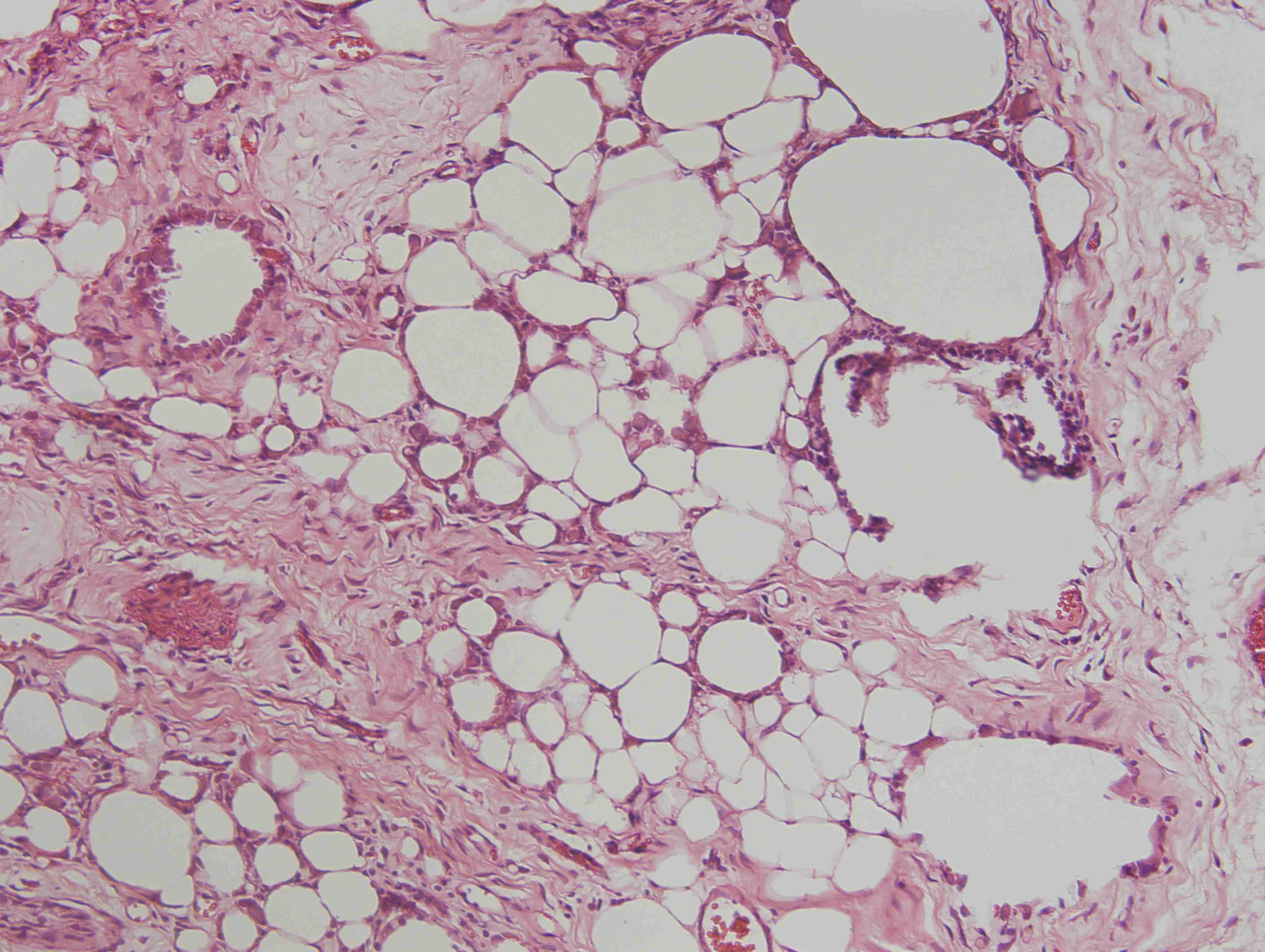

Supplement: Supplementary file 5 [file DataSheet5.ZIP › data for figure 2/ND-HC-HF 1M HE figure/HC-1M-1-12.jpg]

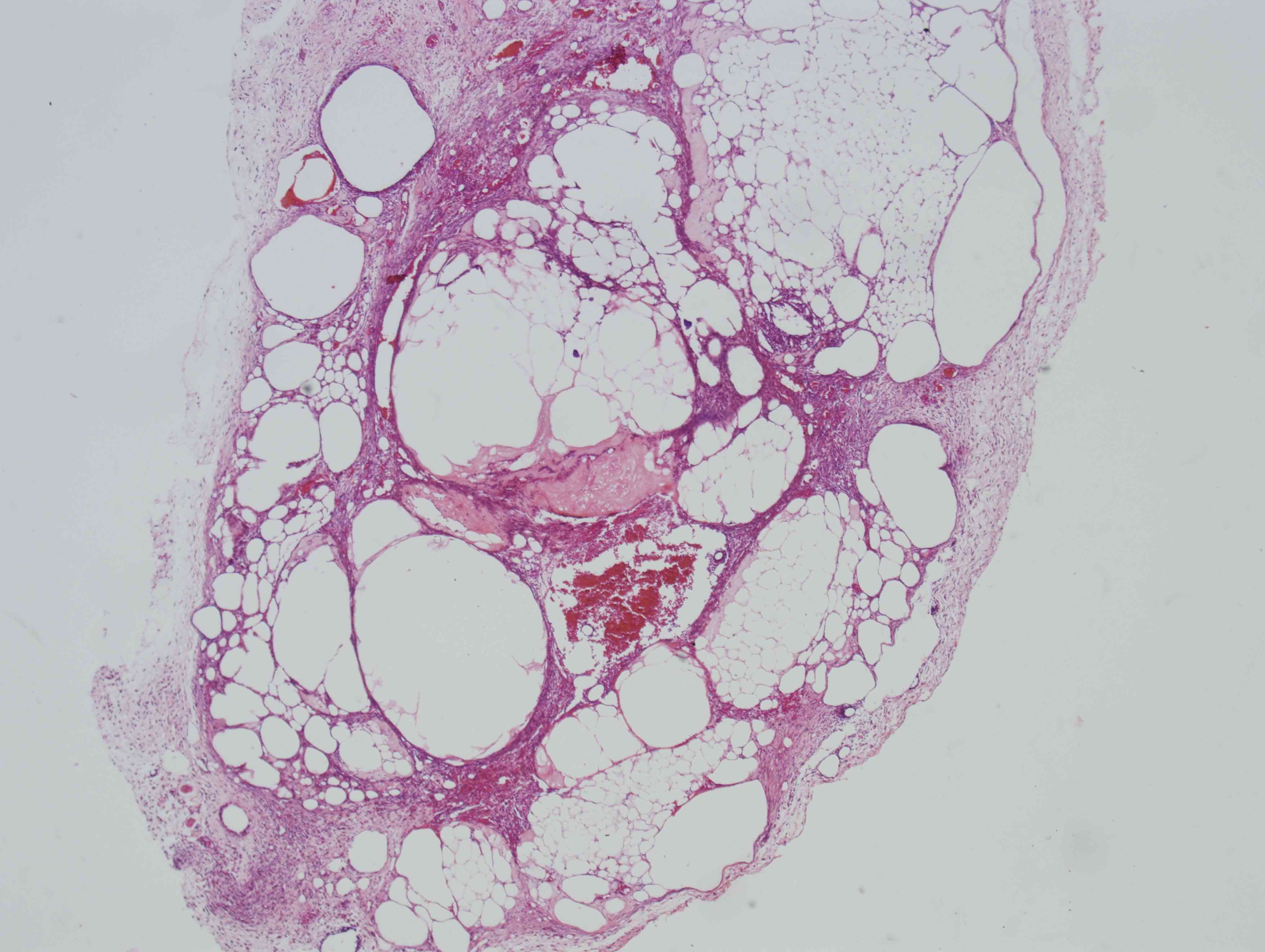

Supplement: Supplementary file 5 [file DataSheet5.ZIP › data for figure 2/ND-HC-HF 1M HE figure/HC-1M-1-3.jpg]

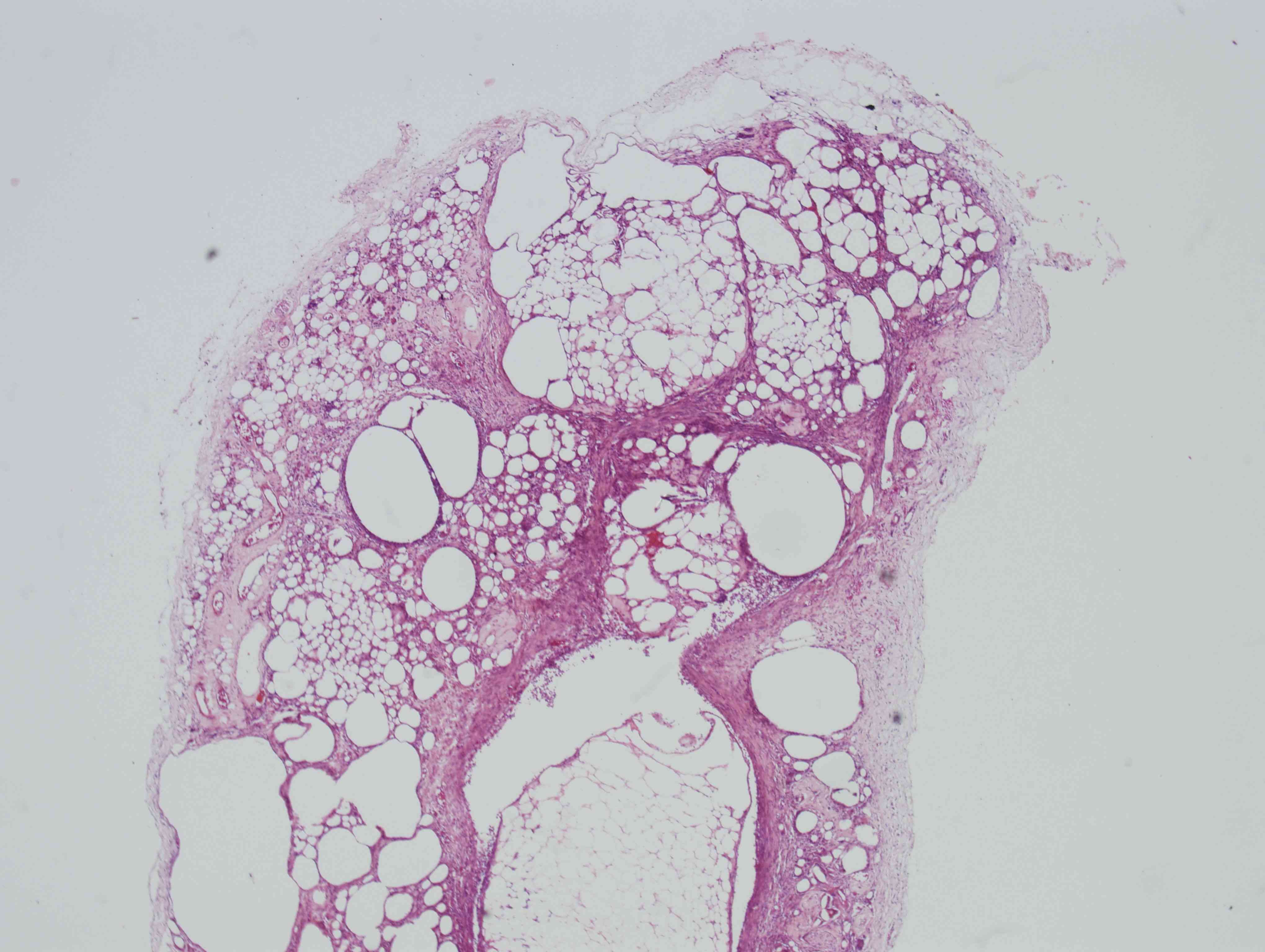

Supplement: Supplementary file 5 [file DataSheet5.ZIP › data for figure 2/ND-HC-HF 1M HE figure/HC-1M-2-1.jpg]

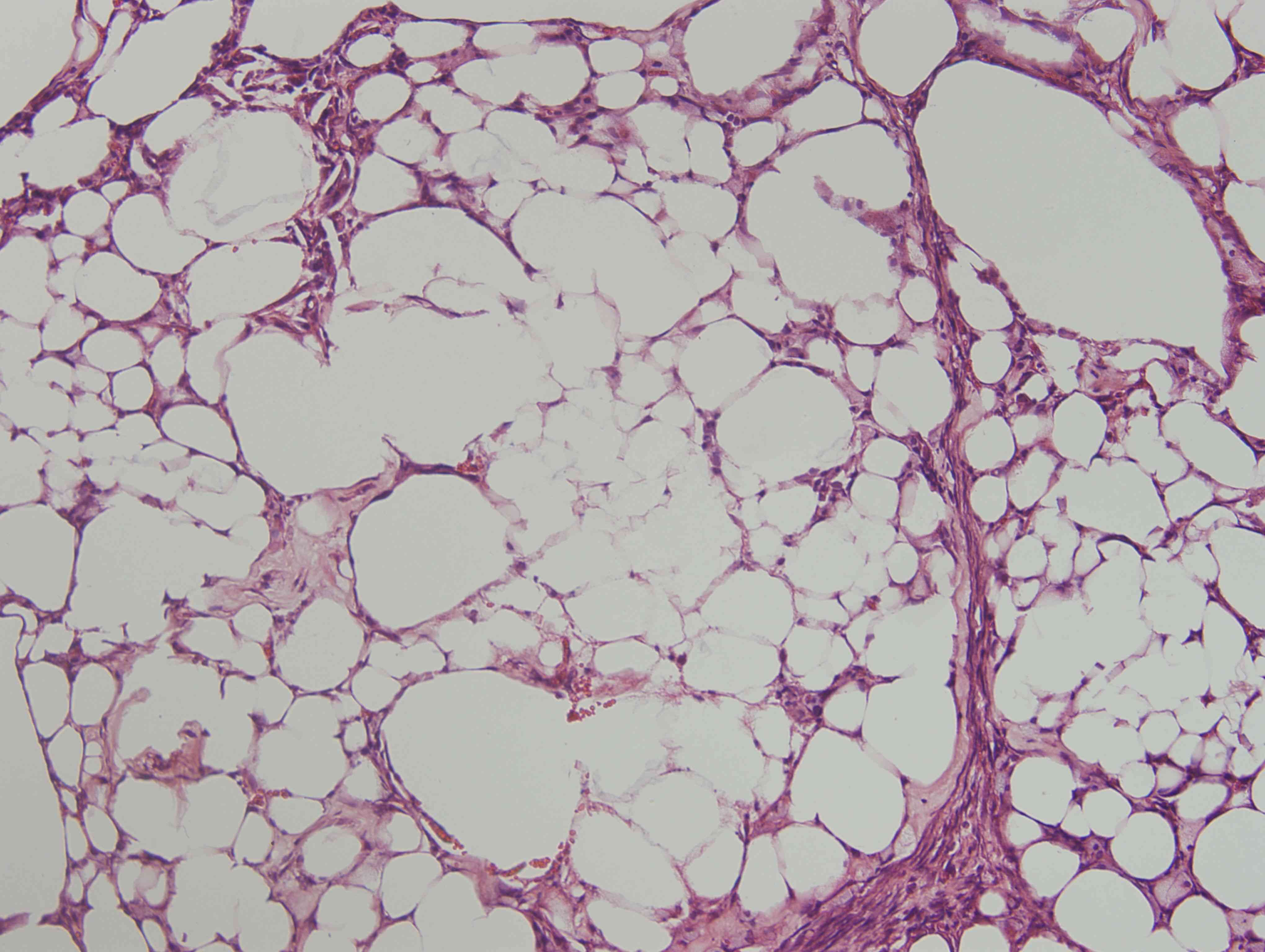

Supplement: Supplementary file 5 [file DataSheet5.ZIP › data for figure 2/ND-HC-HF 1M HE figure/HC-1M-2-10.jpg]

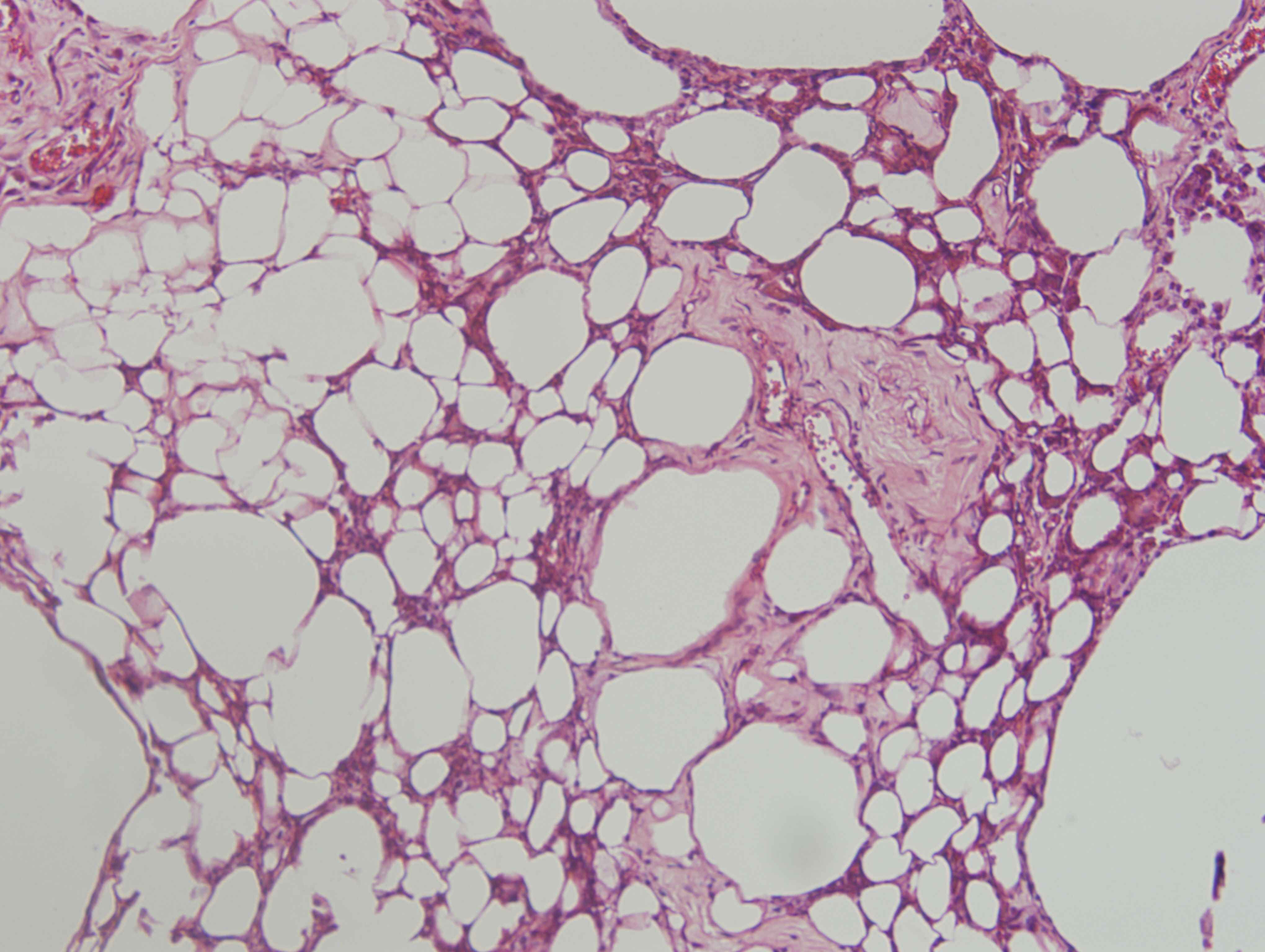

Supplement: Supplementary file 5 [file DataSheet5.ZIP › data for figure 2/ND-HC-HF 1M HE figure/HC-1M-2-13.jpg]

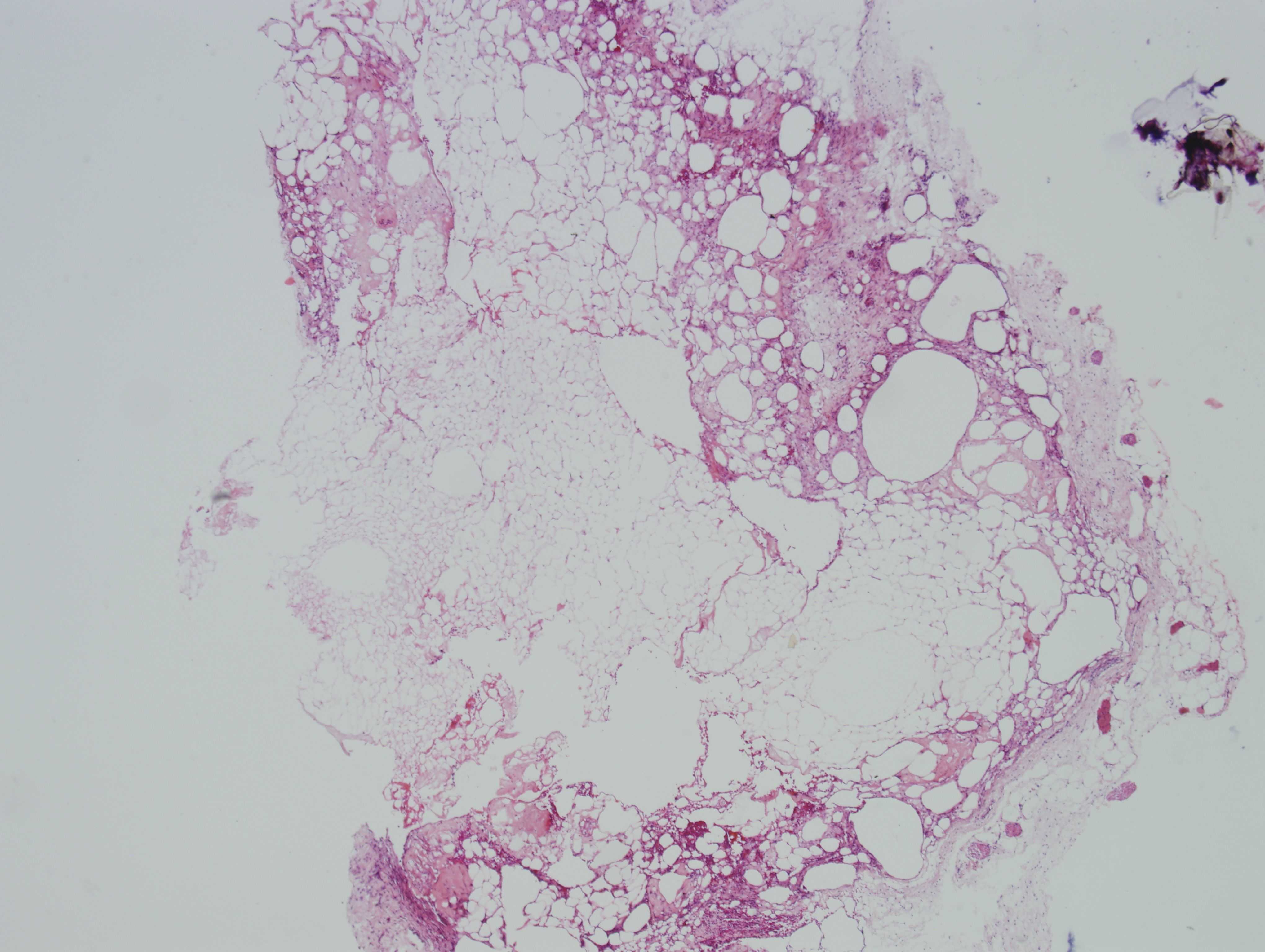

Supplement: Supplementary file 5 [file DataSheet5.ZIP › data for figure 2/ND-HC-HF 1M HE figure/HC-1M-2-2.jpg]

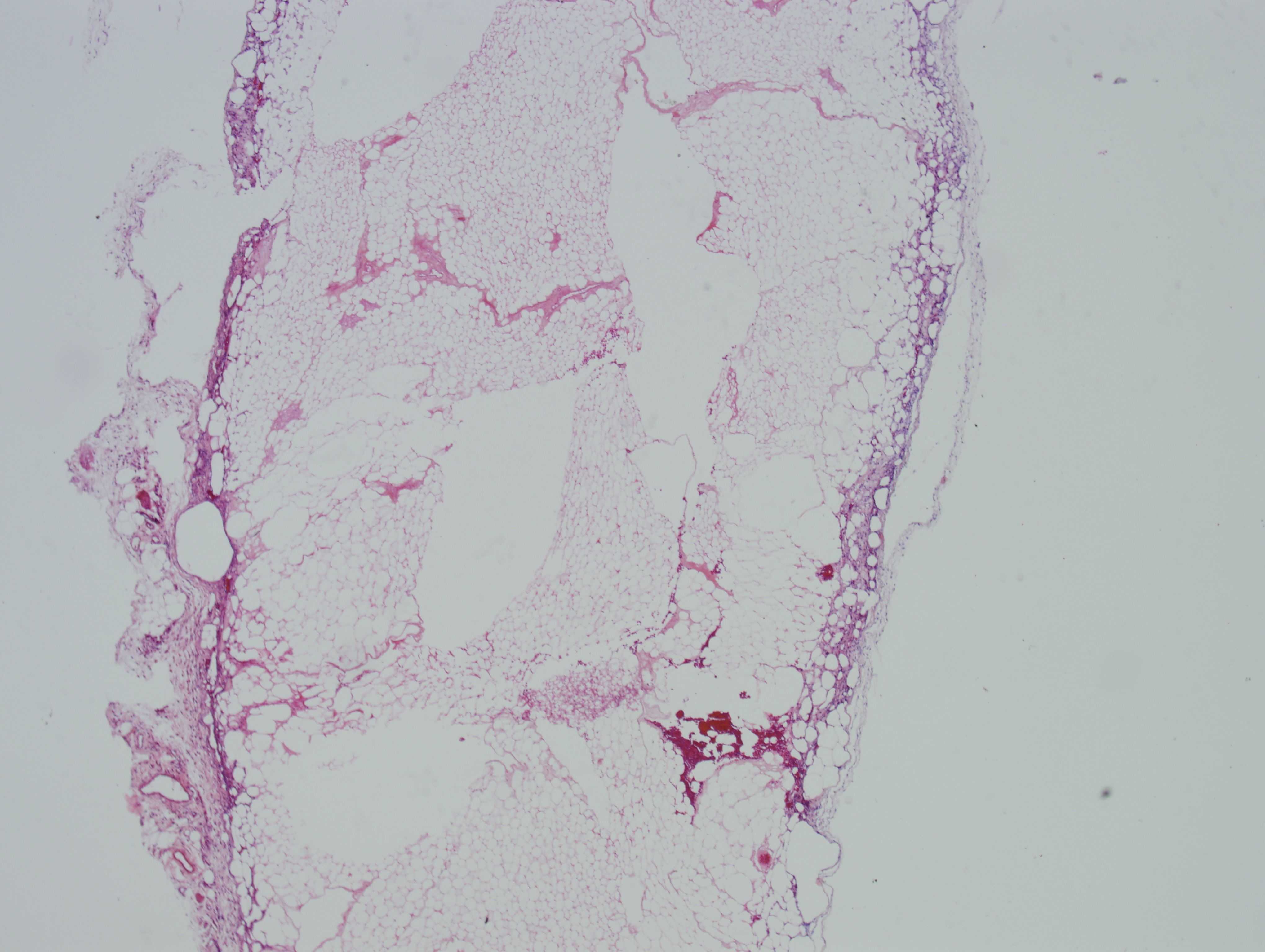

Supplement: Supplementary file 5 [file DataSheet5.ZIP › data for figure 2/ND-HC-HF 1M HE figure/HF-1M-2-20.jpg]

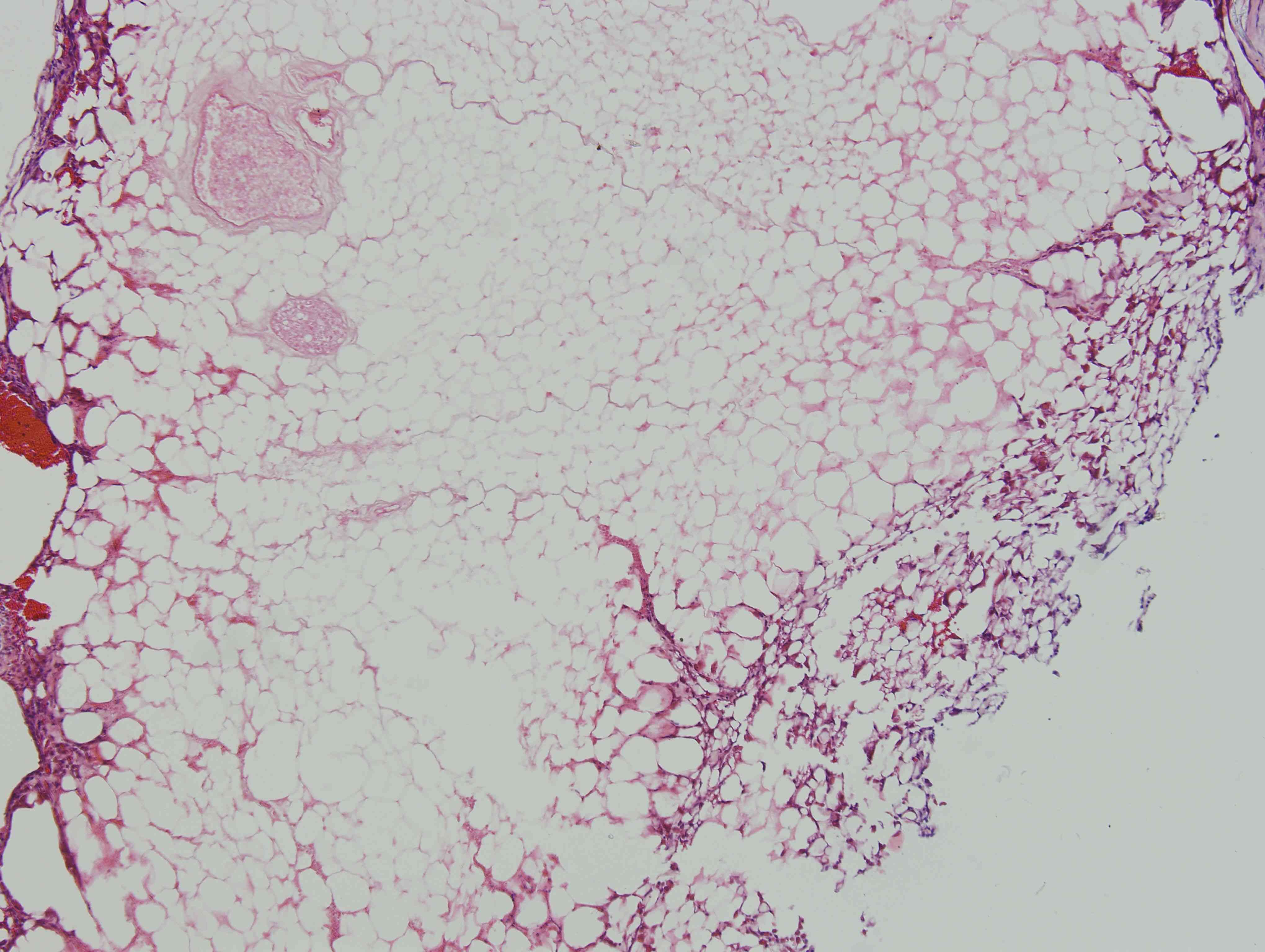

Supplement: Supplementary file 5 [file DataSheet5.ZIP › data for figure 2/ND-HC-HF 1M HE figure/HF-1M-2-24.jpg]

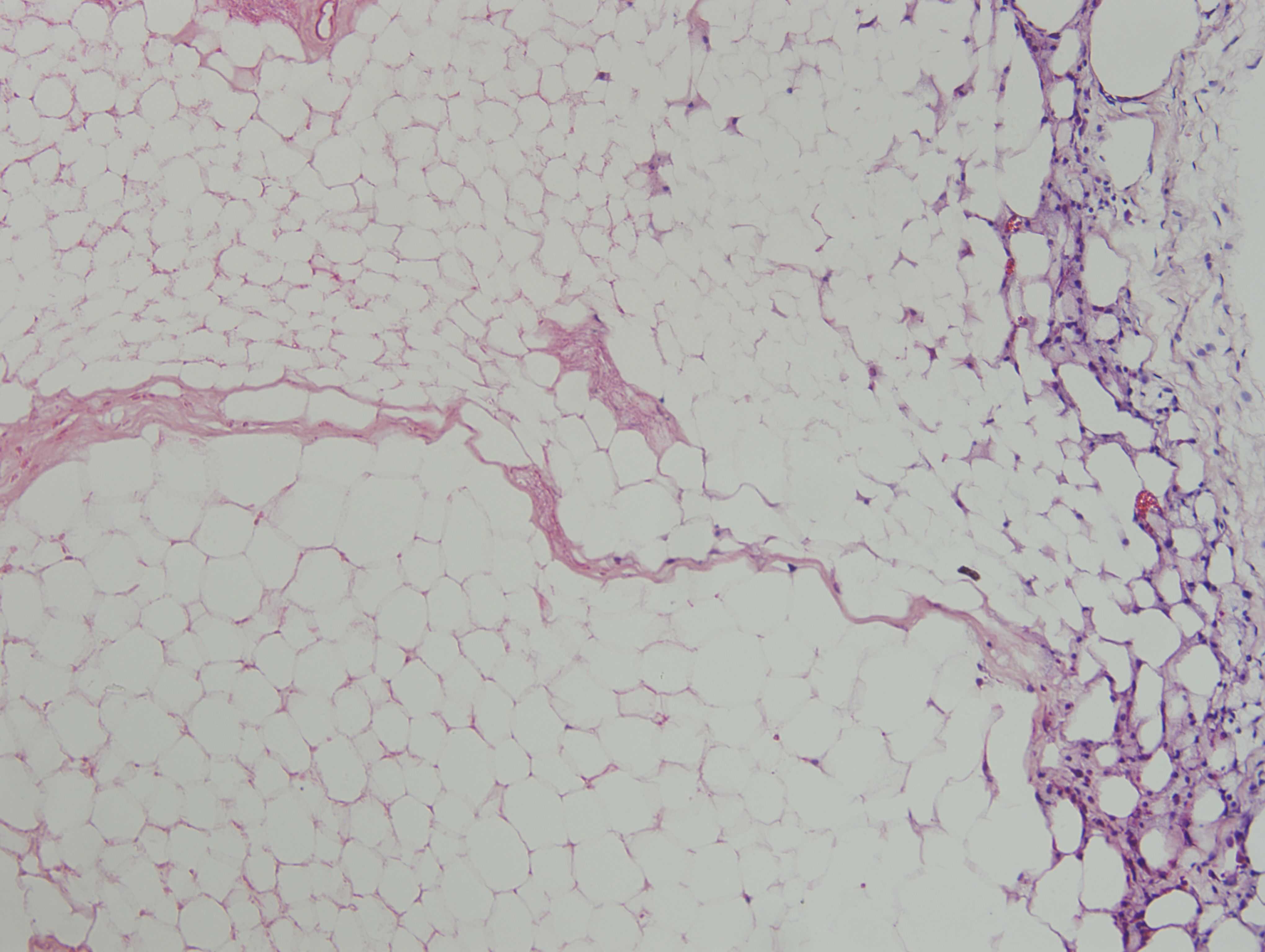

Supplement: Supplementary file 5 [file DataSheet5.ZIP › data for figure 2/ND-HC-HF 1M HE figure/HF-1M-2-26.jpg]

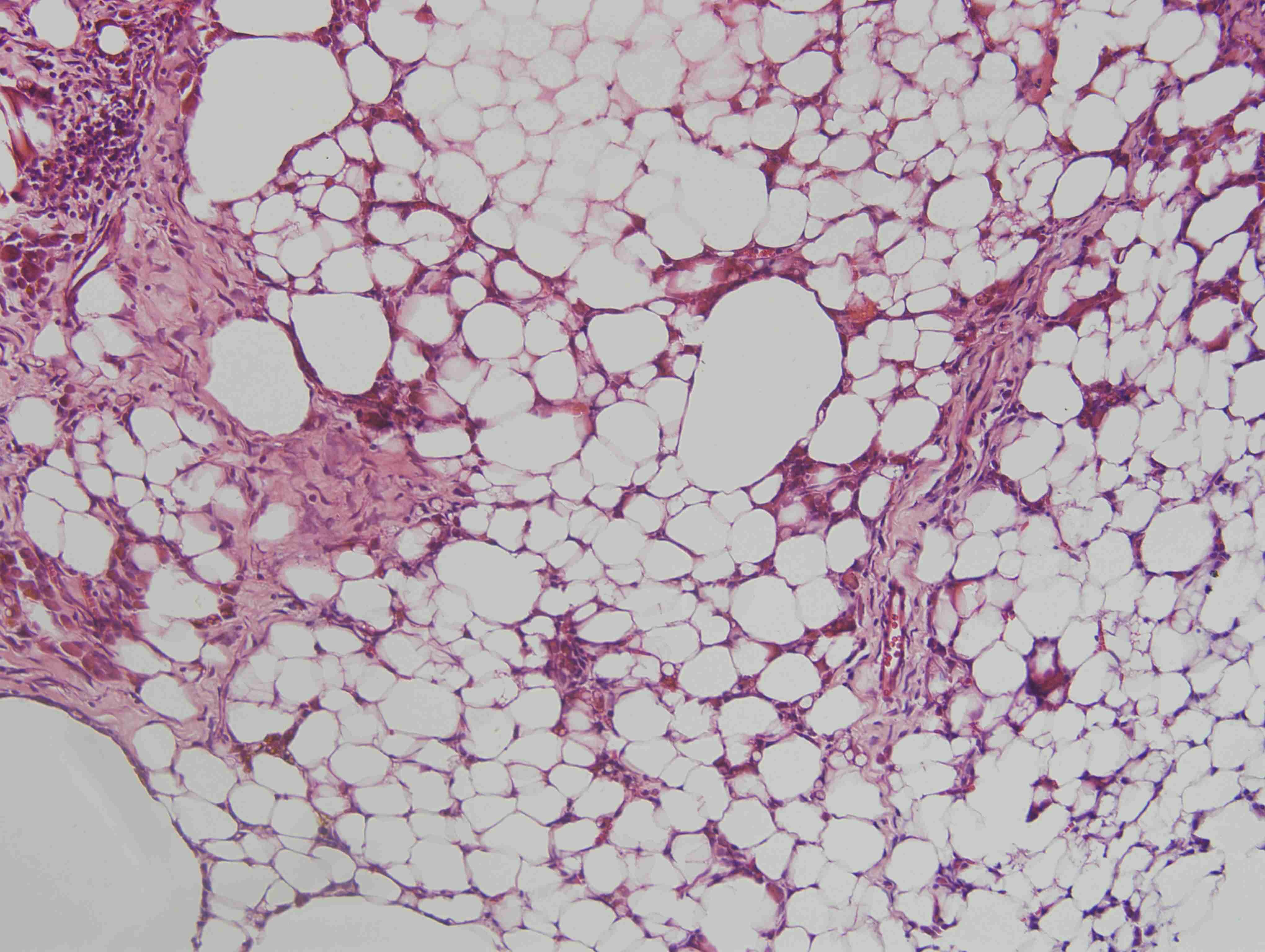

Supplement: Supplementary file 5 [file DataSheet5.ZIP › data for figure 2/ND-HC-HF 1M HE figure/HF-1M-2-31.jpg]

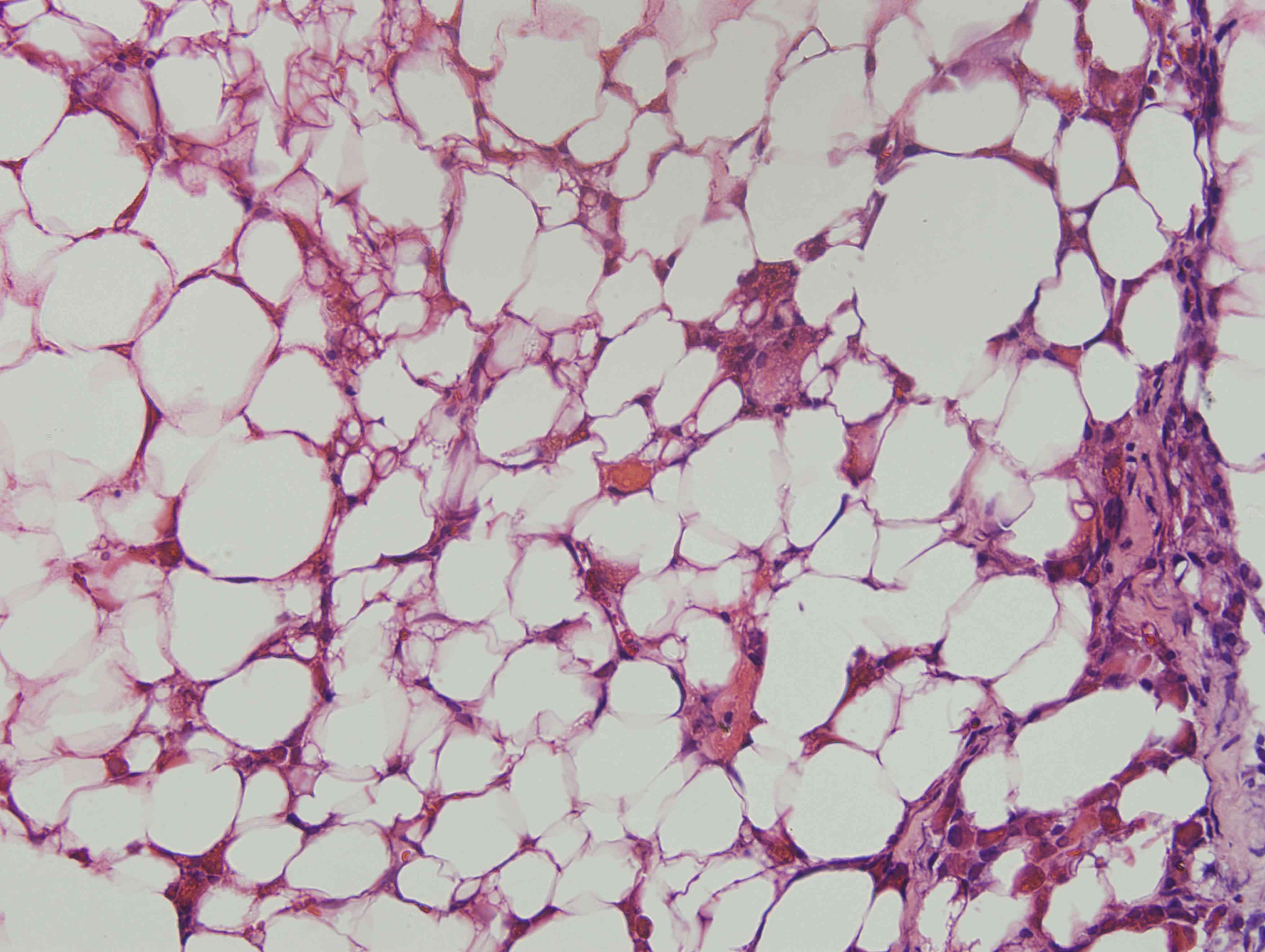

Supplement: Supplementary file 5 [file DataSheet5.ZIP › data for figure 2/ND-HC-HF 1M HE figure/HF-1M-2-33.jpg]

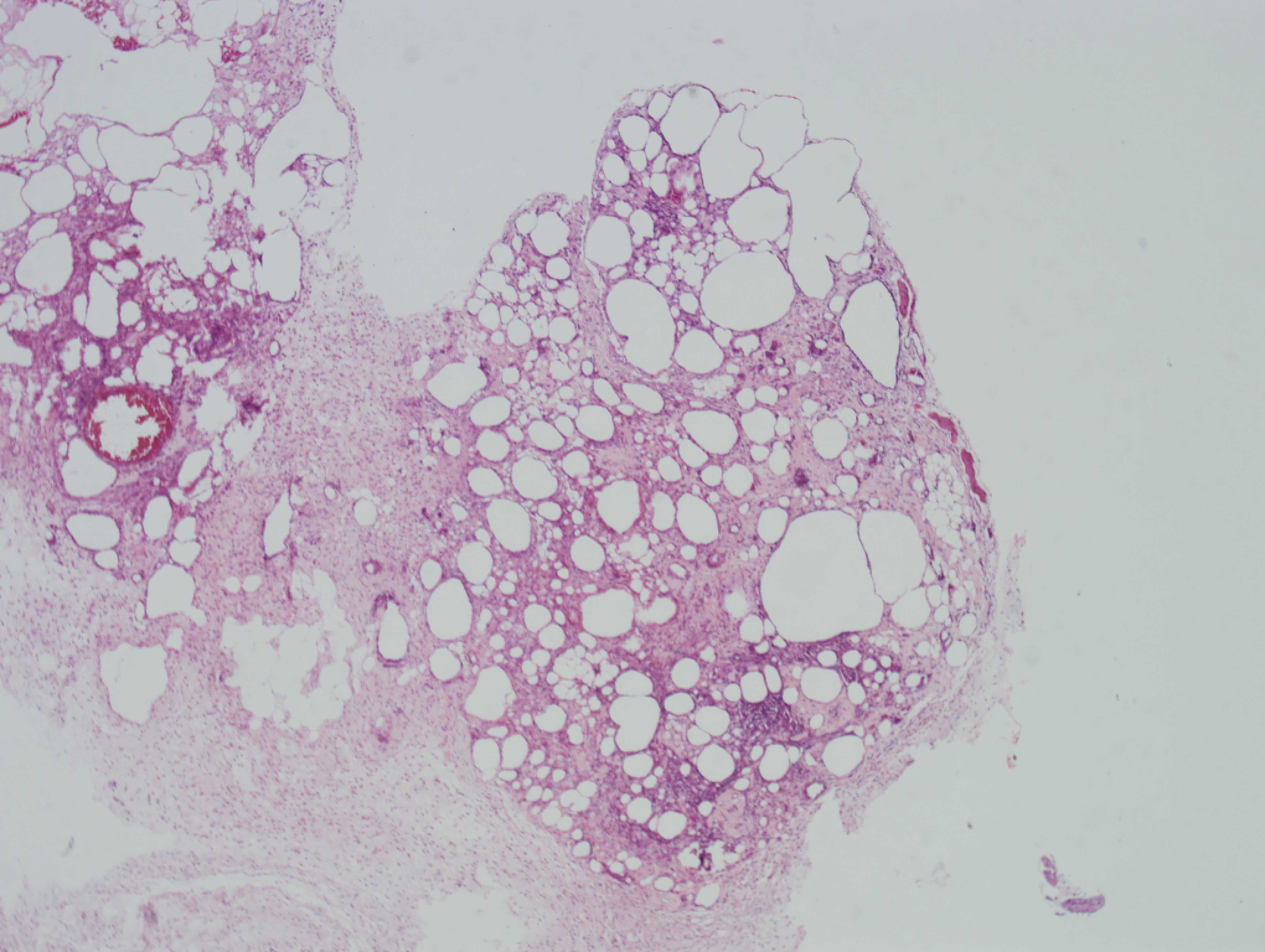

Supplement: Supplementary file 5 [file DataSheet5.ZIP › data for figure 2/ND-HC-HF 1M HE figure/ND-1M-1-1.jpg]
