# Supplementary figures and images for: A Short-Term High-Fat Diet Improved the Survival of Fat Grafts in Mice by Promoting Macrophage Infiltration and Angiogenesis (part 2 of 2)
Source: Front Cell Dev Biol. 2022 Mar 17;10:856839. doi: 10.3389/fcell.2022.856839 (PMC8968084; doi:10.3389/fcell.2022.856839)

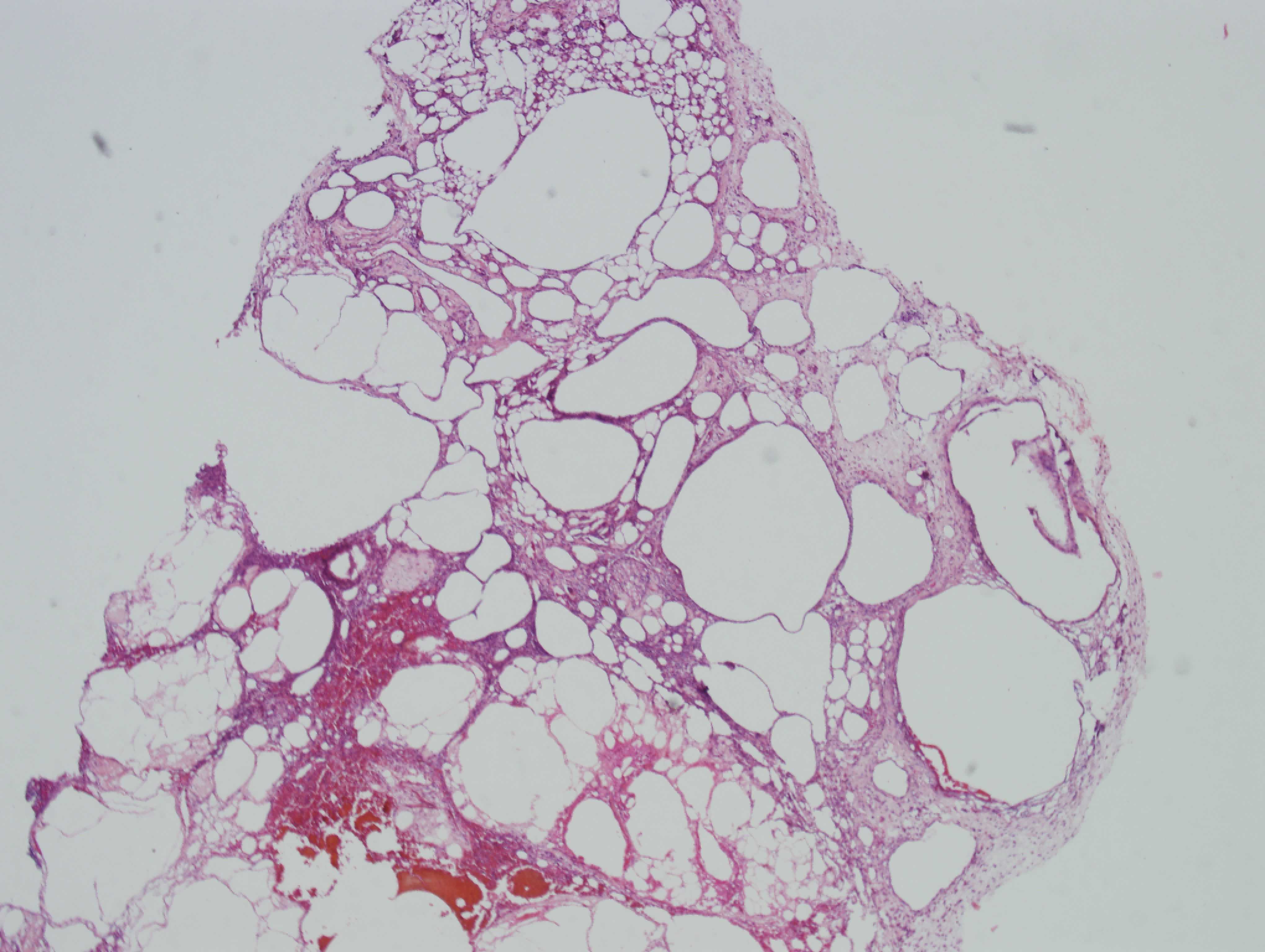

Supplement: Supplementary file 5 [file DataSheet5.ZIP › data for figure 2/ND-HC-HF 1M HE figure/ND-1M-1-2.jpg]

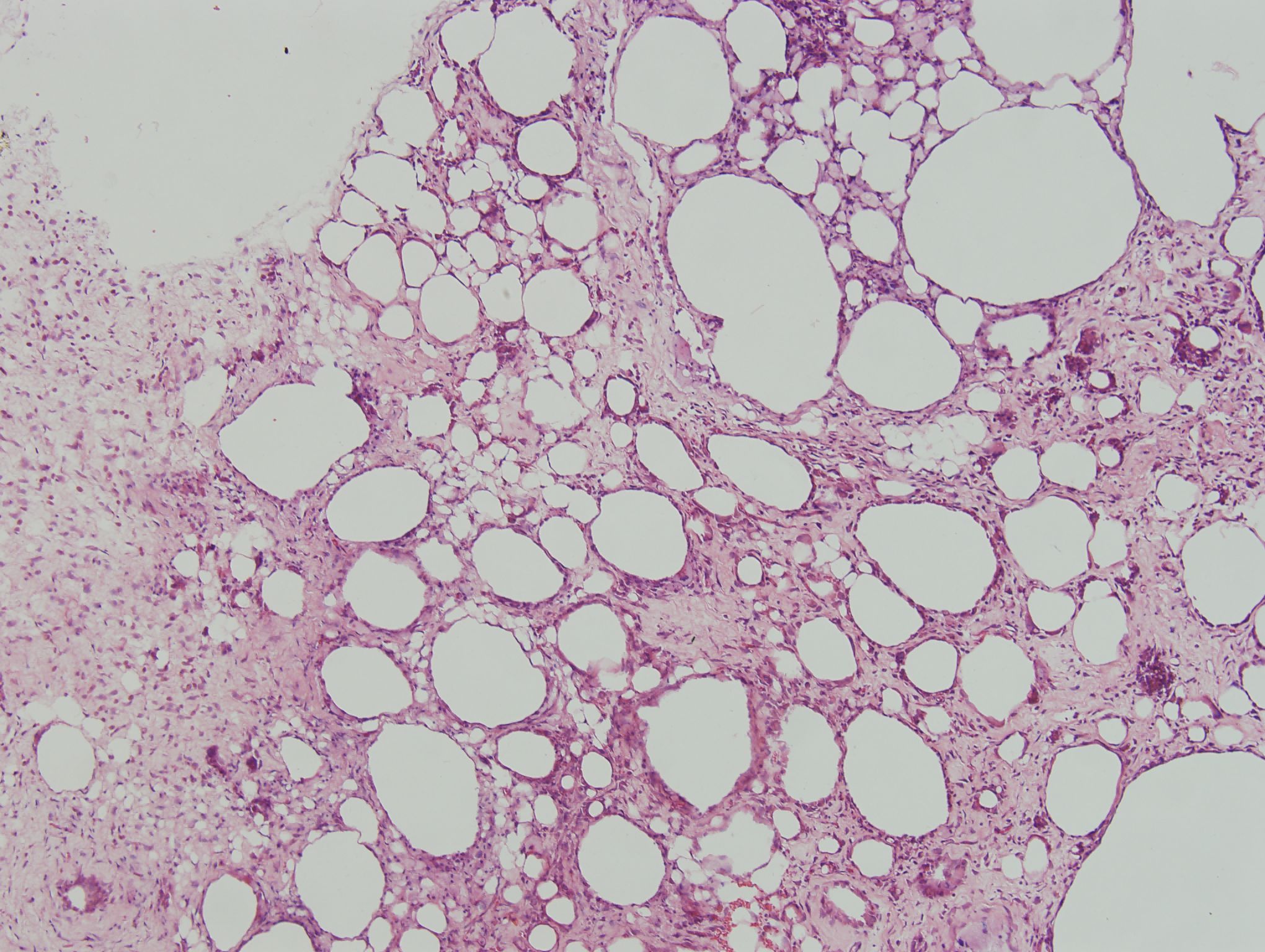

Supplement: Supplementary file 5 [file DataSheet5.ZIP › data for figure 2/ND-HC-HF 1M HE figure/ND-1M-1-7.jpg]

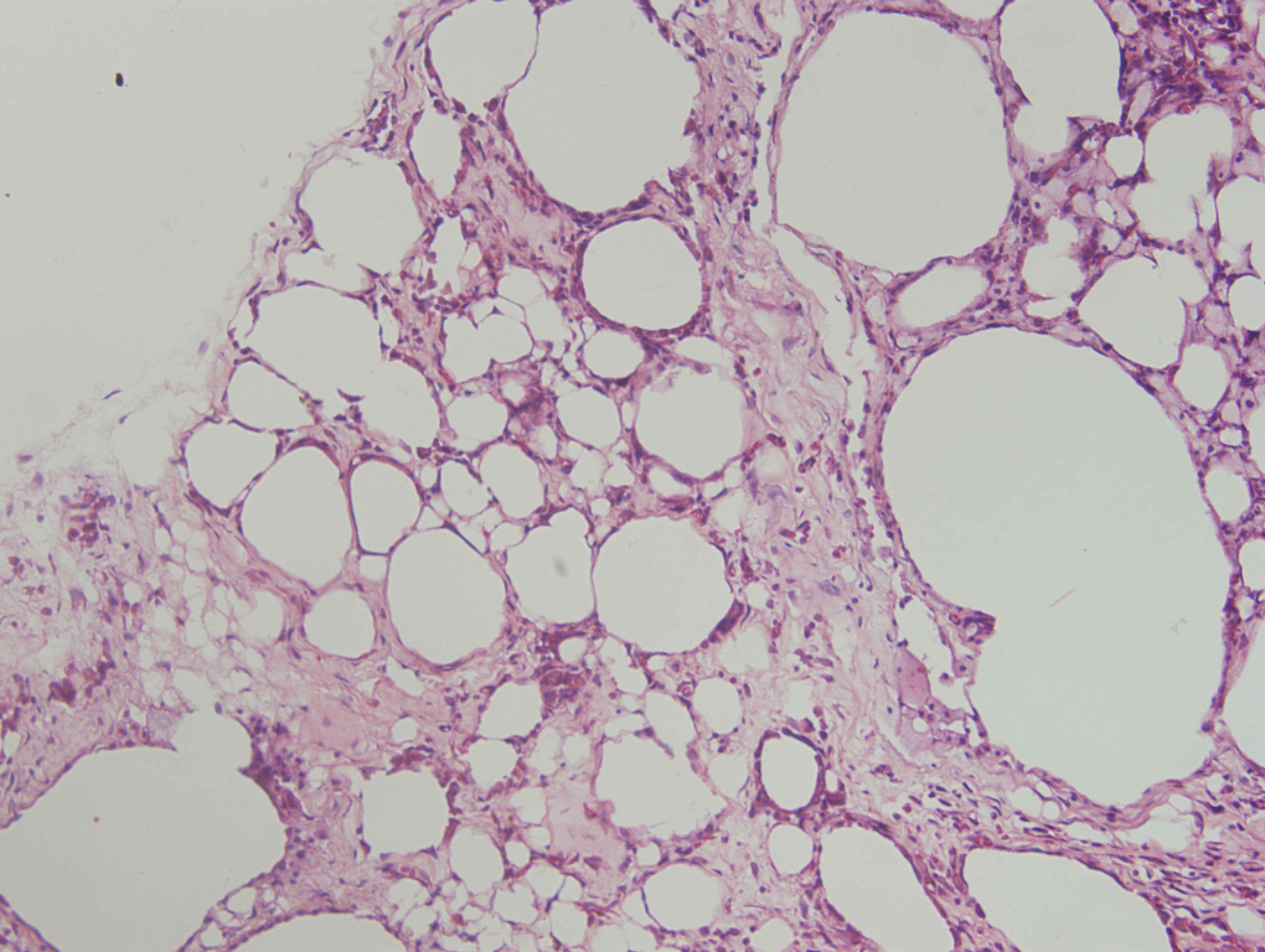

Supplement: Supplementary file 5 [file DataSheet5.ZIP › data for figure 2/ND-HC-HF 1M HE figure/ND-1M-1-8.jpg]

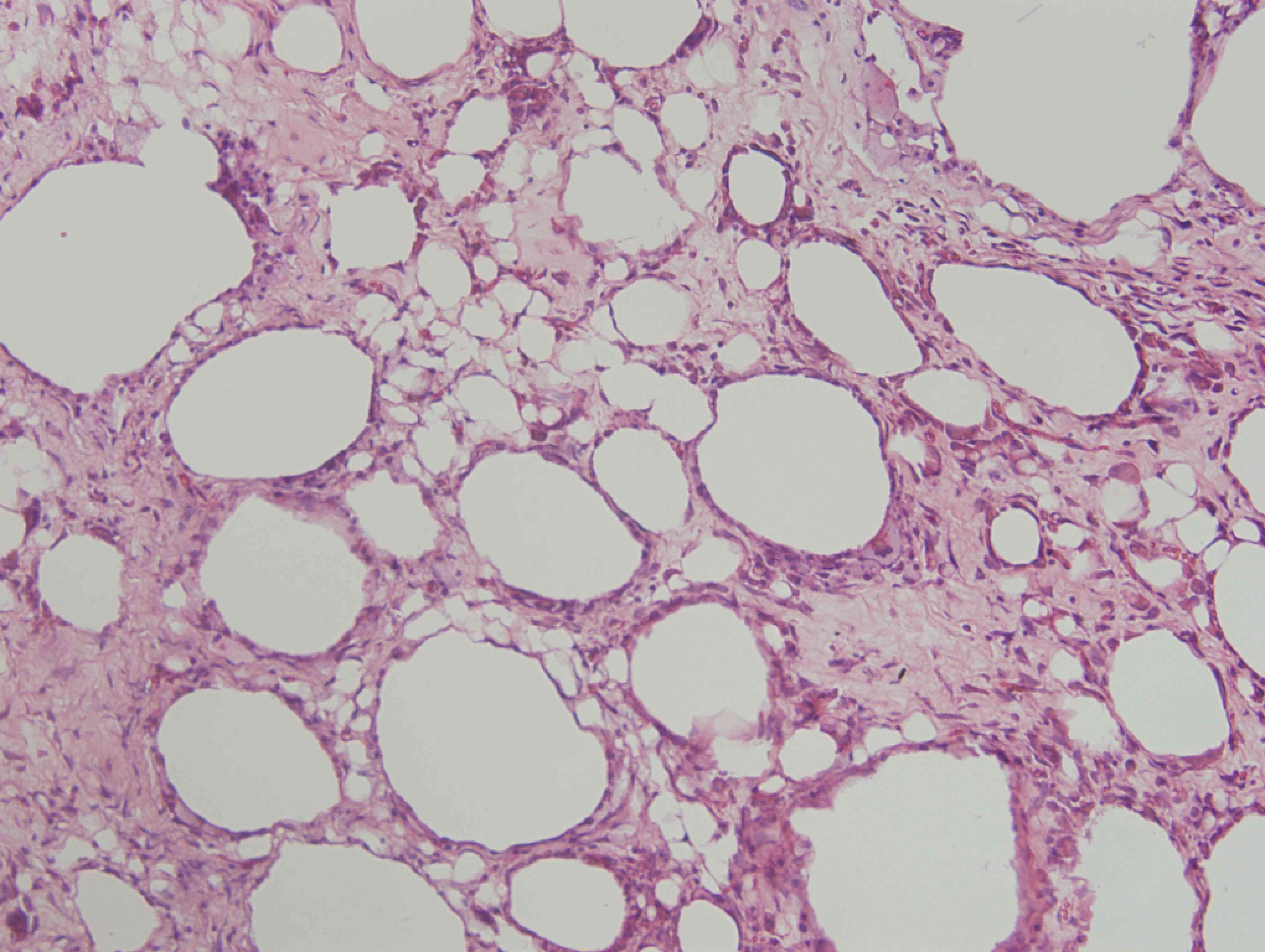

Supplement: Supplementary file 5 [file DataSheet5.ZIP › data for figure 2/ND-HC-HF 1M HE figure/ND-1M-1-9.jpg]

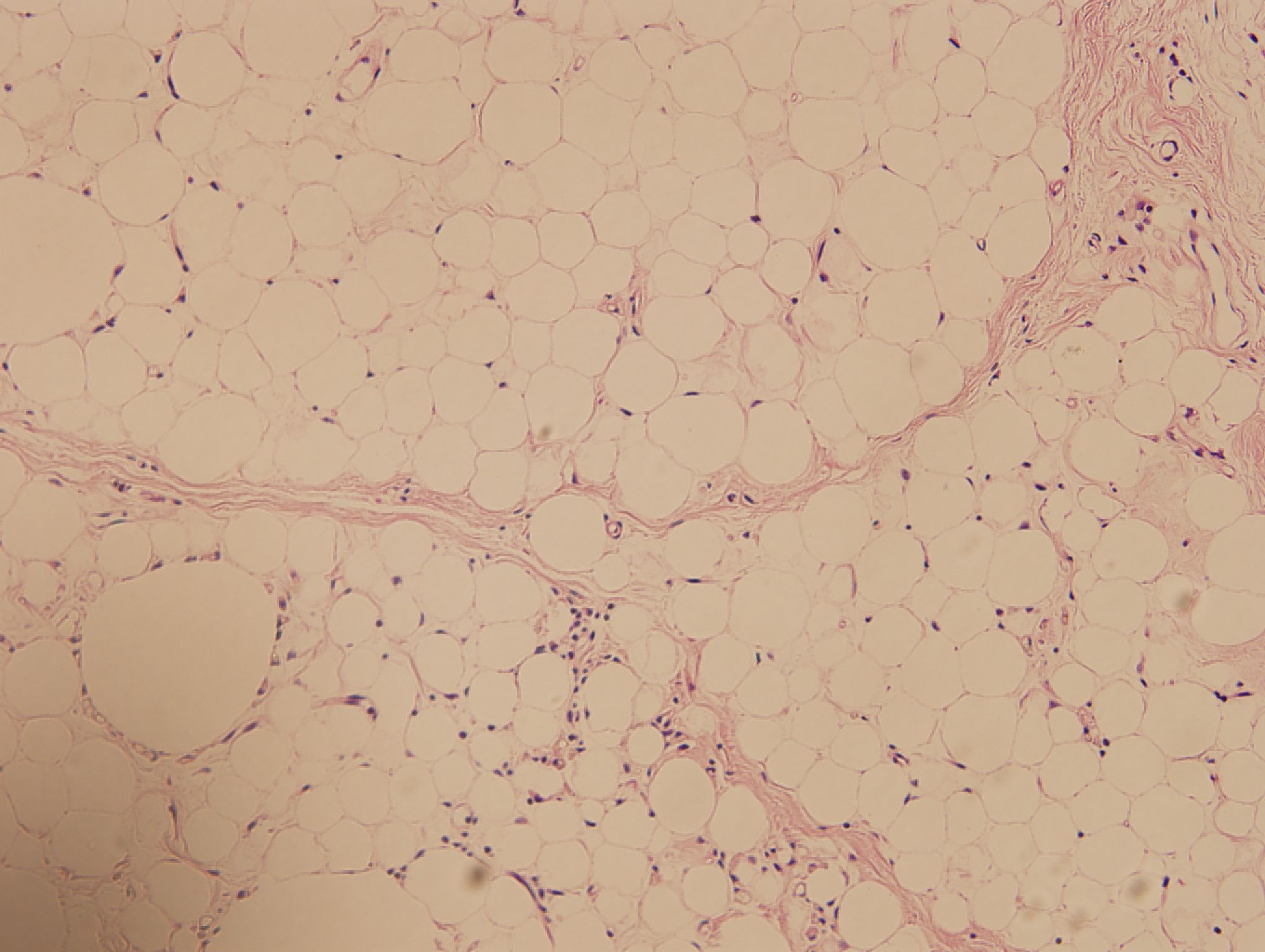

Supplement: Supplementary file 5 [file DataSheet5.ZIP › data for figure 2/ND-HC-HF 2M HE figure/HC-2M HE 20X-20.jpg]

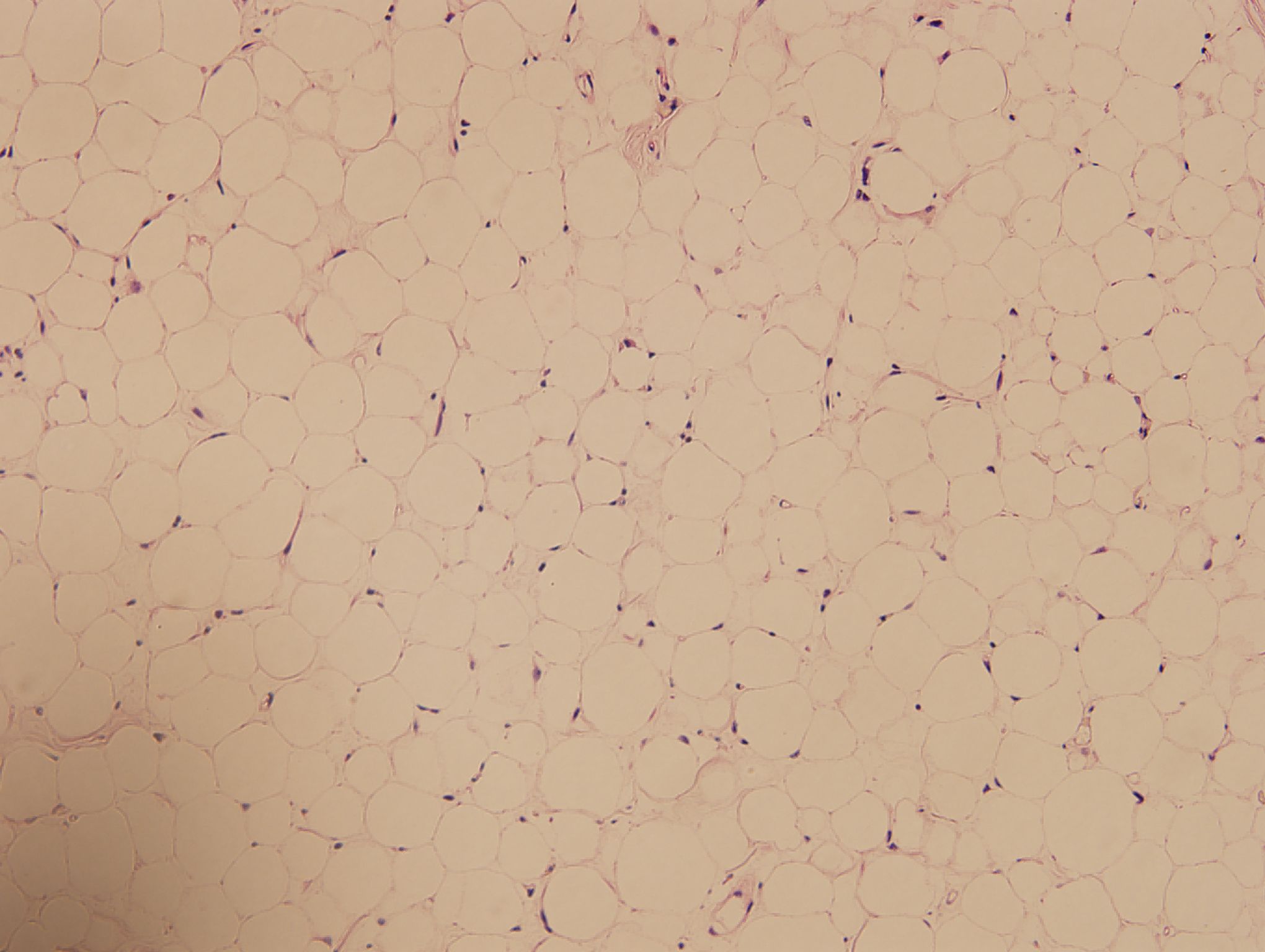

Supplement: Supplementary file 5 [file DataSheet5.ZIP › data for figure 2/ND-HC-HF 2M HE figure/HC-2M HE 20X-21.jpg]

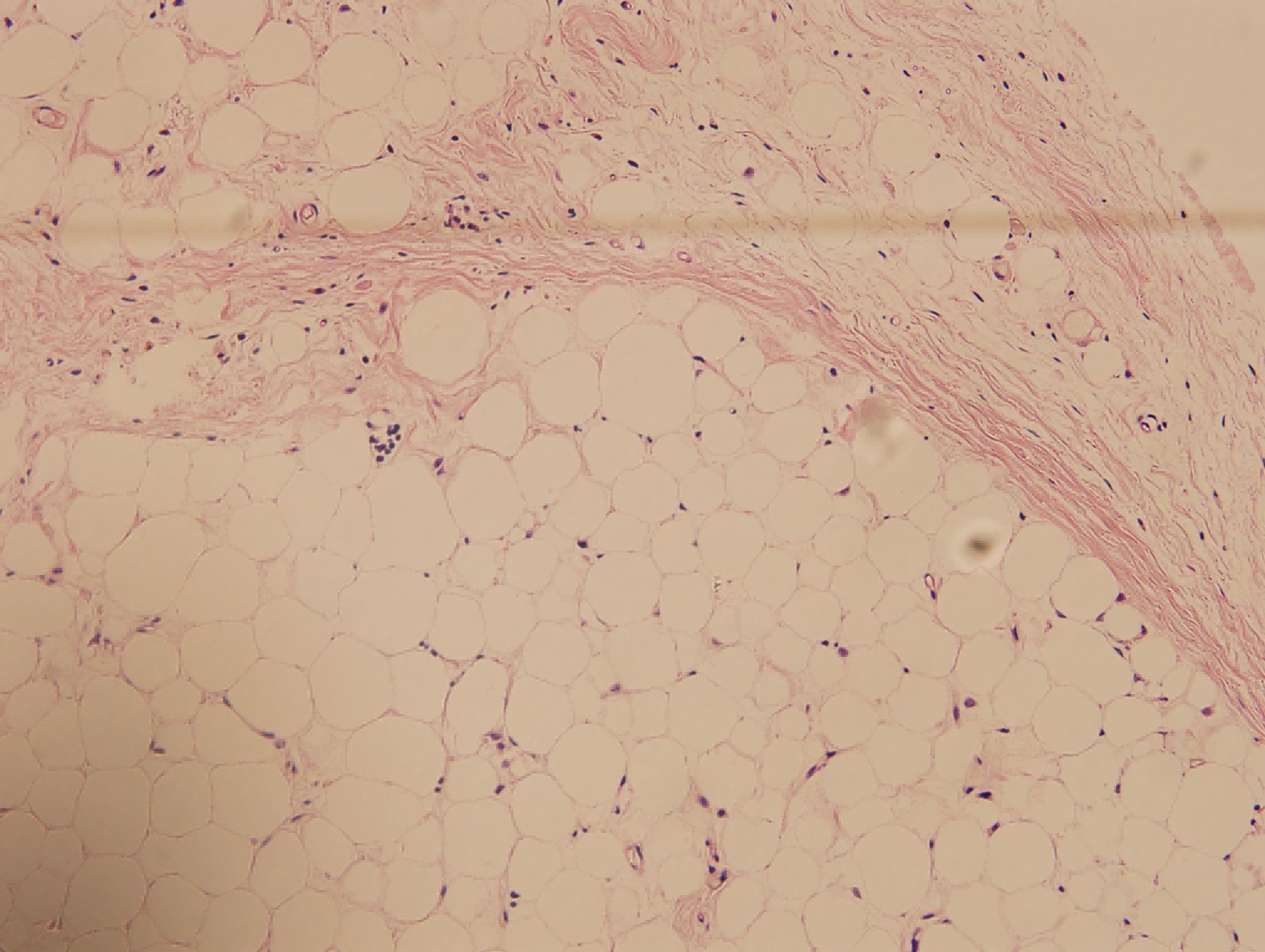

Supplement: Supplementary file 5 [file DataSheet5.ZIP › data for figure 2/ND-HC-HF 2M HE figure/HC-2M HE 20X-22.jpg]

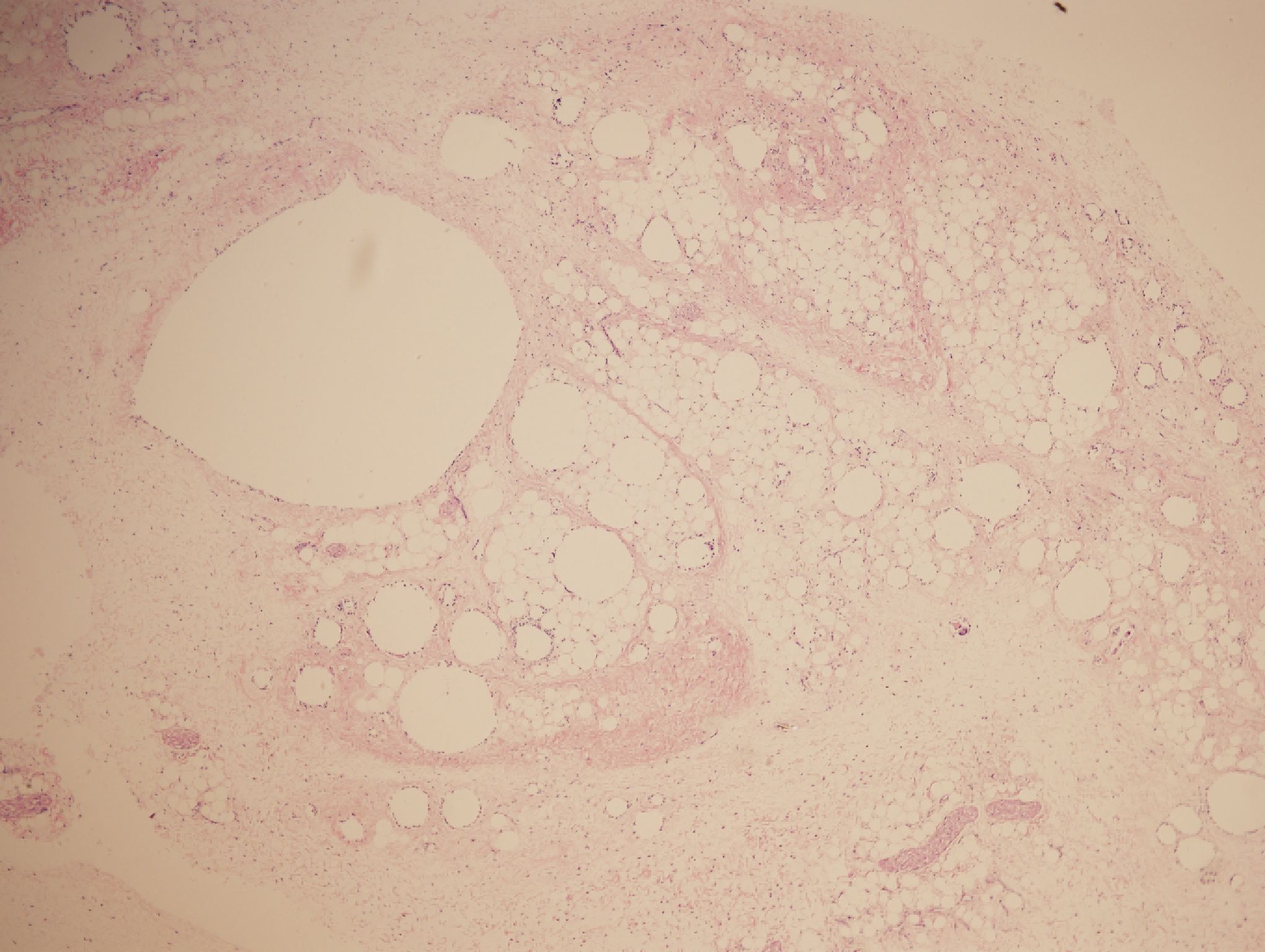

Supplement: Supplementary file 5 [file DataSheet5.ZIP › data for figure 2/ND-HC-HF 2M HE figure/HC-2M HE 4X-09.jpg]

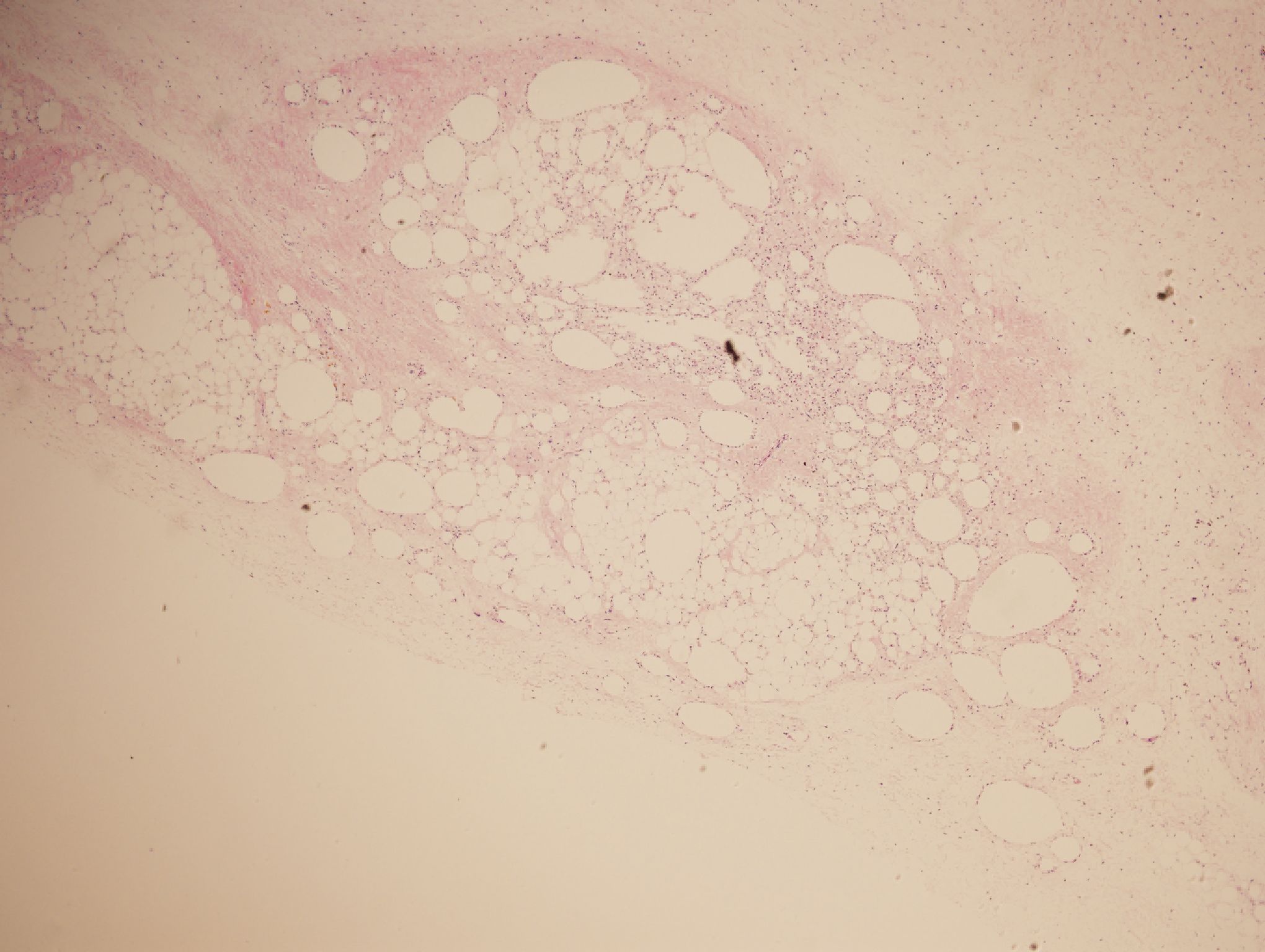

Supplement: Supplementary file 5 [file DataSheet5.ZIP › data for figure 2/ND-HC-HF 2M HE figure/HC-2M HE 4X-10.jpg]

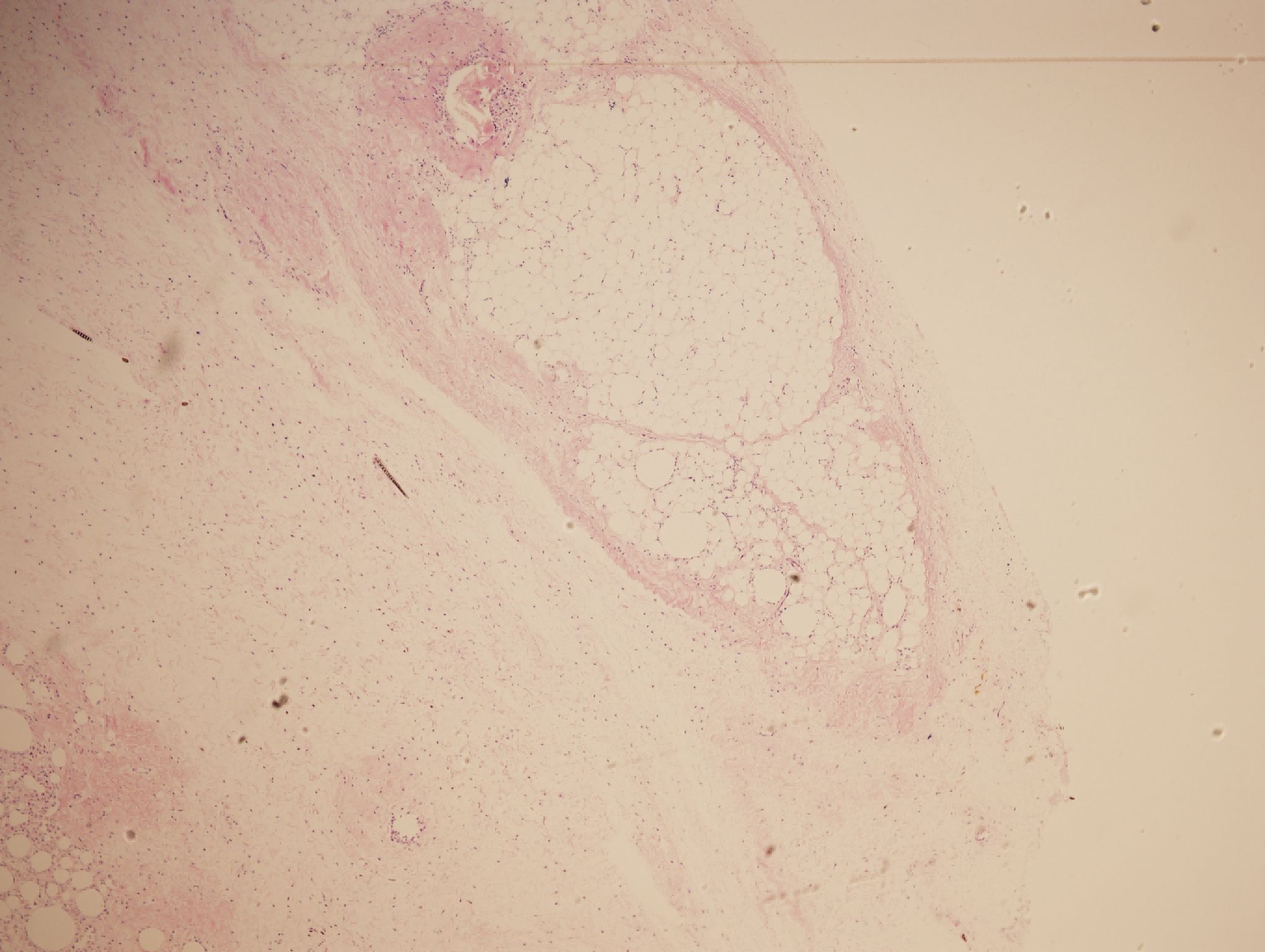

Supplement: Supplementary file 5 [file DataSheet5.ZIP › data for figure 2/ND-HC-HF 2M HE figure/HC-2M HE 4X-11.jpg]

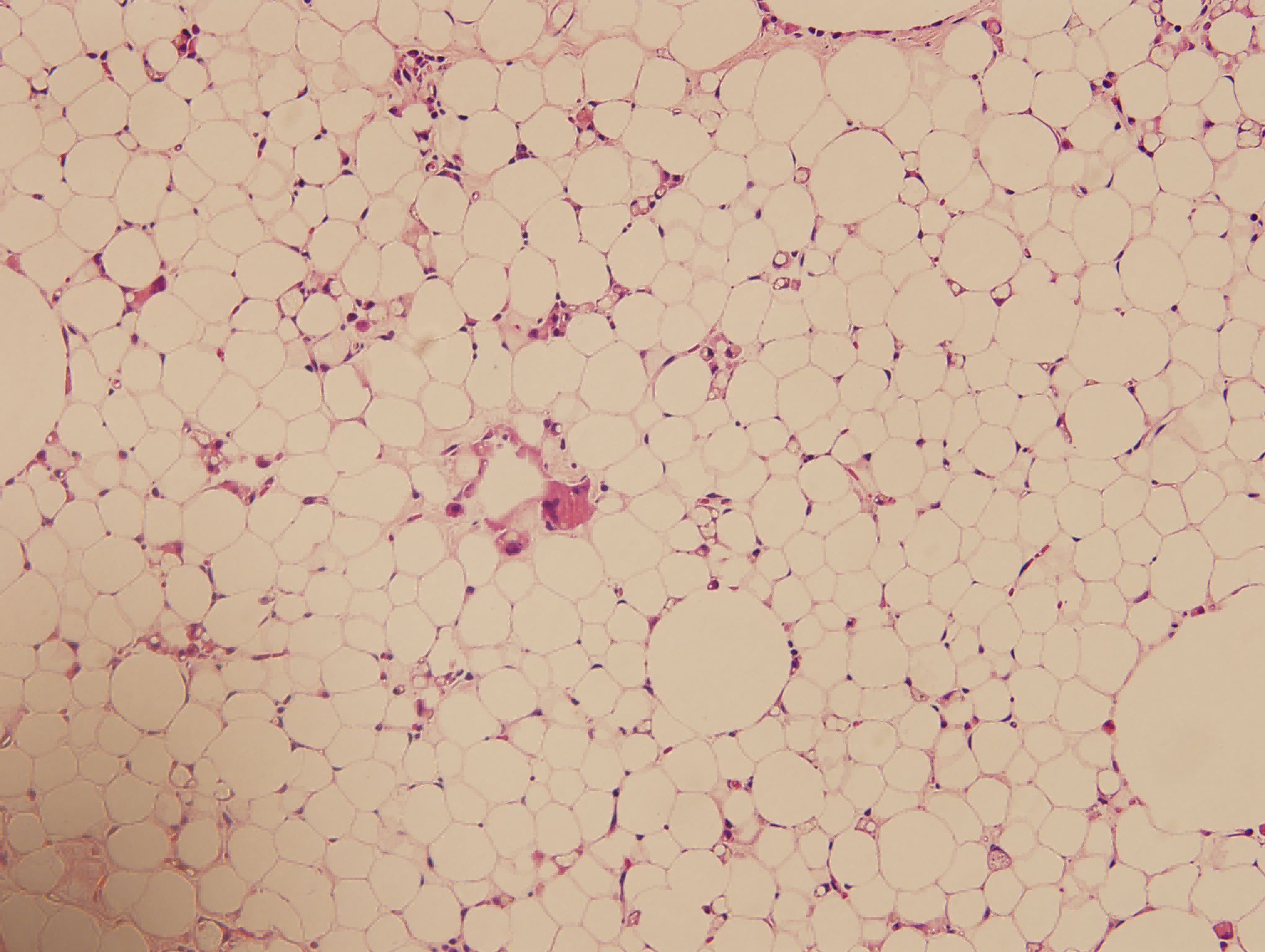

Supplement: Supplementary file 5 [file DataSheet5.ZIP › data for figure 2/ND-HC-HF 2M HE figure/HF-2M HE 20X-10.jpg]

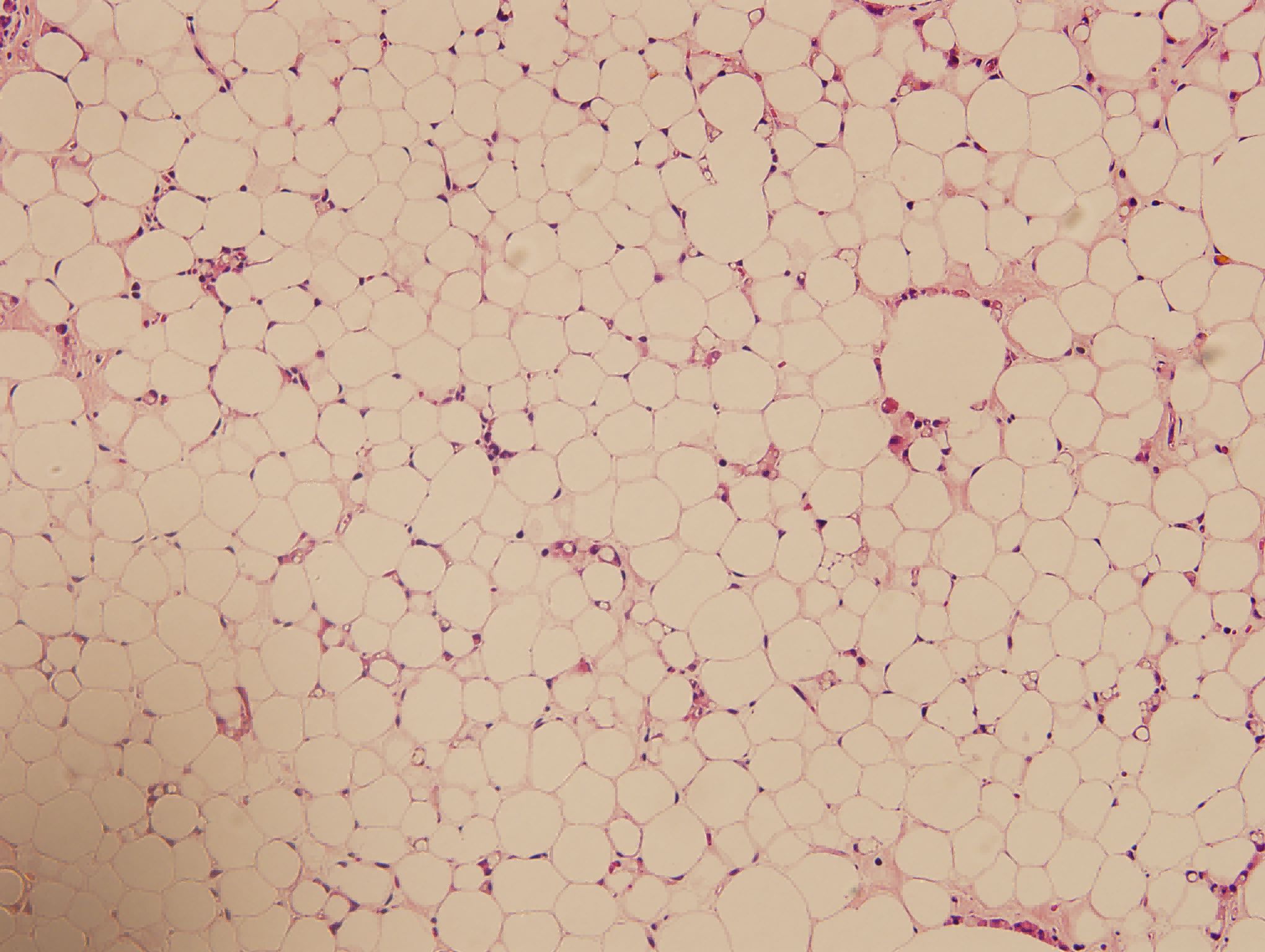

Supplement: Supplementary file 5 [file DataSheet5.ZIP › data for figure 2/ND-HC-HF 2M HE figure/HF-2M HE 20X-11.jpg]

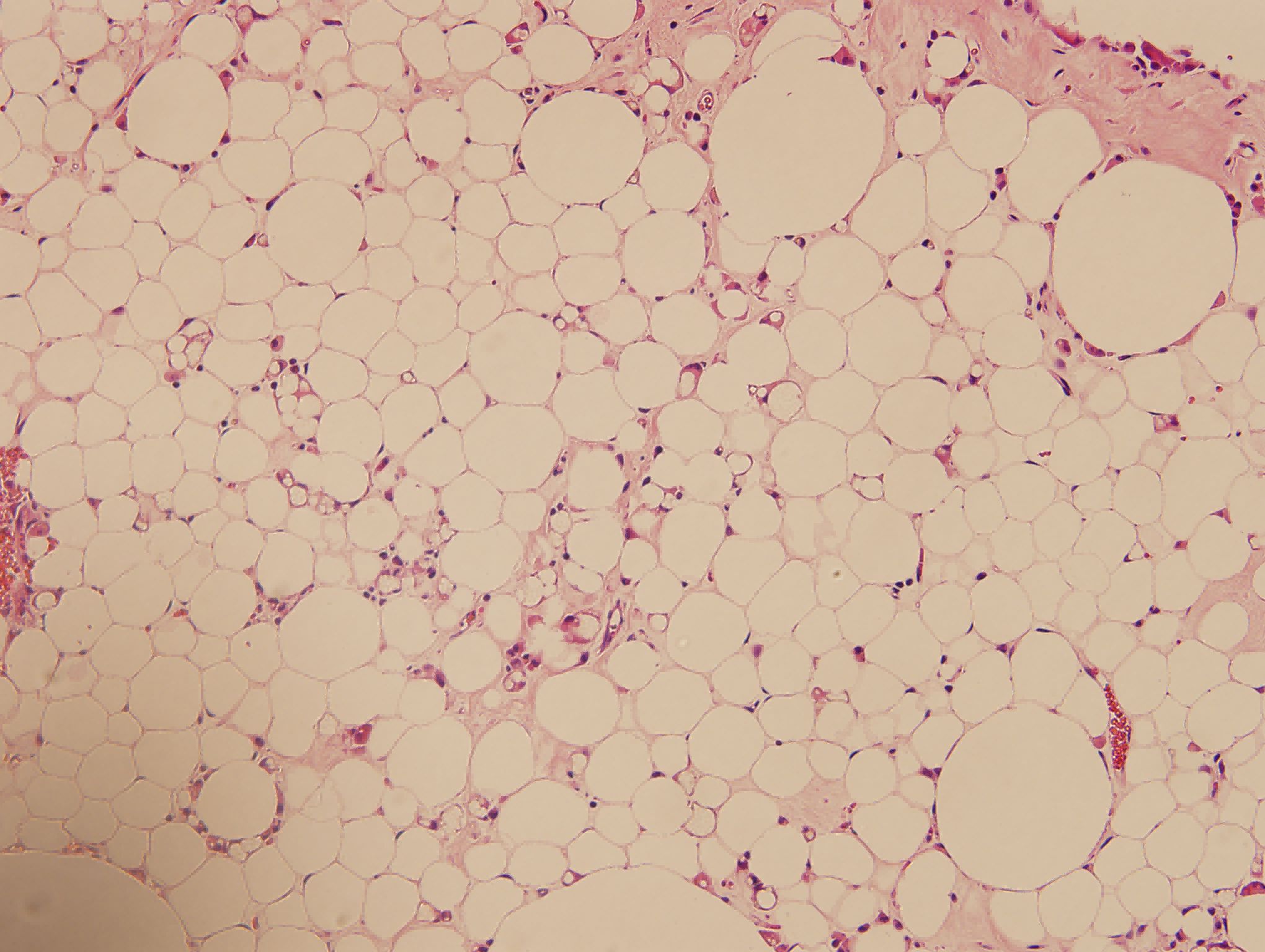

Supplement: Supplementary file 5 [file DataSheet5.ZIP › data for figure 2/ND-HC-HF 2M HE figure/HF-2M HE 20X-12.jpg]

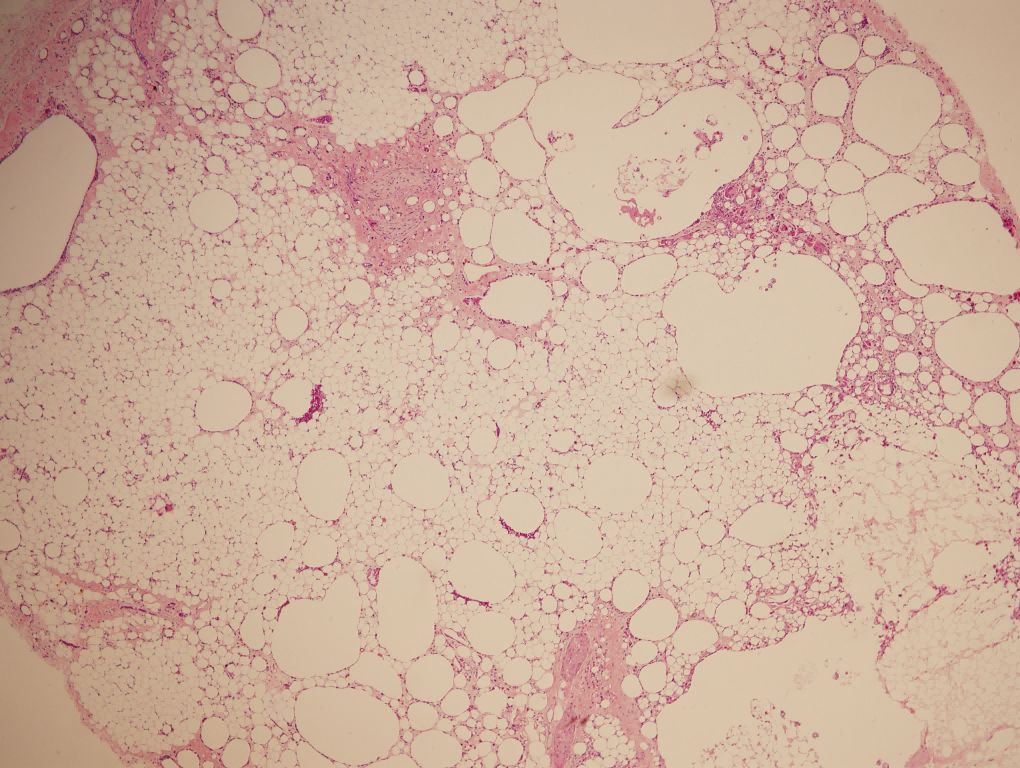

Supplement: Supplementary file 5 [file DataSheet5.ZIP › data for figure 2/ND-HC-HF 2M HE figure/HF-2M HE 4X-01.jpg]

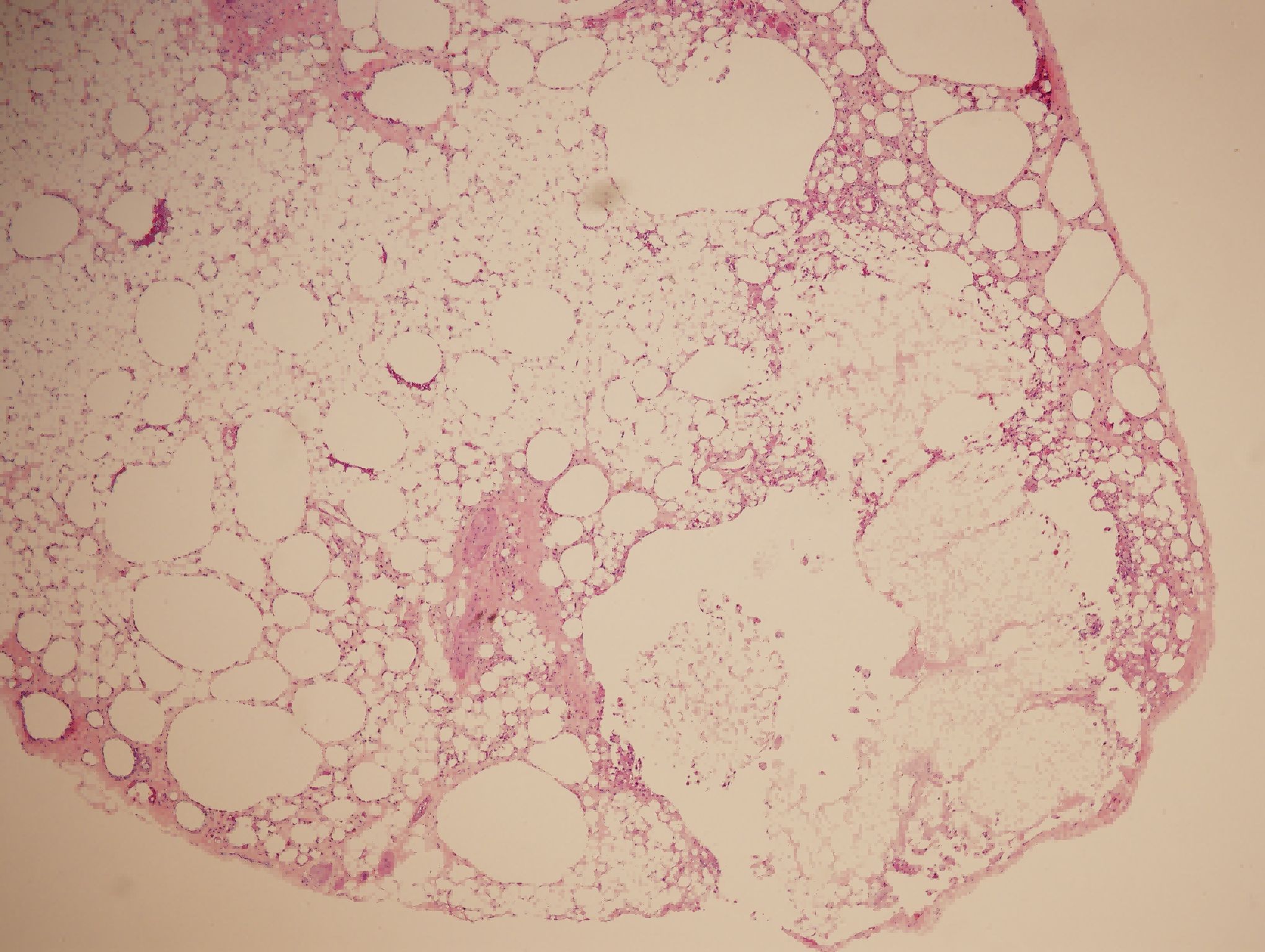

Supplement: Supplementary file 5 [file DataSheet5.ZIP › data for figure 2/ND-HC-HF 2M HE figure/HF-2M HE 4X-02.jpg]

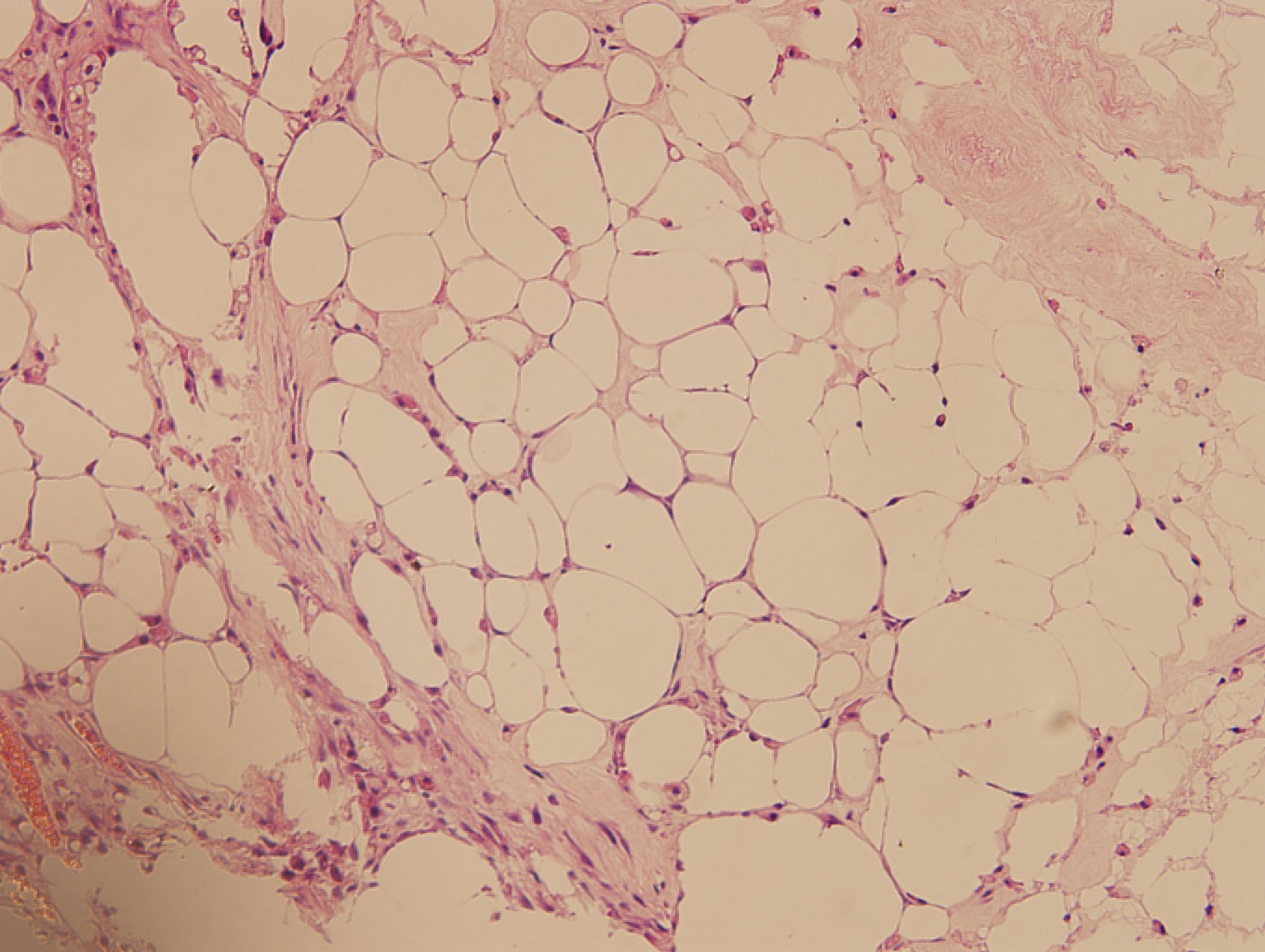

Supplement: Supplementary file 5 [file DataSheet5.ZIP › data for figure 2/ND-HC-HF 2M HE figure/ND-2M HE 20X-06.jpg]

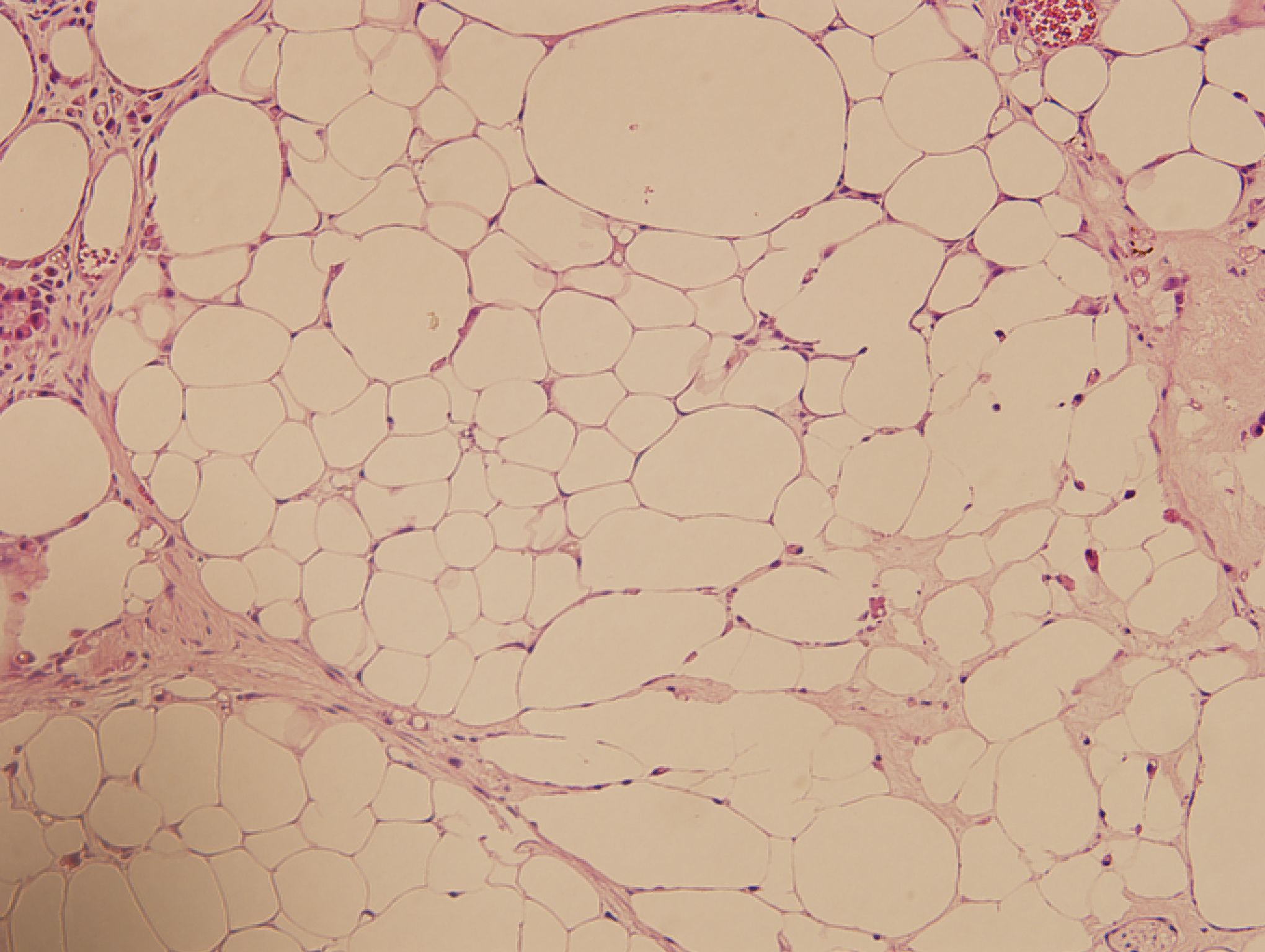

Supplement: Supplementary file 5 [file DataSheet5.ZIP › data for figure 2/ND-HC-HF 2M HE figure/ND-2M HE 20X-07.jpg]

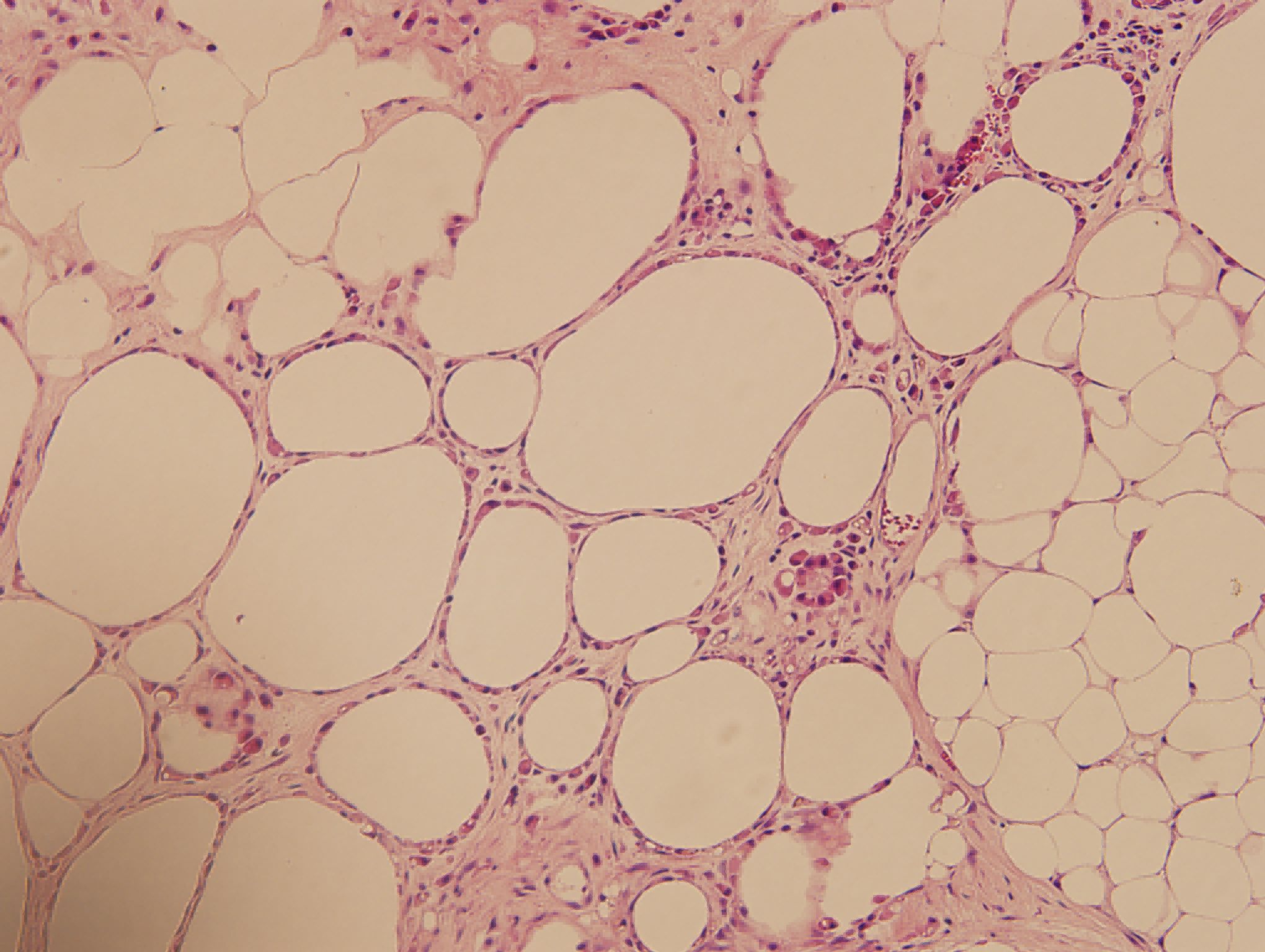

Supplement: Supplementary file 5 [file DataSheet5.ZIP › data for figure 2/ND-HC-HF 2M HE figure/ND-2M HE 20X-08.jpg]

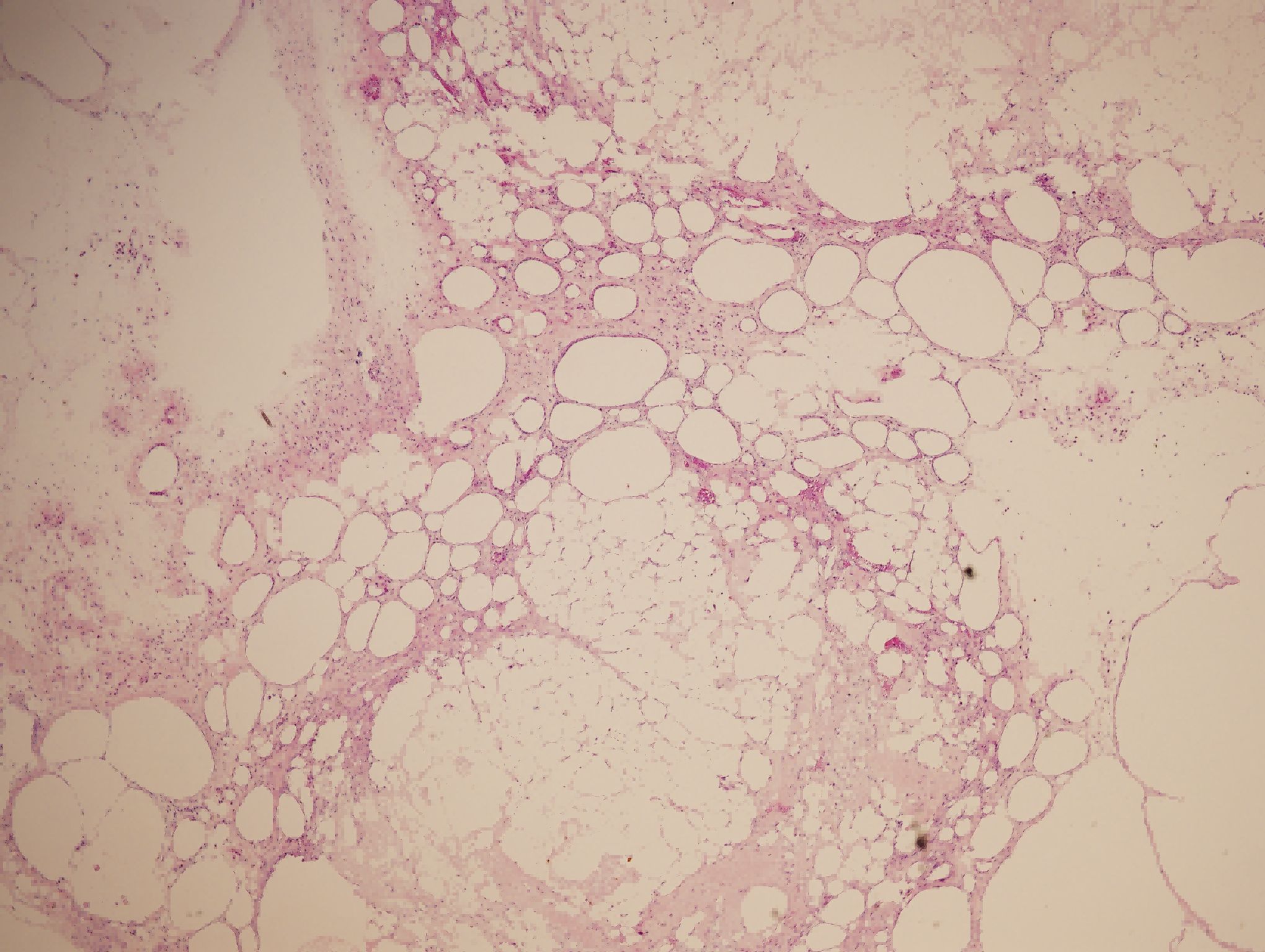

Supplement: Supplementary file 5 [file DataSheet5.ZIP › data for figure 2/ND-HC-HF 2M HE figure/ND-2M HE 4X-01.jpg]

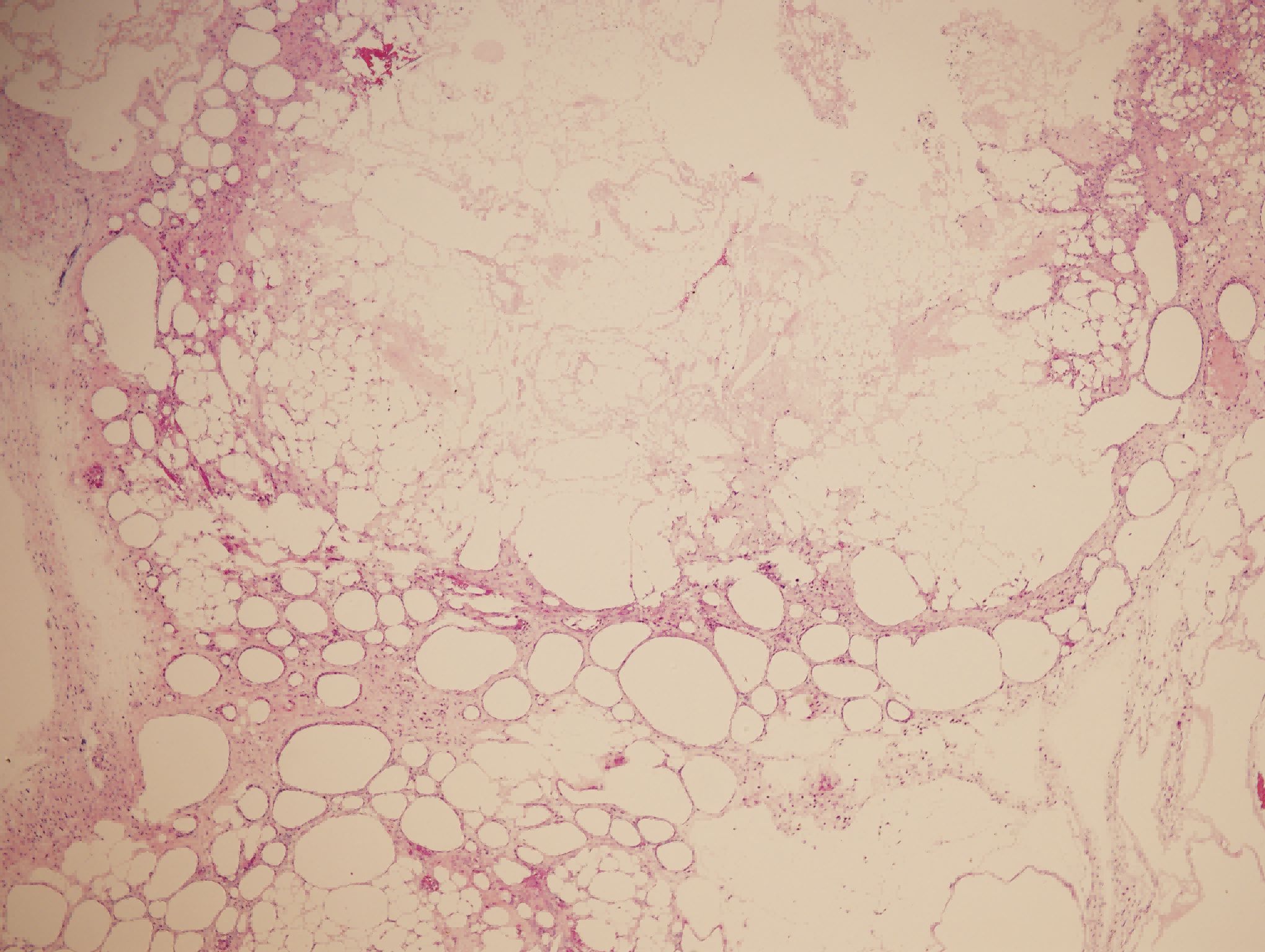

Supplement: Supplementary file 5 [file DataSheet5.ZIP › data for figure 2/ND-HC-HF 2M HE figure/ND-2M HE 4X-02.jpg]
